# Global burden of chronic kidney disease and risk factors, 1990–2021: an update from the global burden of disease study 2021

**DOI:** 10.3389/fpubh.2025.1542329

**Published:** 2025-07-24

**Authors:** Lanhui Wang, Yining He, Chao Han, Peiqi Zhu, Yaping Zhou, Ruijie Tang, Weiming He

**Affiliations:** ^1^Division of Nephrology, Affiliated Hospital of Nanjing University of Chinese Medicine, Jiangsu Province Hospital of Chinese Medicine, Nanjing, China; ^2^Yancheng Dafeng Hospital of Chinese Medicine, Teaching Hospital of Nanjing University of Chinese Medicine, Yancheng, China

**Keywords:** chronic kidney disease, diabetes mellitus, epidemiology, global burden of disease study, glomerulonephritis, hypertension, risk factors

## Abstract

**Introduction:**

The burden of chronic kidney disease (CKD) varies across regions. This study provides comprehensive global, regional, and national estimates of total CKD and CKD due to four specific etiologies from 1990 to 2021.

**Methods:**

Data were extracted from the 2021 Global Burden of Disease study, categorized by sex, 20 age groups, 204 countries or territories, and 5 sociodemographic index (SDI) regions. Age-standardized incidence rates (ASIRs), age-standardized prevalence rates (ASPRs), age-standardized death rates (ASDRs), age-standardized disability-adjusted life year rates (ASDARs) and risk factor burdens for total CKD and four etiology-specific types were analyzed. Temporal trends were assessed using the estimated annual percentage change.

**Results:**

In 2021, CKD remained a significant global burden, with 673 million prevalent cases and 1.5 million deaths, primarily due to metabolic risk factors. The fastest growth in the ASPR and ASIR occurred in the middle-SDI regions, whereas the highest ASDR and ASDAR were observed in low-SDI regions. From 1990 to 2021, global ASIR increased for CKD caused by all four specific etiologies. The ASDR and ASDAR increased for CKD due to type 2 diabetes, glomerulonephritis, and hypertension, whereas there was a decline in CKD due to type 1 diabetes.

**Discussion:**

Between 1990 and 2021, CKD-related disability-adjusted life years (DALYs) and deaths increased substantially, with type 2 diabetes and hypertension accounting for half of the etiology-specific DALYs in 2021. Effective health policies are urgently needed to address CKD risk factors and implement prevention strategies.

## 1 Introduction

As the global population ages, chronic kidney disease (CKD) has emerged as a critical public health crisis. In 2017, CKD affected approximately 9.1% of the global population, equating to 697.5 million cases, with India and China accounting for nearly one-third of the total cases (1). CKD compromises kidney function and leads to severe complications, such as cardiovascular diseases, anemia, bone disorders, metabolic acidosis, and hypertension, significantly impacting health and increasing mortality risks (2, 3). Recent projections suggest that CKD-related deaths may double to 4 million by 2040 (1), underscoring the urgent need for effective preventive and interventional strategies.

The global burden of CKD significantly varies due to differences in etiologies and risk factors. In developed countries, diabetes and hypertension are predominant causes, while glomerulonephritis and diseases of unknown origin are more prevalent in Asia and sub-Saharan Africa (4). Environmental factors, such as exposure to heavy metals and high levels of particulate matter of 2.5 μm, further elevate CKD risk (5). Despite its high prevalence and mortality, awareness of CKD remains critically low, with only 10% of at-risk individuals globally aware of their condition (6). This highlights the urgent need to improve public awareness and promote early screening initiatives.

Previous studies have largely focused on the general state of CKD or examined the effects of individual causes and risk factors in isolation, lacking integration and comparative insights. In this study, we aimed to address this gap by analyzing the prevalence of total CKD and CKD due to 4 specific etiologies and 15 risk factors, using data from the latest Global Burden of Disease (GBD) 2021 study (7–9). Additionally, we perform stratified analyses globally, regionally, and nationally, disaggregated by sex, age, and sociodemographic index (SDI), to inform more targeted prevention and control strategies.

## 2 Materials and methods

### 2.1 Overview

The GBD database, led by the Institute for Health Metrics and Evaluation at the University of Washington, represents the most extensive global health data resource to date. It provides detailed health data between 1990 and 2021 across 204 countries and territories worldwide. The database encompasses 371 diseases and injuries and 88 risk factors (8, 9). Methodologies for estimating the burden of disease and risk factors have been described in the literature. This study was adherent to the Guidelines for Accurate and Transparent Health Estimates Reporting (10). Additionally, this research did not require ethical approval as we utilized available data from the GBD 2021 study. This invaluable resource is pivotal for deciphering and tackling the complexities of global health challenges. It serves as a critical tool for policymakers, researchers, and health professionals in their quest to improve well-being worldwide.

### 2.2 Data source

We gathered data from the Global Health Data Exchange query tool,^1^ including annual incidence, prevalence, death, disability-adjusted life years (DALYs) cases, the age-standardized rates (ASRs) for incidence, prevalence, death, and DALYs of CKD, with 95% uncertainty intervals (UIs) according to country, region, etiology, age, and sex between 1990 and 2021. The data were meticulously organized by dividing ages into 20 cohorts, each spanning 5 years, to facilitate a detailed analysis across various life stages.

### 2.3 SDI

The SDI is a composite measure derived from the geometric mean of lagged income per capita, average years of education, and fertility rate of female individuals aged <25 years. It serves as an indicator of socio-demographic development, with scores ranging from 0 to 1 points. Based on the SDI quintiles ranked from highest to lowest, the 204 countries and territories in the GBD were categorized into 5 tiers: high-, high-middle-, middle-, low-middle-, and low-SDI regions (11). This stratification allowed us to explore the relationship between the CKD burden and the socioeconomic development level.

### 2.4 Estimates of CKD by cause

We utilized two distinct datasets to model the etiological distribution of CKD (9). First, we used data from end-stage kidney disease (ESKD) registries to estimate the proportion of each etiology among patients undergoing renal replacement therapy. The results from all five etiology-specific models were adjusted such that the sum of the estimates for each cause was 100%. Then, these adjusted proportions were applied to DisMod-MR 2.1 model for end-stage renal disease dialysis and transplantation, to generate estimates for each etiology by location, year, age, and sex. The second dataset was obtained from the Geisinger Health System in Pennsylvania. It included age- and sex-specific etiological proportions for identifying patients with CKD stages 1–2, 3, 4, and 5 who were not receiving renal replacement therapy. For each patient with CKD, we used historical records of the International Classification of Diseases (ICD) codes to identify the primary nephropathy ICD codes. Patients with CKD without a history of primary kidney disease ICD code were classified as having CKD of unknown etiology (12).

### 2.5 Risk factors

We assessed the impact of specific risk factors on the total CKD burden and utilized a data-driven approach to determine the risk factor–outcome pairs. For each risk-outcome pair, the relative risks of the given outcome as a function of exposure to the risk factor were estimated. For each risk factor, the overall exposure value representing the risk-weighted exposure prevalence and the theoretical minimum risk exposure level were estimated. The population-attributable fraction was calculated based on these estimates. The population-attributable fractions measured in DALYs were multiplied by the outcomes to determine the attributable years of life lived with a disability, years of life lost, and DALYs (9).

### 2.6 Statistical analysis

In this study, the ASRs based on the GBD global standard population were calculated. Prevalence, incidence, mortality, and DALYs were expressed as predicted values per 100,000 people, accompanied by 95% UI. The 95% UI was estimated using the 2.5th and 97.5th percentile values from 500 draws at each calculation stage (7). Furthermore, to assess the overall trends in the observed disease burden, we computed the estimated annual percentage change (EAPC) for the ASRs for incidence, prevalence, death, and DALYs, evaluating the epidemiological trends between 1990 and 2021. The natural logarithm of the rate is fitted to a linear regression model Y = α + βX + *ε*, where Y is equal to ln (rate), β indicates the positive or negative changing trends, X refers to calendar year, and ε is an error. Thus, the EAPC was calculated as 100 × (eβ − 1), and its 95% confidence interval was derived from the linear regression model. If the EAPC and the lower bound of its 95% UI are both positive, the ASR is considered to exhibit an upward trend. Conversely, if the EAPC and the upper bound of its 95% UI are both negative, the ASR is classified as having a downward trend. If neither condition is met, the ASR is deemed stable (13). All analyses and graphical visualizations were conducted using R statistical computing software (version 3.5.2; R Software for Statistical Computing, Vienna, Austria).

## 3 Results

### 3.1 Total CKD

**Figure 1:**
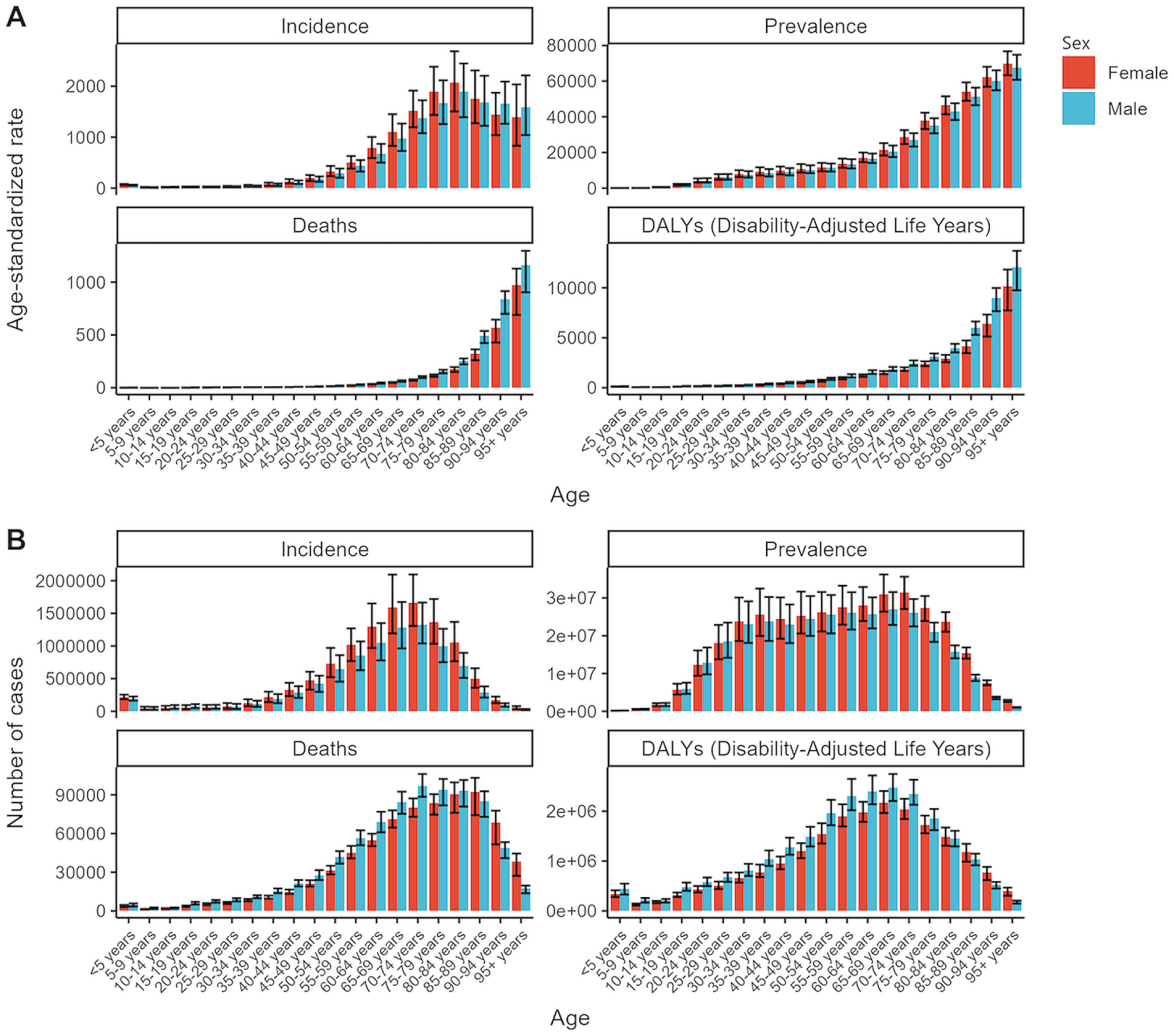
Global age-standardized rates and total numbers for chronic kidney disease metrics by age and sex in 2021. **(A)** Age-standardized rates of incidence, prevalence, deaths, and DALYs. **(B)** Total numbers of incidence, prevalence, deaths, and DALYs. DALYs, disability-adjusted life years.

**Figure 2:**
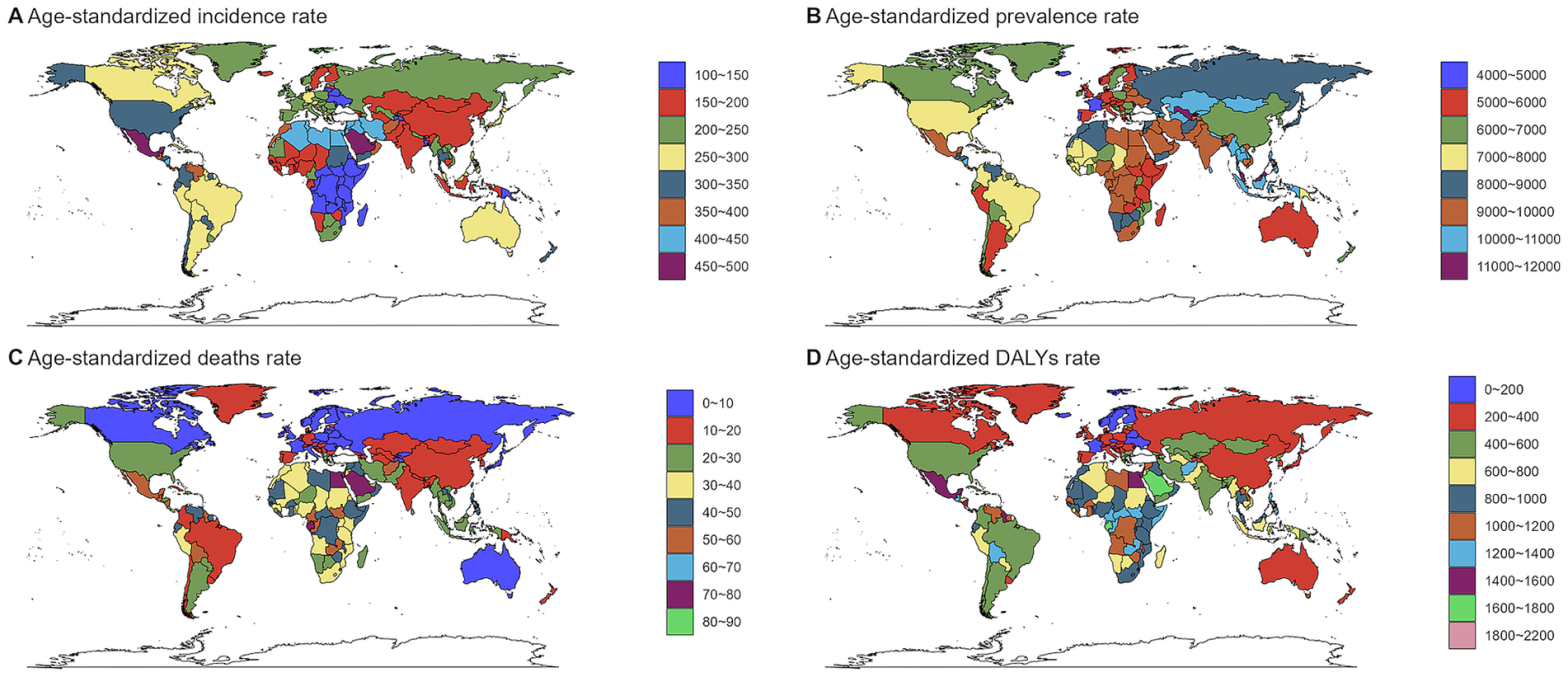
Global burden of chronic kidney disease by age groups, 1990–2021.**(A)** Age-standardized prevalence rates. **(B)**) Age-standardized incidence rates. **(C)** Age-standardized death rates. **(D)** Age-standardized DALYs rates. DALYs, disability-adjusted life years.

**Figure 3:**
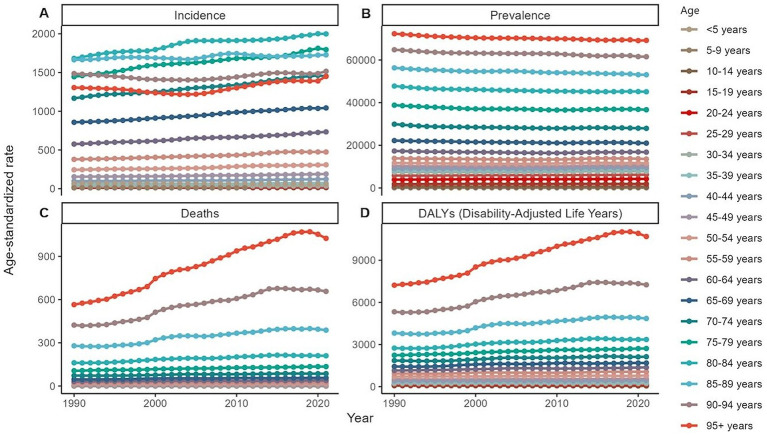
Global burden of chronic kidney disease by age groups, 1990–2021. **(A)** Age-standardized prevalence rates. **(B)** Age-standardized incidence rates. **(C)** Age-standardized death rates. **(D)** Age-standardized DALYs rates. DALYs, disability-adjusted life years.

In 2021, an estimated 673,722,703 individuals globally were affected by CKD (95% UI: 629,095,119–722,364,096), representing a significant rise from 350,962,674 cases in 1990 (95% UI: 326,973,785–376,155,723). Despite this increase, the age-standardized prevalence rate (ASPR) declined from 8072.75 per 100,000 (95% UI: 7560.37–8634.07) in 1990 to 8,006 per 100,000 (95% UI: 7482.12–8575.62) in 2021, with an EAPC of 0.01 (95% UI: −0.02 to 0.04) (Table 1). Female individuals exhibited a higher ASPR for CKD than male individuals did, highlighting a sex-based disparity in prevalence by 2021. The ASPR increased with age, underscoring a positive correlation between advancing age and CKD risk (Figure 1A). Throughout the study period, the prevalence and ASR of CKD were consistently higher in female individuals than in male individuals (Supplementary Figures S2A,B). In 2021, middle- and low-SDI regions, particularly India, reported the highest ASPR, whereas high-SDI regions, notably Iceland, exhibited the lowest rates (Figure 2B; Supplementary Tables S1, S2). Older individuals formed a high-incidence group (Figure 3B; Supplementary Table S3). Among nations, the Republic of Korea demonstrated the greatest ASPR reduction between 1990 and 2021, whereas Guatemala showed the largest increase (Supplementary Table S1). All other SDI regions except the middle-SDI regions saw ASPR declines in 2021 compared to 1990 (Supplementary Table S2; Supplementary Figure S4B).

**Table 1:** The number and age-standardized rates (ASRs) of incidence, prevalence, deaths and disability-adjusted life years of total chronic kidney disease from 1990 to 2021, and estimated annual percentage change of ASRs from 1990 to 2021.

Measure	Year	Age (metric)	Total CKD	Specific cause			
				CKD due to T1DM	CKD due to T2DM	CKD due to glomerulonephritis	CKD due to hypertension
Incidence	1990	All ages (number)	7,790,705 (7,226,165–8,402,568)	63,601 (52,476–76,375)	753,106 (680,930–826,928)	244,229 (218,811–271,857)	463,924 (426,189–505,831)
	2021	All ages (number)	19,935,038 (18,702,793–21,170,794)	95,140 (82,237-111,471)	2,012,025 (1,857,800–2,154,288)	357,288 (329,226–388,483)	1,282,205 (1,195,230–1,366,296)
	1990	ASR (per 100,000)	192.16 (178.69–207.34)	1.1 (0.92–1.31)	19.07 (17.28–20.83)	4.3 (3.88–4.75)	12.24 (11.31–13.33)
	2021	ASR (per 100,000)	233.56 (220.02–247.24)	1.31 (1.12–1.55)	23.07 (21.4–24.72)	4.84 (4.42–5.29)	14.97 (14.02–15.93)
	1990–2021	EAPC (%)	0.64 (0.63–0.65)	0.69 (0.64–0.74)	0.61 (0.6–0.63)	0.39 (0.36–0.43)	0.66 (0.66–0.67)
Prevalence	1990	All ages (number)	350,962,674 (326,973,785–37,615,5,723)	2,967,857 (2,607,069–3,328,285)	58,105,268 (53,056,992–63,286,818)	6,370,882 (5,926,723–6,848,154)	11,712,345 (10,891,658–12,623,876)
	2021	All ages (number)	67,372,2,703 (629,095,119–722,364,096)	6,295,711 (5,459,693–7,114,345)	107,559,955 (99,170,797–115,994,732)	10,735,809 (9,925,500–11,520,171)	24,467,653 (22,861,634–26,230,869)
	1990	ASR (per 100,000)	8,072.75 (7,560.37–8,634.07)	57.54 (50.87–64.11)	1,327.22 (1,223.26–1,439.42)	128.55 (119.33–137.58)	310.68 (289.07–333.84)
	2021	ASR (per 100,000)	8,006 (7,482.12–8,575.62)	77.31 (66.91–87.58)	1,259.63 (1,161.99–1,359.92)	129.94 (120.25–139.51)	291.19 (272.49–311.88)
	1990–2021	EAPC (%)	0.01 (−0.02–0.04)	1.36 (1.26–1.47)	−0.17 (−0.2 to −0.14)	0.06 (0.04–0.08)	−0.16 (−0.18 to −0.13)
Deaths	1990	All ages (number)	552,673 (513,463–607,915)	49,300 (39,088–61,208)	147,970 (124,179–176,413)	83,170 (69,625–97,738)	454,359 (381,291–524,688)
	2021	All ages (number)	1,527,639 (1,389,377–1,638,914)	94,020 (71,457–119,984)	477,273 (401,541–565,951)	193,997 (162,332–226,569)	454,359 (381,291–524,688)
	1990	ASR (per 100,000)	14.85 (13.64–16.38)	1.08 (0.84–1.35)	4.15 (3.5–4.94)	2.02 (1.68–2.38)	5.54 (4.68–6.41)
	2021	ASR (per 100,000)	18.5 (16.72–19.85)	1.08 (0.83–1.38)	5.72 (4.83–6.79)	2.34 (1.96–2.74)	5.54 (4.68–6.41)
	1990–2021	EAPC (%)	0.82 (0.76–0.89)	−0.07 (−0.13 to −0.01)	1.17 (1.1–1.24)	0.54 (0.5–0.59)	0.97 (0.91–1.03)
DALYs	1990	All ages (number)	20,739,895 (18,843,684–22,588,533)	2,227,518 (1,835,373–2,679,208)	4,122,919 (3,498,980–4,818,958)	3,751,088 (3,252,292–4,269,460)	4,344,896 (3,676,494–5,110,004)
	2021	All ages (number)	44,453,684 (40,840,762–48,508,462)	3,875,628 (3,062,396–4,845,503)	11,278,935 (9,682,785–13,103,871)	6,959,758 (6,018,414–7,961,673)	10,850,728 (9,207,080–12,320,650)
	1990	ASR (per 100,000)	479.85 (439.18–523.79)	47.05 (38.4–57.32)	105.71 (90.68–122.67)	77.78 (67.62–88.41)	107.77 (91.26–126.92)
	2021	ASR (per 100,000)	529.62 (486.25–577.42)	45.2 (36.01–56.35)	131.08 (112.75–152.49)	84.47 (73.2–96.13)	128.41 (109.14–145.64)
	1990–2021	EAPC (%)	0.37 (0.33–0.41)	−0.21 (−0.28 to −0.15)	0.81 (0.75–0.87)	0.28 (0.25–0.31)	0.63 (0.58–0.67)

Data in parentheses represent 95% uncertainty intervals (95% UIs).

ASR, age-standardized rate; CKD due to T1DM, chronic kidney disease due to diabetes mellitus type 1; CKD due to T2DM, chronic kidney disease due to diabetes mellitus type 2; DALYs, disability-adjusted life years; EAPC, estimated annual percentage change; CKD, chronic kidney disease.

The total number of CKD incident cases in 2021 was approximately 19,935,038 (95% UI: 18,702,793–21,170,794). The age-standardized incidence rate (ASIR) for CKD increased from 192.16 per 100,000 (95% UI: 178.69–207.34) in 1990 to 233.56 per 100,000 (95% UI: 220.02–247.24) in 2021, reflecting an EAPC of 0.64 (95% UI: 0.63–0.65) (Table 1). Similar to the ASPR, the ASIR demonstrated sex and age-related variations, with higher values among female individuals and a bell-shaped distribution across age groups (Figure 1A). Incident cases were predominantly concentrated among older adults (Figure 3A). Compared with 1990, the ASIR increased across all age groups except those under 5 years, with the most pronounced increase observed in the 15–19 age group (Supplementary Table S3). Among 204 countries and 5 SDI regions, China recorded the highest number of incident cases in 2021. In contrast, Saudi Arabia had the highest ASIR (Figure 2A; Supplementary Table S5), and the high-SDI region displayed the highest ASIR, which was comparable to that of other SDI countries (Supplementary Figure S1A). Between 1990 and 2021, Estonia experienced the greatest annual percentage change in the ASIR, whereas Greece recorded the smallest change (Supplementary Table S5). Across all SDI regions, the ASIR in 2021 exceeded that in 1990, with the middle-SDI region showing the largest increase (Supplementary Table S2; Supplementary Figure S4A).

In 2021, CKD accounted for an estimated 1,527,639 deaths globally (95% UI: 1,389,377–1,638,914), marking an increase in the ASDR from 14.85 per 100,000 (95% UI: 13.64–16.38) in 1990 to 18.5 per 100,000 (95% UI: 16.72–19.85). This rise corresponds to an EAPC of 0.82 (95% UI: 0.76–0.89) (Table 1). Throughout the period, male individuals consistently exhibited higher ASDRs than female individuals did, with significant increases for both sexes compared to 1990 (Supplementary Figure S2A; Supplementary Table S6). Mortality rates were highest among the super-aged population, which also experienced the largest increases in the ASR (Figures 1A,B, 3C). In 2021, Mauritius had the highest ASDR, whereas Belarus reported the lowest (Supplementary Table S7). Low-SDI regions had the highest ASDR, whereas middle-SDI regions accounted for the largest absolute number of deaths (Supplementary Figures S1A,B). Compared to 1990, the United States experienced the fastest ASDR growth, whereas the greatest decrease was observed in Poland (Figure 2C; Supplementary Table S7). Across all SDI regions, the ASDR for CKD significantly increased between 1990 and 2021 (Supplementary Table S2; Supplementary Figure S4C).

The global burden of CKD in 2021 was further underscored by the DALYs, with an ASR of 529.62 per 100,000 (95% UI: 486.25–577.42). This represents a notable increase from 1990, with an annual percentage change of 0.37 (95% UI: 0.33–0.41) (Table 1). Male individuals had higher DALYs and ASDAR than female individuals did, with the sex disparity widening with age, particularly among individuals over 90 years (Figure 1A). The ASR of DALYs increased with age, and trends since 1990 reveal contrasting patterns: among individuals aged <35 years, younger age groups experienced steeper declines in the ASR, whereas for those aged >35 years, the ASR positively increased with age (Figure 3D; Supplementary Table S3). Low-SDI regions exhibited the highest ASR of DALYs, whereas Finland reported the lowest rates. Middle-SDI regions, particularly India, bore the highest burden in terms of absolute DALYs attributed to CKD (Figure 2D; Supplementary Tables S2, S8). Between 1990 and 2021, Lesotho and Ethiopia recorded the fastest increase in the ASR of DALYs among the 204 countries and 5 SDI regions, whereas Poland achieved the most significant decline (Supplementary Table S8). Within the SDI spectrum, reductions in the ASR of DALYs were noted in low-and upper-middle-SDI regions, whereas high-SDI regions experienced the largest increases (Supplementary Table S2; Supplementary Figure S4D).

### 3.2 CKD due to type 1 diabetes mellitus

**Figure 4:**
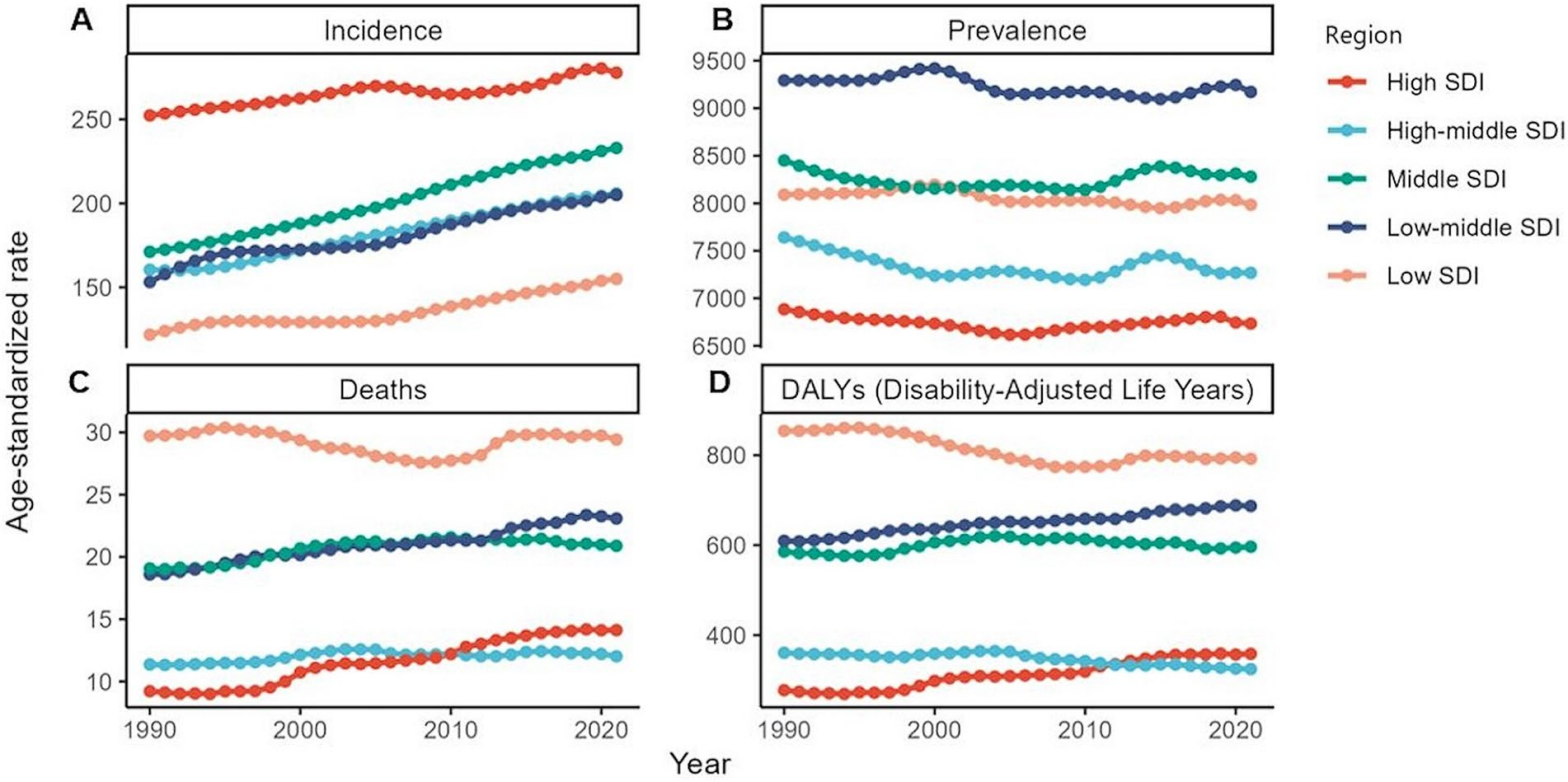
Global burden of chronic kidney disease by SDI quintile, 1990–2021. **(A)** Age-standardized prevalence rates. **(B)** Age-standardized incidence rates. **(C)** Age-standardized death rates. **(D)** Age-standardized DALYs rates. DALYs, disability-adjusted life years.

In 2021, CKD due to type 1 diabetes mellitus (T1DM) affected an estimated 6,295,711 individuals worldwide (95% UI: 5,459,693–7,114,345), with 95,140 new cases (95% UI: 82,237–111,471) identified during the same year (Table 1). Across all age groups, female individuals exhibited a higher prevalence of CKD due to T1DM, compared with male individuals, with the most pronounced disparity observed in adolescents aged 20–34 years (Figure 4). Conversely, male individuals had a higher incidence of new cases. Notably, the incidence rate among infants and young children aged 0–5 years was significantly higher than in other age groups (Supplementary Figures S3A,B). Male patients also recorded higher ASDR and age-standardized DALY rates (ASDAR) than female patients did, particularly in the 45–64-year-old population (Supplementary Figure S3A). Among countries, Belarus reported the highest ASIR, Canada had the highest ASPR, and the United States recorded the highest ASDR and ASDAR (Supplementary Tables S10–S13). The middle-SDI regions led in ASR metrics for deaths and DALYs (Supplementary Table S14).

Globally, a comparison with 1990 data revealed an increase in both the ASIR and ASPR by 2021, whereas the ASDR and ASDAR showed a decline, albeit with some fluctuations around 2000 (Supplementary Table S15). Between 1990 and 2021, the 0–5-year age group consistently exhibited high numbers of new cases. For ASPR, a declining trend was observed exclusively in the population under 15 years of age (Supplementary Table S9). Over this period, male individuals consistently recorded higher ASIR, ASDR, and ASDAR than did female individuals. However, a downward trend in the ASDR and ASDAR among female individuals was observed during the survey period (Supplementary Table S15). By 2021, the United States experienced the most rapid escalation in the ASDR and ASDAR compared to 1990 (Supplementary Tables S12, S13). Across all SDI regions, both the ASIR and ASPR increased. Notably, only the high-and lower-middle-SDI regions displayed slight increases in the ASDR and ASDAR (Supplementary Table S14).

### 3.3 CKD due to type 2 diabetes mellitus

In 2021, CKD due to type 2 diabetes mellitus (T2DM) was the leading global cause of CKD, affecting approximately 107,559,955 individuals (95% UI: 99,170,797–115,994,732), with 2,012,025 new cases identified that year (95% UI: 1,857,800–2,154,288). This condition contributed to 477,273 deaths (95% UI: 401,541–565,951) and 11,278,935 DALYs (95% UI: 9,682,785–13,103,871) (Table 1). CKD due to T2DM was rare among individuals aged <15 years; however, elevated ASMR and ASDAR were observed in the older population (Supplementary Table S16). A consistent sex difference was observed, with male individuals exhibiting higher ASRs across incidence, prevalence, mortality, and DALYs compared with female individuals (Supplementary Table S17).

Geographically, regions in North Africa and Western Asia reported higher ASIR and ASPR, whereas high-middle-SDI regions had the lowest ASDR and ASDAR (Supplementary Tables S18–S20). Conversely, high-SDI regions exhibited the highest ASIR, highlighting regional disparities (Supplementary Table S18). Between 1990 and 2021, CKD due to T2DM saw significant increases in the ASRs for incidence, mortality, and DALYs, with EAPCs of 0.61 (95% UI: 0.60–0.63), 1.17 (95% UI: 1.10–1.24), and 0.81 (95% UI: 0.75–0.87), respectively. However, the ASPR showed a modest decline of 0.17 (95% UI: 0.14–0.20) over the same period (Table 1). Across age groups, only individuals < 40 years exhibited declining trends in ASDAR and ADMR, while the most significant increase observed among individuals ≥ 80 years. For ASIR and ASPR, the burden decreased among individuals aged 15–34 years (Supplementary Table S16). Over the past three decades, males consistently demonstrated higher ASIR, ASPR, ASMR, and ASDAR values compared with females, along with a greater magnitude of changes in these indicators (Supplementary Table S17). The United States experienced the most rapid increases in the ASIR, ASDR, and ASDAR, reflecting its evolving burden of CKD due to T2DM (Supplementary Tables S19, S21, S22). High-SDI regions also demonstrated the steepest EAPCs for death and DALYs, a trend that aligns with global patterns observed in other regions (Supplementary Table S18).

### 3.4 CKD due to glomerulonephritis

In 2021, CKD due to glomerulonephritis contributed to a significant health burden, with 6,959,758 DALYs (95% UI: 6,018,414–7,961,673) DALYs. The patient population totaled 10,735,809 (95% UI: 9,925,500–11,520,171) individuals, and the number of new patients was 357,288 (95% UI: 292,260–388,483) (Table 1). New cases were mainly concentrated in the 0–5-year age group and showed a degree of sex difference. Moreover, males exhibited higher overall burden indicators compared with females (Supplementary Table S24). Across all age groups, with the ASMR and ASDAR being significantly elevated among older patients (Supplementary Table S23). Nicaragua and Mauritius had the highest ASIR and ASPR, respectively, whereas the United Republic of Tanzania and El Salvador had the highest ASDR and ASDAR (Supplementary Tables S25–S28). Notably, regions with higher SDI, specifically the high-and high-to-middle-SDI regions, had lower ASDR and ASDAR. In contrast, the middle- and low-to-middle-SDI regions showed higher ASIR and ASPR (Supplementary Table S29).

During 1990–2021, a global uptick was observed in the ASR of incidence, prevalence, death, and DALYs for CKD due to glomerulonephritis, with EAPCs of 0.39 (95% UI: 0.36–0.43), 0.06 (95% UI: 0.04–0.08), 0.54 (95% UI: 0.50–0.59), and 0.28 (95% UI: 0.25–0.31), respectively (Table 1). The ASIR showed an upward trend across all age groups, with the 0–5-year age group leading in the ASIR despite a decline from the 1990 levels. A decrease was also observed in the ASPR, ASDR, and ASDAR for the 0–14-year age group (Supplementary Table S23). Males exhibited greater increases in ASIR and ASDAR compared with females, whereas the growth trends for ASPR and ASMR were lower in males than in females (Supplementary Table S24). Among the 204 GBD countries, South Korea achieved the most notable reduction in the ASDR and ASDAR, with EAPCs of −4.67 (95% UI: −5.43 to −3.91) and −4.58 (95% UI: −5.21 to −3.94), respectively (Supplementary Tables S27, S28). Compared with 1990, all SDI regions exhibited an increase in the ASIR, with the ASPR remaining relatively stable, and only the high-middle- and low-SDI regions reported a decrease in the ASDR and ASDAR (Supplementary Table S29).

### 3.5 CKD due to hypertension

In 2021, CKD due to hypertension emerged with 1,282,205 (95% UI: 1,195,230–1,366,296) new cases and 24,467,653 (95% UI: 22,861,634–26,230,869) individuals. This condition contributed to 10,850,728 (95% UI: 1,195,230–1,366,296) DALYs (Table 1). A notable sex difference that increased with age was observed in the ASIR and ASPR (Supplementary Figures S4A,B), and the ASDR and ASDAR were predominantly concentrated among older and super-aged patients (Supplementary Table S30). Mauritius exhibited the highest ASRs for both death and DALYs, whereas the United Arab Emirates and Nicaragua reported the highest ASIR and ASPR, respectively (Supplementary Tables S31–S34). A minimal variation was observed in the ASPR across all SDI regions, with the high-SDI region showing the highest ASIR and the low-SDI region bearing the highest ASDR and ASDAR (Supplementary Table S4).

Compared to 1990, the ASPR for CKD due to hypertension saw a slight decline in 2021 by 0.16 (95% UI: 0.13–0.18). In contrast, the ASRs of incidence, death, and DALYs exhibited growth, with respective EAPCs of 0.66 (95% UI: 0.66–0.67), 0.97 (95% UI: 0.91–1.03), and 0.63 (95% UI: 0.58–0.67) (Table 1). Female individuals experienced greater changes in these metrics than male individuals did, among these metrics, only the ASPR exhibited a declining trend (Supplementary Table 35). In 2021, the ASIR increased across all age groups, compared with 1990, whereas a decrease was observed in the ASDR and ASDAR for individuals aged <35 years. Additionally, during the study period, the ASPR exhibited an upward trend among individuals aged 25–44 years (Supplementary Table S30). Among the 204 GBD countries, Ireland was the sole nation to record a decrease in the ASIR, with an EAPC of −0.09 (95% UI: −0.14 to −0.04) (Supplementary Table S31). The United States experienced the most rapid increase in the ASDR (Supplementary Table S33). All other SDI regions witnessed an increase in the ASDR and ASDAR except for the low-SDI region, with the high-SDI region showing the most significant increase (Supplementary Table S4).

### 3.6 Risk factors of total CKDs

Globally, CKD can be attributed to a constellation of 15 identifiable risk factors, which include three main categories: environmental factors, including high temperature, low temperature, and lead exposure; behavioral factors, such as a diet low in fruits, whole grains, and vegetables; diet high in red meat, sugar-sweetened beverages, sodium, and processed meats; and low physical and metabolic factors, including high fasting plasma glucose, high systolic blood pressure, high body mass index (BMI), and impaired kidney function. These factors collectively illuminate the complex interplay contributing to CKD, emphasizing the necessity of a holistic strategy for its prevention and treatment.

In 2021, the leading risk factors contributing to ASDAR in male patients with total CKD were impaired kidney function, high fasting plasma glucose levels, high systolic blood pressure, high BMI, and a low-fruit diet. For female individuals, the risk factors were largely aligned, with impaired kidney function and high fasting plasma glucose at the forefront, followed by high BMI, high systolic blood pressure, and a low-fruit diet. Notably, female individuals were more significantly affected by a high BMI than were male individuals (25.0% vs. 21.3%). These conclusions are also reflected in the ASDR (Supplementary Table S36). Across different SDI regions, the four most prevalent attributable risk factors for total CKD were consistent in both the ASDR and ASDAR: impaired kidney function, high fasting plasma glucose, high BMI, and high systolic blood pressure. Low-SDI regions were least affected by high BMI but were disproportionately impacted by impaired kidney function, indicating a stronger association with this risk factor in these areas (Supplementary Table S37).

During 1990–2021, the fastest increase in DALYs and deaths due to CKD was attributed to impaired kidney function, which has always been the leading cause of CKD. The subsequent risk factors, in descending order of impact, were high fasting plasma glucose levels, high BMI, and high systolic blood pressure. Other contributing risk factors showed relatively stable progression over the same period (Supplementary Tables S36, S38). Compared to the 1990 data, across all SDI regions, a noticeable decline was observed in the proportion of the ASDR and ASDAR due to impaired kidney function. Conversely, the influence of high fasting plasma glucose levels and high BMI increased. In more economically advanced areas, such as high-and high-middle-SDI regions, the proportion of risk attributed to high BMI remained above the global average. In contrast, in less economically developed low-SDI regions, the ASDR and ASDAR due to impaired kidney function and a diet low in fruits were notably higher than their global shares (Supplementary Tables S37, S39).

## 4 Discussion

Globally, CKD cases doubled during 1990–2021. The ASR of incidence, death, and DALYs for CKD has increased over the past three decades, nearly all countries saw ASIR increases, with approximately 70% also experiencing rising ASDARs, indicating that CKD remains a serious global health issue that requires focused attention. Demographically, older adults have the highest ASDARs, and male individuals have higher ASDAR than do female individuals, with the largest gap observed in CKD due to hypertension. Among all CKD cases, CKD due to T2DM has the highest ASDR and ASDAR and is growing at the fastest rate. Only CKD due to T1DM has shown a decrease in the ASDR and ASDAR since 1990. Among the four distinct CKD etiologies, those due to T1DM exhibited the most rapid increases in both the ASIR and ASPR. However, patients with CKD due to T2DM and hypertension experienced a decline in both the ASIR and ASPR. Moreover, the ASIR and ASPR for nephritis-related CKD showed a growing trend.

Risk factors for CKD include environmental influences, lifestyle choices, dietary habits, and metabolic conditions. Over the past 30 years, the ASDARs attributable to nearly all CKD risk factors have increased, indicating that efforts for the early diagnosis and prevention of CKD are insufficient. Among all the global risk factors in 2021, the DALYs caused by impaired kidney function were higher than those attributed to drug use, low physical activity, second-hand smoke, and diet (8). However, despite these risk factors, public health policymakers and the general population do not pay sufficient attention to kidney health. According to the latest research findings from the Global Atlas of Kidney Health, only 25% of the countries have developed national strategies for CKD, and 48% have identified CKD as a health priority (14). Additionally, the awareness rate of patients with early-stage CKD concerning their condition is extremely low (<5%), possibly because people mistakenly believe that CKD is merely a complication of diabetes or hypertension (15). Many patients seek medical help when they progress to ESKD. Once ESKD is achieved, life only can be sustained through renal replacement therapy or kidney transplantation. This transition imposes a heavy economic burden on patients and often triggers anxiety and fear of death due to uncertainty about prognosis. In response, many patients turn to spiritual beliefs, attempting to heal their illness through religion and attain inner peace. Studies have shown that for patients with ESKD, particularly those with lower socioeconomic status, seeking meaning in life through spirituality or religious connections can enhance psychological adaptation to severe illness (16). It is estimated that approximately 5.4 million patients will require renal replacement therapy by 2030 (17). Approximately 2–3.7 million people die prematurely due to the unavailability of this life-saving but expensive treatment (18). Therefore, effectively reducing the high incidence and mortality rates associated with CKD depends on early identification of CKD risk factors, timely diagnosis, and implementation of effective intervention measures to delay or prevent further deterioration of kidney function (19). In addition to conventional modern medical treatments, many countries—particularly China, South Korea, and Japan—are increasingly utilizing traditional herbal medicines or bioactive extracts in clinical practice to alleviate discomfort caused by toxin accumulation in patients with ESKD (20).

Metabolic risk factors, including impaired kidney function, high systolic blood pressure, high fasting plasma glucose levels, and high BMI, have consistently been the primary causes of CKD between 1990 and 2021. This is partly attributed to sociodemographic aging and lifestyle changes. Globally, CKD and kidney function impairment remain a heavy burden. When examining the correlation between the ASDR and ASDAR of CKD caused by kidney injury and SDI, a negative correlation was revealed between these metrics. Fortunately, the proportions of the ASDR and ASDAR attributable to kidney function impairment have decreased. This indicates the progress in healthcare services, preventive strategies, and disease management. High systolic blood pressure, the second-largest third-level contributor to the risk burden in 2021, along with high fasting plasma glucose and high BMI, revealed a trend of increasing health burdens due to major metabolic risk factors (8). Over the past 30 years, high systolic blood pressure has consistently been a primary risk factor for the onset and progression of CKD (21). The burden of CKD attributed to high systolic blood pressure varies by region, with middle-high SDI areas had minimal ASDAR growth and middle-low-SDI regions bore heaviest burden with rapid rise. This may be related to different dietary structures, preventive health policies, medical standards, and blood pressure management across regions. Elevated blood pressure is associated with kidney function progression, and reports have suggested that enhanced blood pressure control may lower the estimated glomerular filtration rate (eGFR) and increase the tubular biomarker levels (22). However, globally, only 23% of female individuals and 18% of male individuals have well-controlled blood pressure (23). Therefore, maintaining optimal blood pressure control is crucial for delaying kidney function decline and preventing complications in patients with CKD (24). A high fasting plasma glucose level is a recognized risk factor for CKD. It progresses gradually into diabetic nephropathy and leads to impaired kidney function (25). This was confirmed in a previous *in vitro* study, where high fasting plasma glucose levels impaired mitochondrial respiration in mesangial and tubular cells (26). Owing to population aging, rapid urbanization, and industrialization, unhealthy lifestyles are becoming more prevalent, including increased consumption of high-sugar and high-fat diets, reduced physical activity, and rising obesity rates, leading to a global increase in average fasting plasma glucose levels (27). Notably, high-middle-SDI regions have surpassed high-SDI areas to achieve the lowest ASDAR through effective control. This highlights the need to recognize that the association between high fasting plasma glucose-induced CKD burden and the SDI should not be oversimplified or linearized (28). The burden of CKD owing to high BMI significantly increased in nearly all regions worldwide, with the ASDR and ASDARs positively correlated with the SDI in 2021. High and middle-high-SDI regions have higher obesity rates, potentially making relevant interventions more valuable in these areas (29). Simultaneously, economic development in low-and middle-income countries has prompted significant lifestyle changes, characterized by increased sedentary behavior and the adoption of Westernized dietary habits. These changes may increase exposure to risk factors and exacerbate the global burden of CKD. Contributing factors include obesity, physical inactivity, and unhealthy diets—particularly those deficient in fruits and vegetables (30, 31). Notably, many fruits and vegetables contain natural antioxidants, which have been shown in multiple studies to reduce cellular oxidative damage, offering diverse health benefits relevant to disease prevention and treatment (32).

Geographically, significant differences are observed in the contribution of the CKD burden across regions and countries. The gap in the ASDAR between low-, middle-low-, and middle-SDI regions and high-and middle-high-SDI regions reflects inequalities in access to preventive care and renal replacement therapy due to varying levels of socio-economic development. Additionally, a sizeable amount of research data primarily originates from developed countries, which may be partly attributed to the limited access to comprehensive CKD testing in regions where medical resources and advanced laboratory diagnostic services are constrained, leading to incomplete diagnosis and data reporting of CKD in low-and middle-SDI regions (33). Therefore, CKD should receive greater attention in global health policy decisions, particularly in the low-and middle-SDI regions. Early screening and implementation of kidney-preserving treatments, including effective control of CKD risk factors (such as high systolic blood pressure and high fasting plasma glucose), can reduce the incidence of ESKD (34). Simultaneously, we should recognize that implementing screening and intervention measures in these countries may pose even greater challenges to their existing overburdened health resources.

Over the past 30 years, the ASIR of total CKD has consistently been higher in female individuals than in male individuals; however, the ASDAR has been lower in female individuals. However, among the four specific causes of CKD, the ASIR in male individuals exceeded that in female individuals, suggesting that, besides the known specific causes, female individuals are also affected by other unspecified factors, leading to a higher ASIR. Therefore, more screenings are required for female individuals. The simplest explanation is that female individuals generally live longer, and kidney function naturally declines with age, thus increasing the incidence of CKD (35). This sex difference highlights the role of sex hormones, such as the protective effect of premenopausal sex hormones. A longer reproductive period is associated with a lower risk of CKD, indicating that the cumulative protective effect of estrogen becomes apparent over time (36, 37). Male individuals have higher ASDR and ASDAR, indicating that they may suffer more long-term health losses due to CKD. CKD poses a higher fatal risk to male individuals, meaning that they progress to ESKD faster. This may be related to androgenic effects, NO metabolism, and excessive oxidative stress (38). Additionally, previous studies have shown that the lifetime risk of kidney replacement therapy is higher in male individuals than that in female individuals, while the usage rate of kidney replacement therapy is also higher in male individuals. This indirectly supports the observation that male individuals with CKD tend to progress more rapidly than female individuals (39). These findings suggest that female patients are more likely to opt for conservative treatment approaches, potentially due to slower progression of CKD or other factors influencing treatment choices. Overall, the observed sex disparity in CKD incidence fundamentally represents the interplay of multiple biological mechanisms—specifically sex hormone regulation and aging processes—with clinical phenotypes including disease progression rate and treatment requirements, rather than being attributable to a singular risk factor.

In addition to exhibiting sex dimorphism, the CKD burden is most prominent in older adults. Regardless of total CKD or the four specific causes of CKD, the ASIR and ASDARs in older adults were higher than those in other age groups. The ASDARs peaked in 2010 and have remained stable since then, possibly related to the World Health Organization’s implementation of the global strategy action plan for the prevention and control of non-communicable diseases (NCDs) around 2010 (40). Older adults have a higher prevalence of hypertension and diabetes, making them more susceptible to kidney damage. However, natural aging also leads to structural changes in the kidneys, resulting in decreased eGFR. CKD and aging influence each other; the prevalence of CKD is positively correlated with age, and CKD accelerates biological aging through multiple mechanisms (41). In a longitudinal study of healthy populations, the urinary creatinine clearance rate decreased at approximately 0.75 mL/min per year (42). Therefore, according to the single standard definition of eGFR <60 mL/min/1.73 m^2^, approximately half of the individuals aged >70 years may be diagnosed with CKD; however, it might just be physiological aging rather than a disease (43). With age, reduced muscle mass can lead to a decrease in serum creatinine levels, which may mask a true decline in kidney function (44). Thus, the eGFR standard for diagnosing CKD should be modified based on age to account for the physiological decline in eGFR due to healthy aging, thereby avoiding the overdiagnosis of CKD in older individuals (45).

NCDs have replaced infectious diseases as the most common causes of global morbidity and premature death (46–48). In response, the United Nations set a sustainable development goal to reduce premature mortality from NCDs by one-third by 2030. The impact of CKD on the NCD burden extends beyond ESKD or CKD itself; it often acts as a key risk factor for major NCDs (cardiovascular disease, hypertension, and diabetes), necessitating proactive prevention and treatment of this disease. The global ASDR for cardiovascular diseases, cancer, and chronic respiratory diseases decreased by 34.3, 21.4, and 36.7%, respectively, between 1990 and 2021. However, the ASDR for CKD has increased by 24.6% (49). Ample evidence supports the idea that the early diagnosis and timely treatment of CKD are key components of comprehensive national NCD strategies (50). Implementing cost-effective interventions for individuals aged ≥55 years, including those with a history of diabetes, hypertension, cardiovascular disease, and a family history of kidney disease, will reduce the risk of ESKD and cardiovascular diseases, thereby significantly alleviating the burden of CKD. Low-income, middle-income, and high-risk populations are primary beneficiaries.

However, this study has some limitations. First, the GBD relies on reporting data from different countries and regions, which may vary in quality and completeness. In areas where data collection is incomplete or inaccurate, GBD estimates may be biased. Moreover, GBD data typically exhibit a time lag, meaning that the latest data may not reflect current health conditions and may fail to capture the impact of sudden public health events on the disease burden. Second, different diagnostic criteria and methods may have been used across countries and periods, which can affect regional and temporal comparisons. Furthermore, changes in disease coding and classification systems, such as updates to ICD codes, can affect the consistency and comparability of data. Third, a GBD study employs complex statistical models to estimate disease burden, which are based on a series of assumptions that may affect the accuracy of the estimates. Fourth, GBD research primarily focuses on estimating the disease burden rather than the effectiveness of interventions, which may make it challenging to evaluate and accurately adjust public policy and health planning. These limitations highlight the importance of considering potential biases and constraints when developing public health policies and clinical practice guidelines, ensuring that the measures implemented effectively address both current and future health challenges.

## Data Availability

Publicly available datasets were analyzed in this study. This data can be found here: https://ghdx.healthdata.org/gbd-2021, GBD 2021. The original contributions presented in the study are included in the article/Supplementary material, further inquiries can be directed to the corresponding author.

## Footnotes

^1^ http://ghdx.healthdata.org/gbd-results-tool
