# Correction: Global burden of chronic kidney disease and risk factors, 1990–2021: an update from the global burden of disease study 2021

**DOI:** 10.3389/fpubh.2025.1672557

**Published:** 2025-08-06

**Authors:** Lanhui Wang, Yining He, Chao Han, Peiqi Zhu, Yaping Zhou, Ruijie Tang, Weiming He

**Affiliations:** ^1^Division of Nephrology, Affiliated Hospital of Nanjing University of Chinese Medicine, Jiangsu Province Hospital of Chinese Medicine, Nanjing, China; ^2^Yancheng Dafeng Hospital of Chinese Medicine, Teaching Hospital of Nanjing University of Chinese Medicine, Yancheng, China

**Keywords:** chronic kidney disease, diabetes mellitus, epidemiology, global burden of disease study, glomerulonephritis, hypertension, risk factors

Author Yining He was erroneously omitted as equal contributing author. “These authors contributed equally to this work” has been added to authors Lanhui Wang and Yining He.

The original version of this article has been updated.

