# Supplementary material for: Global burden of chronic kidney disease and risk factors, 1990–2021: an update from the global burden of disease study 2021
Source: Front Public Health. 2025 Jul 24;13:1542329. doi: 10.3389/fpubh.2025.1542329 (PMC12366504; doi:10.3389/fpubh.2025.1542329)
Supplement: Supplementary file 1 [file Table_1.docx]

| **TableS1: National Burden of chronic kidney disease: prevalence cases, ASPR, and EAPC (1990–2021).** | | | | | |
| --- | --- | --- | --- | --- | --- |
| Country | **1990** | | **2021** | | **EAPC_95%CI** |
|  | Number_95%UI | ASR | Number_95%UI | ASR |  |
| Afghanistan | 611982 (569081-656341) | 8808.89 (8175.35-9452.34) | 1440765 (1317910-1571513) | 8934.9 (8262.77-9627.27) | 0.05 (0.03-0.06) |
| Albania | 152351 (142462-163786) | 6243.74 (5830.9-6667.44) | 230609 (214471-246180) | 6153.95 (5711.78-6544.94) | -0.05 (-0.06--0.04) |
| Algeria | 1413887 (1302734-1527151) | 8945.97 (8267.19-9615.92) | 3553584 (3275804-3852065) | 8807.19 (8150.01-9515.2) | -0.11 (-0.14--0.08) |
| American Samoa | 2729 (2519-2958) | 8831.31 (8197.44-9501.62) | 4468 (4153-4802) | 9256.65 (8599.04-9943.02) | 0.17 (0.16-0.19) |
| Andorra | 3305 (3059-3549) | 5604.92 (5216.11-5996.52) | 7623 (7073-8129) | 5534.99 (5147.91-5919.35) | -0.03 (-0.04--0.02) |
| Angola | 473841 (440001-510739) | 9090.82 (8510.84-9718.36) | 1482282 (1374318-1605870) | 9018.88 (8383.16-9636.04) | -0.03 (-0.04--0.03) |
| Antigua and Barbuda | 3729 (3474-3994) | 6700.12 (6253.5-7179.1) | 7270 (6762-7815) | 6956.33 (6479.95-7455.65) | 0.15 (0.14-0.15) |
| Argentina | 1774583 (1648138-1900776) | 5585.84 (5197.53-5980) | 3114327 (2893145-3345866) | 5876.08 (5442.4-6323.9) | 0.24 (0.21-0.28) |
| Armenia | 300635 (278865-323273) | 10265.94 (9580.75-10973.98) | 404070 (378068-430935) | 10245.02 (9598.01-10928.04) | -0.03 (-0.05--0.01) |
| Australia | 1165447 (1094777-1238029) | 6076.77 (5714.75-6452.52) | 2353593 (2211156-2523412) | 5877.91 (5525.88-6272.06) | -0.07 (-0.09--0.04) |
| Austria | 611202 (567555-653621) | 5590.87 (5205.69-5978.51) | 892225 (838208-953902) | 5671.05 (5326.44-6084.53) | 0.08 (0.07-0.09) |
| Azerbaijan | 593465 (554684-636804) | 10594.84 (9927.52-11288.36) | 1146469 (1073053-1221894) | 10695.96 (10018.97-11338.19) | 0.03 (0.02-0.04) |
| Bahamas | 12715 (11806-13734) | 6542.75 (6098.88-7002.89) | 28177 (26096-30221) | 6759.28 (6258.56-7239.09) | 0.13 (0.13-0.14) |
| Bahrain | 31525 (28728-34958) | 9152.15 (8487.52-9866.93) | 130181 (119335-142831) | 9108.49 (8445.16-9765.84) | -0.02 (-0.03--0.01) |
| Bangladesh | 5331909 (4950686-5772489) | 8395.62 (7832.81-8968.06) | 12882108 (11949483-13899210) | 8555.39 (7963.38-9177.27) | -0.02 (-0.06-0.02) |
| Barbados | 17997 (16818-19169) | 6459.85 (6051.67-6909.15) | 28988 (27004-31010) | 6676.76 (6215.33-7161.73) | 0.13 (0.12-0.14) |
| Belarus | 1162192 (1079543-1248012) | 9654.09 (8993.23-10364.91) | 1314740 (1220742-1405904) | 9704.35 (9000.43-10430.51) | -0.01 (-0.02-0) |
| Belgium | 808609 (750019-867640) | 5722.14 (5342.51-6109.53) | 1120459 (1051828-1196504) | 5633.19 (5305.47-6009.37) | 0.01 (-0.01-0.02) |
| Belize | 7609 (7071-8133) | 6629.92 (6188.8-7130.86) | 24923 (23248-26780) | 6996.94 (6519.92-7476.11) | 0.2 (0.19-0.2) |
| Benin | 188015 (174766-201634) | 7343.41 (6857.48-7853.45) | 552484 (512421-599062) | 7482.3 (7003.24-8021.89) | 0.07 (0.05-0.09) |
| Bermuda | 4041 (3755-4358) | 6209.73 (5792.54-6656.89) | 6571 (6087-7048) | 6114.22 (5679.31-6583.54) | -0.04 (-0.06--0.03) |
| Bhutan | 31715 (29384-34240) | 9135.27 (8522.13-9749.86) | 64186 (59913-69314) | 9213.54 (8612.13-9876.63) | 0.03 (0.02-0.04) |
| Bolivia (Plurinational State of) | 242900 (225810-260389) | 6093.33 (5681.07-6496.89) | 628320 (584306-676494) | 6177.14 (5740.24-6632.37) | 0.06 (0.04-0.09) |
| Bosnia and Herzegovina | 260939 (242972-279575) | 6132.37 (5735.3-6544.34) | 323305 (300335-346499) | 6233.46 (5827.17-6665.08) | 0.06 (0.05-0.06) |
| Botswana | 60835 (56449-65830) | 8684.67 (8075.88-9285.61) | 164894 (153184-177219) | 8867.29 (8244.14-9441.16) | 0.1 (0.09-0.11) |
| Brazil | 8215420 (7640129-8812335) | 7772.73 (7248.74-8291.21) | 18814692 (17566365-20174535) | 7576.55 (7078.47-8111.19) | -0.08 (-0.09--0.06) |
| Brunei Darussalam | 15220 (13998-16528) | 9497.75 (8852.21-10130.49) | 39451 (36520-42780) | 9461.95 (8796.56-10168.95) | -0.03 (-0.04--0.03) |
| Bulgaria | 673785 (626288-720194) | 6187.85 (5772.96-6611.13) | 726001 (674875-778060) | 6282.06 (5866.87-6704.4) | 0.05 (0.05-0.06) |
| Burkina Faso | 365246 (337985-392297) | 7076.19 (6568.58-7570.49) | 901049 (834507-973982) | 7181.79 (6699.93-7686.7) | 0.05 (0.05-0.06) |
| Burundi | 187278 (170970-204259) | 5868.15 (5412.1-6352.77) | 456223 (414259-502680) | 5921.59 (5472.49-6436.62) | 0.04 (0.04-0.05) |
| C?te d'Ivoire | 17443 (16294-18677) | 7283.33 (6802.8-7767.94) | 38020 (35307-41075) | 7366.66 (6831.08-7880.32) | 0.05 (0.04-0.07) |
| Cabo Verde | 566875 (518696-615039) | 9455.04 (8749.38-10222.83) | 1378217 (1266270-1503301) | 9452.98 (8751.36-10231.34) | 0 (-0.01-0.01) |
| Cambodia | 463067 (431742-496225) | 8270.67 (7742.07-8827.31) | 1594660 (1486358-1713045) | 9043.6 (8503.39-9609.76) | 0.33 (0.22-0.45) |
| Cameroon | 2229477 (2055472-2415616) | 7026.44 (6480.64-7603.72) | 3809329 (3540568-4060952) | 6524.37 (6091.51-7000.69) | -0.1 (-0.16--0.05) |
| Canada | 133406 (123251-143340) | 9207.41 (8552.54-9860.33) | 276101 (255353-297770) | 9141.25 (8513.25-9769.2) | -0.02 (-0.02--0.01) |
| Central African Republic | 241571 (223146-258353) | 7195.07 (6688.44-7683.2) | 603512 (558192-650782) | 7271.05 (6764.68-7787.57) | 0.04 (0.03-0.04) |
| Chad | 699136 (650457-755320) | 6210.96 (5786.85-6653.92) | 1444161 (1343232-1545985) | 6106.55 (5659.58-6545.88) | -0.03 (-0.04--0.01) |
| Chile | 67398525 (62608781-72614585) | 7091.9 (6614.31-7603.09) | 118403911 (109388671-127484286) | 6249.41 (5812.36-6720.21) | -0.19 (-0.33--0.05) |
| China | 1697055 (1583361-1826064) | 7912.44 (7376.24-8461.75) | 4222423 (3939788-4478779) | 7695.61 (7194.03-8158.77) | -0.05 (-0.07--0.03) |
| Colombia | 15628 (14361-16936) | 5870.66 (5455.67-6305.4) | 35339 (32389-38509) | 5851.82 (5407.43-6323.6) | -0.01 (-0.01-0) |
| Comoros | 122987 (114132-132085) | 9370.06 (8763.1-9997.99) | 331895 (307907-357776) | 9319.63 (8696.92-9931.83) | -0.02 (-0.03--0.02) |
| Congo | 1150 (1062-1242) | 8019.63 (7447.92-8644.54) | 1841 (1712-1970) | 8230.01 (7649.94-8830.44) | 0.09 (0.08-0.1) |
| Cook Islands | 197345 (183956-211905) | 9636.74 (8996.25-10298.8) | 517788 (484174-550601) | 9532.08 (8909.84-10127.1) | 0.06 (0.04-0.08) |
| Costa Rica | 352794 (326765-378976) | 6188.88 (5755.54-6628.99) | 441010 (407740-471831) | 6109.13 (5670.01-6576.33) | -0.05 (-0.05--0.04) |
| Croatia | 670575 (622916-717810) | 6258.54 (5824.65-6706.15) | 1023168 (949443-1092062) | 6157.34 (5728.33-6614.34) | -0.06 (-0.07--0.04) |
| Cuba | 47988 (44580-51332) | 6008.97 (5608.31-6395.05) | 112031 (104079-120582) | 5817.59 (5430.82-6247.88) | -0.11 (-0.12--0.1) |
| Cyprus | 777458 (723745-829773) | 6159.6 (5756.88-6580.72) | 1033665 (960274-1099534) | 5875.02 (5479.31-6268.38) | -0.13 (-0.15--0.11) |
| Czechia | 462576 (427133-500797) | 7562 (7049.37-8106.42) | 1249475 (1157292-1360894) | 7597.71 (7075.19-8139.17) | 0.02 (0.01-0.02) |
| Democratic People's Republic of Korea | 1150078 (1067243-1233674) | 6571.77 (6117.41-7043.46) | 2027239 (1883443-2173423) | 6390.78 (5955.9-6816.79) | -0.15 (-0.18--0.13) |
| Democratic Republic of the Congo | 1824071 (1691866-1955647) | 9255.33 (8613.46-9831.06) | 4529099 (4238150-4861233) | 9196.85 (8707.06-9726.56) | -0.1 (-0.12--0.08) |
| Denmark | 415234 (386838-442513) | 5589.55 (5229.32-5953.9) | 564571 (524056-607172) | 5607.02 (5237.41-6009.77) | 0 (-0.02-0.01) |
| Djibouti | 13012 (11876-14272) | 5545.12 (5142.12-5961.77) | 55546 (50733-60942) | 5776.89 (5342.94-6232.52) | 0.16 (0.15-0.16) |
| Dominica | 4263 (3961-4572) | 6991.41 (6509.2-7479.94) | 5745 (5329-6134) | 7303.91 (6813.01-7818.62) | 0.16 (0.16-0.17) |
| Dominican Republic | 299732 (277339-324408) | 6357.9 (5927.63-6822.57) | 710332 (660993-766986) | 6688.81 (6211.08-7193.25) | 0.21 (0.19-0.22) |
| Ecuador | 392453 (366304-421830) | 5893.37 (5491.99-6356.84) | 1073132 (996177-1149397) | 6249.3 (5811.7-6689.18) | 0.25 (0.23-0.28) |
| Egypt | 3069526 (2834187-3319651) | 8613.59 (8031.71-9206.03) | 7320032 (6762507-7887829) | 9167.78 (8515.01-9825.36) | 0.27 (0.24-0.29) |
| El Salvador | 239034 (222018-257099) | 6892.21 (6378.22-7415.71) | 460009 (431238-489587) | 7296.14 (6841.63-7770.96) | 0.34 (0.29-0.39) |
| Equatorial Guinea | 20935 (19447-22604) | 9087.34 (8498.49-9734.45) | 75546 (69523-82103) | 9342.3 (8719.87-9945.44) | 0.11 (0.09-0.12) |
| Eritrea | 99470 (90769-109284) | 5529.86 (5117.87-5982.06) | 244600 (224167-268056) | 5676.57 (5255.07-6117.57) | 0.11 (0.1-0.11) |
| Estonia | 181645 (168130-196037) | 9678.72 (8968.05-10435.79) | 206581 (192387-221597) | 9660.85 (9006.34-10389.66) | 0.01 (0-0.02) |
| Eswatini | 33811 (31458-36377) | 8783.16 (8183.64-9417.55) | 66871 (61736-72023) | 9047.6 (8414.14-9624.29) | 0.13 (0.12-0.14) |
| Ethiopia | 1542523 (1422919-1673239) | 5569.28 (5168.79-5985.12) | 3762262 (3443544-4113603) | 5649.28 (5230.25-6098.21) | 0.1 (0.08-0.12) |
| Fiji | 43084 (39626-46792) | 8584.29 (7980.69-9231.03) | 74318 (68583-80409) | 8913.76 (8286.56-9600.93) | 0.16 (0.14-0.17) |
| Finland | 368157 (345612-392962) | 5556.65 (5209.26-5940.57) | 555910 (513129-595333) | 5349.21 (4968.62-5714.85) | -0.11 (-0.13--0.09) |
| France | 3209806 (2980224-3426824) | 4202.18 (3898.73-4488.25) | 5214795 (4858423-5614715) | 4368.82 (4085.59-4698.68) | 0.12 (0.03-0.21) |
| Gabon | 57987 (53943-62001) | 9126.03 (8487.06-9779.76) | 117028 (108707-125772) | 9237.99 (8645.4-9881.02) | 0.05 (0.04-0.05) |
| Gambia | 36537 (33712-39478) | 7285.83 (6764.38-7776.61) | 103566 (95771-112154) | 7469.17 (6954.59-8002.94) | 0.1 (0.08-0.12) |
| Georgia | 632971 (590879-679007) | 10617.53 (9919.39-11342.7) | 544204 (506648-579897) | 10551.11 (9851.3-11224) | -0.03 (-0.05-0) |
| Germany | 6261066 (5866849-6644060) | 5349.62 (5017.36-5674.99) | 8931944 (8467049-9441979) | 5278.41 (4985.46-5622.16) | -0.05 (-0.11-0.01) |
| Ghana | 516871 (472923-562875) | 5943.09 (5509.33-6423.82) | 1422793 (1305227-1553890) | 6033.8 (5584.86-6513.19) | 0.06 (0.05-0.06) |
| Greece | 798820 (740666-853941) | 5762.34 (5361.41-6162.89) | 1143247 (1066167-1220356) | 5587.78 (5236.26-5992.78) | -0.11 (-0.12--0.09) |
| Greenland | 2958 (2729-3212) | 6970.68 (6515.6-7414.55) | 4412 (4094-4732) | 6988.24 (6509.14-7491.4) | 0.01 (0.01-0.01) |
| Grenada | 5042 (4684-5387) | 6983.13 (6481.81-7464.02) | 8182 (7614-8784) | 7279.46 (6800.35-7818.68) | 0.17 (0.16-0.17) |
| Guam | 8188 (7532-8928) | 7935.12 (7378.36-8544.49) | 16160 (14994-17291) | 8480.2 (7890.47-9105.98) | 0.26 (0.24-0.27) |
| Guatemala | 342544 (318619-365583) | 8065.47 (7574.08-8638.44) | 1096863 (1023152-1176256) | 8857.74 (8275.1-9478.35) | 0.38 (0.35-0.41) |
| Guinea | 275726 (256995-296333) | 7313.88 (6820.17-7803.84) | 568301 (528813-612479) | 7498.27 (6998.79-7995.51) | 0.1 (0.1-0.11) |
| Guinea-Bissau | 40061 (37305-43253) | 7641.76 (7095.74-8167.34) | 85214 (78709-92411) | 7643.78 (7104.09-8145.37) | 0 (0-0.01) |
| Guyana | 34192 (31637-36857) | 6803.89 (6325.87-7280.49) | 48899 (45521-52822) | 7062.48 (6578.99-7608.24) | 0.15 (0.14-0.15) |
| Haiti | 274957 (254724-296317) | 6959.23 (6473.01-7441.57) | 662956 (613153-717829) | 7123.74 (6614.82-7647.93) | 0.1 (0.09-0.1) |
| Honduras | 209413 (194859-225041) | 8405.32 (7844.1-8968.83) | 633449 (591340-678996) | 8612.98 (8025.92-9216.03) | 0.1 (0.09-0.11) |
| Hungary | 816867 (760112-874417) | 6193.16 (5788.18-6630.36) | 965275 (898080-1028450) | 5992.86 (5610.73-6393.59) | -0.11 (-0.12--0.1) |
| Iceland | 13378 (12516-14287) | 4739.04 (4420.17-5063.17) | 23128 (21625-24673) | 4528.93 (4231.01-4840.19) | -0.14 (-0.17--0.1) |
| India | 60111283 (55460535-65038616) | 10258.75 (9547.88-10986.05) | 128031911 (118513994-138721278) | 9710.51 (9024.24-10420.26) | -0.25 (-0.31--0.19) |
| Indonesia | 13599739 (12496422-14810294) | 10447.73 (9661.88-11264.48) | 29413896 (26931317-32010165) | 10740.3 (9942.08-11598.81) | 0.09 (0.08-0.1) |
| Iran (Islamic Republic of) | 3141831 (2920460-3371296) | 9513.67 (8874.05-10180.4) | 8145077 (7562399-8737333) | 9474.79 (8837.14-10118.39) | 0 (-0.02-0.01) |
| Iraq | 1026557 (949992-1108210) | 9536.93 (8851.49-10239.34) | 2977727 (2740400-3234895) | 9464.18 (8764.44-10188.22) | -0.04 (-0.05--0.02) |
| Ireland | 261985 (241344-282388) | 6681.69 (6202.54-7184.27) | 465353 (440528-488132) | 6594.14 (6237.54-6952.01) | -0.03 (-0.06-0.01) |
| Israel | 288923 (270285-308809) | 6007.76 (5618.93-6420.26) | 674873 (630913-722204) | 5866.76 (5487.92-6273.74) | -0.08 (-0.09--0.08) |
| Italy | 4457622 (4142484-4769497) | 5591.86 (5235.46-5978.78) | 5921997 (5501809-6294811) | 5061.28 (4746.22-5410.53) | -0.27 (-0.31--0.23) |
| Jamaica | 122884 (115047-131333) | 6456.88 (6045.33-6921.34) | 209554 (194578-224704) | 6708.14 (6219-7188.82) | 0.17 (0.14-0.2) |
| Japan | 13889911 (12903717-14885294) | 8744.79 (8150.41-9364.15) | 22877631 (21341971-24294034) | 8264.3 (7717.71-8855.73) | -0.16 (-0.22--0.1) |
| Jordan | 187654 (172497-203471) | 9249.53 (8606.77-9945.62) | 889238 (817131-968041) | 8989.67 (8318.59-9729.3) | -0.12 (-0.15--0.1) |
| Kazakhstan | 1471284 (1368347-1573180) | 10730.43 (10056.28-11411.96) | 1952569 (1812225-2090896) | 10662.37 (9928.1-11394.35) | -0.02 (-0.03--0.01) |
| Kenya | 700742 (642264-764896) | 5761.73 (5343.22-6221.9) | 1979251 (1807371-2167364) | 5900.75 (5470-6368.96) | 0.09 (0.08-0.09) |
| Kiribati | 4029 (3716-4354) | 8459.58 (7856.76-9093.47) | 7836 (7239-8460) | 8718.24 (8109.87-9329.42) | 0.13 (0.12-0.14) |
| Kuwait | 106214 (96298-117759) | 8982.78 (8321.07-9651.59) | 393166 (356902-436552) | 8593.08 (7931.84-9269.39) | -0.16 (-0.16--0.15) |
| Kyrgyzstan | 349912 (326466-372023) | 10650.21 (9986.27-11306.14) | 554675 (518240-592588) | 10147.73 (9422.6-10780.31) | -0.19 (-0.21--0.16) |
| Lao People's Democratic Republic | 277122 (254762-299243) | 10702 (9904.76-11504.62) | 621716 (570484-678042) | 10478.69 (9688.02-11292.97) | -0.06 (-0.07--0.05) |
| Latvia | 313358 (290679-336853) | 9660.91 (8967.62-10391.33) | 303626 (282328-323377) | 9785.76 (9110.46-10514.29) | 0.05 (0.04-0.06) |
| Lebanon | 211783 (195737-228352) | 8827.59 (8183.84-9494.6) | 548008 (508010-589947) | 8988.21 (8309.11-9607.83) | 0.09 (0.05-0.12) |
| Lesotho | 79823 (74328-85461) | 8435.22 (7898.67-8992.7) | 114258 (105784-123018) | 8824.57 (8201.21-9408.83) | 0.19 (0.18-0.2) |
| Liberia | 107783 (99699-116096) | 7548.18 (7002.1-8076.21) | 244755 (226016-265719) | 7551.19 (7049.82-8085.22) | 0.02 (0.02-0.03) |
| Libya | 228746 (211473-246316) | 9024.45 (8359.99-9727.35) | 610601 (560616-667943) | 9269.11 (8567.5-10032.22) | 0.11 (0.09-0.13) |
| Lithuania | 414295 (384463-446122) | 9790.23 (9086.72-10563.46) | 441211 (411206-472545) | 9837.2 (9134.92-10590.03) | 0.03 (0.02-0.04) |
| Luxembourg | 30243 (27852-32537) | 5937.62 (5491.41-6400.12) | 55107 (50921-59381) | 5775.64 (5340.05-6220.77) | -0.05 (-0.08--0.03) |
| Madagascar | 389060 (359027-423079) | 5625.82 (5196.63-6061.5) | 994473 (908917-1089154) | 5685.27 (5246.18-6164.05) | 0.05 (0.04-0.06) |
| Malawi | 326018 (298440-355273) | 5918.53 (5492.05-6409.4) | 705439 (644778-770229) | 6124.79 (5658.19-6619.29) | 0.13 (0.11-0.14) |
| Malaysia | 1328704 (1222289-1442379) | 10828.26 (10076.24-11666.58) | 3443338 (3171290-3729769) | 11008.65 (10171.17-11849.41) | 0.19 (0.14-0.25) |
| Maldives | 12689 (11699-13709) | 10424.93 (9701.37-11220.84) | 52048 (47116-57976) | 10202.65 (9431.96-11087.04) | -0.07 (-0.08--0.06) |
| Mali | 358053 (333871-381898) | 7349.84 (6875.48-7816.73) | 909702 (841914-979718) | 7419.92 (6929.48-7932.56) | 0.03 (0.02-0.03) |
| Malta | 24308 (22689-26237) | 5881.7 (5499.49-6324.87) | 46498 (43037-49500) | 5665.77 (5292.24-6053.13) | -0.13 (-0.14--0.11) |
| Marshall Islands | 1905 (1760-2065) | 8175.25 (7594.65-8766.64) | 3728 (3432-4030) | 8476 (7879.65-9041.98) | 0.14 (0.13-0.15) |
| Mauritania | 91156 (84420-98252) | 7479.43 (6957.67-8019.85) | 197970 (183860-213616) | 7285.48 (6794.61-7783.79) | -0.1 (-0.11--0.09) |
| Mauritius | 96542 (88907-105229) | 10712.2 (9939.06-11620.82) | 189008 (175935-203155) | 11411.55 (10649.12-12263.72) | 0.24 (0.21-0.28) |
| Mexico | 4594717 (4287702-4924515) | 8781.48 (8206.61-9393.83) | 11780174 (11009027-12569139) | 9052.16 (8453.85-9672.71) | 0.15 (0.13-0.17) |
| Micronesia (Federated States of) | 5110 (4740-5505) | 8313.35 (7738.62-8898.94) | 7394 (6849-8003) | 8744.07 (8145.41-9371.47) | 0.19 (0.17-0.21) |
| Monaco | 3295 (3048-3530) | 5539.92 (5150.24-5916.29) | 4350 (4042-4640) | 5497.16 (5141.16-5862.88) | -0.02 (-0.02--0.01) |
| Mongolia | 146040 (136801-156132) | 11239.6 (10569.26-11958.83) | 287978 (267255-309250) | 10667.74 (9937.15-11381.17) | -0.19 (-0.21--0.18) |
| Montenegro | 40300 (37650-42973) | 6416.04 (6010.81-6835.49) | 54152 (50266-57833) | 6414.96 (5962.07-6863.77) | -0.01 (-0.01-0) |
| Morocco | 1521333 (1399644-1649317) | 8496.57 (7863.97-9155.09) | 3125307 (2880319-3372664) | 8498.36 (7864.02-9135.02) | -0.03 (-0.05--0.01) |
| Mozambique | 471644 (433799-513878) | 5918.7 (5479.55-6395.09) | 1066862 (978804-1170332) | 6162.57 (5705.7-6708.87) | 0.14 (0.13-0.15) |
| Myanmar | 2982783 (2758045-3235228) | 10357.18 (9596.64-11185.41) | 5373282 (4929525-5841826) | 10172.97 (9393.11-11017.34) | -0.02 (-0.03-0) |
| Namibia | 68175 (63117-73308) | 8664.38 (8056.49-9233.53) | 144692 (133912-155904) | 8510.12 (7908.45-9112.39) | -0.04 (-0.05--0.03) |
| Nauru | 544 (502-592) | 8632.12 (8048.05-9264.68) | 698 (644-757) | 9104.97 (8473.18-9748.89) | 0.19 (0.17-0.21) |
| Nepal | 1177468 (1101596-1257964) | 10501.16 (9843.11-11204.3) | 2762106 (2575557-3032129) | 10887.67 (10177.44-11883.1) | -0.02 (-0.06-0.03) |
| Netherlands | 1147619 (1073580-1226033) | 6067.39 (5675.85-6498.24) | 1745668 (1634215-1877107) | 6048.17 (5674.28-6481.09) | -0.13 (-0.18--0.08) |
| New Zealand | 234292 (217327-250752) | 6128.41 (5708.2-6546.64) | 457184 (423466-488353) | 6072.56 (5649.2-6499.05) | -0.04 (-0.05--0.03) |
| Nicaragua | 190029 (177519-203548) | 9675.18 (9075.43-10313.24) | 575347 (537918-614144) | 10511.16 (9794.29-11157.83) | 0.24 (0.23-0.26) |
| Niger | 265038 (246140-284905) | 6880.22 (6411.46-7331.36) | 790430 (731816-852136) | 6881.82 (6387.81-7357.14) | 0 (-0.01-0.02) |
| Nigeria | 4951044 (4609650-5305923) | 9402.32 (8798.45-10034.7) | 11730967 (10926433-12598672) | 9488.9 (8881.14-10113.28) | 0.12 (0.08-0.15) |
| Niue | 186 (173-199) | 8670.53 (8041.87-9333.97) | 175 (162-188) | 8957.48 (8282.57-9662.99) | 0.13 (0.11-0.14) |
| North Macedonia | 140331 (130960-149494) | 7386.01 (6902.04-7876.8) | 220103 (205048-234493) | 7377.4 (6929.87-7832.19) | -0.02 (-0.03--0.01) |
| Northern Mariana Islands | 2867 (2626-3141) | 8797.6 (8200.85-9441.36) | 4746 (4387-5099) | 9078.76 (8455.65-9714.06) | 0.11 (0.1-0.13) |
| Norway | 314586 (291897-335963) | 5207.12 (4866.27-5562.04) | 469377 (437256-500382) | 5534.4 (5167.84-5922.49) | 0.25 (0.22-0.28) |
| Oman | 98358 (89680-108123) | 8427.69 (7838.18-9068.71) | 344284 (313617-379720) | 9024.46 (8385.68-9702.41) | 0.29 (0.26-0.31) |
| Pakistan | 5931806 (5491196-6396491) | 8589.09 (7994.62-9202.42) | 15063043 (13945283-16289223) | 9164.6 (8522.84-9774.19) | 0.2 (0.17-0.23) |
| Palau | 1027 (956-1114) | 8544.17 (7955.58-9159.8) | 1947 (1797-2108) | 8900.4 (8266.84-9595.25) | 0.16 (0.15-0.18) |
| Palestine | 105072 (97202-113180) | 9224.59 (8569.81-9850.23) | 316240 (292025-342754) | 8942.6 (8314.54-9603.52) | -0.09 (-0.13--0.06) |
| Panama | 142393 (132809-153036) | 8261.64 (7709.25-8864.77) | 373196 (347346-398963) | 8453.52 (7857.18-9031.74) | 0.1 (0.09-0.11) |
| Papua New Guinea | 179603 (165528-195405) | 7388.6 (6896.15-7935.26) | 538462 (494564-590048) | 7685.65 (7155-8282.92) | 0.16 (0.13-0.19) |
| Paraguay | 192742 (179980-206867) | 7374.35 (6876.73-7891.93) | 481010 (448476-516329) | 7561.39 (7051.56-8091.32) | 0.09 (0.08-0.1) |
| Peru | 830105 (773577-894482) | 5621.62 (5237.55-6016.02) | 2042137 (1897519-2195297) | 5732.73 (5325.1-6154.29) | 0.1 (0.07-0.12) |
| Philippines | 4286723 (3941958-4660864) | 10549.81 (9776.74-11356.13) | 10193697 (9395294-11071571) | 10530.35 (9775.3-11372.86) | -0.02 (-0.04-0) |
| Poland | 2866015 (2680925-3059816) | 6850.93 (6418.54-7310.93) | 3872502 (3620089-4134821) | 6415.29 (6012.65-6855.34) | -0.29 (-0.31--0.27) |
| Portugal | 641583 (596339-685707) | 5124.52 (4804.74-5501.1) | 941606 (850524-1011350) | 4777.02 (4420.43-5132.33) | -0.23 (-0.27--0.19) |
| Puerto Rico | 241664 (224964-259466) | 6681.74 (6222.45-7166.22) | 380144 (353031-405700) | 6833.02 (6356.42-7333.3) | 0.09 (0.08-0.09) |
| Qatar | 28926 (26054-32385) | 9257.25 (8540.34-9961.49) | 238389 (214245-267350) | 9111.21 (8447.96-9852.64) | -0.1 (-0.13--0.08) |
| Republic of Korea | 2703535 (2505415-2935211) | 7789.36 (7279.97-8381.08) | 5620395 (5254764-6008210) | 6872.19 (6416.99-7364.36) | -0.57 (-0.63--0.51) |
| Republic of Moldova | 469578 (435381-501649) | 10729.28 (10014.3-11456.67) | 596118 (553806-636138) | 11293.66 (10519.57-12048.12) | 0.16 (0.12-0.21) |
| Romania | 1605525 (1495525-1715394) | 6180.42 (5775.44-6591.06) | 1993675 (1890808-2098153) | 6300.66 (5964.64-6652.48) | -0.17 (-0.24--0.1) |
| Russian Federation | 15387961 (14274967-16655381) | 9062.21 (8429.24-9771.39) | 18056081 (16744178-19455656) | 8973.81 (8345.48-9704.77) | -0.03 (-0.04--0.02) |
| Rwanda | 234499 (214398-255853) | 5852.58 (5422.12-6320) | 513932 (471486-561218) | 5696.48 (5277.18-6164.15) | -0.08 (-0.1--0.07) |
| Saint Kitts and Nevis | 2582 (2409-2774) | 7034.18 (6570.19-7551.83) | 4826 (4470-5192) | 6962.38 (6470.03-7455.74) | -0.04 (-0.06--0.03) |
| Saint Lucia | 6820 (6312-7305) | 6901.64 (6410.08-7371.53) | 15679 (14634-16864) | 6954.06 (6489.84-7468.21) | 0.07 (0.05-0.08) |
| Saint Vincent and the Grenadines | 5218 (4816-5601) | 6544.36 (6043.47-7001.28) | 9229 (8590-9910) | 6902.6 (6435.84-7413.47) | 0.19 (0.17-0.2) |
| Samoa | 8628 (8012-9299) | 8335.31 (7759.99-8940.84) | 13962 (12935-15079) | 8626.94 (8017.8-9290.73) | 0.13 (0.11-0.15) |
| San Marino | 1771 (1651-1899) | 5453.49 (5097.14-5832.29) | 3397 (3165-3648) | 5392.57 (5034.83-5768.75) | -0.04 (-0.05--0.03) |
| Sao Tome and Principe | 5810 (5424-6228) | 7967.41 (7434.12-8520.1) | 12272 (11408-13236) | 8194.64 (7639.23-8749.18) | 0.11 (0.1-0.12) |
| Saudi Arabia | 854299 (786274-926817) | 9213.18 (8560.75-9892.33) | 3067523 (2798321-3367524) | 9245.46 (8568.21-9935.62) | -0.03 (-0.05--0.01) |
| Senegal | 294300 (271578-318380) | 6986.31 (6472.32-7529.83) | 679249 (627366-734863) | 6614.01 (6185.73-7067.77) | -0.2 (-0.21--0.18) |
| Serbia | 603591 (559791-648669) | 5667.21 (5283.66-6068.98) | 753941 (699986-811166) | 5593.29 (5189.17-6041.03) | -0.06 (-0.08--0.05) |
| Seychelles | 6521 (6032-7044) | 10613.02 (9840.16-11453.24) | 12837 (11856-13936) | 10884.54 (10070.48-11752.96) | 0.09 (0.09-0.1) |
| Sierra Leone | 191300 (177895-206334) | 7521.27 (7006.42-8070.43) | 405349 (372950-439223) | 7610.57 (7071.49-8155.26) | 0.05 (0.04-0.06) |
| Singapore | 269496 (247876-292201) | 9889.29 (9177.06-10490.16) | 738749 (680520-792459) | 9229.95 (8547.75-9877.06) | -0.25 (-0.3--0.2) |
| Slovakia | 357483 (334696-382908) | 6221.57 (5843.73-6644.97) | 486371 (454761-518472) | 5960.02 (5575.94-6352.67) | -0.14 (-0.15--0.13) |
| Slovenia | 142299 (133101-152114) | 6083.57 (5696.48-6507.42) | 213545 (199557-227477) | 5968.15 (5539.43-6369.43) | -0.1 (-0.12--0.09) |
| Solomon Islands | 14747 (13720-15956) | 8066.65 (7526-8676.1) | 37933 (34962-41296) | 8231.57 (7603.53-8851.13) | 0.08 (0.07-0.09) |
| Somalia | 224333 (205558-246035) | 5516.9 (5100.65-5954.84) | 603690 (549230-663805) | 5564.97 (5152.59-6029.57) | 0.05 (0.04-0.05) |
| South Africa | 2257302 (2099020-2428551) | 9062.77 (8465.35-9680.97) | 4677573 (4347292-5025759) | 9097.4 (8501.89-9729.11) | 0 (-0.02-0.01) |
| South Sudan | 193641 (177480-211370) | 5618.84 (5175.07-6089.53) | 323627 (296973-354259) | 5730.31 (5317.92-6182.15) | 0.09 (0.08-0.1) |
| Spain | 2768974 (2588950-2935877) | 5464.92 (5131.06-5781.93) | 4288430 (3948305-4576051) | 5022.83 (4690.95-5348.81) | -0.25 (-0.28--0.22) |
| Sri Lanka | 1386481 (1270757-1506959) | 10274.64 (9504.26-11118.7) | 2647885 (2443533-2855363) | 10450.52 (9660.37-11299.19) | 0.11 (0.09-0.13) |
| Sudan | 1061226 (976894-1142517) | 8869.18 (8238.7-9502.05) | 2607644 (2388823-2834157) | 9227.47 (8545.45-9928.63) | 0.13 (0.11-0.14) |
| Suriname | 19697 (18356-21176) | 6671.59 (6215.35-7151.58) | 42747 (39950-45625) | 6878.34 (6435.81-7347.1) | 0.12 (0.11-0.13) |
| Sweden | 821047 (759582-881515) | 6415.63 (5942.97-6890.71) | 1121779 (1040388-1207272) | 6424.01 (5973.5-6887.77) | 0.06 (0.04-0.09) |
| Switzerland | 639460 (600031-681319) | 6582.84 (6179.02-7026.16) | 995630 (934109-1061548) | 6353.97 (5957.15-6788.6) | -0.13 (-0.19--0.07) |
| Syrian Arab Republic | 651505 (602699-705033) | 9137.89 (8502.54-9774.17) | 1213830 (1120670-1309607) | 8947.44 (8264.65-9632.36) | -0.08 (-0.09--0.07) |
| Taiwan (Province of China) | 1249266 (1163816-1344723) | 7155.39 (6728.12-7647.1) | 2418082 (2270339-2579826) | 6571.23 (6152.15-7040.81) | -0.26 (-0.28--0.24) |
| Tajikistan | 322282 (300325-346451) | 9924.67 (9264.55-10630.85) | 708588 (659872-761599) | 9851.2 (9246.71-10479.04) | -0.03 (-0.05--0.02) |
| Thailand | 4699377 (4333121-5101891) | 10448.07 (9717.11-11188.54) | 10328746 (9637500-11140578) | 10839.23 (10107.78-11724.93) | 0.1 (0.08-0.12) |
| Timor-Leste | 45151 (41110-49397) | 9936.45 (9194.89-10715.17) | 104170 (96163-112715) | 10410.83 (9601.84-11301.73) | 0.18 (0.17-0.19) |
| Togo | 131761 (121749-142772) | 7346 (6804.03-7869.05) | 382466 (353706-416128) | 7283.93 (6781.11-7815.85) | -0.07 (-0.09--0.05) |
| Tokelau | 107 (99-114) | 8149.51 (7578.01-8739.12) | 121 (112-130) | 8483.99 (7876.48-9158.2) | 0.16 (0.14-0.17) |
| Tonga | 5372 (4996-5776) | 8442.75 (7847.36-9065.87) | 7502 (6986-8081) | 8763.81 (8179.97-9417.79) | 0.16 (0.15-0.18) |
| Trinidad and Tobago | 64179 (59428-68941) | 6674.1 (6204.16-7119.34) | 124244 (115206-133282) | 7009.53 (6525.04-7524.91) | 0.17 (0.16-0.18) |
| Tunisia | 522181 (483654-562934) | 8690.33 (8101.63-9339.38) | 1144106 (1055790-1229704) | 8690.16 (8056.88-9347.17) | -0.01 (-0.02--0.01) |
| Türkiye | 254957 (238341-273926) | 11011.08 (10342.05-11708.9) | 484074 (452902-515793) | 10909.7 (10221.7-11599.92) | -0.02 (-0.03--0.01) |
| Turkmenistan | 609 (566-656) | 8324.33 (7745.82-8960.21) | 948 (879-1026) | 8641.39 (8027.92-9325.05) | 0.14 (0.13-0.15) |
| Tuvalu | 4172206 (3844649-4504302) | 9813.75 (9115.61-10517.89) | 8863959 (8169195-9513046) | 9503.67 (8759.62-10189.42) | -0.06 (-0.1--0.02) |
| Uganda | 554191 (507911-603268) | 6119.94 (5653.83-6571.6) | 1414668 (1296989-1547642) | 6081.41 (5638.5-6582.44) | -0.03 (-0.05-0) |
| Ukraine | 6244758 (5793184-6760948) | 9772.49 (9071.53-10538.34) | 6394485 (5969352-6867844) | 9814.15 (9113.94-10548.54) | 0 (0-0.01) |
| United Arab Emirates | 123088 (111138-138046) | 9906.14 (9179.72-10668.9) | 940579 (846409-1053023) | 9512.71 (8796.6-10259.41) | -0.14 (-0.16--0.12) |
| United Kingdom | 5130703 (4779037-5480493) | 6119.79 (5722.53-6520.16) | 6254851 (5837368-6635678) | 5449.07 (5106.17-5788.72) | -0.52 (-0.61--0.43) |
| United Republic of Tanzania | 793891 (729869-860415) | 5368.92 (4960.67-5794.76) | 2186544 (2008467-2390011) | 5861.53 (5421.89-6348.59) | 0.34 (0.32-0.37) |
| United States of America | 22892228 (21306718-24521743) | 7438.31 (6947.03-7942.8) | 38674171 (35778672-41193631) | 7550.75 (7050.6-8067.36) | 0.21 (0.15-0.26) |
| United States Virgin Islands | 6113 (5650-6602) | 6554.33 (6091.76-7043.68) | 9684 (8946-10346) | 6791.65 (6302.59-7285.97) | 0.13 (0.13-0.14) |
| Uruguay | 221996 (206709-237075) | 6192.59 (5767.88-6613.59) | 301874 (280956-322913) | 6282.78 (5818.03-6738.2) | 0.08 (0.06-0.1) |
| Uzbekistan | 1462693 (1370470-1565221) | 10757.79 (10084.06-11467.13) | 3273806 (3046385-3512972) | 11069.08 (10338.81-11793.57) | 0.12 (0.1-0.14) |
| Vanuatu | 7356 (6741-7986) | 8514.55 (7893.37-9206.91) | 19551 (18111-21165) | 8886.13 (8277.84-9532.41) | 0.16 (0.15-0.17) |
| Venezuela (Bolivarian Republic of) | 1015584 (943619-1092889) | 8453.02 (7868.51-9056.88) | 2442552 (2275365-2599385) | 8317.3 (7756.36-8846.76) | -0.06 (-0.09--0.02) |
| Viet Nam | 4431264 (4056446-4826467) | 9094.4 (8336.88-9858.13) | 9699996 (8886323-10558442) | 9152.06 (8434.76-9895.76) | 0.15 (0.1-0.2) |
| Yemen | 560437 (518019-609175) | 8318.03 (7725.77-8913.91) | 1752190 (1608715-1906736) | 8511.9 (7887.12-9137.9) | 0.07 (0.06-0.08) |
| Zambia | 231401 (212298-251949) | 5513.63 (5099.73-5947.91) | 662316 (603070-727722) | 5769.88 (5330.94-6256.55) | 0.2 (0.18-0.22) |
| Zimbabwe | 445253 (413595-477795) | 8575.21 (7995.12-9192.84) | 804662 (746942-870315) | 8832.91 (8242.96-9439.29) | 0.13 (0.12-0.14) |

| **TableS2: Trends in the burden of chronic kidney disease by SDI: incidence, prevalence, deaths, and disability-adjusted life years (1990–2021).** | | | | | |
| --- | --- | --- | --- | --- | --- |
| SDI | 1990 | | 2021 | | **EAPC_95%CI** |
|  | Number_95%UI | ASR | Number_95%UI | ASR |  |
| **Incidence** |  |  |  |  |  |
| High-middle SDI | 1574444 (1447925-1709190) | 160.44 (148.12-174.09) | 3917149 (3653537-4175391) | 205.9 (194.1-218.61) | 0.92 (0.89-0.95) |
| High SDI | 2789648 (2576788-3024433) | 252.3 (234.15-272.41) | 5666653 (5273251-6033381) | 277.75 (260.7-295.01) | 0.28 (0.24-0.32) |
| Low-middle SDI | 1107201 (1026640-1198009) | 153.07 (141.43-166.4) | 3130960 (2912350-3362301) | 204.97 (189.81-220.29) | 0.82 (0.76-0.88) |
| Low SDI | 348934 (324143-375458) | 121.74 (112.82-132.3) | 950557 (888494-1011543) | 155 (143.4-167.34) | 0.7 (0.62-0.78) |
| Middle SDI | 1963348 (1808277-2127370) | 171.16 (157.68-185.82) | 6251147 (5866964-6655539) | 232.96 (219.53-246.12) | 1.06 (1.04-1.08) |
| **Prevalence** |  |  |  |  |  |
| High-middle SDI | 78208075 (72803156-84086890) | 7641.21 (7149.96-8188.95) | 127111061 (118337580-136309239) | 7267.74 (6782.08-7816.7) | -0.09 (-0.14--0.04) |
| High SDI | 72796005 (68039542-77665594) | 6883.99 (6452.03-7322.72) | 120879407 (113333443-127967827) | 6733.55 (6322.09-7159.65) | -0.03 (-0.07-0.01) |
| Low-middle SDI | 70588690 (65338440-76043937) | 9292.53 (8660.54-9952.48) | 149861034 (139108422-161539154) | 9171.03 (8543.99-9848.15) | -0.07 (-0.1--0.05) |
| Low SDI | 23236078 (21506138-25034232) | 8090.37 (7566.41-8624.95) | 54528960 (50362040-59039888) | 7984.37 (7449.39-8549.73) | -0.07 (-0.09--0.05) |
| Middle SDI | 105819342 (98201190-113755650) | 8450.59 (7891.9-9069.19) | 220823054 (205500732-237345634) | 8280.06 (7728.02-8885.29) | 0.01 (-0.03-0.05) |
| **Deaths** |  |  |  |  |  |
| High-middle SDI | 99196 (91758-110668) | 11.36 (10.43-12.65) | 226797 (201672-252703) | 12.02 (10.68-13.38) | 0.25 (0.16-0.34) |
| High SDI | 100025 (92113-104189) | 9.22 (8.45-9.62) | 340083 (289016-369665) | 14.11 (12.3-15.21) | 1.73 (1.61-1.86) |
| Low-middle SDI | 110899 (98721-127673) | 18.59 (16.43-22.23) | 309509 (280315-349455) | 23.08 (20.97-26.31) | 0.71 (0.66-0.75) |
| Low SDI | 64656 (57360-73648) | 29.72 (26.31-34.62) | 136797 (118867-157579) | 29.43 (26.13-33.79) | -0.08 (-0.2-0.03) |
| Middle SDI | 177302 (162783-198065) | 19.07 (17.39-21.35) | 513051 (458865-556752) | 20.89 (18.45-22.67) | 0.38 (0.29-0.48) |
| **DALYs** |  |  |  |  |  |
| High-middle SDI | 3534406 (3184087-3954975) | 360.84 (325.48-401.53) | 5944277 (5372850-6623407) | 324.64 (293.58-360.92) | -0.35 (-0.42--0.28) |
| High SDI | 2898450 (2605440-3169018) | 277.64 (250.03-303.24) | 7115740 (6464112-7759250) | 358.51 (324.74-390.2) | 1.07 (0.99-1.16) |
| Low-middle SDI | 4690095 (4056126-5163160) | 609.44 (545.07-686.29) | 10611260 (9599821-11771755) | 686.98 (622.5-765.2) | 0.4 (0.38-0.42) |
| Low SDI | 2564545 (2265146-2870454) | 853.94 (760.14-971.76) | 5042405 (4408653-5841516) | 791.8 (704.14-909.1) | -0.34 (-0.42--0.25) |
| Middle SDI | 7031758 (6325501-7794727) | 585.56 (531.8-652.64) | 15700568 (14206921-17147169) | 596.45 (540.33-650.48) | 0.12 (0.04-0.2) |

| **TableS3: Trends in the burden of chronic kidney disease across different age groups: incidence, prevalence, deaths, and disability-adjusted life years (1990–2021).** | | | | | |
| --- | --- | --- | --- | --- | --- |
| Age | 1990 | | 2021 | | **EAPC_95%CI** |
|  | Number_95%UI | ASR | Number_95%UI | ASR |  |
| **Incidence** |  |  |  |  |  |
| <5 years | 413252 (344673-491290) | 66.66 (55.6-79.25) | 411195 (350263-480080) | 62.48 (53.22-72.94) | -0.17 (-0.19--0.15) |
| 5–9 years | 68502 (35310-113701) | 11.74 (6.05-19.48) | 87942 (47747-140374) | 12.8 (6.95-20.43) | 0.22 (0.1-0.33) |
| 10–14 years | 68300 (35177-113176) | 12.75 (6.57-21.13) | 116478 (69508-178075) | 17.47 (10.43-26.71) | 1.05 (1.01-1.1) |
| 15–19 years | 79998 (39868-131185) | 15.4 (7.68-25.26) | 138648 (82838-205213) | 22.22 (13.28-32.89) | 1.19 (1.14-1.24) |
| 20–24 years | 83728 (39380-135022) | 17.01 (8-27.44) | 131944 (74022-197404) | 22.1 (12.4-33.06) | 0.87 (0.84-0.9) |
| 25–29 years | 94454 (44225-159667) | 21.34 (9.99-36.07) | 148274 (76971-242291) | 25.2 (13.08-41.18) | 0.61 (0.58-0.64) |
| 30–34 years | 128041 (71537-194688) | 33.22 (18.56-50.51) | 240596 (145230-350045) | 39.8 (24.03-57.91) | 0.68 (0.61-0.75) |
| 35–39 years | 205256 (127126-297032) | 58.27 (36.09-84.33) | 413146 (263663-569502) | 73.66 (47.01-101.54) | 0.78 (0.7-0.85) |
| 40–44 years | 279528 (186866-400652) | 97.57 (65.23-139.85) | 616803 (438856-816932) | 123.3 (87.73-163.3) | 0.73 (0.67-0.79) |
| 45–49 years | 359626 (237525-486938) | 154.88 (102.3-209.71) | 901200 (628260-1150613) | 190.33 (132.68-243) | 0.67 (0.63-0.71) |
| 50–54 years | 520184 (343501-715549) | 244.71 (161.59-336.62) | 1376940 (979620-1829922) | 309.48 (220.18-411.29) | 0.82 (0.77-0.88) |
| 55–59 years | 702420 (475552-932721) | 379.28 (256.78-503.63) | 1877640 (1399991-2343011) | 474.48 (353.78-592.08) | 0.82 (0.78-0.86) |
| 60–64 years | 925207 (641743-1237035) | 576.06 (399.57-770.21) | 2348771 (1753270-3004975) | 733.88 (547.82-938.91) | 0.75 (0.73-0.77) |
| 65–69 years | 1060554 (735951-1396842) | 857.99 (595.38-1130.04) | 2876743 (2158191-3764727) | 1042.89 (782.4-1364.81) | 0.69 (0.67-0.71) |
| 70–74 years | 988341 (731608-1276667) | 1167.41 (864.16-1507.97) | 2990771 (2340116-3765114) | 1452.96 (1136.87-1829.15) | 0.72 (0.7-0.74) |
| 75–79 years | 889725 (634835-1185677) | 1445.4 (1031.32-1926.19) | 2367398 (1791214-2982556) | 1795.06 (1358.17-2261.49) | 0.66 (0.62-0.7) |
| 80–84 years | 595143 (424970-798993) | 1682.34 (1201.3-2258.58) | 1750386 (1277496-2262851) | 1998.54 (1458.61-2583.66) | 0.52 (0.47-0.57) |
| 85–89 years | 251459 (176089-344052) | 1664.07 (1165.3-2276.82) | 789943 (573410-1034709) | 1727.72 (1254.13-2263.06) | 0.11 (0.07-0.14) |
| 90–94 years | 63700 (44569-86403) | 1486.52 (1040.07-2016.31) | 271193 (199408-346529) | 1515.94 (1114.67-1937.06) | 0.1 (0.02-0.19) |
| 95+ years | 13288 (7825-20183) | 1305.24 (768.57-1982.4) | 79026 (48424-113740) | 1449.93 (888.46-2086.85) | 0.34 (0.2-0.49) |
| **Prevalence** |  |  |  |  |  |
| <5 years | 315088 (275868-358222) | 50.83 (44.5-57.78) | 328932 (272290-400565) | 49.98 (41.37-60.86) | -0.15 (-0.19--0.1) |
| 5–9 years | 1040068 (888223-1247189) | 178.24 (152.22-213.73) | 1040775 (856529-1301199) | 151.48 (124.67-189.39) | -0.58 (-0.62--0.54) |
| 10–14 years | 3014083 (2434375-3701054) | 562.66 (454.44-690.9) | 3515811 (2758385-4396615) | 527.4 (413.78-659.52) | -0.21 (-0.22--0.19) |
| 15–19 years | 9348569 (7309255-11705929) | 1799.8 (1407.19-2253.64) | 11724708 (9043292-14878162) | 1879.02 (1449.29-2384.4) | 0.13 (0.1-0.17) |
| 20–24 years | 19162023 (14782374-24917274) | 3894.02 (3004.01-5063.58) | 25188822 (19182883-32987497) | 4218.12 (3212.36-5524.08) | 0.22 (0.18-0.27) |
| 25–29 years | 25796978 (19836837-32443648) | 5828.25 (4481.69-7329.92) | 36603713 (27923652-46345398) | 6221.49 (4746.15-7877.27) | 0.22 (0.2-0.23) |
| 30–34 years | 28691096 (22685954-36072544) | 7444.06 (5886-9359.22) | 46926748 (36683747-59200075) | 7763.16 (6068.64-9793.55) | 0.18 (0.15-0.22) |
| 35–39 years | 29627604 (23304755-37370072) | 8411.09 (6616.07-10609.13) | 49439849 (38555212-62716489) | 8814.91 (6874.23-11182.08) | 0.19 (0.14-0.24) |
| 40–44 years | 25976735 (20566356-31832200) | 9067.5 (7178.94-11111.43) | 47464382 (37302324-58405441) | 9488.12 (7456.72-11675.23) | 0.16 (0.11-0.2) |
| 45–49 years | 23913862 (19739600-29641677) | 10299.02 (8501.28-12765.83) | 49761987 (40547232-62143159) | 10509.29 (8563.22-13124.09) | 0.1 (0.06-0.14) |
| 50–54 years | 24890243 (20319590-29501606) | 11709.12 (9558.95-13878.44) | 51815812 (41656848-62349186) | 11646.02 (9362.71-14013.48) | 0.05 (0-0.1) |
| 55–59 years | 25740750 (21498603-30961060) | 13898.9 (11608.32-16717.64) | 53686965 (44530918-64707521) | 13566.63 (11252.91-16351.51) | 0 (-0.06-0.06) |
| 60–64 years | 27823278 (23823972-32425248) | 17323.59 (14833.5-20188.91) | 53756190 (45825573-63023034) | 16796.3 (14318.35-19691.75) | -0.09 (-0.13--0.04) |
| 65–69 years | 27457862 (23456676-31868811) | 22213.4 (18976.44-25781.86) | 57959585 (49228634-67745201) | 21011.87 (17846.67-24559.41) | -0.15 (-0.17--0.12) |
| 70–74 years | 25334512 (22011940-28700184) | 29924.6 (26000.05-33900.06) | 57544654 (49509967-65187159) | 27956.12 (24052.74-31668.97) | -0.15 (-0.18--0.11) |
| 75–79 years | 23895843 (20958893-26583095) | 38819.99 (34048.77-43185.56) | 48381798 (42205592-53895824) | 36685 (32001.96-40865.96) | -0.14 (-0.18--0.11) |
| 80–84 years | 16905963 (15086156-18680891) | 47789.45 (42645.26-52806.79) | 39518437 (35079861-43675611) | 45121.1 (40053.25-49867.65) | -0.16 (-0.18--0.14) |
| 85–89 years | 8513842 (7682623-9393977) | 56341.75 (50841.02-62166.18) | 24280930 (21959600-26581983) | 53105.81 (48028.74-58138.54) | -0.16 (-0.17--0.14) |
| 90–94 years | 2777694 (2519946-3047649) | 64820.83 (58805.96-71120.55) | 11010281 (10059859-12045399) | 61546.49 (56233.72-67332.71) | -0.14 (-0.15--0.12) |
| 95+ years | 736581 (665079-806852) | 72349.54 (65326.35-79251.8) | 3772324 (3409259-4153970) | 69213.03 (62551.68-76215.31) | -0.12 (-0.14--0.11) |
| **Deaths** |  |  |  |  |  |
| <5 years | 16380 (11106-18941) | 2.64 (1.79-3.06) | 8440 (6460-10258) | 1.28 (0.98-1.56) | -2.1 (-2.18--2.01) |
| 5–9 years | 5026 (3224-5983) | 0.86 (0.55-1.03) | 3566 (2897-4106) | 0.52 (0.42-0.6) | -1.37 (-1.49--1.26) |
| 10–14 years | 3947 (3183-4388) | 0.74 (0.59-0.82) | 4103 (3569-4673) | 0.62 (0.54-0.7) | -0.39 (-0.47--0.32) |
| 15–19 years | 7881 (6750-8846) | 1.52 (1.3-1.7) | 9514 (8292-10783) | 1.52 (1.33-1.73) | -0.08 (-0.13--0.02) |
| 20–24 years | 9725 (8493-10885) | 1.98 (1.73-2.21) | 12581 (11186-13994) | 2.11 (1.87-2.34) | -0.01 (-0.11-0.09) |
| 25–29 years | 10394 (9276-11723) | 2.35 (2.1-2.65) | 14958 (13545-16469) | 2.54 (2.3-2.8) | 0.04 (-0.07-0.16) |
| 30–34 years | 12133 (10973-13589) | 3.15 (2.85-3.53) | 19333 (17485-21075) | 3.2 (2.89-3.49) | -0.09 (-0.17-0) |
| 35–39 years | 16149 (14737-17943) | 4.58 (4.18-5.09) | 26088 (23782-28727) | 4.65 (4.24-5.12) | -0.03 (-0.11-0.06) |
| 40–44 years | 18978 (16653-20991) | 6.62 (5.81-7.33) | 36181 (32998-39414) | 7.23 (6.6-7.88) | 0.09 (-0.01-0.2) |
| 45–49 years | 21859 (19824-24294) | 9.41 (8.54-10.46) | 48813 (43974-53451) | 10.31 (9.29-11.29) | 0.27 (0.22-0.32) |
| 50–54 years | 31749 (29418-35231) | 14.94 (13.84-16.57) | 73336 (66635-79438) | 16.48 (14.98-17.85) | 0.28 (0.25-0.31) |
| 55–59 years | 40971 (37710-45844) | 22.12 (20.36-24.75) | 101628 (93189-110959) | 25.68 (23.55-28.04) | 0.49 (0.4-0.57) |
| 60–64 years | 50477 (46802-57428) | 31.43 (29.14-35.76) | 123934 (114304-135517) | 38.72 (35.71-42.34) | 0.58 (0.5-0.65) |
| 65–69 years | 57230 (52985-64804) | 46.3 (42.87-52.43) | 155649 (143526-167401) | 56.43 (52.03-60.69) | 0.68 (0.58-0.78) |
| 70–74 years | 61376 (56429-70267) | 72.5 (66.65-83) | 177139 (164653-191749) | 86.06 (79.99-93.15) | 0.7 (0.63-0.78) |
| 75–79 years | 65360 (60193-73250) | 106.18 (97.79-119) | 177758 (162652-189622) | 134.78 (123.33-143.78) | 0.79 (0.75-0.82) |
| 80–84 years | 57002 (51437-64059) | 161.13 (145.4-181.08) | 183851 (161990-199792) | 209.92 (184.96-228.12) | 1.04 (0.95-1.14) |
| 85–89 years | 42164 (37425-47167) | 279.03 (247.66-312.13) | 177386 (147637-193976) | 387.97 (322.9-424.25) | 1.41 (1.27-1.55) |
| 90–94 years | 18119 (15162-20265) | 422.83 (353.83-472.91) | 117515 (93028-130224) | 656.9 (520.02-727.94) | 1.83 (1.67-1.99) |
| 95+ years | 5752 (4470-6477) | 564.97 (439.09-636.18) | 55867 (40448-63833) | 1025.02 (742.11-1171.18) | 2.29 (2.14-2.44) |
| **DALYs** |  |  |  |  |  |
| <5 years | 1476816 (1016346-1698744) | 238.22 (163.94-274.02) | 783688 (608082-943773) | 119.07 (92.39-143.39) | -2.01 (-2.09--1.93) |
| 5–9 years | 464741 (312046-548841) | 79.64 (53.48-94.06) | 349678 (285919-400940) | 50.9 (41.62-58.36) | -1.23 (-1.32--1.14) |
| 10–14 years | 363924 (299371-415119) | 67.94 (55.89-77.49) | 380998 (331983-431903) | 57.15 (49.8-64.79) | -0.42 (-0.48--0.37) |
| 15–19 years | 668428 (573573-753063) | 128.69 (110.42-144.98) | 797196 (697774-910717) | 127.76 (111.83-145.95) | -0.12 (-0.18--0.07) |
| 20–24 years | 796330 (691717-915786) | 161.83 (140.57-186.1) | 1012023 (886500-1132616) | 169.47 (148.45-189.67) | -0.04 (-0.13-0.04) |
| 25–29 years | 838538 (732936-969503) | 189.45 (165.59-219.04) | 1185523 (1041978-1348165) | 201.5 (177.1-229.15) | 0.03 (-0.06-0.12) |
| 30–34 years | 926430 (808452-1076922) | 240.37 (209.76-279.41) | 1471579 (1278839-1696471) | 243.45 (211.56-280.65) | -0.04 (-0.1-0.02) |
| 35–39 years | 1115793 (986354-1293826) | 316.77 (280.02-367.31) | 1813289 (1603450-2088274) | 323.3 (285.89-372.33) | 0.03 (-0.03-0.09) |
| 40–44 years | 1178619 (1035286-1337677) | 411.41 (361.38-466.93) | 2230284 (1983808-2524763) | 445.83 (396.56-504.7) | 0.13 (0.05-0.2) |
| 45–49 years | 1216628 (1083292-1376012) | 523.97 (466.54-592.61) | 2691291 (2400805-3005086) | 568.38 (507.03-634.65) | 0.27 (0.23-0.31) |
| 50–54 years | 1531234 (1390751-1715156) | 720.34 (654.25-806.86) | 3506263 (3158788-3940982) | 788.06 (709.96-885.77) | 0.29 (0.26-0.31) |
| 55–59 years | 1732903 (1560509-1960291) | 935.69 (842.61-1058.47) | 4203942 (3786691-4750050) | 1062.33 (956.89-1200.33) | 0.45 (0.39-0.52) |
| 60–64 years | 1851633 (1657075-2101728) | 1152.88 (1031.74-1308.6) | 4377372 (3955948-4910554) | 1367.72 (1236.05-1534.32) | 0.51 (0.45-0.58) |
| 65–69 years | 1778806 (1606983-2023827) | 1439.05 (1300.05-1637.27) | 4645441 (4254097-5085305) | 1684.09 (1542.22-1843.56) | 0.59 (0.49-0.68) |
| 70–74 years | 1572650 (1414533-1789528) | 1857.58 (1670.82-2113.75) | 4383629 (4010471-4801099) | 2129.64 (1948.35-2332.45) | 0.6 (0.53-0.68) |
| 75–79 years | 1377167 (1244151-1542303) | 2237.28 (2021.19-2505.55) | 3581478 (3246795-3914141) | 2715.62 (2461.85-2967.86) | 0.66 (0.62-0.7) |
| 80–84 years | 971119 (866734-1092267) | 2745.14 (2450.07-3087.6) | 2941752 (2607163-3250378) | 3358.81 (2976.79-3711.19) | 0.83 (0.74-0.92) |
| 85–89 years | 576261 (504857-650987) | 3813.5 (3340.97-4308.02) | 2218239 (1887504-2475952) | 4851.6 (4128.24-5415.26) | 1.06 (0.95-1.17) |
| 90–94 years | 228333 (194265-259997) | 5328.42 (4533.41-6067.34) | 1297391 (1067745-1466295) | 7252.3 (5968.6-8196.46) | 1.3 (1.19-1.42) |
| 95+ years | 73545 (59900-85938) | 7223.82 (5883.6-8441.16) | 582626 (453032-670543) | 10689.78 (8312.05-12302.85) | 1.55 (1.45-1.64) |

| **TableS4: Trends in the burden of chronic kidney disease due to hypertension by SDI: incidence, prevalence, deaths, and disability-adjusted life years (1990–2021).** | | | | | |
| --- | --- | --- | --- | --- | --- |
| SDI | 1990 | | 2021 | | **EAPC_95%CI** |
|  | Number_95%UI | ASR | Number_95%UI | ASR |  |
| **Incidence** |  |  |  |  |  |
| High-middle SDI | 95847 (87157-105017) | 10.05 (9.23-11.01) | 261612 (242543-280128) | 13.26 (12.33-14.18) | 1 (0.97-1.03) |
| High SDI | 190961 (175653-209973) | 16.83 (15.57-18.35) | 410383 (380798-438430) | 18.85 (17.6-20.1) | 0.34 (0.3-0.38) |
| Low-middle SDI | 55821 (50787-61356) | 9.13 (8.32-10.05) | 178746 (164553-193874) | 12.48 (11.52-13.56) | 0.88 (0.81-0.94) |
| Low SDI | 16147 (14585-17743) | 7.34 (6.67-8.01) | 47366 (43044-51429) | 9.51 (8.66-10.36) | 0.75 (0.67-0.83) |
| Middle SDI | 104728 (94930-115150) | 10.32 (9.42-11.32) | 382916 (355847-407967) | 14.36 (13.41-15.3) | 1.13 (1.11-1.15) |
| **Prevalence** |  |  |  |  |  |
| High-middle SDI | 2644466 (2456190-2853366) | 283.65 (264.47-305.48) | 4691235 (4368783-5037826) | 246.07 (229.28-263.95) | -0.37 (-0.41--0.33) |
| High SDI | 3327902 (3090476-3607872) | 300.99 (279.95-325.27) | 6485801 (6030426-6971249) | 298.79 (279.36-320.13) | 0 (-0.02-0.03) |
| Low-middle SDI | 1904070 (1762081-2055558) | 326.55 (303.2-353) | 4313875 (4016708-4626611) | 311.16 (289.73-333.12) | -0.15 (-0.17--0.13) |
| Low SDI | 644444 (590736-697557) | 300.18 (277.46-323.75) | 1446438 (1340396-1551763) | 286.5 (265.93-307.27) | -0.16 (-0.17--0.15) |
| Middle SDI | 3180093 (2935071-3442168) | 324.01 (299.52-349.9) | 7510313 (6981613-8056655) | 293.8 (273.89-315.54) | -0.21 (-0.26--0.17) |
| **Deaths** |  |  |  |  |  |
| High-middle SDI | 24909 (20319-30229) | 2.98 (2.44-3.58) | 64745 (52653-78133) | 3.42 (2.78-4.13) | 0.57 (0.5-0.65) |
| High SDI | 25961 (21214-31197) | 2.4 (1.95-2.86) | 102338 (81584-118817) | 4.09 (3.34-4.71) | 2.23 (2.07-2.39) |
| Low-middle SDI | 28277 (22512-35715) | 5.46 (4.37-6.99) | 83172 (67290-99390) | 6.62 (5.44-7.85) | 0.61 (0.55-0.66) |
| Low SDI | 15305 (12181-18932) | 8.69 (7.03-10.73) | 33880 (27407-41452) | 8.62 (7.09-10.51) | -0.08 (-0.2-0.04) |
| Middle SDI | 54388 (45047-64626) | 6.53 (5.39-7.75) | 169822 (140032-198167) | 7.13 (5.86-8.28) | 0.34 (0.25-0.42) |
| **DALYs** |  |  |  |  |  |
| High-middle SDI | 24909 (20319-30229) | 2.98 (2.44-3.58) | 64745 (52653-78133) | 3.42 (2.78-4.13) | 0.57 (0.5-0.65) |
| High SDI | 25961 (21214-31197) | 2.4 (1.95-2.86) | 102338 (81584-118817) | 4.09 (3.34-4.71) | 2.23 (2.07-2.39) |
| Low-middle SDI | 28277 (22512-35715) | 5.46 (4.37-6.99) | 83172 (67290-99390) | 6.62 (5.44-7.85) | 0.61 (0.55-0.66) |
| Low SDI | 15305 (12181-18932) | 8.69 (7.03-10.73) | 33880 (27407-41452) | 8.62 (7.09-10.51) | -0.08 (-0.2-0.04) |
| Middle SDI | 54388 (45047-64626) | 6.53 (5.39-7.75) | 169822 (140032-198167) | 7.13 (5.86-8.28) | 0.34 (0.25-0.42) |

| **TableS5: National Burden of chronic kidney disease: incidence Cases, ASIR, and EAPC (1990–2021).** | | | | | |
| --- | --- | --- | --- | --- | --- |
| Country | **1990** | | **2021** | | **EAPC_95%CI** |
|  | Number_95%UI | ASR | Number_95%UI | ASR |  |
| Afghanistan | 18833 (16975-21041) | 255.14 (230.97-282.18) | 45514 (42294-49787) | 367.19 (338.49-402.22) | 1.1 (0.91-1.28) |
| Albania | 2815 (2542-3126) | 120.75 (108.14-134.53) | 8621 (7734-9518) | 206.7 (188.54-225.03) | 2.05 (1.87-2.23) |
| Algeria | 35771 (32578-39279) | 256.61 (233.4-281.88) | 153075 (140677-165614) | 407.45 (375.52-438.8) | 1.42 (1.35-1.48) |
| American Samoa | 61 (57-66) | 212.79 (195.75-230.96) | 156 (143-171) | 312.56 (289.11-337.2) | 1.25 (1.17-1.33) |
| Andorra | 126 (113-142) | 224.52 (203.43-250.77) | 336 (307-370) | 222.64 (202.74-245.42) | 0.08 (0.01-0.15) |
| Angola | 4839 (4507-5162) | 89.54 (82.64-97.1) | 19883 (18458-21540) | 129.13 (118.15-140.51) | 1.15 (0.98-1.33) |
| Antigua and Barbuda | 114 (105-124) | 219.08 (200.99-238.07) | 375 (343-409) | 344.75 (318.73-371.78) | 1.55 (1.43-1.66) |
| Argentina | 68543 (62476-75476) | 213.61 (195.31-234.45) | 150963 (137930-163854) | 269.56 (246.95-290.68) | 0.9 (0.78-1.01) |
| Armenia | 2819 (2506-3157) | 94.28 (84.4-105.1) | 7532 (6762-8397) | 184.33 (168.31-201.69) | 2.5 (2.35-2.66) |
| Australia | 51807 (49084-54962) | 258.05 (245-272.1) | 135305 (123723-145659) | 296.27 (272-317.25) | 0.45 (0.39-0.51) |
| Austria | 27499 (24724-30726) | 224.6 (203.96-247.29) | 50647 (46366-55368) | 275.88 (249.36-302.98) | 0.79 (0.72-0.86) |
| Azerbaijan | 6575 (5913-7257) | 110.18 (99.67-121.02) | 22387 (19939-24791) | 203.81 (187.05-221.06) | 2.32 (2.13-2.52) |
| Bahamas | 346 (321-374) | 197.33 (182.27-216.2) | 1252 (1141-1353) | 295.86 (274.03-317.19) | 1.47 (1.37-1.58) |
| Bahrain | 612 (557-676) | 292.78 (268-320.81) | 4873 (4361-5457) | 451.21 (413.39-487.18) | 1.36 (1.3-1.42) |
| Bangladesh | 52608 (48339-57513) | 89.98 (81.87-99.39) | 187707 (170316-207797) | 131.83 (119.95-144.85) | 1.14 (1.04-1.24) |
| Barbados | 523 (480-576) | 194.41 (178.58-213.82) | 1473 (1348-1605) | 308 (286.36-332.91) | 1.55 (1.44-1.65) |
| Belarus | 10524 (9525-11767) | 87.22 (79.76-96.22) | 20010 (18173-22131) | 141.29 (128.88-154.23) | 1.61 (1.39-1.84) |
| Belgium | 38016 (34545-42363) | 240.48 (220.28-264.1) | 58480 (53425-63159) | 247.39 (226.54-265.93) | 0.2 (0.15-0.25) |
| Belize | 213 (197-231) | 193.55 (176.1-212.41) | 1052 (972-1129) | 312.05 (288.73-335.71) | 1.55 (1.47-1.63) |
| Benin | 3701 (3454-3984) | 135.76 (124.78-147.44) | 12189 (11329-13016) | 177.75 (162.42-192.63) | 0.84 (0.76-0.91) |
| Bermuda | 121 (110-133) | 192.65 (175.32-211.44) | 392 (356-428) | 321.1 (295.63-348.34) | 1.88 (1.68-2.08) |
| Bhutan | 425 (391-459) | 125.4 (115.46-137.73) | 1197 (1094-1305) | 185.79 (169.68-202.65) | 1.42 (1.35-1.48) |
| Bolivia (Plurinational State of) | 6471 (5994-6956) | 175.99 (162-190.05) | 24667 (22843-26922) | 262.38 (242.5-285.89) | 1.46 (1.41-1.52) |
| Bosnia and Herzegovina | 5153 (4623-5700) | 121.04 (109.7-132.48) | 13515 (12363-14759) | 227.92 (209.44-246.38) | 2.29 (2.12-2.47) |
| Botswana | 1010 (928-1097) | 151.59 (138.71-165.3) | 3507 (3222-3805) | 217.22 (199.9-237.03) | 1.09 (0.91-1.27) |
| Brazil | 189483 (174873-205093) | 192.91 (176.91-209.57) | 644744 (602333-688117) | 258.11 (241.7-274.48) | 0.89 (0.84-0.94) |
| Brunei Darussalam | 322 (296-352) | 295.01 (269.33-322.69) | 1126 (1022-1250) | 328.34 (302.52-357.58) | 0.42 (0.33-0.52) |
| Bulgaria | 15334 (13681-17204) | 129.03 (117.32-142.46) | 29662 (26863-32623) | 232.22 (214.99-250.97) | 1.95 (1.89-2) |
| Burkina Faso | 7168 (6643-7771) | 126.47 (116.58-138.3) | 20261 (19126-21650) | 165.8 (153.53-179.63) | 0.95 (0.88-1.02) |
| Burundi | 2856 (2658-3083) | 93.91 (86.38-102.24) | 6740 (6244-7274) | 109.68 (101.01-119.57) | 0.44 (0.33-0.54) |
| C?te d'Ivoire | 304 (280-331) | 114.82 (105.2-125.22) | 816 (742-885) | 179.47 (162.8-195.61) | 1.51 (1.38-1.64) |
| Cabo Verde | 7527 (6954-8200) | 126.42 (115.83-139.56) | 24542 (22491-27011) | 183.25 (168.5-200.91) | 1.19 (1.05-1.32) |
| Cambodia | 10676 (9919-11509) | 178.41 (163.99-194.72) | 39453 (36599-41901) | 236.22 (219.22-253.76) | 0.84 (0.73-0.94) |
| Cameroon | 85191 (77748-90551) | 260.65 (239.01-276.27) | 184153 (167875-204154) | 256.43 (234.42-282.28) | 0.24 (0.14-0.34) |
| Canada | 1379 (1268-1499) | 91.18 (83.57-99.19) | 3387 (3140-3657) | 118.17 (109.2-128.46) | 0.78 (0.65-0.9) |
| Central African Republic | 4705 (4391-5083) | 127.55 (117.01-139.45) | 13105 (12245-14003) | 154.87 (142.44-168.79) | 0.57 (0.42-0.73) |
| Chad | 21716 (19660-24017) | 216.57 (196.57-239.17) | 80300 (73877-86347) | 316.55 (292.45-340) | 1.47 (1.31-1.62) |
| Chile | 1267533 (1145753-1385805) | 147.29 (133.86-161.46) | 3323175 (3068978-3559324) | 163.74 (153.03-174.11) | 0.52 (0.44-0.59) |
| China | 45649 (41528-50029) | 227.23 (206.64-250.95) | 171450 (156961-186839) | 314.56 (289.42-341.07) | 1.21 (1.17-1.26) |
| Colombia | 268 (249-290) | 103.43 (95.25-112.63) | 672 (627-725) | 127.5 (118.48-138.78) | 0.57 (0.5-0.64) |
| Comoros | 1393 (1291-1512) | 102.25 (94.45-111.22) | 4645 (4325-5062) | 147.09 (136.7-160) | 1.13 (1.01-1.24) |
| Congo | 24 (22-26) | 174.21 (158.51-190.06) | 70 (63-76) | 284.54 (260.54-308.16) | 1.62 (1.52-1.73) |
| Cook Islands | 8200 (7966-8432) | 436.29 (425.05-446.44) | 24923 (24422-25363) | 454.46 (445.16-462.62) | 0.14 (0.14-0.14) |
| Costa Rica | 9145 (8175-10238) | 150.56 (136.31-166.12) | 21856 (19855-23659) | 259.73 (238.67-279.34) | 2.01 (1.87-2.15) |
| Croatia | 15078 (13825-16515) | 146.81 (133.91-160.69) | 49193 (44769-53427) | 271.14 (248.75-295.36) | 2.18 (2.07-2.28) |
| Cuba | 2321 (2070-2607) | 275.5 (254.54-300.59) | 6413 (5860-7052) | 296.02 (272.09-324.03) | 0.27 (0.16-0.39) |
| Cyprus | 17191 (15474-19227) | 129.98 (118.05-143.63) | 44762 (40513-49824) | 217.99 (200.55-238.47) | 1.73 (1.55-1.9) |
| Czechia | 9012 (8352-9722) | 150.85 (139.01-164.29) | 28314 (26275-30296) | 193.17 (178.04-209.93) | 0.71 (0.61-0.8) |
| Democratic People's Republic of Korea | 23090 (20851-25212) | 138.39 (125.15-150.99) | 58636 (52975-64306) | 185.31 (168.51-201.74) | 0.98 (0.93-1.03) |
| Democratic Republic of the Congo | 19562 (18062-21127) | 94.59 (86.76-103.22) | 57306 (53005-62101) | 128.3 (117.98-140.77) | 0.92 (0.78-1.07) |
| Denmark | 17126 (15298-19107) | 207.06 (187.32-228.28) | 29642 (27034-32472) | 243.44 (224.01-263.12) | 0.66 (0.61-0.72) |
| Djibouti | 185 (171-200) | 98.69 (90.6-107.55) | 936 (862-1011) | 128.56 (117.96-139) | 0.75 (0.6-0.9) |
| Dominica | 139 (129-151) | 229.62 (212.37-247.74) | 262 (238-285) | 320.81 (295.72-345.22) | 1.09 (1-1.17) |
| Dominican Republic | 6202 (5697-6821) | 139.23 (126.43-153.72) | 24963 (23057-26895) | 243.01 (223.96-261.73) | 1.94 (1.86-2.02) |
| Ecuador | 10748 (9822-11799) | 180.68 (163.64-199.76) | 57934 (53462-63236) | 348.19 (321.29-379.96) | 2.44 (2.3-2.57) |
| Egypt | 80783 (73484-89103) | 263.81 (240.7-289.49) | 301550 (272715-333684) | 428.76 (397.28-465.61) | 1.49 (1.42-1.57) |
| El Salvador | 7712 (7063-8457) | 229.52 (209.07-253.91) | 24978 (23302-26600) | 408.39 (379.98-435.7) | 2.28 (2.13-2.44) |
| Equatorial Guinea | 227 (210-246) | 92.55 (85.25-101.49) | 1065 (987-1146) | 165.2 (151.27-180.45) | 2.16 (2.02-2.3) |
| Eritrea | 1470 (1358-1586) | 91.2 (83.99-99.69) | 3984 (3698-4299) | 116.86 (106.79-127.37) | 0.68 (0.55-0.81) |
| Estonia | 1993 (1779-2238) | 104.45 (94.27-116.27) | 4909 (4493-5418) | 215.89 (198.03-235.66) | 2.62 (2.52-2.71) |
| Eswatini | 689 (635-750) | 184.48 (169.23-202.93) | 1568 (1441-1700) | 232.86 (212.29-252.21) | 0.57 (0.36-0.78) |
| Ethiopia | 25075 (23288-27040) | 92.87 (85.97-100.67) | 60892 (56785-64771) | 114.61 (104.99-124.17) | 0.55 (0.4-0.7) |
| Fiji | 881 (811-959) | 195.28 (179.74-212.95) | 2163 (1978-2345) | 261.89 (243.66-283.22) | 0.86 (0.81-0.91) |
| Finland | 12464 (11114-14015) | 171.14 (154.02-189.57) | 25918 (23654-28781) | 195.36 (180.13-214.08) | 0.44 (0.37-0.51) |
| France | 179845 (162635-201949) | 207.47 (188.8-229.56) | 326611 (300075-352984) | 229.79 (212.38-249.84) | 0.36 (0.3-0.42) |
| Gabon | 755 (698-818) | 115.79 (106.83-125.64) | 2115 (1944-2293) | 182.33 (168.31-196.86) | 1.42 (1.3-1.55) |
| Gambia | 662 (623-706) | 133.86 (123.59-144.69) | 2198 (2064-2346) | 176.56 (162.39-191.77) | 0.86 (0.74-0.98) |
| Georgia | 6362 (5720-7116) | 105.04 (95.24-116.56) | 9241 (8320-10163) | 177.75 (164.07-193.02) | 1.94 (1.79-2.09) |
| Germany | 312633 (283719-347849) | 242.78 (222.26-269.74) | 526670 (491104-565042) | 269.85 (254.46-287.45) | 0.38 (0.35-0.41) |
| Ghana | 9860 (9230-10595) | 120.08 (111.48-130.61) | 34499 (31872-37394) | 171.14 (157.26-186.33) | 1.16 (1.06-1.26) |
| Greece | 44006 (40001-48285) | 283.94 (258.67-310.51) | 65278 (60702-70955) | 274.86 (254.18-297.79) | 0 (-0.09-0.09) |
| Greenland | 60 (54-65) | 200.98 (181.77-220.52) | 149 (136-165) | 231.05 (213.58-252.8) | 0.59 (0.53-0.65) |
| Grenada | 149 (139-160) | 210.49 (194.11-227.44) | 413 (380-447) | 355.05 (328.52-380.04) | 1.65 (1.54-1.76) |
| Guam | 166 (152-183) | 180.83 (165.51-199.06) | 534 (488-587) | 265.31 (244.89-289.5) | 1.28 (1.19-1.37) |
| Guatemala | 10599 (9674-11591) | 244.05 (221.48-267) | 45885 (42467-49772) | 387.9 (358.04-420.42) | 1.65 (1.56-1.74) |
| Guinea | 5459 (5060-5901) | 134.1 (123.08-146.22) | 12458 (11579-13322) | 171.1 (157.52-184.4) | 0.73 (0.66-0.81) |
| Guinea-Bissau | 795 (741-851) | 142.9 (133.39-154.42) | 1762 (1638-1884) | 170.44 (157.21-184.07) | 0.52 (0.41-0.62) |
| Guyana | 836 (776-899) | 177.15 (163.43-191.62) | 2046 (1880-2219) | 294.58 (273.7-317.6) | 1.64 (1.59-1.7) |
| Haiti | 6772 (6073-7507) | 170.38 (152.56-191.54) | 19290 (17777-20946) | 225.97 (207.08-245.88) | 0.96 (0.91-1.01) |
| Honduras | 5948 (5452-6582) | 236.15 (214.11-264.73) | 22686 (20945-24585) | 320.5 (295.77-348.5) | 1.11 (1.07-1.15) |
| Hungary | 15676 (14056-17503) | 111.68 (101.48-123.79) | 42185 (38412-46628) | 227.65 (209.3-248.77) | 2.46 (2.31-2.61) |
| Iceland | 560 (509-616) | 192.18 (175.12-210.84) | 1166 (1066-1264) | 199.78 (182.82-216.94) | 0.15 (0.05-0.25) |
| India | 849302 (780462-920171) | 152.76 (140.36-165.72) | 2235892 (2056366-2414555) | 180.72 (166.53-195.39) | 0.33 (0.24-0.42) |
| Indonesia | 155644 (144275-167963) | 130.27 (120.15-141.66) | 469570 (432377-511150) | 185.09 (171.22-200.1) | 0.99 (0.86-1.13) |
| Iran (Islamic Republic of) | 93053 (83778-103125) | 304.23 (277.32-334.65) | 330887 (308639-354798) | 408.97 (382.61-436) | 0.9 (0.85-0.95) |
| Iraq | 27066 (24993-29348) | 291.5 (267.28-319.75) | 117615 (108689-127825) | 440.28 (407.19-473.91) | 1.31 (1.19-1.44) |
| Ireland | 11405 (10693-11949) | 273.96 (257.21-285.84) | 19201 (18876-19506) | 245.55 (242.28-248.78) | -0.16 (-0.22--0.11) |
| Israel | 13680 (12393-15202) | 273.62 (249.31-301.51) | 37867 (34116-41515) | 305.76 (275.26-333.62) | 0.47 (0.35-0.58) |
| Italy | 195977 (175595-217213) | 217.42 (196.25-239.32) | 333142 (306700-361005) | 218.54 (202.72-235.12) | 0.13 (0.09-0.17) |
| Jamaica | 3710 (3408-4045) | 203.9 (186.17-222.8) | 8989 (8236-9704) | 295.44 (271.45-318.44) | 1.32 (1.2-1.44) |
| Japan | 471525 (434120-511167) | 280.03 (259.15-302.1) | 996708 (911293-1081250) | 285.18 (263.72-305.66) | 0.11 (0.06-0.17) |
| Jordan | 4664 (4322-5035) | 289.6 (266.65-312.65) | 37316 (34745-40073) | 448.81 (420.86-478.15) | 1.47 (1.41-1.54) |
| Kazakhstan | 14602 (13439-15891) | 101.76 (93.51-111.24) | 35051 (31601-38749) | 183.92 (168.89-201.62) | 2.07 (1.87-2.27) |
| Kenya | 10255 (9567-10957) | 92.88 (85.46-101.07) | 31651 (29148-34092) | 118.9 (109.21-129.48) | 0.55 (0.39-0.72) |
| Kiribati | 77 (71-84) | 154.97 (142.78-169.03) | 192 (177-207) | 215.92 (199.81-232.07) | 1.05 (1.02-1.09) |
| Kuwait | 2252 (2070-2441) | 293.05 (271.19-317.02) | 13109 (11785-14632) | 397.04 (363.69-431.75) | 1.1 (1.01-1.2) |
| Kyrgyzstan | 3991 (3710-4283) | 105.47 (97.4-113.97) | 8823 (8020-9622) | 151.01 (137.96-164.46) | 1.18 (1.04-1.33) |
| Lao People's Democratic Republic | 4381 (4022-4785) | 169.68 (155.23-185.94) | 12021 (11060-12971) | 227.71 (209.17-246.18) | 0.96 (0.87-1.06) |
| Latvia | 2877 (2583-3214) | 87.64 (79.51-96.72) | 5423 (4898-6022) | 165.91 (152.48-181.71) | 2.06 (1.92-2.2) |
| Lebanon | 6124 (5556-6781) | 267.39 (245.37-292.65) | 25285 (23327-27213) | 435.3 (397.96-470.82) | 1.59 (1.52-1.65) |
| Lesotho | 1419 (1305-1543) | 149.85 (137.2-163.67) | 2455 (2265-2646) | 201.81 (186.09-216.67) | 0.78 (0.57-1) |
| Liberia | 1959 (1816-2115) | 130.28 (119.69-142.17) | 4919 (4558-5317) | 177.94 (161.69-194.48) | 1.17 (1.09-1.25) |
| Libya | 5633 (5189-6163) | 262.39 (239.49-288.31) | 23278 (21519-25396) | 417.75 (384.87-453.56) | 1.32 (1.2-1.45) |
| Lithuania | 4205 (3814-4676) | 99.04 (90.44-109.62) | 6974 (6366-7568) | 147.64 (137.04-159.15) | 1.36 (1.2-1.53) |
| Luxembourg | 1367 (1246-1511) | 243.76 (223.33-267.27) | 2759 (2529-2961) | 263.07 (240.94-282.99) | 0.37 (0.25-0.48) |
| Madagascar | 5781 (5385-6208) | 86.15 (79.26-93.66) | 15047 (13993-16361) | 107 (97.67-116.36) | 0.65 (0.52-0.78) |
| Malawi | 4890 (4550-5238) | 96.85 (89.11-105.85) | 11239 (10343-11977) | 124.02 (112.77-133.9) | 0.79 (0.66-0.91) |
| Malaysia | 18546 (17026-20013) | 175.49 (159.31-190.28) | 77138 (70879-83439) | 259.98 (240.09-281.17) | 1.3 (1.26-1.35) |
| Maldives | 214 (193-237) | 186.89 (169.21-206.05) | 1059 (952-1155) | 288.32 (259.9-317.9) | 1.51 (1.38-1.63) |
| Mali | 6631 (6181-7134) | 125.11 (115.55-135.77) | 19506 (18189-20764) | 159.68 (147.15-172.12) | 0.79 (0.7-0.87) |
| Malta | 1095 (1000-1202) | 256.24 (235.59-279.7) | 2663 (2436-2926) | 266.7 (246.75-289.77) | 0.17 (0.09-0.25) |
| Marshall Islands | 39 (36-42) | 167.21 (154.19-182.59) | 102 (93-110) | 245.16 (225.91-264.04) | 1.16 (1.11-1.22) |
| Mauritania | 1888 (1754-2046) | 148.64 (136.98-161.33) | 5232 (4833-5667) | 200.89 (183.17-219.1) | 0.98 (0.89-1.08) |
| Mauritius | 2071 (1884-2281) | 254.92 (231.38-279.25) | 7451 (6925-8055) | 406.79 (383.3-433.7) | 1.6 (1.54-1.67) |
| Mexico | 151265 (138996-165600) | 304.82 (277.96-335.41) | 612577 (580429-645104) | 464.33 (440.38-488.1) | 1.48 (1.39-1.57) |
| Micronesia (Federated States of) | 115 (106-126) | 186.54 (171.93-204.53) | 247 (223-269) | 290.81 (268.19-313.56) | 1.43 (1.37-1.5) |
| Monaco | 156 (140-175) | 211.38 (191.51-233.27) | 227 (206-250) | 228.39 (209.34-249.89) | 0.35 (0.31-0.39) |
| Mongolia | 2040 (1864-2228) | 139.64 (125.96-153.16) | 5269 (4830-5728) | 188.33 (171.5-203.8) | 0.91 (0.86-0.97) |
| Montenegro | 1134 (1029-1251) | 181.18 (164.66-199.23) | 2639 (2368-2916) | 274.18 (249.16-301.37) | 1.52 (1.46-1.59) |
| Morocco | 32617 (29938-35664) | 210.12 (191.81-230.84) | 135407 (123555-147157) | 384.26 (352.31-415.8) | 1.95 (1.81-2.09) |
| Mozambique | 6980 (6451-7573) | 93.07 (85.24-102.04) | 17355 (16180-18602) | 118.65 (109.29-129.06) | 0.7 (0.56-0.84) |
| Myanmar | 43845 (39884-48151) | 159.87 (145.82-176.19) | 116368 (106212-125983) | 227.58 (209.28-245.02) | 1.15 (1.03-1.28) |
| Namibia | 1156 (1061-1260) | 153.57 (140.73-167.23) | 2985 (2740-3260) | 198.04 (181.92-217.06) | 0.75 (0.56-0.94) |
| Nauru | 11 (10-12) | 179.92 (165.28-195.96) | 18 (17-20) | 256.09 (234.76-279.13) | 1.09 (1.02-1.17) |
| Nepal | 14842 (13643-16160) | 124.26 (114-136.79) | 54613 (49880-59454) | 213.74 (195.45-232.37) | 1.58 (1.51-1.64) |
| Netherlands | 44064 (40881-47584) | 213.48 (198.88-229.67) | 88460 (81320-95824) | 242.77 (224.71-261.33) | 0.59 (0.53-0.65) |
| New Zealand | 10613 (9606-11747) | 264.73 (242.79-290.53) | 25356 (23124-27531) | 301.67 (277.33-327.31) | 0.47 (0.4-0.53) |
| Nicaragua | 5618 (5153-6112) | 277.67 (252.08-306.69) | 24136 (22516-25614) | 438.24 (408.73-465.27) | 1.52 (1.46-1.59) |
| Niger | 5267 (4896-5655) | 125.05 (115.67-136.13) | 17881 (16629-19193) | 152.09 (138.04-165.17) | 0.66 (0.6-0.72) |
| Nigeria | 76055 (70880-81863) | 135.98 (125.16-148.38) | 210760 (196709-225476) | 181.33 (165.23-196.44) | 0.93 (0.78-1.08) |
| Niue | 4 (4-4) | 182.65 (166.78-200.17) | 6 (5-6) | 277.86 (252.87-305.83) | 1.36 (1.3-1.41) |
| North Macedonia | 2592 (2353-2867) | 135.2 (123.5-147.43) | 8708 (7721-9571) | 260.47 (236.73-282.51) | 2.31 (2.09-2.52) |
| Northern Mariana Islands | 62 (55-68) | 239.87 (216.44-262.37) | 192 (170-217) | 340.32 (312.96-378.11) | 0.97 (0.82-1.11) |
| Norway | 13125 (11763-14781) | 182.58 (166.38-202.55) | 21817 (19979-23671) | 212.31 (195.33-229.09) | 0.61 (0.57-0.65) |
| Oman | 1754 (1614-1896) | 218.4 (197.23-237.94) | 9118 (8291-9915) | 396.6 (366.06-431.67) | 1.89 (1.71-2.07) |
| Pakistan | 98898 (91761-107003) | 142.46 (131.44-155.25) | 275774 (256521-294773) | 181.88 (167.78-195.95) | 0.89 (0.86-0.92) |
| Palau | 24 (21-27) | 213.24 (190.87-238.54) | 82 (74-93) | 348.84 (322.35-385.33) | 1.59 (1.46-1.73) |
| Palestine | 2997 (2749-3268) | 290.3 (264.86-317.37) | 12369 (11405-13455) | 430.73 (398.67-464.21) | 1.17 (1.06-1.27) |
| Panama | 3972 (3641-4354) | 243.65 (221.54-269.13) | 15956 (15016-17069) | 363.79 (341.46-389.29) | 1.4 (1.35-1.45) |
| Papua New Guinea | 2466 (2243-2731) | 97.31 (87.81-108.55) | 8614 (7916-9315) | 128.73 (116.82-139.84) | 0.89 (0.74-1.05) |
| Paraguay | 4761 (4373-5210) | 191.86 (174.61-210.72) | 18724 (17247-20324) | 307.76 (282.75-334.34) | 1.59 (1.53-1.65) |
| Peru | 19680 (18106-21486) | 148.84 (136.14-163.48) | 95333 (88068-103496) | 284.09 (261.38-308.38) | 2.4 (2.3-2.5) |
| Philippines | 66080 (61640-70710) | 184.45 (170.37-199.25) | 245592 (230483-262725) | 271.1 (254.37-289.22) | 1.22 (1.19-1.25) |
| Poland | 63497 (56153-71143) | 148.95 (133.49-165.67) | 137937 (123815-154597) | 201.67 (183.51-223.14) | 0.37 (0.18-0.57) |
| Portugal | 29572 (26316-33103) | 211.28 (190.16-233.78) | 60079 (54791-66350) | 236.41 (215.27-260.2) | 0.5 (0.44-0.56) |
| Puerto Rico | 8257 (7527-9060) | 228.91 (208.57-251.43) | 21077 (19420-22999) | 354.68 (332.4-384.64) | 1.55 (1.36-1.73) |
| Qatar | 463 (413-513) | 294.64 (267.04-326.89) | 6175 (5515-6879) | 467.39 (432.92-503.88) | 1.32 (1.25-1.39) |
| Republic of Korea | 61023 (54616-68362) | 214.95 (193.76-239.29) | 226256 (210509-241377) | 242.84 (225.84-258.55) | 0.5 (0.39-0.62) |
| Republic of Moldova | 2996 (2720-3309) | 69.38 (63.48-76.22) | 6551 (5870-7305) | 123.63 (112.7-136.18) | 1.75 (1.55-1.96) |
| Romania | 30710 (27578-34445) | 113.06 (103.32-125.21) | 72834 (67186-78514) | 210.87 (196.04-225.81) | 2.1 (1.93-2.28) |
| Russian Federation | 201892 (185153-222440) | 121.16 (112.76-131.68) | 422141 (385788-459285) | 201.91 (187.06-217.07) | 1.71 (1.64-1.77) |
| Rwanda | 3596 (3326-3889) | 95.81 (88.13-104.85) | 8666 (7877-9399) | 120.75 (110.03-132.18) | 0.77 (0.66-0.89) |
| Saint Kitts and Nevis | 81 (73-89) | 223.11 (203.62-245.66) | 253 (228-280) | 343.85 (319.49-374.16) | 1.5 (1.34-1.65) |
| Saint Lucia | 190 (176-205) | 200.55 (185.46-216.19) | 736 (682-807) | 314.36 (293.07-343.46) | 1.46 (1.33-1.58) |
| Saint Vincent and the Grenadines | 145 (134-157) | 189.14 (175.46-206.37) | 411 (373-445) | 294.08 (270.42-314.58) | 1.47 (1.37-1.57) |
| Samoa | 196 (182-212) | 190.4 (176.36-206.44) | 433 (402-471) | 269.71 (250.26-292.63) | 1.08 (1.04-1.13) |
| San Marino | 70 (63-79) | 194.97 (175.17-216.38) | 157 (141-174) | 207.35 (186.79-229.43) | 0.27 (0.21-0.32) |
| Sao Tome and Principe | 130 (119-142) | 163.22 (148.02-178.81) | 325 (302-350) | 240.52 (222.6-260.76) | 1.32 (1.25-1.4) |
| Saudi Arabia | 21157 (19638-22839) | 295.83 (272.14-320.38) | 123154 (113311-133632) | 495.83 (465.09-529.64) | 1.58 (1.47-1.69) |
| Senegal | 5896 (5479-6341) | 136.13 (125.93-146.92) | 15909 (14807-17097) | 171.24 (158.95-185.48) | 0.68 (0.65-0.72) |
| Serbia | 15572 (13985-17192) | 134.77 (123.15-146.9) | 39137 (35808-43025) | 246.2 (226.98-268.22) | 2.27 (2.14-2.4) |
| Seychelles | 127 (117-138) | 217.23 (200.1-236.55) | 379 (345-414) | 315.56 (289.33-341.18) | 1.19 (1.08-1.3) |
| Sierra Leone | 3353 (3108-3634) | 126.59 (115.64-139) | 7939 (7427-8505) | 163.15 (149.59-176.27) | 0.81 (0.67-0.96) |
| Singapore | 5595 (5322-5869) | 254.11 (241.44-266.8) | 25251 (23413-27411) | 298.99 (279.18-325.6) | 0.64 (0.57-0.72) |
| Slovakia | 8255 (7427-9098) | 140.95 (128.07-154.9) | 20773 (18945-22792) | 226.55 (207.13-244.61) | 1.54 (1.47-1.62) |
| Slovenia | 3003 (2710-3343) | 125.99 (114.01-139.41) | 9213 (8322-10191) | 220.86 (200.94-243.54) | 1.93 (1.78-2.08) |
| Solomon Islands | 347 (324-372) | 162.81 (150.76-175.88) | 825 (765-887) | 178.99 (165.17-193.51) | 0.1 (-0.02-0.23) |
| Somalia | 3438 (3200-3700) | 96.22 (88.58-104.36) | 10305 (9580-10987) | 113.03 (103.05-122.34) | 0.43 (0.33-0.53) |
| South Africa | 40375 (37410-43475) | 172.86 (160.07-187.5) | 116840 (108463-124634) | 242.59 (225.07-257.95) | 1.03 (0.91-1.16) |
| South Sudan | 3133 (2915-3386) | 97.83 (90.15-106.87) | 5863 (5451-6351) | 120.2 (110.54-131.24) | 0.58 (0.47-0.69) |
| Spain | 132317 (121229-145627) | 237.83 (219.69-258.28) | 235484 (215974-254667) | 232.21 (211.91-250.03) | 0.06 (-0.01-0.14) |
| Sri Lanka | 21972 (20287-23967) | 186.57 (171.8-203.07) | 77127 (70184-84714) | 280.69 (257.94-305.2) | 1.39 (1.34-1.44) |
| Sudan | 22886 (20725-25488) | 216.76 (195.29-241.94) | 72866 (66723-78876) | 334.15 (303.81-366.32) | 1.34 (1.12-1.55) |
| Suriname | 540 (493-591) | 188.36 (171.76-206.62) | 1937 (1753-2093) | 303 (278.56-326.7) | 1.69 (1.61-1.78) |
| Sweden | 28865 (26001-32782) | 180.28 (164.1-201.27) | 38458 (34200-43016) | 171.34 (155.23-188.6) | 0.02 (-0.04-0.08) |
| Switzerland | 25404 (23197-28054) | 237.27 (218.25-259.52) | 47328 (42804-51366) | 256.74 (232.96-278.09) | 0.36 (0.28-0.43) |
| Syrian Arab Republic | 16987 (15686-18588) | 269.1 (247.07-295.78) | 56594 (52294-61422) | 407.4 (380.27-438.93) | 1.31 (1.23-1.39) |
| Taiwan (Province of China) | 41232 (38269-44342) | 253.71 (236.83-271.74) | 123945 (116112-131502) | 302.04 (284.6-319) | 0.56 (0.47-0.65) |
| Tajikistan | 3032 (2708-3348) | 79.38 (70.82-88.37) | 9226 (8357-10093) | 127.73 (116.14-140) | 1.83 (1.61-2.06) |
| Thailand | 83500 (76620-91125) | 209.97 (193.6-228.52) | 317728 (294103-344411) | 301.42 (281.43-325.41) | 1.13 (1.03-1.24) |
| Timor-Leste | 589 (545-639) | 148.86 (136.71-164.16) | 1927 (1773-2103) | 202.45 (186.05-220.15) | 1.09 (0.98-1.2) |
| Togo | 2470 (2302-2654) | 137.25 (126.56-148.73) | 8279 (7641-8954) | 176.54 (160.07-192.24) | 0.72 (0.58-0.85) |
| Tokelau | 2 (2-2) | 156.46 (144.13-170.08) | 4 (3-4) | 248.64 (228.62-269.09) | 1.47 (1.41-1.53) |
| Tonga | 120 (110-131) | 184.57 (169.88-203.59) | 226 (209-243) | 264.39 (244.83-284.9) | 1.11 (1.08-1.15) |
| Trinidad and Tobago | 1663 (1540-1811) | 183.34 (168.93-200.97) | 5778 (5277-6286) | 308.46 (285.17-332.51) | 1.84 (1.75-1.94) |
| Tunisia | 13411 (12220-14805) | 249.46 (228.06-273.32) | 54366 (49514-59189) | 402.84 (370.59-435.32) | 1.48 (1.39-1.56) |
| Türkiye | 3149 (2914-3384) | 115.59 (107.05-125.72) | 8401 (7732-9038) | 179 (165.84-191.12) | 1.59 (1.44-1.74) |
| Turkmenistan | 11 (10-12) | 149.43 (137.38-164.84) | 26 (24-29) | 239.74 (221.71-260.75) | 1.51 (1.47-1.55) |
| Tuvalu | 80831 (75539-86876) | 223.53 (208.16-239.61) | 384231 (352192-414424) | 404.1 (371.72-432.15) | 1.88 (1.8-1.95) |
| Uganda | 7432 (6918-7994) | 86.93 (79.94-94.55) | 19849 (18513-21371) | 106.71 (97.77-116.52) | 0.57 (0.41-0.73) |
| Ukraine | 52974 (47075-59414) | 81.45 (73.62-89.89) | 82042 (75038-90447) | 123.52 (113.87-134.46) | 1.41 (1.31-1.5) |
| United Arab Emirates | 2135 (1941-2336) | 327.34 (300.54-358.32) | 31938 (28288-36332) | 466.42 (432.99-505.58) | 0.99 (0.88-1.09) |
| United Kingdom | 195682 (178100-218323) | 209 (191.31-229.71) | 290434 (265595-316836) | 219.73 (201.61-237.17) | -0.02 (-0.11-0.06) |
| United Republic of Tanzania | 14481 (13439-15616) | 104.14 (95.2-113.18) | 38553 (35790-41463) | 128.12 (117.66-139.12) | 0.58 (0.47-0.68) |
| United States of America | 965462 (882901-1056989) | 303.42 (278.01-331.01) | 1842212 (1695726-1979976) | 323.19 (300.14-346.3) | 0.09 (0-0.18) |
| United States Virgin Islands | 186 (171-202) | 201.39 (186.04-217.73) | 512 (467-565) | 315.13 (291.58-344.04) | 1.52 (1.4-1.64) |
| Uruguay | 7727 (7006-8507) | 197.08 (179.76-215.55) | 13240 (12080-14386) | 238.26 (219.31-257.28) | 0.71 (0.66-0.77) |
| Uzbekistan | 21869 (20280-23618) | 143.91 (133.02-157.29) | 62513 (56559-68430) | 205.69 (188.05-223.65) | 1.2 (1.06-1.34) |
| Vanuatu | 124 (114-132) | 144.67 (133.48-157.38) | 447 (413-485) | 215.16 (198.25-231.45) | 1.21 (1.16-1.27) |
| Venezuela (Bolivarian Republic of) | 27615 (25358-30284) | 246.23 (225.06-272.61) | 113852 (105689-122187) | 371.16 (346.13-396.46) | 1.49 (1.38-1.61) |
| Viet Nam | 61003 (55831-66358) | 136.89 (125.14-149.23) | 215387 (195155-237672) | 214.7 (196.62-234.13) | 1.68 (1.62-1.74) |
| Yemen | 12658 (11432-13948) | 212.97 (191.63-236.63) | 48349 (44356-52264) | 303.73 (277.2-330.97) | 1.15 (0.93-1.36) |
| Zambia | 4313 (4015-4642) | 108.48 (100.19-118.21) | 12225 (11417-13120) | 137.13 (126.39-148.92) | 0.71 (0.55-0.88) |
| Zimbabwe | 7854 (7224-8575) | 158.79 (145.39-175.57) | 15456 (14249-16766) | 189.54 (174.01-205.68) | 0.29 (0.09-0.49) |

**TableS6: Trends in the burden of chronic kidney disease by sex: incidence, prevalence, deaths, and disability-adjusted life years (1990–2021).**

| Sex | 1990 | | 2021 | | **EAPC_95%CI** |
| --- | --- | --- | --- | --- | --- |
|  | Number_95%UI | ASR | Number_95%UI | ASR |  |
| **Incidence** |  |  |  |  |  |
| Global | 7790705 (7226165-8402568) | 192.16 (178.69-207.34) | 19935038 (18702793-21170794) | 233.56 (220.02-247.24) | 0.64 (0.63-0.65) |
| Female | 4405633 (4083071-4760087) | 201.88 (187.73-218.45) | 11128691 (10445862-11806798) | 246.03 (231.84-260.67) | 0.65 (0.64-0.66) |
| Male | 3385072 (3133504-3642480) | 180.89 (167.97-195.41) | 8806346 (8248899-9359371) | 220.11 (207.07-233.07) | 0.65 (0.64-0.66) |
| **Prevalence** |  |  |  |  |  |
| Global | 350962674 (326973785-376155723) | 8072.75 (7560.37-8634.07) | 673722703 (629095119-722364096) | 8006 (7482.12-8575.62) | 0.01 (-0.02-0.04) |
| Female | 188185026 (175770189-201580675) | 8293.08 (7778.75-8870.27) | 358777424 (335684717-383643812) | 8182.65 (7653.14-8764.76) | 0 (-0.04-0.03) |
| Male | 162777648 (151189910-174916268) | 7812.8 (7301.88-8356.28) | 314945280 (293385891-338118606) | 7808.96 (7288.71-8366.6) | 0.03 (0-0.05) |
| **Deaths** |  |  |  |  |  |
| Global | 552673 (513463-607915) | 14.85 (13.64-16.38) | 1527639 (1389377-1638914) | 18.5 (16.72-19.85) | 0.82 (0.76-0.89) |
| Female | 261007 (237127-287997) | 12.64 (11.44-13.99) | 733120 (654830-795633) | 15.9 (14.22-17.27) | 0.83 (0.76-0.9) |
| Male | 291666 (259303-334444) | 18.13 (16.26-21.03) | 794519 (719354-856326) | 21.91 (19.66-23.6) | 0.75 (0.69-0.8) |
| **DALYs** |  |  |  |  |  |
| Global | 20739895 (18843684-22588533) | 479.85 (439.18-523.79) | 44453684 (40840762-48508462) | 529.62 (486.25-577.42) | 20739895 (18843684-22588533) |
| Female | 9670407 (8788280-10592764) | 426.9 (389.12-467.43) | 20693932 (18836614-22728832) | 465.69 (424.02-511.12) | 9670407 (8788280-10592764) |
| Male | 11069488 (9601627-12296840) | 546.41 (476.93-612.62) | 23759752 (21473235-26215374) | 603.4 (546.08-663.35) | 11069488 (9601627-12296840) |

| **TableS7: National Burden of chronic kidney disease: deaths cases, ASDR, and EAPC (1990–2021).** | | | | | |
| --- | --- | --- | --- | --- | --- |
| Country | **1990** | | **2021** | | **EAPC_95%CI** |
|  | Number_95%UI | ASR | Number_95%UI | ASR |  |
| Afghanistan | 3009 (2175-4675) | 47.31 (34.52-75.86) | 4893 (2926-8625) | 53.88 (32.94-102.2) | 0.5 (0.45-0.55) |
| Albania | 234 (196-277) | 11.99 (10.05-14.31) | 424 (322-541) | 10.6 (8.07-13.41) | -0.14 (-0.39-0.11) |
| Algeria | 2344 (1667-4128) | 27.12 (19.03-47.74) | 9785 (7909-12092) | 37.33 (30.61-45.46) | 1.55 (1.33-1.78) |
| American Samoa | 7 (5-10) | 33.53 (24.6-48.13) | 31 (24-39) | 73.81 (57.35-91.7) | 2.75 (2.51-2.99) |
| Andorra | 5 (4-7) | 12.15 (8.81-16.68) | 17 (12-23) | 9.32 (6.85-12.45) | -0.5 (-0.72--0.29) |
| Angola | 1417 (1093-1793) | 37.62 (29.25-46.86) | 4073 (2988-5384) | 39.16 (27.9-50.34) | 0.01 (-0.1-0.12) |
| Antigua and Barbuda | 17 (16-18) | 30.15 (27.88-32.24) | 46 (42-49) | 47.12 (43.05-50.47) | 2.08 (1.83-2.33) |
| Argentina | 9185 (8697-9648) | 30.39 (28.61-31.92) | 15213 (13851-16266) | 26.34 (24.04-28.12) | -0.3 (-0.59--0.01) |
| Armenia | 27 (22-31) | 0.97 (0.82-1.13) | 432 (354-523) | 10.25 (8.4-12.44) | 7.31 (6.07-8.57) |
| Australia | 1580 (1436-1674) | 8.72 (7.87-9.26) | 5011 (4201-5515) | 9.41 (8.01-10.32) | 0.96 (0.68-1.24) |
| Austria | 786 (724-834) | 6.58 (6.07-6.98) | 3411 (2801-3759) | 14.28 (11.9-15.62) | 4.06 (3.46-4.66) |
| Azerbaijan | 357 (277-452) | 6.59 (5.09-8.35) | 992 (717-1295) | 10.4 (7.56-13.38) | 1.69 (1.4-1.97) |
| Bahamas | 37 (34-40) | 23.09 (21.31-25.08) | 144 (117-178) | 37.5 (30.66-46.19) | 2.14 (1.9-2.38) |
| Bahrain | 48 (36-63) | 41.68 (31.28-55.66) | 259 (207-316) | 52.48 (42.08-63.37) | 0.39 (0.01-0.78) |
| Bangladesh | 9553 (6572-11510) | 14.89 (12.84-18.33) | 15175 (11740-20173) | 12.27 (9.51-16.21) | -0.62 (-0.88--0.36) |
| Barbados | 60 (57-64) | 20.4 (19.14-21.43) | 148 (117-182) | 29.32 (23.28-35.97) | 1.7 (1.4-2) |
| Belarus | 100 (93-106) | 0.83 (0.78-0.89) | 360 (296-427) | 2.35 (1.94-2.78) | 3.22 (2.38-4.07) |
| Belgium | 1386 (1260-1478) | 8.81 (7.98-9.39) | 2816 (2274-3150) | 9 (7.46-9.99) | 0.67 (0.43-0.91) |
| Belize | 24 (23-26) | 24.4 (22.82-25.97) | 134 (117-151) | 45.45 (39.52-51.13) | 2.52 (2.05-2.99) |
| Benin | 782 (673-912) | 37.31 (32.31-44.65) | 1940 (1558-2414) | 39.41 (32.46-48.4) | 0.11 (0.01-0.21) |
| Bermuda | 9 (9-10) | 15.49 (14.55-16.51) | 23 (19-27) | 15.65 (13.18-19.01) | 0.5 (0.18-0.81) |
| Bhutan | 42 (30-58) | 16.98 (12.44-24.17) | 120 (81-165) | 20.86 (14.03-28.51) | 0.71 (0.64-0.78) |
| Bolivia (Plurinational State of) | 1325 (1115-1675) | 42.98 (36.19-53.94) | 4731 (3675-6140) | 58.23 (45.72-75.35) | 1.08 (0.99-1.18) |
| Bosnia and Herzegovina | 361 (307-422) | 9.76 (8.26-11.43) | 686 (512-869) | 10.99 (8.27-13.95) | 0.3 (0.16-0.43) |
| Botswana | 109 (79-157) | 23.13 (17.01-32.05) | 348 (270-479) | 28.52 (23.01-39.03) | 0.81 (0.6-1.01) |
| Brazil | 15168 (14505-15699) | 17.98 (16.9-18.76) | 45537 (41271-47987) | 18.7 (16.88-19.75) | 0.18 (0.02-0.33) |
| Brunei Darussalam | 32 (26-42) | 37.51 (30.68-47.76) | 86 (73-100) | 36.33 (30.61-42.27) | 0.54 (0.29-0.8) |
| Bulgaria | 729 (668-785) | 7.63 (6.98-8.17) | 2428 (2049-2872) | 17.38 (14.56-20.82) | 3.54 (3.06-4.02) |
| Burkina Faso | 1555 (1281-1850) | 38.32 (31.8-45.82) | 3812 (3071-4633) | 43.65 (35.29-53.03) | 0.48 (0.4-0.55) |
| Burundi | 909 (711-1148) | 39.49 (31.04-50.64) | 1515 (1109-2172) | 34.97 (25.85-49.93) | -0.77 (-0.92--0.62) |
| C?te d'Ivoire | 38 (32-44) | 15.37 (12.74-17.94) | 124 (84-155) | 28.9 (19.42-35.98) | 1.79 (1.52-2.05) |
| Cabo Verde | 1050 (857-1277) | 21.21 (17.48-25.7) | 2681 (2007-3484) | 23.47 (17.8-30.16) | 0.27 (0.13-0.41) |
| Cambodia | 2151 (1677-2797) | 52 (40-67.11) | 5928 (4179-8334) | 51.19 (37.14-71.11) | -0.26 (-0.37--0.15) |
| Cameroon | 2119 (1921-2236) | 6.77 (6.11-7.17) | 7789 (6588-8473) | 9.48 (8.16-10.26) | 1.47 (1.28-1.66) |
| Canada | 507 (420-615) | 46.99 (38.58-56.38) | 963 (698-1352) | 47.39 (35.38-63.92) | 0.05 (0-0.09) |
| Central African Republic | 751 (593-1013) | 26.39 (20.47-36.89) | 1811 (1330-2568) | 31.6 (23.42-45.57) | 0.45 (0.28-0.61) |
| Chad | 1471 (1390-1543) | 16 (14.99-16.88) | 5202 (4582-5596) | 19.81 (17.48-21.3) | 1.16 (0.77-1.55) |
| Chile | 102726 (89745-120908) | 14.16 (12.44-16.78) | 204230 (164736-246372) | 10.84 (8.77-12.96) | -0.89 (-0.97--0.81) |
| China | 3340 (3177-3471) | 19.52 (18.39-20.41) | 8646 (7184-10116) | 15.48 (12.88-18.12) | -0.51 (-0.66--0.37) |
| Colombia | 70 (54-88) | 38.99 (30.8-47.99) | 182 (132-235) | 42.26 (30.99-54.37) | 0.09 (-0.02-0.19) |
| Comoros | 531 (406-637) | 55.83 (41.04-67.22) | 1252 (847-1626) | 54.79 (35.56-69) | -0.24 (-0.36--0.12) |
| Congo | 2 (2-3) | 17.5 (13.81-23.18) | 5 (4-7) | 22.01 (16.6-27.23) | 0.8 (0.71-0.89) |
| Cook Islands | 264 (247-279) | 15.27 (14.25-16.16) | 1605 (1391-1794) | 28.95 (25.22-32.3) | 2.15 (1.68-2.63) |
| Costa Rica | 502 (464-540) | 9.47 (8.76-10.19) | 1337 (1158-1549) | 13.47 (11.6-15.74) | 0.94 (0.57-1.31) |
| Croatia | 831 (789-866) | 8.31 (7.87-8.67) | 3209 (2758-3638) | 16.14 (13.88-18.31) | 2.7 (2.41-2.99) |
| Cuba | 188 (156-238) | 41.28 (34.14-51.47) | 343 (284-403) | 21.54 (18.09-25.01) | -2.28 (-2.56--2.01) |
| Cyprus | 1096 (999-1200) | 8.18 (7.47-8.9) | 1562 (1326-1820) | 6.8 (5.76-7.99) | -0.46 (-0.66--0.27) |
| Czechia | 1455 (1171-1787) | 38.9 (32.03-47.66) | 4060 (3151-5240) | 40.49 (32.99-51.2) | -0.03 (-0.13-0.08) |
| Democratic People's Republic of Korea | 2021 (1438-2685) | 14.03 (10.07-19.34) | 4298 (3284-5596) | 13.95 (10.65-18.26) | 0.1 (-0.01-0.21) |
| Democratic Republic of the Congo | 5868 (4671-7377) | 42.25 (34.51-51.95) | 13845 (9966-18759) | 42.95 (31.38-57.74) | -0.04 (-0.15-0.07) |
| Denmark | 413 (379-437) | 4.83 (4.45-5.09) | 1699 (1457-1893) | 12.1 (10.45-13.46) | 3.08 (2.86-3.29) |
| Djibouti | 35 (26-47) | 29.35 (22.4-37.73) | 211 (152-285) | 41.82 (31.38-54.67) | 1.14 (1.02-1.26) |
| Dominica | 19 (16-21) | 32.36 (28.23-36.34) | 40 (32-49) | 50.29 (40.46-61.83) | 1.64 (1.56-1.73) |
| Dominican Republic | 621 (533-750) | 16.66 (14.3-20.34) | 2392 (1646-3064) | 23.99 (16.46-30.7) | 1.97 (1.72-2.22) |
| Ecuador | 1227 (1150-1287) | 23.7 (22.11-24.95) | 6502 (4898-8822) | 42.06 (32.21-56.05) | 1.95 (1.02-2.88) |
| Egypt | 10610 (8478-16104) | 51.75 (40.34-82.61) | 33311 (27183-40816) | 71.73 (59.72-86.59) | 1.44 (1.31-1.57) |
| El Salvador | 797 (702-1141) | 25.26 (22.08-36.29) | 4441 (3314-5504) | 69.81 (52.25-86.75) | 3.36 (2.9-3.82) |
| Equatorial Guinea | 79 (62-98) | 43.75 (35.01-53.38) | 255 (156-372) | 54.7 (31.29-76.52) | 1.01 (0.63-1.4) |
| Eritrea | 358 (267-498) | 32.63 (24.67-45.46) | 902 (610-1462) | 38.08 (26.57-60.61) | 0.5 (0.42-0.58) |
| Estonia | 120 (112-127) | 6.39 (5.98-6.77) | 527 (437-605) | 17.03 (14.19-19.73) | 2.57 (1.98-3.15) |
| Eswatini | 100 (79-125) | 38.66 (30.49-48.34) | 274 (174-383) | 53.52 (36.51-71.62) | 1.42 (0.78-2.06) |
| Ethiopia | 12912 (10097-14949) | 69.02 (56.98-79.61) | 16918 (14059-20068) | 42.38 (34.84-50.16) | -2.03 (-2.21--1.86) |
| Fiji | 95 (67-136) | 27.7 (19.63-40.48) | 314 (231-410) | 47.97 (36.39-61.87) | 1.32 (0.98-1.66) |
| Finland | 214 (194-227) | 3.03 (2.74-3.22) | 818 (657-927) | 4.89 (3.99-5.5) | 2.09 (1.88-2.29) |
| France | 6888 (6157-7355) | 7.75 (6.94-8.26) | 15133 (12606-16843) | 7.33 (6.23-8.09) | 0.28 (0.06-0.49) |
| Gabon | 265 (212-318) | 51.1 (40.66-61.64) | 621 (324-822) | 71.55 (35.5-92.96) | 1.04 (0.88-1.2) |
| Gambia | 115 (90-143) | 33.7 (26.83-41.33) | 403 (305-513) | 43.16 (33.15-54.7) | 0.65 (0.54-0.76) |
| Georgia | 197 (161-247) | 3.3 (2.7-4.06) | 713 (574-898) | 12.28 (9.9-15.55) | 4.64 (3.98-5.29) |
| Germany | 11051 (10128-11739) | 8.31 (7.6-8.81) | 39563 (31943-45262) | 15.37 (12.62-17.42) | 3.22 (2.78-3.66) |
| Ghana | 1745 (1343-2490) | 29.52 (22.83-41.83) | 7076 (5563-8854) | 47.67 (37.88-59.27) | 1.84 (1.66-2.02) |
| Greece | 2985 (2744-3174) | 21.33 (19.45-22.71) | 7053 (6077-7641) | 21.35 (18.7-23.01) | -0.64 (-1.67-0.41) |
| Greenland | 3 (3-4) | 13.11 (10.99-15.31) | 8 (6-10) | 14.79 (10.65-18.57) | 0.89 (0.68-1.09) |
| Grenada | 27 (24-29) | 35.72 (32.47-39.23) | 62 (54-69) | 58.28 (51.02-64.92) | 1.99 (1.75-2.23) |
| Guam | 14 (12-17) | 22.93 (20.02-28.29) | 51 (42-58) | 24.05 (20.39-27.2) | 1.13 (0.71-1.55) |
| Guatemala | 1108 (1063-1158) | 33.69 (31.98-35.31) | 5448 (4533-6387) | 50.65 (42.25-59.27) | 2.13 (1.71-2.55) |
| Guinea | 1055 (850-1342) | 32.32 (25.52-41.77) | 1874 (1414-2582) | 34.51 (25.87-47.82) | 0.24 (0.19-0.29) |
| Guinea-Bissau | 201 (157-246) | 50.12 (40.29-60.74) | 316 (246-404) | 46.37 (36.98-58.88) | -0.31 (-0.35--0.27) |
| Guyana | 117 (106-128) | 30.52 (27.65-33.44) | 349 (269-446) | 56.53 (43.78-71.21) | 3.02 (2.66-3.38) |
| Haiti | 962 (707-1555) | 28.7 (20.53-48.93) | 2177 (1207-4587) | 30.89 (17.21-64.76) | 0.47 (0.4-0.55) |
| Honduras | 259 (215-328) | 12.32 (10.08-15.98) | 1439 (1077-1889) | 24.58 (18.76-31.69) | 2.49 (2.24-2.74) |
| Hungary | 842 (795-888) | 6.26 (5.91-6.59) | 2173 (1852-2483) | 10.14 (8.66-11.63) | 2.52 (2.23-2.8) |
| Iceland | 11 (10-12) | 3.59 (3.23-3.88) | 41 (33-46) | 5.79 (4.71-6.47) | 2.06 (1.89-2.22) |
| India | 58475 (50679-67536) | 13.1 (11.18-15.16) | 175637 (146072-208715) | 15.85 (13.14-18.73) | 0.52 (0.35-0.69) |
| Indonesia | 18490 (15846-22280) | 17.33 (14.88-21.77) | 51165 (41553-63408) | 22.98 (18.83-29.04) | 0.98 (0.88-1.09) |
| Iran (Islamic Republic of) | 3718 (2816-5017) | 17.81 (12.99-24.73) | 13627 (11262-15175) | 20.23 (16.49-22.59) | 0.53 (0.42-0.64) |
| Iraq | 2948 (2325-3775) | 36.71 (28.74-47.56) | 8279 (5635-10644) | 43.43 (30.46-54.84) | 0.21 (0.02-0.4) |
| Ireland | 311 (288-330) | 8.2 (7.54-8.69) | 703 (587-792) | 8.18 (6.85-9.2) | 0.68 (0.48-0.89) |
| Israel | 970 (893-1037) | 22.19 (20.17-23.75) | 2790 (2314-3075) | 19.69 (16.5-21.68) | 0.28 (-0.24-0.8) |
| Italy | 7528 (6774-7922) | 8.76 (7.81-9.24) | 19086 (15314-21515) | 9.29 (7.62-10.48) | 0.21 (0.03-0.39) |
| Jamaica | 434 (408-457) | 23.34 (21.9-24.6) | 935 (707-1205) | 29.15 (22.02-37.49) | 0.17 (-0.44-0.78) |
| Japan | 17679 (16010-18557) | 11.61 (10.35-12.26) | 52717 (41038-59205) | 9.48 (7.78-10.42) | -0.82 (-0.92--0.73) |
| Jordan | 360 (293-470) | 31.64 (25.56-41.46) | 1876 (1492-2406) | 33.14 (26.22-41.85) | -0.01 (-0.28-0.26) |
| Kazakhstan | 737 (644-832) | 5.33 (4.65-6.05) | 1982 (1631-2335) | 12.22 (10.08-14.43) | 2.26 (1.9-2.62) |
| Kenya | 1950 (1548-3039) | 25.18 (19.62-40.84) | 7330 (5884-9860) | 37.56 (29.77-49.48) | 1.52 (1.45-1.6) |
| Kiribati | 11 (9-13) | 29.96 (24.18-36.76) | 29 (18-42) | 43.1 (28-62.31) | 0.93 (0.62-1.23) |
| Kuwait | 160 (144-178) | 30.68 (26.6-34.35) | 374 (276-459) | 15.98 (11.7-19.74) | -2.11 (-2.35--1.86) |
| Kyrgyzstan | 159 (141-180) | 4.57 (4.04-5.18) | 498 (415-596) | 9.98 (8.34-11.9) | 1.16 (0.35-1.98) |
| Lao People's Democratic Republic | 917 (677-1191) | 43.94 (33.2-56.93) | 1775 (1242-2408) | 40.89 (29.08-54.72) | -0.35 (-0.4--0.3) |
| Latvia | 95 (89-101) | 2.9 (2.72-3.07) | 321 (265-392) | 7.74 (6.36-9.54) | 3.01 (2.57-3.46) |
| Lebanon | 592 (455-790) | 32.26 (24.68-42.72) | 2102 (1733-2534) | 31.87 (26.28-38.54) | 0.16 (0.04-0.28) |
| Lesotho | 153 (119-202) | 19.86 (15.35-26.5) | 445 (311-595) | 46.19 (32.76-59.42) | 3.68 (3.21-4.16) |
| Liberia | 533 (427-673) | 46.42 (38.02-57.83) | 954 (706-1263) | 48.42 (36.28-63.33) | 0.36 (0.09-0.64) |
| Libya | 503 (374-704) | 28.09 (20.54-40.14) | 2127 (1413-2796) | 46.94 (31.29-61.08) | 2.42 (2.18-2.66) |
| Lithuania | 104 (97-109) | 2.46 (2.31-2.58) | 332 (281-400) | 5.79 (4.87-7.07) | 1.61 (1.17-2.05) |
| Luxembourg | 52 (49-55) | 10.28 (9.5-10.87) | 158 (130-184) | 12.6 (10.53-14.5) | 1.06 (0.84-1.29) |
| Madagascar | 1300 (1031-1793) | 26.7 (20.97-38.18) | 2660 (1941-3524) | 26.98 (19.68-35.45) | 0.08 (-0.02-0.17) |
| Malawi | 1438 (1170-1736) | 38.69 (31.24-48.98) | 3141 (2543-3818) | 45.45 (37.84-54.52) | 0.37 (0.17-0.56) |
| Malaysia | 2075 (1795-2376) | 22.56 (19.43-25.89) | 7575 (6352-8581) | 29.16 (24.39-32.96) | 0.62 (0.4-0.85) |
| Maldives | 40 (34-51) | 48.4 (39.66-69.29) | 88 (71-105) | 28.89 (23.43-34.46) | -1.93 (-2.11--1.74) |
| Mali | 1405 (1153-1734) | 38.3 (31.27-47.75) | 3001 (2407-3922) | 37.4 (30.33-48.05) | 0.04 (-0.09-0.17) |
| Malta | 46 (42-49) | 12.09 (10.91-12.89) | 141 (116-160) | 12.47 (10.44-14.1) | 0.21 (0-0.42) |
| Marshall Islands | 5 (3-9) | 32.53 (20.19-58.7) | 17 (6-49) | 54.79 (17.42-160.83) | 1.68 (1.45-1.9) |
| Mauritania | 394 (305-502) | 42.58 (33.22-54.27) | 774 (532-1095) | 40.37 (27.46-56.45) | -0.49 (-0.67--0.31) |
| Mauritius | 302 (288-315) | 44.32 (42.02-46.39) | 1414 (1304-1494) | 80.13 (74.12-84.55) | 2.12 (1.73-2.5) |
| Mexico | 14133 (13701-14565) | 36.94 (35.44-38.37) | 67044 (58757-76983) | 54.32 (47.78-61.89) | 1.86 (1.24-2.48) |
| Micronesia (Federated States of) | 16 (11-25) | 33.55 (22.78-54.5) | 38 (27-53) | 57.6 (42.14-80.91) | 1.71 (1.31-2.12) |
| Monaco | 5 (4-6) | 6.05 (4.64-7.41) | 12 (9-14) | 9.56 (7.61-11.38) | 1.69 (1.42-1.97) |
| Mongolia | 173 (139-213) | 15.37 (12.4-18.87) | 296 (236-365) | 13.61 (10.77-16.75) | -0.77 (-0.92--0.63) |
| Montenegro | 76 (61-93) | 12.91 (10.38-15.77) | 165 (124-208) | 19.07 (14.04-23.87) | 1.42 (1.21-1.63) |
| Morocco | 3070 (2131-5653) | 23.11 (15.7-45.57) | 11890 (8911-15306) | 39.98 (30.25-51.29) | 2.05 (1.89-2.2) |
| Mozambique | 1433 (1145-1942) | 26.02 (20.77-37.37) | 3627 (2744-4922) | 35.66 (26.92-48.79) | 1.46 (1.32-1.61) |
| Myanmar | 6501 (4977-8336) | 26.56 (20.56-34.38) | 11244 (9001-14131) | 24.94 (20.3-31.09) | -0.46 (-0.55--0.37) |
| Namibia | 119 (92-169) | 21.79 (16.74-30.97) | 313 (231-422) | 26.65 (20.29-35.15) | 0.47 (0.17-0.77) |
| Nauru | 2 (1-3) | 41.07 (26.11-68.81) | 3 (2-5) | 62.96 (42.86-105.35) | 1.23 (1.1-1.35) |
| Nepal | 1249 (960-1663) | 13.47 (10.41-18.13) | 4048 (2947-5424) | 19.19 (14.08-25.14) | 1.4 (1-1.79) |
| Netherlands | 1325 (1179-1417) | 6.65 (5.89-7.11) | 4435 (3715-4925) | 10.89 (9.18-12.06) | 1.69 (1.31-2.07) |
| New Zealand | 271 (249-288) | 7.25 (6.63-7.71) | 957 (835-1041) | 10.67 (9.41-11.56) | 1.48 (1.09-1.87) |
| Nicaragua | 492 (444-663) | 29.38 (26.2-40.14) | 2686 (2148-3230) | 55.35 (44.71-66.47) | 2.62 (2.2-3.03) |
| Niger | 874 (706-1119) | 29.49 (23.91-38.74) | 2007 (1440-3038) | 26.96 (19.88-40.06) | -0.32 (-0.41--0.24) |
| Nigeria | 12895 (10282-15658) | 30.97 (25.14-37.63) | 25429 (19318-32599) | 31.27 (24.98-38.12) | -0.09 (-0.15--0.04) |
| Niue | 1 (1-1) | 29.88 (23.07-40.27) | 1 (1-2) | 56.11 (33.12-86.18) | 1.89 (1.73-2.06) |
| North Macedonia | 173 (145-208) | 10.08 (8.49-12.16) | 370 (282-484) | 13.59 (10.66-17.26) | 0.86 (0.48-1.25) |
| Northern Mariana Islands | 6 (5-8) | 37.25 (30.15-47.45) | 25 (20-30) | 57.62 (47.32-68.42) | 1.54 (1.37-1.71) |
| Norway | 268 (241-282) | 3.53 (3.18-3.7) | 805 (673-878) | 6.48 (5.5-7.02) | 2.15 (1.82-2.48) |
| Oman | 140 (102-207) | 22.91 (16.58-33.81) | 616 (490-771) | 41.71 (33.02-50.37) | 2.63 (2.31-2.96) |
| Pakistan | 10395 (8266-13167) | 18.75 (14.93-23.71) | 31063 (24330-39844) | 26.59 (21.4-33.9) | 0.92 (0.69-1.15) |
| Palau | 3 (2-4) | 29.53 (21.14-39.31) | 9 (6-12) | 50.19 (36.24-66.32) | 1.9 (1.7-2.1) |
| Palestine | 348 (260-460) | 45.37 (34.16-61.6) | 838 (711-1010) | 43.22 (36.65-51.88) | -0.19 (-0.44-0.07) |
| Panama | 201 (188-215) | 13.5 (12.58-14.47) | 1381 (1090-1653) | 30.72 (24.32-36.83) | 2.96 (2.49-3.44) |
| Papua New Guinea | 220 (145-312) | 11.28 (7.85-15.91) | 728 (561-945) | 13.62 (10.35-19.02) | 0.59 (0.54-0.63) |
| Paraguay | 355 (301-418) | 16.14 (13.59-19.07) | 1389 (1036-1761) | 24.92 (18.68-31.54) | 1.58 (1.45-1.7) |
| Peru | 3242 (2796-3748) | 27 (23.29-31.43) | 10386 (7395-13295) | 30.94 (22.09-39.66) | 0.18 (-0.03-0.4) |
| Philippines | 8626 (7839-9922) | 31.72 (28.86-35.59) | 32883 (27438-38113) | 41.71 (35.01-48.11) | 1.37 (1.22-1.51) |
| Poland | 5309 (5123-5460) | 12.85 (12.33-13.23) | 4981 (4384-5654) | 6.61 (5.81-7.57) | -2.64 (-3.15--2.13) |
| Portugal | 1597 (1486-1687) | 13.03 (12.04-13.78) | 4736 (3954-5301) | 14.51 (12.28-16.23) | 0.38 (-0.07-0.84) |
| Puerto Rico | 1091 (1031-1148) | 31.76 (29.9-33.44) | 2537 (2093-2995) | 32.14 (26.55-37.93) | 0.71 (0.29-1.13) |
| Qatar | 28 (16-43) | 40.71 (22.74-65.96) | 198 (153-254) | 39.89 (32.38-49.22) | -0.57 (-1.15-0.02) |
| Republic of Korea | 4257 (3775-4740) | 18.61 (16.33-21.39) | 9823 (7902-11473) | 10.84 (8.73-12.65) | -1.56 (-1.76--1.35) |
| Republic of Moldova | 83 (79-87) | 2.02 (1.93-2.12) | 206 (179-242) | 3.61 (3.15-4.25) | 1.2 (0.76-1.64) |
| Romania | 2551 (2408-2682) | 10.42 (9.84-10.94) | 3325 (2869-3831) | 8.62 (7.42-10.04) | -0.3 (-0.82-0.22) |
| Russian Federation | 8804 (8612-8979) | 5.14 (5.03-5.25) | 14471 (13013-16149) | 6.18 (5.56-6.89) | -0.14 (-0.6-0.31) |
| Rwanda | 1301 (1059-1534) | 47.93 (39.12-56.99) | 2068 (1481-2765) | 38.67 (28-51.22) | -1.52 (-1.86--1.19) |
| Saint Kitts and Nevis | 16 (15-17) | 43.57 (40.54-46.8) | 34 (28-40) | 57.57 (48.51-65.85) | 1.8 (1.49-2.1) |
| Saint Lucia | 30 (29-32) | 37.32 (35.33-39.16) | 93 (77-111) | 40.31 (33.5-47.93) | 0.52 (0.24-0.8) |
| Saint Vincent and the Grenadines | 19 (17-20) | 26.9 (24.86-28.68) | 54 (48-61) | 41.06 (36.19-46.44) | 2.01 (1.69-2.32) |
| Samoa | 24 (17-33) | 31 (22.2-41.05) | 58 (44-75) | 43.46 (33.04-55.51) | 0.99 (0.87-1.11) |
| San Marino | 2 (2-2) | 5.46 (4.53-6.57) | 4 (3-6) | 3.89 (2.64-5.26) | 0.18 (-0.24-0.61) |
| Sao Tome and Principe | 34 (28-38) | 53.67 (45.26-60.79) | 69 (50-85) | 72.37 (51.38-86.92) | 1.05 (0.97-1.12) |
| Saudi Arabia | 2408 (1798-3342) | 45.07 (33.47-64.13) | 12226 (9096-15498) | 79.26 (59.76-95.73) | 1.66 (1.5-1.83) |
| Senegal | 1300 (1084-1593) | 40.62 (34.05-50.3) | 3035 (2315-4175) | 43.53 (33.56-59.16) | 0.14 (0.06-0.22) |
| Serbia | 1464 (1214-1828) | 16.49 (13.61-20.76) | 2713 (2196-3252) | 15.88 (12.83-19.07) | -0.1 (-0.21-0.01) |
| Seychelles | 17 (15-19) | 29.29 (25.52-33.44) | 50 (40-58) | 47.22 (37.91-55.38) | 1.71 (1.45-1.96) |
| Sierra Leone | 628 (495-784) | 30.08 (23.77-37.16) | 1048 (802-1392) | 28.95 (22.59-37.45) | -0.12 (-0.15--0.08) |
| Singapore | 264 (250-275) | 13.74 (12.83-14.43) | 833 (718-926) | 10.18 (8.73-11.34) | -0.39 (-0.94-0.16) |
| Slovakia | 617 (530-708) | 10.59 (9.14-12.2) | 831 (677-1002) | 8.9 (7.25-10.74) | -0.5 (-0.56--0.45) |
| Slovenia | 152 (142-163) | 6.37 (5.92-6.88) | 327 (261-415) | 6.1 (4.88-7.81) | 0.51 (0.23-0.79) |
| Solomon Islands | 31 (13-50) | 22.14 (11.64-34.59) | 90 (69-116) | 25.03 (20.06-31.45) | 0.36 (0.15-0.56) |
| Somalia | 1051 (755-1454) | 45.7 (33.39-63.19) | 2791 (1917-4130) | 47.94 (33.14-68.33) | 0.3 (0.21-0.38) |
| South Africa | 4020 (3508-4993) | 19.75 (16.85-25.08) | 13502 (12124-14916) | 33.24 (29.6-36.64) | 1.78 (1.42-2.14) |
| South Sudan | 1009 (753-1391) | 40.95 (31.05-56.24) | 1949 (1428-2539) | 54.98 (39.86-71.17) | 0.88 (0.7-1.06) |
| Spain | 6907 (6235-7340) | 13.3 (11.93-14.14) | 14802 (11854-16621) | 10.49 (8.59-11.76) | -0.46 (-0.57--0.35) |
| Sri Lanka | 2614 (2219-3080) | 27.67 (23.33-32.77) | 5243 (3567-7155) | 21.22 (14.56-28.53) | -0.85 (-1.02--0.68) |
| Sudan | 2043 (1462-3607) | 22.73 (15.89-43.49) | 5602 (4127-7495) | 32.06 (24.26-43.82) | 1.23 (1.04-1.41) |
| Suriname | 71 (61-81) | 27.68 (24.19-31.82) | 253 (189-329) | 41.4 (31.04-53.55) | 1.67 (1.47-1.88) |
| Sweden | 636 (578-678) | 3.82 (3.48-4.07) | 2518 (2087-2834) | 8.85 (7.43-9.92) | 3.25 (3.04-3.45) |
| Switzerland | 808 (722-860) | 7.15 (6.43-7.61) | 2599 (2042-2973) | 10.21 (8.14-11.71) | 2.02 (1.74-2.29) |
| Syrian Arab Republic | 1951 (1524-2563) | 39.11 (30.41-52.82) | 4836 (3387-6504) | 47.74 (33.59-62.79) | 0.32 (0.1-0.54) |
| Taiwan (Province of China) | 2954 (2802-3083) | 24.13 (22.45-25.37) | 8814 (7593-9771) | 20 (17.37-22.09) | -0.2 (-0.53-0.12) |
| Tajikistan | 59 (48-83) | 1.76 (1.42-2.56) | 152 (106-220) | 2.37 (1.68-3.26) | 0.37 (-0.01-0.75) |
| Thailand | 7941 (6624-9995) | 24.26 (20.24-30.58) | 31734 (24197-39501) | 29.9 (22.77-37.14) | 0.19 (-0.03-0.4) |
| Timor-Leste | 76 (57-101) | 25.64 (19.69-35.58) | 238 (172-335) | 29.76 (21.78-41.42) | 0.68 (0.46-0.9) |
| Togo | 381 (314-467) | 30.96 (26.13-38) | 1136 (854-1520) | 34.53 (26.7-45.18) | 0.22 (0.14-0.3) |
| Tokelau | 0 (0-1) | 24.56 (17.15-42.52) | 1 (0-1) | 37.26 (27.02-54.74) | 1.28 (1.17-1.39) |
| Tonga | 7 (5-10) | 13.38 (9.39-18.57) | 16 (11-21) | 20.55 (14.34-27.02) | 1.26 (1.01-1.5) |
| Trinidad and Tobago | 192 (184-201) | 24.7 (23.52-25.82) | 755 (564-967) | 40.17 (30.11-51.33) | 2.24 (1.89-2.6) |
| Tunisia | 926 (706-1392) | 22.22 (16.99-33.34) | 3765 (2674-5162) | 32.12 (23.11-43.87) | 1.22 (1.16-1.28) |
| Türkiye | 209 (191-228) | 8.94 (8.12-9.83) | 744 (574-947) | 17.99 (13.95-22.74) | 1.88 (1.34-2.43) |
| Turkmenistan | 2 (1-3) | 27.78 (20.77-43.65) | 4 (3-6) | 40.69 (29.68-58.88) | 1.31 (1.21-1.41) |
| Tuvalu | 9286 (7515-12586) | 30.59 (24.46-42.54) | 24805 (19764-30524) | 29.53 (23.34-36.39) | 0.34 (-0.16-0.84) |
| Uganda | 1868 (1404-2443) | 30.99 (23.46-40.74) | 5279 (4093-6903) | 38.83 (30.81-50.76) | 0.45 (0.29-0.6) |
| Ukraine | 84 (80-88) | 0.13 (0.13-0.14) | 1605 (1204-2049) | 2.4 (1.79-3.07) | 13.07 (11.09-15.07) |
| United Arab Emirates | 83 (57-119) | 23.06 (16.05-32.58) | 672 (427-914) | 37.54 (20.93-49.19) | 3.94 (3.18-4.71) |
| United Kingdom | 4239 (3908-4410) | 4.6 (4.23-4.79) | 9679 (8207-10541) | 6.25 (5.36-6.79) | 1.92 (1.57-2.27) |
| United Republic of Tanzania | 3665 (3049-4635) | 36.08 (30.25-46.22) | 8860 (7121-11155) | 37.65 (30.14-46.93) | 0.11 (0.06-0.16) |
| United States of America | 27826 (25245-29210) | 8.49 (7.72-8.9) | 135880 (118167-146606) | 21.93 (19.31-23.51) | 3.56 (3.36-3.76) |
| United States Virgin Islands | 15 (12-19) | 19.89 (16.55-23.98) | 33 (23-43) | 20.47 (14.73-26.56) | 0.5 (0.21-0.78) |
| Uruguay | 598 (561-628) | 15.5 (14.5-16.23) | 1167 (1034-1270) | 17.91 (16.11-19.42) | 0.58 (0.36-0.81) |
| Uzbekistan | 617 (441-920) | 5.05 (3.43-7.88) | 3589 (3013-4204) | 14.24 (12.06-16.66) | 2.31 (1.49-3.13) |
| Vanuatu | 13 (8-22) | 22.02 (14.27-38.66) | 55 (40-85) | 34.34 (24.7-52.56) | 1.46 (1.4-1.53) |
| Venezuela (Bolivarian Republic of) | 1587 (1501-1651) | 16.32 (15.23-17.09) | 11753 (8833-14984) | 40.52 (30.44-51.29) | 2.65 (2.21-3.1) |
| Viet Nam | 9150 (6657-11695) | 23.45 (17.13-30.09) | 23706 (16677-29759) | 27.03 (19.07-33.4) | 0.61 (0.52-0.71) |
| Yemen | 871 (580-1629) | 19.15 (12.74-38.89) | 2471 (1678-4179) | 20.63 (14.22-34.62) | 0.19 (0.08-0.31) |
| Zambia | 1293 (1071-1518) | 46.31 (39.52-54.33) | 3432 (2424-4962) | 52.24 (39.21-69.77) | 0.09 (-0.06-0.25) |
| Zimbabwe | 874 (693-1148) | 26 (20.48-34.24) | 2483 (1893-3317) | 41.43 (31.84-53.59) | 1.94 (1.46-2.41) |

| **TableS8: National Burden of chronic kidney disease: DALYs cases, ASDAR, and EAPC (1990–2021).** | | | | | |
| --- | --- | --- | --- | --- | --- |
| Country | **1990** | | **2021** | | **EAPC_95%CI** |
|  | Number_95%UI | ASR | Number_95%UI | ASR |  |
| Afghanistan | 94773 (70923-137292) | 1274.29 (955.98-1918.62) | 173983 (108001-286182) | 1343.24 (830.87-2358.01) | 94773 (70923-137292) |
| Albania | 9416 (8198-10999) | 378.25 (328.57-441.42) | 11183 (9088-13561) | 303.95 (249.15-366.31) | 9416 (8198-10999) |
| Algeria | 79125 (60711-119848) | 593.89 (442.44-962.72) | 241373 (197533-295747) | 724.15 (595.94-868.77) | 79125 (60711-119848) |
| American Samoa | 260 (201-353) | 953.16 (740.75-1294.18) | 938 (740-1163) | 1950.94 (1545.88-2408.41) | 260 (201-353) |
| Andorra | 148 (115-187) | 286.89 (224.78-360.92) | 357 (285-435) | 229.21 (181.74-279.93) | 148 (115-187) |
| Angola | 60560 (47304-74043) | 1068.56 (834.87-1326.96) | 158051 (120480-207619) | 1011.81 (761.71-1314.95) | 60560 (47304-74043) |
| Antigua and Barbuda | 436 (405-466) | 807.8 (751.92-862.86) | 1162 (1070-1242) | 1116.49 (1028.08-1190.11) | 436 (405-466) |
| Argentina | 223942 (212662-235443) | 705.33 (669.33-740.3) | 315536 (295283-335059) | 573.23 (537.71-608.51) | 223942 (212662-235443) |
| Armenia | 4757 (3535-5893) | 168.21 (126.18-208.59) | 15239 (12675-18395) | 380.18 (316.18-457.36) | 4757 (3535-5893) |
| Australia | 38617 (35222-42200) | 211.2 (193.05-230.14) | 93074 (82639-102916) | 207.14 (183.49-229.01) | 38617 (35222-42200) |
| Austria | 23090 (20228-25966) | 213.38 (187-238.48) | 56356 (49530-62593) | 288.2 (253.46-323.87) | 23090 (20228-25966) |
| Azerbaijan | 22175 (18576-26606) | 374.09 (313.47-447.81) | 47430 (37765-57959) | 451.9 (364.79-546.71) | 22175 (18576-26606) |
| Bahamas | 1314 (1209-1413) | 710.66 (653.57-766.6) | 4315 (3520-5314) | 1035.59 (848.99-1270.93) | 1314 (1209-1413) |
| Bahrain | 1611 (1272-2077) | 856.94 (670.32-1110.27) | 7698 (6382-9087) | 963.54 (793.27-1153.68) | 1611 (1272-2077) |
| Bangladesh | 551460 (306452-697970) | 604.3 (447.85-701.97) | 577364 (481335-715354) | 407.97 (343.85-505.99) | 551460 (306452-697970) |
| Barbados | 1555 (1455-1646) | 564.83 (528.52-598.06) | 3422 (2743-4181) | 732.57 (589.23-902.98) | 1555 (1455-1646) |
| Belarus | 13038 (10184-15922) | 109.57 (86.12-132.79) | 21603 (17974-26090) | 149.78 (125.81-179.79) | 13038 (10184-15922) |
| Belgium | 36155 (31529-40640) | 248.07 (215.35-279.24) | 55840 (47926-63208) | 234.03 (201.28-266.85) | 36155 (31529-40640) |
| Belize | 848 (796-903) | 718.79 (674.47-763.42) | 4198 (3725-4677) | 1236.14 (1098.78-1377.85) | 848 (796-903) |
| Benin | 29653 (25048-34516) | 999.34 (868.44-1155.68) | 71721 (57427-87409) | 990.1 (808.63-1210.26) | 29653 (25048-34516) |
| Bermuda | 264 (244-284) | 421.43 (389.52-452.83) | 482 (414-570) | 395.45 (339.73-465.48) | 264 (244-284) |
| Bhutan | 1915 (1398-2531) | 571.18 (432.97-749.01) | 3865 (2821-5112) | 600.1 (438.76-786.09) | 1915 (1398-2531) |
| Bolivia (Plurinational State of) | 45375 (38779-56751) | 1123.66 (953.42-1408.46) | 121335 (92262-157620) | 1307.42 (1008.75-1688.68) | 45375 (38779-56751) |
| Bosnia and Herzegovina | 13424 (11787-15111) | 326.86 (287.86-366.2) | 17500 (14014-21236) | 311.17 (253.16-378.48) | 13424 (11787-15111) |
| Botswana | 4159 (3143-5808) | 645.59 (492.96-883.78) | 11766 (9282-16126) | 734.52 (596.11-978.22) | 4159 (3143-5808) |
| Brazil | 582735 (547944-618485) | 565.18 (529.7-600.78) | 1273112 (1182007-1361964) | 513.85 (476.98-550.1) | 582735 (547944-618485) |
| Brunei Darussalam | 1038 (881-1248) | 843.62 (710.86-1036.17) | 2607 (2282-2940) | 776.58 (676.45-884.25) | 1038 (881-1248) |
| Bulgaria | 27816 (24930-30650) | 274.08 (246.81-301.38) | 59631 (50184-71490) | 487.91 (408.34-590.67) | 27816 (24930-30650) |
| Burkina Faso | 60272 (50415-71118) | 1014.18 (852.47-1190.59) | 140312 (112697-173625) | 1094.71 (897.51-1322.28) | 60272 (50415-71118) |
| Burundi | 31943 (25440-39942) | 1032.58 (815.32-1304.68) | 52752 (39451-75471) | 837.29 (623.89-1184.66) | 31943 (25440-39942) |
| C?te d'Ivoire | 1204 (1015-1391) | 462.89 (388.99-534.49) | 3178 (2305-3934) | 677.38 (486.86-834.96) | 1204 (1015-1391) |
| Cabo Verde | 48373 (39570-58172) | 757.37 (633.33-895.87) | 101003 (77788-132516) | 733.58 (574.41-943.93) | 48373 (39570-58172) |
| Cambodia | 80907 (63460-102530) | 1348.1 (1082.99-1721.87) | 224422 (163790-306794) | 1319.97 (973-1795.06) | 80907 (63460-102530) |
| Cameroon | 55398 (49172-61889) | 178.37 (158.92-198.89) | 150955 (134749-165748) | 224.7 (200.23-246.05) | 55398 (49172-61889) |
| Canada | 20433 (16924-24299) | 1327.27 (1105.97-1592.63) | 39107 (28354-53349) | 1303.33 (972.36-1758.22) | 20433 (16924-24299) |
| Central African Republic | 28291 (22969-36811) | 736.77 (598.1-968.73) | 73527 (56687-98420) | 840.46 (646.72-1175.37) | 28291 (22969-36811) |
| Chad | 43420 (40600-46183) | 413.69 (384.78-440.39) | 104137 (95786-111766) | 415.15 (383.43-445.69) | 43420 (40600-46183) |
| Chile | 4195631 (3673738-4813077) | 457.69 (402.14-526.92) | 6127923 (5184384-7208227) | 315.33 (266.58-371.53) | 4195631 (3673738-4813077) |
| China | 122275 (114363-131312) | 573.83 (533.11-613.66) | 233106 (198873-269966) | 424.45 (362.45-491.3) | 122275 (114363-131312) |
| Colombia | 2494 (1867-3109) | 984.8 (763.59-1217.01) | 5242 (3955-6654) | 990.68 (748.15-1260.42) | 2494 (1867-3109) |
| Comoros | 19457 (15403-23362) | 1483.45 (1157.43-1769.47) | 44198 (31384-57320) | 1365.57 (964.25-1712.34) | 19457 (15403-23362) |
| Congo | 71 (59-89) | 535.04 (446.1-669.07) | 151 (120-180) | 631.41 (503.79-752.99) | 71 (59-89) |
| Cook Islands | 9157 (8313-10118) | 468.52 (425.52-515.95) | 41809 (37382-46132) | 764.45 (683.83-842.99) | 9157 (8313-10118) |
| Costa Rica | 15608 (14199-16944) | 284.15 (259.01-307.03) | 26558 (22898-31103) | 308.78 (265.45-364.88) | 15608 (14199-16944) |
| Croatia | 29762 (27495-32222) | 285.64 (263.5-308.72) | 79000 (69453-88900) | 436.64 (383.77-494.15) | 29762 (27495-32222) |
| Cuba | 3922 (3338-4773) | 660.43 (562.01-797.89) | 6772 (5876-7764) | 377.75 (329.25-432.39) | 3922 (3338-4773) |
| Cyprus | 35113 (31942-38431) | 277.03 (253-303.17) | 39158 (33376-45630) | 197.69 (167.8-233.11) | 35113 (31942-38431) |
| Czechia | 62444 (50103-77072) | 1012.18 (827.29-1226.77) | 153321 (116483-194558) | 1019.68 (809.33-1277.16) | 62444 (50103-77072) |
| Democratic People's Republic of Korea | 83478 (63914-105819) | 483.43 (373.12-605.87) | 153167 (124247-191899) | 479.21 (390.56-602.59) | 83478 (63914-105819) |
| Democratic Republic of the Congo | 238276 (194665-290653) | 1138.92 (935.7-1393.94) | 513717 (387253-677593) | 1112.28 (844.16-1452.76) | 238276 (194665-290653) |
| Denmark | 13497 (11412-15540) | 177.56 (150.59-205.12) | 31945 (28185-35763) | 266.66 (235.02-300.93) | 13497 (11412-15540) |
| Djibouti | 1395 (1048-1830) | 727.5 (561.13-939.78) | 6952 (5050-9547) | 959.05 (723.5-1275.45) | 1395 (1048-1830) |
| Dominica | 502 (447-555) | 836.06 (744.57-925.44) | 1029 (819-1263) | 1295.53 (1035.63-1585.14) | 502 (447-555) |
| Dominican Republic | 25415 (22411-29374) | 515.44 (449.45-606.13) | 73332 (54273-90211) | 701.53 (518.5-864.79) | 25415 (22411-29374) |
| Ecuador | 40275 (37767-42623) | 619.34 (581.45-655.64) | 155833 (114778-216359) | 950.39 (705.52-1310.61) | 40275 (37767-42623) |
| Egypt | 344984 (287412-482687) | 1140.62 (932.58-1686.68) | 933318 (767956-1131670) | 1501.66 (1248.83-1804.26) | 344984 (287412-482687) |
| El Salvador | 28536 (25800-38102) | 784.26 (704.61-1063.58) | 118020 (88527-144949) | 1904.01 (1419.63-2342.53) | 28536 (25800-38102) |
| Equatorial Guinea | 3014 (2404-3713) | 1226.61 (976.57-1504.5) | 9444 (6004-13862) | 1323.06 (834.28-1860.78) | 3014 (2404-3713) |
| Eritrea | 15112 (11352-20868) | 900.21 (680.9-1231.14) | 32532 (22413-51729) | 930.22 (649.13-1463.48) | 15112 (11352-20868) |
| Estonia | 5689 (5107-6257) | 315.43 (284.6-344.78) | 11443 (9973-13008) | 455.76 (396.13-518.5) | 5689 (5107-6257) |
| Eswatini | 3616 (2910-4479) | 1010.49 (813.54-1237.87) | 9663 (6172-13306) | 1431.04 (945-1924.97) | 3616 (2910-4479) |
| Ethiopia | 466721 (355203-540047) | 1765.66 (1400.45-2039.43) | 524752 (440481-620029) | 966 (806.93-1140.34) | 466721 (355203-540047) |
| Fiji | 3880 (2939-5283) | 849.72 (647.64-1147.1) | 10463 (7966-13388) | 1312.98 (1004.23-1661.09) | 3880 (2939-5283) |
| Finland | 8482 (7019-9973) | 126.01 (103.95-147.99) | 18425 (15613-21274) | 145.15 (120.51-168.55) | 8482 (7019-9973) |
| France | 146507 (130176-162496) | 180.76 (160.54-199.74) | 266719 (232254-297859) | 174.03 (151.48-196.15) | 146507 (130176-162496) |
| Gabon | 8190 (6669-9730) | 1304.75 (1072.43-1558) | 19184 (11205-25666) | 1670.81 (951.89-2188.47) | 8190 (6669-9730) |
| Gambia | 4626 (3620-5727) | 892.62 (713.95-1097.58) | 13852 (10535-17707) | 1074.77 (822.36-1368.13) | 4626 (3620-5727) |
| Georgia | 15560 (12706-18459) | 264.19 (216.63-310.62) | 24983 (20472-30628) | 478.54 (394.29-587.65) | 15560 (12706-18459) |
| Germany | 300086 (265594-331706) | 249.22 (219.66-276.89) | 635444 (555490-709238) | 302.92 (265.96-338.81) | 300086 (265594-331706) |
| Ghana | 67702 (52675-92629) | 772.79 (608.91-1076.69) | 237458 (184981-297856) | 1144.99 (904.59-1420.12) | 67702 (52675-92629) |
| Greece | 59717 (54719-64585) | 424.92 (388.7-459.38) | 112985 (100431-122488) | 435.48 (396.06-475.3) | 59717 (54719-64585) |
| Greenland | 117 (102-135) | 335.71 (294.94-381.58) | 216 (178-257) | 352.22 (285.85-418.33) | 117 (102-135) |
| Grenada | 742 (678-812) | 1044.67 (958.18-1144.04) | 1684 (1467-1904) | 1487.17 (1300.75-1673.58) | 742 (678-812) |
| Guam | 535 (470-650) | 626.57 (552.23-754.35) | 1682 (1452-1875) | 846.82 (733.41-946.49) | 535 (470-650) |
| Guatemala | 44304 (42312-46649) | 920.2 (874.26-970.62) | 167359 (143276-194780) | 1387.5 (1182.18-1617.19) | 44304 (42312-46649) |
| Guinea | 39480 (32723-48547) | 900.19 (741.28-1118.65) | 67059 (52117-90058) | 896.96 (697.85-1221.3) | 39480 (32723-48547) |
| Guinea-Bissau | 8272 (6561-10146) | 1414.38 (1129.62-1709.67) | 12715 (9952-16050) | 1220.65 (970.38-1525.53) | 8272 (6561-10146) |
| Guyana | 4232 (3811-4631) | 883.22 (800.53-971.4) | 10889 (8428-13860) | 1574.82 (1227.32-1986.44) | 4232 (3811-4631) |
| Haiti | 40217 (31355-58452) | 909.47 (697.65-1415.13) | 82512 (50633-163742) | 916.41 (561.09-1807.57) | 40217 (31355-58452) |
| Honduras | 11630 (10042-13654) | 444.7 (379.37-536.18) | 46141 (35816-59163) | 682.72 (536.58-870.96) | 11630 (10042-13654) |
| Hungary | 30171 (27070-33010) | 235.27 (211.2-256.76) | 46215 (39960-53280) | 249.28 (214.43-289) | 30171 (27070-33010) |
| Iceland | 348 (298-399) | 120.54 (102.8-138.35) | 884 (755-1011) | 149.81 (127.15-172.36) | 348 (298-399) |
| India | 2650925 (2335452-3030444) | 481.56 (422.54-547.62) | 6487629 (5592187-7501487) | 521.84 (451.82-601.82) | 2650925 (2335452-3030444) |
| Indonesia | 875942 (750362-1005150) | 664.03 (575.98-770.26) | 1990496 (1655069-2360240) | 760.37 (644.61-906.69) | 875942 (750362-1005150) |
| Iran (Islamic Republic of) | 139435 (116402-174468) | 463.62 (372.31-595.58) | 356124 (310077-393518) | 467.46 (406.76-515.06) | 139435 (116402-174468) |
| Iraq | 94335 (75090-120601) | 950.26 (757.69-1205.44) | 232396 (157615-296780) | 940.84 (660.57-1195.03) | 94335 (75090-120601) |
| Ireland | 9959 (8464-11701) | 256.44 (219.03-299.89) | 18990 (16118-22176) | 246.73 (206.96-289.36) | 9959 (8464-11701) |
| Israel | 22516 (20420-24257) | 480.75 (435.3-518.1) | 52713 (46207-58485) | 417.34 (366.33-463.46) | 22516 (20420-24257) |
| Italy | 198890 (174312-221494) | 245.83 (215.46-275.21) | 328820 (282849-371176) | 216.97 (185-247.9) | 198890 (174312-221494) |
| Jamaica | 11644 (10948-12362) | 617.4 (578.48-656.55) | 25773 (19982-32671) | 831.31 (642.36-1055.41) | 11644 (10948-12362) |
| Japan | 460213 (416011-499612) | 290.39 (262.49-315.05) | 923022 (792680-1028099) | 239.94 (209.67-266.06) | 460213 (416011-499612) |
| Jordan | 12490 (10264-15613) | 749.85 (617.21-949.47) | 54300 (44489-67799) | 710.1 (581.36-884.9) | 12490 (10264-15613) |
| Kazakhstan | 49636 (42866-56925) | 351.46 (300.01-403.84) | 80323 (68213-94683) | 448.35 (381.6-524.18) | 49636 (42866-56925) |
| Kenya | 66436 (54525-94453) | 608.86 (488.93-921.43) | 230896 (188910-304540) | 863.66 (701.7-1139.37) | 66436 (54525-94453) |
| Kiribati | 460 (380-548) | 969.96 (797.74-1154.42) | 1109 (759-1633) | 1285.47 (892.42-1861.85) | 460 (380-548) |
| Kuwait | 5748 (5276-6312) | 716.82 (647.33-792.74) | 11585 (9417-13665) | 360.69 (286.12-427.92) | 5748 (5276-6312) |
| Kyrgyzstan | 12369 (10585-14240) | 346.85 (294.57-401.76) | 25313 (21875-29466) | 448.27 (386.94-523.65) | 12369 (10585-14240) |
| Lao People's Democratic Republic | 36532 (27933-46903) | 1380.46 (1067.06-1764.16) | 63295 (45478-85994) | 1174.57 (854.11-1578.45) | 36532 (27933-46903) |
| Latvia | 5859 (5024-6735) | 184.25 (159.87-210.47) | 9429 (7832-11172) | 271.26 (225.78-322.44) | 5859 (5024-6735) |
| Lebanon | 16012 (12337-21059) | 730.51 (569.04-955.01) | 39230 (33152-46228) | 630.32 (532.31-739.35) | 16012 (12337-21059) |
| Lesotho | 5121 (4128-6439) | 560.65 (450.89-707.1) | 14547 (10549-19456) | 1221.51 (900.96-1605.09) | 5121 (4128-6439) |
| Liberia | 20964 (16740-25988) | 1240.99 (1006.84-1562.17) | 35243 (26717-46785) | 1197.03 (916.25-1548.13) | 20964 (16740-25988) |
| Libya | 15078 (11908-19609) | 675.23 (524.42-903.51) | 58512 (37581-76498) | 1066.96 (712.68-1379.31) | 15078 (11908-19609) |
| Lithuania | 7168 (6069-8288) | 172.28 (147.24-198.05) | 11654 (9803-13772) | 237.6 (201.66-283.94) | 7168 (6069-8288) |
| Luxembourg | 1331 (1185-1491) | 264.27 (234.27-295.59) | 3013 (2613-3410) | 276.37 (240-313.98) | 1331 (1185-1491) |
| Madagascar | 47382 (38780-60549) | 702.76 (568.49-945.44) | 100664 (76256-129965) | 678.66 (508.16-884.33) | 47382 (38780-60549) |
| Malawi | 56194 (46192-66313) | 1003.16 (830.02-1214.13) | 110135 (88010-133712) | 1114.87 (915.06-1337.98) | 56194 (46192-66313) |
| Malaysia | 77325 (68494-86547) | 713.37 (633.73-796.59) | 238095 (207099-268811) | 817.72 (710.59-922.9) | 77325 (68494-86547) |
| Maldives | 1558 (1280-1852) | 1347.65 (1152.43-1701.12) | 2804 (2338-3335) | 719.76 (606.71-852.73) | 1558 (1280-1852) |
| Mali | 56774 (47423-68232) | 1040.28 (873.63-1283.93) | 114837 (94129-145340) | 950.46 (780.38-1210.6) | 56774 (47423-68232) |
| Malta | 1203 (1076-1338) | 303.04 (271.34-335.96) | 2837 (2467-3202) | 305.75 (267.12-347.23) | 1203 (1076-1338) |
| Marshall Islands | 212 (144-351) | 990.73 (669.09-1662.57) | 669 (256-1739) | 1587.83 (613.11-4196.39) | 212 (144-351) |
| Mauritania | 13123 (10398-16336) | 1080.69 (859.44-1353.39) | 23187 (16553-31976) | 937.84 (665.72-1292.5) | 13123 (10398-16336) |
| Mauritius | 10096 (9532-10683) | 1253.62 (1184.91-1323.58) | 38582 (35823-40737) | 2196.12 (2043.11-2318.87) | 10096 (9532-10683) |
| Mexico | 474218 (450937-498550) | 932.17 (884.86-983.28) | 1940953 (1680240-2260842) | 1489.68 (1295.18-1728) | 474218 (450937-498550) |
| Micronesia (Federated States of) | 595 (425-860) | 1017.49 (724.12-1495.09) | 1337 (979-1830) | 1629.46 (1205.42-2229.31) | 595 (425-860) |
| Monaco | 121 (100-145) | 182.17 (150.33-216.07) | 228 (194-266) | 233.45 (198.78-273.13) | 121 (100-145) |
| Mongolia | 8119 (6668-9950) | 603.16 (498.71-719.26) | 13518 (11300-16158) | 508.14 (425.37-602.36) | 8119 (6668-9950) |
| Montenegro | 2342 (1990-2734) | 382.9 (325.89-447.32) | 3844 (3139-4690) | 440.54 (361.73-537.89) | 2342 (1990-2734) |
| Morocco | 94039 (70697-146372) | 572.69 (420.85-963.46) | 289479 (223424-366427) | 861.54 (664.15-1088.94) | 94039 (70697-146372) |
| Mozambique | 59057 (48400-73912) | 681.4 (559.04-901.29) | 141035 (107847-186077) | 882.16 (684.62-1181.5) | 59057 (48400-73912) |
| Myanmar | 292701 (227943-364529) | 964.29 (759.56-1204.66) | 410628 (337703-500939) | 799.64 (662.62-966.4) | 292701 (227943-364529) |
| Namibia | 4420 (3529-5944) | 613.58 (493.19-821.67) | 10401 (7883-13859) | 695.84 (539.88-902.08) | 4420 (3529-5944) |
| Nauru | 70 (47-100) | 1204.19 (807.06-1774.85) | 127 (95-167) | 1785 (1311.59-2476.39) | 70 (47-100) |
| Nepal | 65171 (52099-79837) | 548.64 (437.43-676.33) | 160662 (126887-204193) | 652.71 (513.55-826.05) | 65171 (52099-79837) |
| Netherlands | 37878 (32720-42847) | 202.05 (174.6-228.66) | 82167 (72158-93046) | 238.48 (208.27-270.25) | 37878 (32720-42847) |
| New Zealand | 7788 (7047-8501) | 211.3 (190.84-230.61) | 20618 (18809-22453) | 263.59 (241.22-286.59) | 7788 (7047-8501) |
| Nicaragua | 20446 (18371-26070) | 922.33 (832.85-1189.34) | 86099 (68224-102148) | 1596.85 (1282.9-1885.75) | 20446 (18371-26070) |
| Niger | 41923 (33643-50607) | 842.51 (698.06-1076.13) | 80122 (59677-120176) | 687.81 (523.37-1007.48) | 41923 (33643-50607) |
| Nigeria | 506251 (409099-599064) | 880.97 (719.4-1053.33) | 1007285 (771422-1268598) | 824.39 (662.51-1015.24) | 506251 (409099-599064) |
| Niue | 19 (16-25) | 880.22 (706.01-1130.78) | 32 (20-47) | 1612.3 (1011.21-2371.92) | 19 (16-25) |
| North Macedonia | 6763 (5934-7869) | 365.47 (320.31-422.99) | 10889 (8783-13799) | 373.54 (303.82-461.85) | 6763 (5934-7869) |
| Northern Mariana Islands | 259 (200-334) | 1022.79 (833.17-1275.72) | 773 (644-914) | 1474.97 (1249.09-1715.8) | 259 (200-334) |
| Norway | 9013 (7541-10541) | 137.17 (113.6-160.28) | 17052 (14870-19536) | 168.58 (144-194.09) | 9013 (7541-10541) |
| Oman | 4965 (3836-6906) | 578.2 (442.15-815.39) | 19110 (15607-23751) | 878.99 (718.95-1088.52) | 4965 (3836-6906) |
| Pakistan | 404931 (332986-499986) | 590.5 (491.66-724.18) | 1213820 (972621-1508801) | 795.11 (645.52-990.21) | 404931 (332986-499986) |
| Palau | 97 (72-125) | 880.09 (657.44-1119.24) | 293 (214-385) | 1382.22 (1017.84-1796.05) | 97 (72-125) |
| Palestine | 9946 (7801-12853) | 979.35 (756.41-1283.12) | 23468 (20331-27681) | 885.69 (758.84-1049) | 9946 (7801-12853) |
| Panama | 7457 (6800-8224) | 442.04 (401.21-486.71) | 36062 (29067-42682) | 814.72 (656.02-963.88) | 7457 (6800-8224) |
| Papua New Guinea | 11577 (8052-15381) | 451.54 (332.06-589.01) | 36547 (30525-44537) | 515.14 (430.93-644.45) | 11577 (8052-15381) |
| Paraguay | 12158 (10659-13822) | 465.64 (403.39-535.98) | 38803 (31124-48290) | 643.36 (516.02-802.81) | 12158 (10659-13822) |
| Peru | 105337 (92303-120681) | 713.69 (619.92-822.67) | 247079 (184433-311887) | 720.9 (537.44-910.56) | 105337 (92303-120681) |
| Philippines | 354857 (323905-395725) | 919.29 (837.47-1037.38) | 1136959 (958347-1307840) | 1232.68 (1049.55-1414.45) | 354857 (323905-395725) |
| Poland | 166060 (154655-176751) | 405.68 (378.08-432.19) | 136197 (117984-158787) | 206.2 (177.91-241.21) | 166060 (154655-176751) |
| Portugal | 40610 (36847-44376) | 330.49 (300.61-360.89) | 76569 (67526-85773) | 296.47 (262.68-332.8) | 40610 (36847-44376) |
| Puerto Rico | 28212 (26770-29738) | 793 (753.43-835.16) | 51201 (43010-59665) | 821.94 (692.54-956.42) | 28212 (26770-29738) |
| Qatar | 1101 (745-1589) | 821.94 (512.07-1248.95) | 7653 (6095-9741) | 757.02 (614.47-939.61) | 1101 (745-1589) |
| Republic of Korea | 130194 (117908-142679) | 429.64 (388.71-470.41) | 200278 (171976-226706) | 225.58 (193.71-255.8) | 130194 (117908-142679) |
| Republic of Moldova | 7738 (6388-9225) | 184.45 (151.18-219.78) | 12608 (10418-14983) | 233.78 (193.33-279.17) | 7738 (6388-9225) |
| Romania | 87976 (81527-94928) | 348.63 (324-375.68) | 92610 (78865-107339) | 277.98 (236.88-323.94) | 87976 (81527-94928) |
| Russian Federation | 428507 (391751-466416) | 259.23 (236.91-281.99) | 464004 (409800-529294) | 213.61 (189.1-243.61) | 428507 (391751-466416) |
| Rwanda | 48445 (39539-56316) | 1255.64 (1043.57-1472.42) | 65862 (48112-87152) | 891.79 (663.55-1176.62) | 48445 (39539-56316) |
| Saint Kitts and Nevis | 420 (392-454) | 1164.39 (1090.21-1254.49) | 914 (747-1082) | 1350.37 (1133.2-1575.83) | 420 (392-454) |
| Saint Lucia | 885 (837-936) | 955.02 (904.03-1009.73) | 2376 (1996-2807) | 1041.57 (875.76-1229.97) | 885 (837-936) |
| Saint Vincent and the Grenadines | 569 (534-610) | 737.17 (689.71-789) | 1457 (1294-1663) | 1083.48 (962.51-1234.39) | 569 (534-610) |
| Samoa | 868 (651-1147) | 891.33 (674.94-1163.79) | 1888 (1466-2394) | 1214.39 (948.01-1524.87) | 868 (651-1147) |
| San Marino | 56 (47-66) | 167.12 (138.93-195.72) | 113 (89-140) | 145.82 (113.78-182.28) | 56 (47-66) |
| Sao Tome and Principe | 1102 (882-1290) | 1348.97 (1113.57-1530.18) | 2097 (1609-2634) | 1636.6 (1240.02-1979.71) | 1102 (882-1290) |
| Saudi Arabia | 80180 (61535-108291) | 1089.81 (832.64-1468.18) | 416093 (307761-529386) | 1762.78 (1351.11-2169.63) | 80180 (61535-108291) |
| Senegal | 49306 (41083-60783) | 1065.12 (898.19-1309.69) | 95390 (73587-131487) | 1027.56 (798.98-1391.7) | 49306 (41083-60783) |
| Serbia | 42655 (36005-50866) | 434.23 (367.69-519.71) | 58087 (48712-67910) | 374.35 (316.47-434.51) | 42655 (36005-50866) |
| Seychelles | 522 (466-589) | 886.74 (790.44-1000.76) | 1424 (1193-1643) | 1226.42 (1031.36-1409.77) | 522 (466-589) |
| Sierra Leone | 24287 (18928-30087) | 832.15 (664.2-1021.91) | 39388 (29974-52547) | 770.1 (604.34-1010.78) | 24287 (18928-30087) |
| Singapore | 8136 (7624-8708) | 355.79 (333.26-379.48) | 20705 (18435-23087) | 251.85 (223.87-280.27) | 8136 (7624-8708) |
| Slovakia | 19699 (17271-22267) | 344.18 (302.53-388.6) | 22738 (19458-26211) | 265.28 (228.19-305.88) | 19699 (17271-22267) |
| Slovenia | 5091 (4580-5635) | 220.21 (197.75-243.27) | 7512 (6152-9249) | 175.21 (142.33-217.91) | 5091 (4580-5635) |
| Solomon Islands | 1408 (669-2226) | 774.73 (410.44-1191.76) | 4011 (3105-5040) | 870.92 (699.08-1073.28) | 1408 (669-2226) |
| Somalia | 42592 (31540-57931) | 1182.7 (866.99-1615.4) | 109717 (77473-161379) | 1218.61 (853.72-1741) | 42592 (31540-57931) |
| South Africa | 155415 (136560-179958) | 616.68 (540.36-733.34) | 415624 (377739-462537) | 862.34 (784.92-953.72) | 155415 (136560-179958) |
| South Sudan | 33407 (25693-43506) | 1014.31 (768.79-1354.62) | 69366 (50852-90453) | 1345.01 (1002.09-1742.34) | 33407 (25693-43506) |
| Spain | 156194 (141120-171158) | 309.55 (280.34-339.01) | 243084 (212322-273844) | 225.37 (196.25-255.48) | 156194 (141120-171158) |
| Sri Lanka | 91811 (79288-106066) | 768.96 (660-894.39) | 161739 (117194-209551) | 628.14 (460.84-813.21) | 91811 (79288-106066) |
| Sudan | 76183 (58482-110063) | 624.87 (468.41-1022.65) | 180786 (136192-237944) | 770.74 (589.96-1012.61) | 76183 (58482-110063) |
| Suriname | 2433 (2055-2721) | 831.29 (717.62-937.39) | 7429 (5840-9298) | 1181.21 (931.76-1473.05) | 2433 (2055-2721) |
| Sweden | 19881 (16791-23071) | 141.21 (119.74-164.35) | 44455 (38202-50852) | 196.33 (169.22-224.94) | 19881 (16791-23071) |
| Switzerland | 21014 (18209-23719) | 208.22 (180.63-235.2) | 44996 (38424-51285) | 228.57 (194.36-262.67) | 21014 (18209-23719) |
| Syrian Arab Republic | 68209 (55197-84454) | 968.46 (772.3-1242.81) | 126862 (93695-168253) | 1020.57 (759.99-1328.87) | 68209 (55197-84454) |
| Taiwan (Province of China) | 93499 (86835-100493) | 604.47 (560.65-649.34) | 205078 (181400-224760) | 502.71 (447.01-550.52) | 93499 (86835-100493) |
| Tajikistan | 6728 (5319-8239) | 198.22 (154.91-245.31) | 15292 (12007-19273) | 217.64 (169.26-274.65) | 6728 (5319-8239) |
| Thailand | 306169 (262369-368081) | 746.17 (641.01-897.81) | 865932 (699035-1045232) | 859.06 (698.47-1033.12) | 306169 (262369-368081) |
| Timor-Leste | 3510 (2766-4402) | 805.69 (633.98-1037.57) | 8029 (6032-10955) | 851.98 (645.26-1147.67) | 3510 (2766-4402) |
| Togo | 16010 (13190-19324) | 839.21 (706.86-1015.63) | 42127 (31777-54924) | 888.19 (691.77-1154.82) | 16010 (13190-19324) |
| Tokelau | 10 (7-16) | 752.82 (553.02-1180.89) | 16 (12-22) | 1136.19 (878.92-1548.38) | 10 (7-16) |
| Tonga | 294 (223-388) | 481.54 (368.88-628.62) | 535 (402-689) | 639.25 (479.51-818.76) | 294 (223-388) |
| Trinidad and Tobago | 6161 (5850-6493) | 681.79 (647.7-716.4) | 20236 (15316-25658) | 1101.28 (836.59-1394.7) | 6161 (5850-6493) |
| Tunisia | 28625 (23139-40343) | 534.62 (427.15-765.07) | 85581 (62643-113057) | 677.13 (500.89-889.4) | 28625 (23139-40343) |
| Türkiye | 12416 (11128-13743) | 475.91 (423.17-536.5) | 33906 (27087-41471) | 730.88 (586.81-893.28) | 12416 (11128-13743) |
| Turkmenistan | 65 (51-93) | 888.07 (693.67-1286.52) | 127 (95-175) | 1178.46 (885-1617.17) | 65 (51-93) |
| Tuvalu | 288241 (239747-364731) | 749.18 (624.47-967.68) | 552195 (453860-666416) | 619.43 (509.5-750.05) | 288241 (239747-364731) |
| Uganda | 65235 (50387-82208) | 764.55 (582.01-980.29) | 183294 (144104-239789) | 919.24 (725.47-1193.43) | 65235 (50387-82208) |
| Ukraine | 53706 (38096-69878) | 84.8 (60.75-108.71) | 111725 (89366-135575) | 171.79 (138.89-207.44) | 53706 (38096-69878) |
| United Arab Emirates | 3892 (2982-5153) | 586.6 (434.33-801.63) | 29680 (21617-37421) | 782.88 (522.73-997.84) | 3892 (2982-5153) |
| United Kingdom | 155905 (129827-180904) | 192.44 (160.59-223.04) | 258501 (216482-299277) | 213.44 (177.75-247.35) | 155905 (129827-180904) |
| United Republic of Tanzania | 131082 (109800-160936) | 903.09 (755.1-1140.85) | 288849 (234235-364048) | 899.32 (731.73-1124.44) | 131082 (109800-160936) |
| United States of America | 839563 (749127-924830) | 276.06 (246.69-303.29) | 2969764 (2728422-3188334) | 543.3 (499.54-582.58) | 839563 (749127-924830) |
| United States Virgin Islands | 510 (432-606) | 563.51 (478.28-667.31) | 835 (611-1068) | 599.2 (452.26-758.01) | 510 (432-606) |
| Uruguay | 13717 (12910-14498) | 364.84 (343.32-385.1) | 21441 (19615-23134) | 387.07 (357.18-417.47) | 13717 (12910-14498) |
| Uzbekistan | 44533 (35798-54486) | 315.26 (247.36-392.7) | 171630 (147004-200979) | 580.79 (501.42-677.54) | 44533 (35798-54486) |
| Vanuatu | 551 (376-866) | 696.8 (485.08-1113.23) | 2161 (1586-3205) | 1042 (776.42-1542.19) | 551 (376-866) |
| Venezuela (Bolivarian Republic of) | 61836 (57241-66347) | 527.63 (484.65-568.04) | 324201 (249903-407035) | 1087.01 (843.33-1362.67) | 61836 (57241-66347) |
| Viet Nam | 305955 (223778-380082) | 671.53 (506.42-832.43) | 676321 (501845-849831) | 685.86 (514.2-847.41) | 305955 (223778-380082) |
| Yemen | 33670 (24098-53572) | 521.1 (367.11-927.38) | 82899 (60825-131865) | 509.97 (372.62-812.52) | 33670 (24098-53572) |
| Zambia | 49375 (39792-57931) | 1179.33 (995.88-1379.63) | 123798 (84564-188445) | 1289.74 (932.63-1817.44) | 49375 (39792-57931) |
| Zimbabwe | 30248 (24565-39272) | 663.96 (538.02-846.97) | 89465 (68552-116945) | 1084.27 (848.29-1401.87) | 30248 (24565-39272) |

| **TableS9: Trends in the burden of chronic kidney disease due to diabetes mellitus type 1 across different age groups: incidence, prevalence, deaths, and disability-adjusted life years (1990–2021).** | | | | | |
| --- | --- | --- | --- | --- | --- |
| Age | 1990 | | 2021 | | **EAPC_95%CI** |
|  | Number_95%UI | ASR | Number_95%UI | ASR |  |
| **Incidence** |  |  |  |  |  |
| <5 years | 40177 (30327-51820) | 6.48 (4.89-8.36) | 39963 (30393-51899) | 6.07 (4.62-7.89) | -0.17 (-0.19--0.15) |
| 5–9 years | 5221 (2679-8744) | 0.89 (0.46-1.5) | 6794 (3608-11039) | 0.99 (0.53-1.61) | 0.26 (0.14-0.37) |
| 10–14 years | 4253 (2125-7338) | 0.79 (0.4-1.37) | 7478 (4278-11601) | 1.12 (0.64-1.74) | 1.17 (1.13-1.21) |
| 15–19 years | 1616 (812-2666) | 0.31 (0.16-0.51) | 4530 (2569-6912) | 0.73 (0.41-1.11) | 3.16 (3.02-3.31) |
| 20–24 years | 1010 (469-1686) | 0.21 (0.1-0.34) | 2569 (1440-3989) | 0.43 (0.24-0.67) | 2.73 (2.59-2.87) |
| 25–29 years | 801 (378-1397) | 0.18 (0.09-0.32) | 1805 (883-2912) | 0.31 (0.15-0.5) | 2.21 (2.08-2.34) |
| 30–34 years | 857 (452-1325) | 0.22 (0.12-0.34) | 2176 (1280-3298) | 0.36 (0.21-0.55) | 2.19 (1.99-2.39) |
| 35–39 years | 1071 (649-1580) | 0.3 (0.18-0.45) | 3116 (1995-4481) | 0.56 (0.36-0.8) | 2.4 (2.23-2.57) |
| 40–44 years | 1221 (819-1809) | 0.43 (0.29-0.63) | 3734 (2613-5064) | 0.75 (0.52-1.01) | 2.17 (2-2.35) |
| 45–49 years | 1331 (859-1882) | 0.57 (0.37-0.81) | 3970 (2697-5260) | 0.84 (0.57-1.11) | 1.56 (1.45-1.66) |
| 50–54 years | 1541 (1024-2153) | 0.72 (0.48-1.01) | 4274 (2996-5835) | 0.96 (0.67-1.31) | 1.3 (1.15-1.46) |
| 55–59 years | 1441 (980-2006) | 0.78 (0.53-1.08) | 4181 (3077-5336) | 1.06 (0.78-1.35) | 1.41 (1.28-1.53) |
| 60–64 years | 1261 (864-1726) | 0.78 (0.54-1.07) | 3728 (2736-4850) | 1.16 (0.85-1.52) | 1.54 (1.41-1.67) |
| 65–69 years | 894 (594-1208) | 0.72 (0.48-0.98) | 2945 (2161-3945) | 1.07 (0.78-1.43) | 1.73 (1.55-1.91) |
| 70–74 years | 484 (343-645) | 0.57 (0.41-0.76) | 2064 (1573-2629) | 1 (0.76-1.28) | 2.12 (1.99-2.25) |
| 75–79 years | 274 (187-376) | 0.45 (0.3-0.61) | 1067 (799-1396) | 0.81 (0.61-1.06) | 2.32 (2.12-2.52) |
| 80–84 years | 115 (78-163) | 0.32 (0.22-0.46) | 550 (392-760) | 0.63 (0.45-0.87) | 2.47 (2.26-2.68) |
| 85–89 years | 30 (20-43) | 0.2 (0.13-0.28) | 161 (113-226) | 0.35 (0.25-0.49) | 2.23 (2.06-2.4) |
| 90–94 years | 5 (3-7) | 0.11 (0.07-0.16) | 33 (22-49) | 0.18 (0.12-0.27) | 2.1 (1.93-2.26) |
| 95+ years | 0 (0-1) | 0.02 (0.01-0.05) | 2 (1-5) | 0.04 (0.02-0.09) | 2.1 (1.96-2.24) |
| **Prevalence** |  |  |  |  |  |
| <5 years | 20585 (16004-25026) | 3.32 (2.58-4.04) | 16570 (12625-20896) | 2.52 (1.92-3.17) | -1.04 (-1.1--0.98) |
| 5–9 years | 82590 (63651-107503) | 14.15 (10.91-18.42) | 81823 (61096-112589) | 11.91 (8.89-16.39) | -0.61 (-0.64--0.58) |
| 10–14 years | 248386 (182162-325772) | 46.37 (34.01-60.81) | 299216 (212568-398940) | 44.88 (31.89-59.84) | -0.08 (-0.1--0.07) |
| 15–19 years | 267970 (195770-371043) | 51.59 (37.69-71.43) | 542985 (395063-742016) | 87.02 (63.31-118.92) | 2.08 (1.96-2.19) |
| 20–24 years | 355307 (262107-479792) | 72.2 (53.26-97.5) | 729958 (522576-1003554) | 122.24 (87.51-168.06) | 1.96 (1.84-2.09) |
| 25–29 years | 360500 (260016-462386) | 81.45 (58.74-104.47) | 698691 (501614-917088) | 118.76 (85.26-155.88) | 1.67 (1.56-1.79) |
| 30–34 years | 331875 (249757-419648) | 86.11 (64.8-108.88) | 698281 (519223-906079) | 115.52 (85.9-149.89) | 1.56 (1.39-1.73) |
| 35–39 years | 285008 (225734-358242) | 80.91 (64.08-101.7) | 651935 (503971-832001) | 116.24 (89.86-148.34) | 1.69 (1.53-1.85) |
| 40–44 years | 228802 (177954-278979) | 79.87 (62.12-97.38) | 558606 (432312-702662) | 111.67 (86.42-140.46) | 1.57 (1.39-1.75) |
| 45–49 years | 199123 (161178-246771) | 85.76 (69.41-106.28) | 496418 (401165-618880) | 104.84 (84.72-130.7) | 1.1 (0.97-1.23) |
| 50–54 years | 186707 (146243-240215) | 87.83 (68.8-113) | 433555 (339808-534774) | 97.44 (76.37-120.19) | 0.78 (0.65-0.91) |
| 55–59 years | 148245 (117161-184904) | 80.05 (63.26-99.84) | 360116 (284967-440748) | 91 (72.01-111.38) | 0.86 (0.75-0.97) |
| 60–64 years | 110651 (89333-137767) | 68.89 (55.62-85.78) | 266074 (215837-334956) | 83.14 (67.44-104.66) | 1 (0.86-1.15) |
| 65–69 years | 67014 (53432-81951) | 54.21 (43.23-66.3) | 189840 (153273-232590) | 68.82 (55.57-84.32) | 1.22 (1.05-1.4) |
| 70–74 years | 35892 (28728-43505) | 42.4 (33.93-51.39) | 124784 (101210-152741) | 60.62 (49.17-74.2) | 1.53 (1.36-1.69) |
| 75–79 years | 22451 (17905-27957) | 36.47 (29.09-45.42) | 70129 (55954-86551) | 53.17 (42.43-65.63) | 1.68 (1.5-1.86) |
| 80–84 years | 10822 (8409-13758) | 30.59 (23.77-38.89) | 43058 (33825-55208) | 49.16 (38.62-63.03) | 1.82 (1.67-1.97) |
| 85–89 years | 4123 (3170-5441) | 27.28 (20.98-36) | 20474 (15586-26830) | 44.78 (34.09-58.68) | 1.96 (1.8-2.13) |
| 90–94 years | 1196 (854-1636) | 27.9 (19.93-38.18) | 8388 (6049-11890) | 46.89 (33.81-66.46) | 2.02 (1.86-2.18) |
| 95+ years | 611 (302-1255) | 60 (29.62-123.25) | 4812 (2575-8836) | 88.28 (47.25-162.13) | 1.52 (1.39-1.64) |
| **Deaths** |  |  |  |  |  |
| <5 years | 271 (152-434) | 0.04 (0.02-0.07) | 90 (51-142) | 0.01 (0.01-0.02) | -3.73 (-3.87--3.59) |
| 5–9 years | 173 (90-312) | 0.03 (0.02-0.05) | 86 (46-153) | 0.01 (0.01-0.02) | -2.85 (-2.99--2.72) |
| 10–14 years | 252 (125-428) | 0.05 (0.02-0.08) | 162 (79-277) | 0.02 (0.01-0.04) | -2.11 (-2.21--2.01) |
| 15–19 years | 732 (417-1117) | 0.14 (0.08-0.22) | 505 (276-791) | 0.08 (0.04-0.13) | -1.95 (-2.06--1.85) |
| 20–24 years | 1634 (940-2492) | 0.33 (0.19-0.51) | 1301 (734-2008) | 0.22 (0.12-0.34) | -1.59 (-1.67--1.51) |
| 25–29 years | 2412 (1581-3325) | 0.54 (0.36-0.75) | 2423 (1488-3531) | 0.41 (0.25-0.6) | -1.29 (-1.45--1.12) |
| 30–34 years | 3523 (2500-4735) | 0.91 (0.65-1.23) | 4461 (3113-6199) | 0.74 (0.52-1.03) | -1.1 (-1.32--0.88) |
| 35–39 years | 5190 (3575-6819) | 1.47 (1.01-1.94) | 6588 (4433-9157) | 1.17 (0.79-1.63) | -0.95 (-1.12--0.77) |
| 40–44 years | 5571 (3833-7736) | 1.94 (1.34-2.7) | 8919 (5980-12712) | 1.78 (1.2-2.54) | -0.47 (-0.56--0.38) |
| 45–49 years | 5686 (3745-7985) | 2.45 (1.61-3.44) | 11627 (7659-16483) | 2.46 (1.62-3.48) | 0.02 (-0.04-0.07) |
| 50–54 years | 6905 (4253-10052) | 3.25 (2-4.73) | 14983 (8783-22001) | 3.37 (1.97-4.94) | 0.06 (0-0.11) |
| 55–59 years | 6064 (3435-9605) | 3.27 (1.85-5.19) | 14287 (7854-22099) | 3.61 (1.98-5.58) | 0.27 (0.19-0.34) |
| 60–64 years | 4780 (2493-8312) | 2.98 (1.55-5.18) | 11178 (5585-20128) | 3.49 (1.75-6.29) | 0.45 (0.38-0.52) |
| 65–69 years | 3340 (1612-5885) | 2.7 (1.3-4.76) | 9116 (4399-16331) | 3.3 (1.59-5.92) | 0.7 (0.6-0.8) |
| 70–74 years | 1755 (703-3500) | 2.07 (0.83-4.13) | 5195 (2201-10139) | 2.52 (1.07-4.93) | 0.88 (0.78-0.97) |
| 75–79 years | 758 (320-1551) | 1.23 (0.52-2.52) | 2146 (942-4323) | 1.63 (0.71-3.28) | 1.06 (0.99-1.14) |
| 80–84 years | 211 (88-461) | 0.6 (0.25-1.3) | 744 (318-1595) | 0.85 (0.36-1.82) | 1.48 (1.35-1.62) |
| 85–89 years | 38 (14-92) | 0.25 (0.09-0.61) | 177 (65-417) | 0.39 (0.14-0.91) | 1.86 (1.67-2.05) |
| 90–94 years | 4 (1-10) | 0.09 (0.03-0.23) | 29 (9-69) | 0.16 (0.05-0.38) | 2.32 (2.12-2.53) |
| 95+ years | 0 (0-1) | 0.03 (0.01-0.07) | 3 (1-8) | 0.06 (0.01-0.15) | 2.93 (2.72-3.14) |
| **DALYs** |  |  |  |  |  |
| <5 years | 24307 (13768-38662) | 3.92 (2.22-6.24) | 8262 (4837-12853) | 1.26 (0.73-1.95) | -3.66 (-3.8--3.53) |
| 5–9 years | 15335 (8462-26745) | 2.63 (1.45-4.58) | 8037 (4693-13627) | 1.17 (0.68-1.98) | -2.68 (-2.8--2.56) |
| 10–14 years | 20747 (10864-34734) | 3.87 (2.03-6.48) | 13816 (7440-22960) | 2.07 (1.12-3.44) | -2.01 (-2.1--1.92) |
| 15–19 years | 54076 (31100-82205) | 10.41 (5.99-15.83) | 38106 (21241-58671) | 6.11 (3.4-9.4) | -1.88 (-1.98--1.77) |
| 20–24 years | 112542 (66039-170435) | 22.87 (13.42-34.64) | 90855 (52317-139247) | 15.21 (8.76-23.32) | -1.54 (-1.62--1.46) |
| 25–29 years | 156059 (104398-212958) | 35.26 (23.59-48.11) | 158475 (101386-226882) | 26.94 (17.23-38.56) | -1.23 (-1.38--1.07) |
| 30–34 years | 211926 (153709-284353) | 54.99 (39.88-73.78) | 272513 (194447-377414) | 45.08 (32.17-62.44) | -1.01 (-1.22--0.8) |
| 35–39 years | 287626 (201254-373139) | 81.66 (57.13-105.93) | 372981 (259669-509695) | 66.5 (46.3-90.88) | -0.84 (-1.01--0.67) |
| 40–44 years | 283354 (200086-386867) | 98.91 (69.84-135.04) | 460947 (320460-642465) | 92.14 (64.06-128.43) | -0.38 (-0.46--0.29) |
| 45–49 years | 262814 (176544-361869) | 113.19 (76.03-155.85) | 544955 (369059-748979) | 115.09 (77.94-158.18) | 0.09 (0.04-0.15) |
| 50–54 years | 284065 (177515-405517) | 133.63 (83.51-190.77) | 624500 (372755-914735) | 140.36 (83.78-205.59) | 0.13 (0.08-0.18) |
| 55–59 years | 220856 (126513-348312) | 119.25 (68.31-188.07) | 524752 (298131-804102) | 132.6 (75.34-203.2) | 0.34 (0.27-0.41) |
| 60–64 years | 150590 (80202-256519) | 93.76 (49.94-159.72) | 352431 (187009-619073) | 110.12 (58.43-193.43) | 0.51 (0.44-0.58) |
| 65–69 years | 88374 (43864-152412) | 71.49 (35.49-123.3) | 241060 (118988-417006) | 87.39 (43.14-151.18) | 0.76 (0.66-0.86) |
| 70–74 years | 38120 (16136-73563) | 45.03 (19.06-86.89) | 113420 (50075-215367) | 55.1 (24.33-104.63) | 0.93 (0.82-1.03) |
| 75–79 years | 13280 (5998-26724) | 21.57 (9.74-43.41) | 37647 (17537-75027) | 28.55 (13.3-56.89) | 1.11 (1.02-1.2) |
| 80–84 years | 2956 (1338-6209) | 8.35 (3.78-17.55) | 10452 (4946-21368) | 11.93 (5.65-24.4) | 1.52 (1.37-1.66) |
| 85–89 years | 444 (188-995) | 2.94 (1.24-6.58) | 2066 (904-4423) | 4.52 (1.98-9.67) | 1.86 (1.68-2.05) |
| 90–94 years | 45 (20-97) | 1.05 (0.47-2.27) | 324 (149-673) | 1.81 (0.83-3.76) | 2.26 (2.07-2.45) |
| 95+ years | 2 (1-6) | 0.24 (0.07-0.6) | 26 (7-66) | 0.48 (0.14-1.21) | 2.83 (2.63-3.03) |

| **TableS10: National Burden of chronic kidney disease due to diabetes mellitus type 1: incidence cases, ASIR, and EAPC (1990–2021).** | | | | | |
| --- | --- | --- | --- | --- | --- |
| Country | **1990** | | **2021** | | **EAPC_95%CI** |
|  | Number_95%UI | ASR | Number_95%UI | ASR |  |
| Afghanistan | 156 (15-735) | 1.05 (0.17-4.42) | 615 (47-3018) | 1.33 (0.19-5.74) | 0.77 (0.57-0.97) |
| Albania | 40 (5-186) | 1.07 (0.16-4.82) | 47 (16-129) | 2.02 (0.51-6.92) | 2.41 (2.3-2.52) |
| Algeria | 365 (37-1700) | 1.14 (0.18-4.77) | 700 (123-2923) | 1.56 (0.28-6.48) | 1.01 (0.92-1.1) |
| American Samoa | 1 (0-4) | 1.05 (0.1-4.98) | 1 (0-2) | 1.34 (0.12-6.01) | 0.79 (0.76-0.83) |
| Andorra | 0 (0-1) | 0.92 (0.29-2.96) | 1 (1-2) | 1.19 (0.45-3.17) | 1.08 (0.99-1.17) |
| Angola | 142 (8-719) | 0.78 (0.09-3.67) | 444 (31-2198) | 0.9 (0.12-4.09) | 0.45 (0.36-0.55) |
| Antigua and Barbuda | 1 (0-3) | 1.33 (0.32-4.98) | 1 (0-4) | 1.9 (0.46-6.72) | 1.47 (1.36-1.58) |
| Argentina | 369 (66-1503) | 1.11 (0.2-4.46) | 487 (137-1608) | 1.36 (0.29-5.21) | 0.98 (0.81-1.15) |
| Armenia | 37 (5-158) | 1.03 (0.16-4.31) | 44 (14-133) | 1.72 (0.42-5.85) | 1.87 (1.58-2.15) |
| Australia | 181 (67-493) | 1.11 (0.37-3.23) | 558 (240-1128) | 1.86 (0.73-4.67) | 1.89 (1.72-2.07) |
| Austria | 82 (31-175) | 1.06 (0.33-2.79) | 203 (86-393) | 1.72 (0.64-3.63) | 1.85 (1.75-1.94) |
| Azerbaijan | 123 (17-501) | 1.51 (0.25-5.9) | 239 (67-739) | 2.62 (0.6-9.03) | 2.11 (1.87-2.34) |
| Bahamas | 3 (1-13) | 1.29 (0.25-5) | 5 (1-18) | 1.79 (0.35-6.61) | 1.33 (1.23-1.42) |
| Bahrain | 6 (1-32) | 1.06 (0.19-5.31) | 18 (5-70) | 1.5 (0.31-6.88) | 1.12 (0.98-1.26) |
| Bangladesh | 1095 (91-4917) | 0.69 (0.12-2.73) | 1310 (233-4953) | 0.88 (0.15-3.43) | 0.78 (0.74-0.81) |
| Barbados | 3 (1-11) | 1.37 (0.3-5.45) | 4 (1-13) | 1.9 (0.44-7) | 1.34 (1.23-1.46) |
| Belarus | 88 (16-330) | 1.01 (0.15-4.14) | 129 (46-349) | 1.72 (0.42-5.62) | 1.9 (1.54-2.27) |
| Belgium | 82 (29-225) | 0.9 (0.23-3.18) | 143 (61-292) | 1.11 (0.37-3.09) | 0.92 (0.82-1.02) |
| Belize | 3 (0-15) | 1.44 (0.3-5.35) | 9 (2-31) | 2.08 (0.43-7.59) | 1.4 (1.31-1.49) |
| Benin | 99 (6-492) | 1.14 (0.13-5.3) | 248 (17-1226) | 1.2 (0.15-5.46) | 0.21 (0.19-0.23) |
| Bermuda | 1 (0-2) | 1.17 (0.26-4.61) | 1 (0-2) | 1.65 (0.43-6.28) | 1.52 (1.37-1.68) |
| Bhutan | 8 (1-40) | 0.95 (0.14-4.28) | 8 (2-32) | 1.22 (0.23-4.86) | 0.96 (0.9-1.03) |
| Bolivia (Plurinational State of) | 95 (8-403) | 1.05 (0.15-4.15) | 152 (23-591) | 1.29 (0.21-4.96) | 0.84 (0.8-0.89) |
| Bosnia and Herzegovina | 35 (8-116) | 0.85 (0.18-3.1) | 42 (16-99) | 1.49 (0.41-4.2) | 1.94 (1.87-2.02) |
| Botswana | 16 (3-65) | 1.03 (0.29-3.41) | 31 (9-99) | 1.3 (0.39-4.14) | 0.87 (0.65-1.08) |
| Brazil | 1861 (1012-3297) | 1.23 (0.7-2.1) | 3599 (2453-5115) | 1.72 (1.1-2.59) | 1.25 (1.14-1.35) |
| Brunei Darussalam | 3 (1-13) | 1.2 (0.3-3.96) | 5 (1-16) | 1.32 (0.27-4.72) | 0.77 (0.46-1.07) |
| Bulgaria | 77 (21-260) | 1.11 (0.21-4.3) | 133 (50-331) | 2.45 (0.66-7.88) | 2.81 (2.68-2.94) |
| Burkina Faso | 184 (13-883) | 1.13 (0.17-5.07) | 474 (36-2413) | 1.33 (0.19-6.11) | 0.73 (0.62-0.83) |
| Burundi | 79 (8-372) | 0.95 (0.21-3.86) | 170 (23-756) | 1.01 (0.25-3.95) | 0.32 (0.24-0.4) |
| C?te d'Ivoire | 6 (0-29) | 1.07 (0.1-5.15) | 6 (1-25) | 1.2 (0.13-5.39) | 0.35 (0.26-0.44) |
| Cabo Verde | 177 (16-867) | 1.17 (0.21-5) | 225 (41-926) | 1.3 (0.25-5.3) | 0.45 (0.36-0.54) |
| Cambodia | 249 (17-1203) | 1.48 (0.2-6.44) | 703 (66-3243) | 1.68 (0.26-7.07) | 0.39 (0.29-0.5) |
| Cameroon | 555 (217-1216) | 2.05 (0.73-5.43) | 936 (411-1904) | 2.21 (0.79-6.1) | 0.89 (0.71-1.07) |
| Canada | 34 (2-159) | 0.74 (0.1-3.11) | 65 (5-301) | 0.85 (0.12-3.61) | 0.5 (0.46-0.53) |
| Central African Republic | 110 (7-562) | 1.01 (0.14-4.64) | 343 (24-1758) | 1.12 (0.17-5.13) | 0.39 (0.28-0.5) |
| Chad | 139 (14-624) | 0.99 (0.12-4.32) | 187 (49-658) | 1.34 (0.24-5.31) | 1.13 (1.03-1.23) |
| Chile | 9475 (4977-15577) | 0.86 (0.45-1.4) | 6321 (3949-9464) | 0.7 (0.38-1.14) | -0.56 (-0.67--0.45) |
| China | 434 (29-2048) | 1.07 (0.11-4.88) | 502 (94-1998) | 1.33 (0.18-5.63) | 0.83 (0.77-0.89) |
| Colombia | 7 (1-33) | 1.01 (0.18-4.13) | 9 (1-35) | 1.08 (0.2-4.29) | 0.18 (0.14-0.23) |
| Comoros | 31 (2-147) | 0.89 (0.13-3.81) | 59 (7-254) | 0.98 (0.15-4.04) | 0.39 (0.31-0.48) |
| Congo | 0 (0-1) | 0.91 (0.08-4.47) | 0 (0-1) | 1.13 (0.13-5.27) | 0.73 (0.71-0.75) |
| Cook Islands | 48 (4-230) | 1.31 (0.18-5.92) | 53 (10-214) | 1.49 (0.21-6.58) | 0.45 (0.41-0.48) |
| Costa Rica | 33 (9-105) | 0.83 (0.17-3.07) | 68 (30-141) | 1.65 (0.55-4.57) | 2.47 (2.33-2.6) |
| Croatia | 95 (13-412) | 1.03 (0.12-4.47) | 102 (27-366) | 1.35 (0.2-5.96) | 1.09 (1.01-1.17) |
| Cuba | 5 (1-20) | 0.75 (0.17-2.98) | 20 (9-42) | 1.35 (0.48-3.45) | 2.06 (1.94-2.18) |
| Cyprus | 72 (19-265) | 0.87 (0.17-3.65) | 139 (57-346) | 1.43 (0.43-4.7) | 1.7 (1.66-1.73) |
| Czechia | 232 (16-1107) | 1.18 (0.18-5.04) | 500 (49-2266) | 1.33 (0.22-5.45) | 0.4 (0.34-0.46) |
| Democratic People's Republic of Korea | 209 (16-1065) | 0.9 (0.08-4.52) | 179 (31-763) | 0.99 (0.11-4.7) | 0.28 (0.26-0.31) |
| Democratic Republic of the Congo | 515 (33-2459) | 0.78 (0.1-3.4) | 1032 (100-4682) | 0.86 (0.14-3.62) | 0.32 (0.24-0.41) |
| Denmark | 60 (24-128) | 1.15 (0.39-3.16) | 115 (52-220) | 1.58 (0.61-3.78) | 1.47 (1.31-1.64) |
| Djibouti | 5 (1-23) | 0.97 (0.24-3.54) | 15 (3-56) | 1.16 (0.3-4.02) | 0.63 (0.5-0.75) |
| Dominica | 1 (0-6) | 1.55 (0.27-6.55) | 1 (0-4) | 2.32 (0.39-10.08) | 1.53 (1.44-1.63) |
| Dominican Republic | 96 (10-450) | 1.05 (0.17-4.49) | 155 (29-612) | 1.47 (0.27-5.89) | 1.18 (1.14-1.22) |
| Ecuador | 116 (11-530) | 0.92 (0.13-3.94) | 222 (37-909) | 1.31 (0.21-5.41) | 1.38 (1.32-1.45) |
| Egypt | 915 (142-3756) | 1.37 (0.32-4.76) | 1994 (329-7874) | 1.72 (0.34-6.37) | 0.64 (0.55-0.74) |
| El Salvador | 80 (8-363) | 1.14 (0.18-4.79) | 131 (24-538) | 2.13 (0.38-8.79) | 2.44 (2.32-2.56) |
| Equatorial Guinea | 5 (0-26) | 0.73 (0.09-3.16) | 17 (2-71) | 0.98 (0.14-3.83) | 1.22 (1.12-1.32) |
| Eritrea | 47 (6-218) | 0.93 (0.19-3.66) | 81 (13-345) | 1.02 (0.23-3.95) | 0.32 (0.2-0.44) |
| Estonia | 17 (4-57) | 1.27 (0.24-4.67) | 26 (10-60) | 2.27 (0.66-6.69) | 2.11 (1.94-2.28) |
| Eswatini | 14 (2-64) | 1.34 (0.35-4.88) | 22 (5-84) | 1.77 (0.49-6.18) | 0.87 (0.61-1.12) |
| Ethiopia | 735 (205-2009) | 0.89 (0.34-2.22) | 1329 (444-3705) | 1 (0.43-2.5) | 0.43 (0.32-0.53) |
| Fiji | 12 (1-54) | 1.37 (0.2-5.85) | 15 (2-63) | 1.63 (0.21-6.96) | 0.64 (0.59-0.68) |
| Finland | 48 (19-116) | 0.92 (0.32-2.75) | 124 (59-232) | 1.56 (0.66-3.67) | 1.73 (1.61-1.85) |
| France | 425 (141-1260) | 0.81 (0.22-2.86) | 978 (436-1902) | 1.21 (0.46-3.07) | 1.33 (1.25-1.41) |
| Gabon | 13 (1-67) | 0.94 (0.12-4.36) | 23 (3-109) | 1.15 (0.18-5.16) | 0.6 (0.48-0.73) |
| Gambia | 17 (1-85) | 1.05 (0.15-4.77) | 36 (4-173) | 1.16 (0.19-5.11) | 0.28 (0.18-0.39) |
| Georgia | 55 (13-178) | 1.09 (0.22-3.73) | 52 (18-137) | 1.67 (0.46-5.08) | 1.48 (1.26-1.71) |
| Germany | 773 (311-1917) | 1.01 (0.3-3.08) | 1403 (641-2763) | 1.3 (0.51-3.34) | 1.02 (0.84-1.19) |
| Ghana | 208 (15-1010) | 0.9 (0.13-4.03) | 477 (51-2204) | 1.14 (0.17-4.89) | 0.79 (0.71-0.88) |
| Greece | 68 (22-195) | 0.8 (0.16-2.94) | 129 (55-267) | 1.09 (0.34-3.14) | 1.31 (1.17-1.46) |
| Greenland | 1 (0-3) | 1.69 (0.6-4.97) | 1 (0-2) | 1.37 (0.45-4.42) | -0.5 (-0.56--0.44) |
| Grenada | 1 (0-7) | 1.4 (0.26-6.03) | 2 (0-7) | 2.14 (0.41-8.85) | 1.53 (1.45-1.6) |
| Guam | 2 (0-8) | 1 (0.11-4.66) | 2 (0-10) | 1.54 (0.17-7.43) | 1.53 (1.46-1.6) |
| Guatemala | 169 (13-876) | 1.29 (0.19-5.91) | 313 (47-1340) | 1.99 (0.33-8.35) | 1.46 (1.43-1.48) |
| Guinea | 114 (8-534) | 1.15 (0.15-4.85) | 241 (21-1141) | 1.27 (0.2-5.36) | 0.38 (0.35-0.41) |
| Guinea-Bissau | 20 (1-101) | 1.21 (0.17-5.62) | 36 (4-171) | 1.26 (0.2-5.52) | 0.14 (0.09-0.19) |
| Guyana | 12 (1-55) | 1.14 (0.17-4.91) | 12 (2-48) | 1.58 (0.25-6.43) | 1.17 (1.12-1.22) |
| Haiti | 98 (12-463) | 1.1 (0.21-4.59) | 183 (31-739) | 1.27 (0.26-4.84) | 0.6 (0.55-0.66) |
| Honduras | 80 (5-382) | 1.06 (0.11-4.79) | 127 (14-556) | 1.18 (0.16-5.06) | 0.36 (0.33-0.39) |
| Hungary | 60 (15-235) | 0.77 (0.13-3.49) | 119 (48-302) | 1.44 (0.42-4.77) | 2.22 (2.11-2.32) |
| Iceland | 2 (1-6) | 0.79 (0.23-2.59) | 3 (1-8) | 0.87 (0.31-2.61) | 0.53 (0.42-0.63) |
| India | 10061 (5992-15824) | 0.97 (0.6-1.48) | 12162 (8065-18319) | 0.97 (0.63-1.48) | -0.01 (-0.07-0.05) |
| Indonesia | 2903 (1501-5453) | 1.39 (0.75-2.54) | 4601 (2847-7482) | 1.79 (1.06-3.03) | 1.35 (1.13-1.58) |
| Iran (Islamic Republic of) | 1164 (683-1898) | 1.72 (1.1-2.6) | 1448 (1002-2101) | 1.96 (1.26-2.97) | 0.36 (0.24-0.47) |
| Iraq | 336 (19-1708) | 1.2 (0.13-5.7) | 654 (74-3021) | 1.56 (0.21-7.06) | 0.85 (0.7-0.99) |
| Ireland | 45 (14-138) | 1.34 (0.39-4.39) | 130 (60-255) | 2.26 (0.89-5.35) | 2 (1.92-2.08) |
| Israel | 42 (10-151) | 0.85 (0.21-2.98) | 123 (43-334) | 1.24 (0.4-3.54) | 1.52 (1.41-1.63) |
| Italy | 546 (398-738) | 1.06 (0.68-1.65) | 1348 (1031-1788) | 1.69 (1.21-2.35) | 1.43 (1.26-1.61) |
| Jamaica | 32 (6-113) | 1.27 (0.3-4.19) | 37 (11-106) | 1.57 (0.38-4.98) | 1.13 (0.98-1.28) |
| Japan | 777 (548-1103) | 0.83 (0.55-1.28) | 987 (758-1280) | 0.91 (0.62-1.35) | 0.46 (0.4-0.53) |
| Jordan | 66 (5-332) | 1.24 (0.17-5.8) | 180 (27-847) | 1.58 (0.24-7.35) | 0.81 (0.73-0.89) |
| Kazakhstan | 209 (25-997) | 1.18 (0.15-5.55) | 281 (56-1137) | 1.49 (0.29-6.03) | 0.85 (0.62-1.09) |
| Kenya | 305 (193-459) | 0.97 (0.7-1.34) | 639 (465-891) | 1.24 (0.94-1.63) | 0.7 (0.52-0.88) |
| Kiribati | 1 (0-5) | 1.07 (0.1-4.81) | 2 (0-8) | 1.27 (0.13-5.59) | 0.57 (0.51-0.63) |
| Kuwait | 20 (3-87) | 1.15 (0.24-4.7) | 52 (15-173) | 1.46 (0.32-5.66) | 0.73 (0.61-0.84) |
| Kyrgyzstan | 77 (8-322) | 1.39 (0.19-5.5) | 125 (22-436) | 1.71 (0.33-5.76) | 0.67 (0.5-0.84) |
| Lao People's Democratic Republic | 80 (8-370) | 1.34 (0.23-5.4) | 122 (19-522) | 1.54 (0.27-6.38) | 0.63 (0.57-0.7) |
| Latvia | 22 (4-87) | 1.01 (0.15-4.3) | 24 (8-63) | 1.68 (0.4-5.55) | 1.63 (1.38-1.87) |
| Lebanon | 41 (5-191) | 1.13 (0.16-4.9) | 70 (14-272) | 1.53 (0.25-6.48) | 1 (0.94-1.06) |
| Lesotho | 21 (4-85) | 1.16 (0.33-3.89) | 28 (7-100) | 1.52 (0.44-4.92) | 0.76 (0.58-0.95) |
| Liberia | 42 (3-192) | 1.03 (0.13-4.29) | 79 (8-331) | 1.14 (0.16-4.49) | 0.47 (0.41-0.52) |
| Libya | 62 (7-288) | 1.15 (0.21-4.72) | 84 (21-287) | 1.53 (0.31-5.95) | 0.73 (0.61-0.85) |
| Lithuania | 35 (7-132) | 1.1 (0.18-4.45) | 35 (12-91) | 1.64 (0.4-5.54) | 1.42 (1.19-1.66) |
| Luxembourg | 3 (1-8) | 0.87 (0.25-2.94) | 9 (4-18) | 1.21 (0.48-3.08) | 1.47 (1.3-1.64) |
| Madagascar | 162 (19-738) | 0.93 (0.2-3.59) | 324 (49-1290) | 0.96 (0.22-3.43) | 0.16 (0.1-0.22) |
| Malawi | 142 (19-651) | 1.02 (0.27-3.53) | 285 (56-1069) | 1.34 (0.4-4.11) | 1.08 (0.94-1.22) |
| Malaysia | 211 (26-909) | 1.01 (0.18-4.08) | 365 (82-1323) | 1.32 (0.26-5.02) | 0.92 (0.83-1) |
| Maldives | 4 (0-19) | 1.25 (0.25-4.95) | 6 (2-19) | 1.58 (0.39-5.5) | 0.92 (0.82-1.03) |
| Mali | 163 (7-808) | 1 (0.09-4.79) | 449 (21-2249) | 1.07 (0.1-4.93) | 0.26 (0.21-0.32) |
| Malta | 4 (1-10) | 1.14 (0.37-3.26) | 8 (3-16) | 1.46 (0.5-4.05) | 1.07 (0.89-1.26) |
| Marshall Islands | 1 (0-3) | 0.97 (0.09-4.82) | 1 (0-3) | 1.13 (0.11-5.44) | 0.39 (0.3-0.49) |
| Mauritania | 37 (3-176) | 1.19 (0.19-5.05) | 82 (10-366) | 1.49 (0.3-5.98) | 0.8 (0.73-0.86) |
| Mauritius | 17 (3-62) | 1.54 (0.31-5.59) | 27 (9-74) | 2.59 (0.59-8.63) | 1.74 (1.69-1.8) |
| Mexico | 1469 (849-2512) | 1.34 (0.8-2.23) | 2443 (1596-3785) | 2.14 (1.37-3.37) | 1.73 (1.65-1.81) |
| Micronesia (Federated States of) | 2 (0-9) | 1.2 (0.15-5.74) | 2 (0-7) | 1.5 (0.21-6.92) | 0.76 (0.73-0.79) |
| Monaco | 0 (0-1) | 0.91 (0.28-2.76) | 1 (0-1) | 1.17 (0.44-2.98) | 1.06 (0.94-1.17) |
| Mongolia | 41 (4-203) | 1.44 (0.23-6.5) | 60 (12-251) | 1.75 (0.36-7.08) | 0.51 (0.46-0.56) |
| Montenegro | 8 (2-28) | 1.37 (0.29-5.22) | 13 (5-32) | 2.28 (0.71-6.65) | 1.87 (1.76-1.97) |
| Morocco | 319 (31-1481) | 0.98 (0.15-4.2) | 509 (85-2100) | 1.51 (0.23-6.35) | 1.34 (1.18-1.49) |
| Mozambique | 199 (24-925) | 1.02 (0.23-3.89) | 495 (61-2150) | 1.2 (0.26-4.49) | 0.54 (0.48-0.61) |
| Myanmar | 647 (84-2917) | 1.37 (0.24-5.82) | 967 (169-4016) | 1.76 (0.29-7.47) | 1.02 (0.91-1.13) |
| Namibia | 17 (3-73) | 1.04 (0.3-3.6) | 31 (8-109) | 1.28 (0.39-4.1) | 0.75 (0.53-0.97) |
| Nauru | 0 (0-1) | 1.11 (0.1-5.62) | 0 (0-1) | 1.36 (0.12-6.79) | 0.67 (0.55-0.8) |
| Nepal | 252 (17-1331) | 0.84 (0.1-4.13) | 351 (50-1690) | 1.13 (0.17-5.37) | 0.84 (0.77-0.91) |
| Netherlands | 111 (39-291) | 0.81 (0.24-2.66) | 291 (131-562) | 1.3 (0.48-3.33) | 2.07 (1.91-2.23) |
| New Zealand | 35 (13-86) | 1.08 (0.36-2.86) | 79 (36-159) | 1.55 (0.57-3.64) | 1.3 (1-1.59) |
| Nicaragua | 99 (6-455) | 1.64 (0.2-7.12) | 153 (22-630) | 2.31 (0.34-9.52) | 1.16 (1.13-1.2) |
| Niger | 172 (9-804) | 1.11 (0.12-4.79) | 454 (29-2042) | 1.05 (0.14-4.34) | -0.12 (-0.16--0.09) |
| Nigeria | 1753 (1098-2751) | 1.21 (0.81-1.82) | 4276 (2674-6576) | 1.38 (0.92-2.04) | 0.46 (0.36-0.55) |
| Niue | 0 (0-0) | 1.02 (0.1-4.68) | 0 (0-0) | 1.32 (0.13-5.97) | 0.88 (0.85-0.92) |
| North Macedonia | 18 (4-60) | 0.99 (0.21-3.42) | 36 (14-80) | 1.84 (0.54-4.98) | 2.27 (2.21-2.34) |
| Northern Mariana Islands | 1 (0-2) | 1.1 (0.18-4.82) | 1 (0-2) | 1.4 (0.24-6.14) | 0.82 (0.78-0.85) |
| Norway | 34 (23-49) | 0.78 (0.48-1.21) | 95 (69-129) | 1.47 (0.99-2.16) | 2.58 (2.39-2.76) |
| Oman | 28 (5-113) | 1.21 (0.37-3.83) | 81 (26-242) | 1.91 (0.66-5.6) | 1.86 (1.68-2.03) |
| Pakistan | 1665 (412-4781) | 1.06 (0.34-2.77) | 3734 (1155-9816) | 1.37 (0.47-3.44) | 0.82 (0.72-0.92) |
| Palau | 0 (0-1) | 1.32 (0.14-6.16) | 0 (0-1) | 1.91 (0.21-8.86) | 1.3 (1.21-1.39) |
| Palestine | 45 (4-229) | 1.47 (0.27-6.26) | 105 (18-457) | 1.93 (0.41-7.71) | 0.78 (0.66-0.91) |
| Panama | 30 (3-139) | 1.13 (0.18-4.92) | 64 (13-250) | 1.66 (0.3-6.72) | 1.31 (1.28-1.34) |
| Papua New Guinea | 56 (3-266) | 0.91 (0.08-4.16) | 147 (9-707) | 1.02 (0.09-4.68) | 0.36 (0.27-0.45) |
| Paraguay | 49 (5-225) | 0.92 (0.14-3.84) | 96 (18-360) | 1.43 (0.26-5.43) | 1.49 (1.39-1.59) |
| Peru | 230 (20-1146) | 0.83 (0.11-3.93) | 453 (92-1914) | 1.33 (0.26-5.75) | 1.73 (1.64-1.82) |
| Philippines | 1034 (716-1480) | 1.33 (0.97-1.83) | 2400 (1813-3173) | 2.1 (1.6-2.76) | 1.78 (1.68-1.88) |
| Poland | 418 (232-697) | 1.35 (0.71-2.31) | 405 (287-575) | 1.38 (0.83-2.24) | -0.03 (-0.08-0.03) |
| Portugal | 56 (18-164) | 0.7 (0.15-2.54) | 97 (42-192) | 0.87 (0.28-2.56) | 1.04 (0.94-1.14) |
| Puerto Rico | 45 (10-176) | 1.35 (0.28-5.39) | 46 (18-112) | 2.01 (0.46-7.93) | 1.55 (1.46-1.65) |
| Qatar | 5 (1-23) | 1.13 (0.23-4.61) | 36 (11-124) | 1.58 (0.35-5.98) | 0.99 (0.81-1.16) |
| Republic of Korea | 286 (53-1186) | 0.77 (0.13-3.33) | 243 (101-589) | 0.66 (0.14-2.65) | -0.29 (-0.45--0.12) |
| Republic of Moldova | 30 (4-114) | 0.71 (0.1-2.73) | 31 (10-86) | 1.22 (0.26-4.04) | 1.81 (1.53-2.09) |
| Romania | 163 (33-622) | 0.83 (0.13-3.38) | 230 (85-571) | 1.43 (0.4-4.57) | 1.83 (1.77-1.9) |
| Russian Federation | 2394 (1761-3193) | 1.96 (1.39-2.67) | 3231 (2583-4029) | 2.73 (2.06-3.64) | 1.06 (0.89-1.24) |
| Rwanda | 101 (11-439) | 1.01 (0.23-3.56) | 167 (31-643) | 1.17 (0.31-3.9) | 0.77 (0.66-0.88) |
| Saint Kitts and Nevis | 1 (0-2) | 1.31 (0.27-4.91) | 1 (0-3) | 1.91 (0.41-7.32) | 1.62 (1.44-1.8) |
| Saint Lucia | 2 (0-8) | 1.12 (0.18-4.96) | 2 (1-7) | 1.62 (0.29-6.79) | 1.37 (1.28-1.46) |
| Saint Vincent and the Grenadines | 2 (0-7) | 1.31 (0.25-5.51) | 2 (0-6) | 1.86 (0.34-7.75) | 1.35 (1.27-1.43) |
| Samoa | 3 (0-12) | 1.1 (0.11-4.82) | 3 (0-16) | 1.26 (0.13-5.43) | 0.39 (0.35-0.43) |
| San Marino | 0 (0-0) | 0.81 (0.21-2.65) | 0 (0-1) | 1.06 (0.34-2.9) | 1.12 (0.99-1.25) |
| Sao Tome and Principe | 3 (0-14) | 1.42 (0.16-7.04) | 4 (0-21) | 1.77 (0.23-8.22) | 0.8 (0.74-0.87) |
| Saudi Arabia | 242 (32-998) | 1.27 (0.27-4.62) | 749 (221-2125) | 2.29 (0.62-7.32) | 1.98 (1.78-2.17) |
| Senegal | 136 (9-639) | 1.05 (0.13-4.57) | 229 (23-1020) | 1.11 (0.16-4.59) | 0.12 (0.06-0.17) |
| Serbia | 69 (16-233) | 0.82 (0.15-3.1) | 121 (47-249) | 1.44 (0.41-3.89) | 2.28 (2.11-2.46) |
| Seychelles | 1 (0-3) | 1.12 (0.25-4.25) | 1 (0-4) | 1.32 (0.25-5.43) | 0.61 (0.57-0.65) |
| Sierra Leone | 76 (5-361) | 1.08 (0.13-4.62) | 146 (12-660) | 1.2 (0.16-4.98) | 0.39 (0.31-0.47) |
| Singapore | 20 (3-89) | 0.84 (0.1-3.9) | 41 (13-130) | 0.95 (0.18-4.26) | 0.69 (0.56-0.81) |
| Slovakia | 47 (9-182) | 1.04 (0.17-4.34) | 81 (29-201) | 1.7 (0.46-5.65) | 1.71 (1.64-1.79) |
| Slovenia | 12 (3-42) | 0.78 (0.13-3.09) | 25 (10-60) | 1.35 (0.38-4.16) | 2.06 (1.94-2.18) |
| Solomon Islands | 6 (1-27) | 1.29 (0.2-4.85) | 11 (1-44) | 1.29 (0.21-4.91) | -0.11 (-0.23-0.02) |
| Somalia | 114 (12-552) | 0.96 (0.22-3.89) | 334 (35-1572) | 1.08 (0.23-4.17) | 0.51 (0.45-0.57) |
| South Africa | 518 (217-1019) | 1.24 (0.6-2.26) | 777 (427-1339) | 1.45 (0.77-2.54) | 0.52 (0.34-0.7) |
| South Sudan | 79 (10-380) | 0.96 (0.24-3.8) | 153 (20-710) | 1.15 (0.28-4.52) | 0.7 (0.63-0.78) |
| Spain | 219 (74-612) | 0.7 (0.17-2.48) | 457 (217-869) | 0.83 (0.31-2.07) | 1.35 (1.13-1.58) |
| Sri Lanka | 187 (31-831) | 1.08 (0.21-4.69) | 252 (65-923) | 1.31 (0.27-5.41) | 0.68 (0.61-0.76) |
| Sudan | 291 (25-1462) | 0.96 (0.16-4.29) | 662 (88-3043) | 1.33 (0.26-5.61) | 1.06 (0.82-1.31) |
| Suriname | 5 (1-24) | 1.21 (0.15-5.51) | 8 (1-36) | 1.72 (0.2-7.71) | 1.33 (1.28-1.39) |
| Sweden | 74 (35-142) | 0.79 (0.32-1.93) | 127 (74-208) | 0.94 (0.46-1.78) | 1.18 (1.01-1.34) |
| Switzerland | 45 (15-125) | 0.77 (0.19-2.67) | 89 (38-175) | 0.92 (0.29-2.64) | 0.81 (0.7-0.93) |
| Syrian Arab Republic | 258 (30-1081) | 1.52 (0.33-5.43) | 246 (76-753) | 1.83 (0.49-6.01) | 0.74 (0.65-0.83) |
| Taiwan (Province of China) | 174 (51-579) | 0.99 (0.28-3.45) | 264 (113-599) | 1.37 (0.35-4.72) | 1.29 (1.16-1.42) |
| Tajikistan | 78 (7-373) | 1.04 (0.15-4.5) | 160 (26-655) | 1.41 (0.29-5.38) | 1.17 (0.92-1.42) |
| Thailand | 844 (152-3778) | 1.55 (0.3-6.95) | 736 (270-2299) | 1.44 (0.35-5.99) | -0.08 (-0.18-0.02) |
| Timor-Leste | 14 (1-67) | 1.15 (0.19-4.9) | 21 (2-98) | 1.22 (0.2-5.36) | 0.29 (0.21-0.37) |
| Togo | 68 (6-319) | 1.2 (0.19-4.95) | 133 (18-551) | 1.32 (0.25-5.01) | 0.31 (0.22-0.4) |
| Tokelau | 0 (0-0) | 0.88 (0.07-4.07) | 0 (0-0) | 1.09 (0.1-4.78) | 0.66 (0.56-0.77) |
| Tonga | 1 (0-6) | 0.99 (0.1-4.3) | 2 (0-7) | 1.27 (0.13-5.32) | 0.76 (0.71-0.82) |
| Trinidad and Tobago | 15 (2-67) | 1.18 (0.2-5.29) | 17 (4-62) | 1.65 (0.29-7.06) | 1.4 (1.3-1.49) |
| Tunisia | 91 (13-387) | 0.96 (0.18-3.79) | 131 (36-429) | 1.25 (0.27-4.62) | 0.83 (0.69-0.96) |
| Türkiye | 75 (9-305) | 1.58 (0.28-5.86) | 131 (27-457) | 2.48 (0.53-8.6) | 1.62 (1.47-1.77) |
| Turkmenistan | 0 (0-1) | 1.09 (0.11-5.35) | 0 (0-1) | 1.3 (0.17-6.19) | 0.61 (0.58-0.64) |
| Tuvalu | 711 (121-2804) | 1.15 (0.24-4.16) | 1160 (378-3300) | 1.59 (0.41-5.39) | 1.24 (1.13-1.35) |
| Uganda | 236 (21-1118) | 0.84 (0.19-3.3) | 546 (61-2493) | 0.96 (0.21-3.77) | 0.54 (0.4-0.69) |
| Ukraine | 383 (82-1504) | 0.91 (0.15-3.9) | 469 (165-1292) | 1.51 (0.37-5.33) | 1.87 (1.66-2.08) |
| United Arab Emirates | 33 (6-122) | 1.71 (0.47-5.7) | 245 (103-527) | 2.66 (0.82-8.1) | 1.43 (1.22-1.64) |
| United Kingdom | 492 (402-630) | 0.95 (0.74-1.31) | 632 (535-763) | 1.01 (0.8-1.29) | 0.25 (0.08-0.42) |
| United Republic of Tanzania | 435 (50-1848) | 1.17 (0.28-4.06) | 924 (136-3714) | 1.32 (0.32-4.51) | 0.43 (0.29-0.56) |
| United States of America | 4147 (2996-6166) | 1.82 (1.27-2.82) | 5204 (4082-6884) | 1.83 (1.31-2.72) | -0.08 (-0.17-0.01) |
| United States Virgin Islands | 1 (0-5) | 1.19 (0.19-4.76) | 1 (0-3) | 1.56 (0.29-6.15) | 0.98 (0.93-1.02) |
| Uruguay | 32 (7-135) | 1.08 (0.2-4.81) | 35 (11-115) | 1.3 (0.28-5.41) | 0.64 (0.54-0.74) |
| Uzbekistan | 444 (64-1771) | 1.73 (0.36-6.12) | 905 (208-2893) | 2.65 (0.61-8.39) | 1.47 (1.22-1.72) |
| Vanuatu | 2 (0-11) | 0.94 (0.12-4.09) | 4 (0-20) | 1.14 (0.15-4.98) | 0.59 (0.5-0.68) |
| Venezuela (Bolivarian Republic of) | 255 (24-1177) | 1.08 (0.15-4.68) | 328 (62-1264) | 1.36 (0.21-5.52) | 0.93 (0.84-1.02) |
| Viet Nam | 765 (100-3617) | 0.95 (0.18-4.1) | 1270 (352-4389) | 1.36 (0.32-5.11) | 1.42 (1.36-1.47) |
| Yemen | 212 (19-1078) | 0.95 (0.19-4.15) | 493 (68-2263) | 1.24 (0.28-5.07) | 1.15 (1-1.31) |
| Zambia | 125 (11-653) | 0.99 (0.19-4.43) | 263 (32-1322) | 1.07 (0.21-4.74) | 0.29 (0.14-0.44) |
| Zimbabwe | 137 (19-679) | 1.04 (0.28-4.11) | 209 (38-961) | 1.18 (0.31-4.6) | 0.15 (-0.06-0.37) |

| **TableS11: National Burden of chronic kidney disease due to diabetes mellitus type 1: prevalence cases, ASPR, and EAPC (1990–2021).** | | | | | |
| --- | --- | --- | --- | --- | --- |
| Country | **1990** | | **2021** | | **EAPC_95%CI** |
|  | Number_95%UI | ASR | Number_95%UI | ASR |  |
| Afghanistan | 4008 (1591-8304) | 45.81 (19.41-90.17) | 12162 (4404-26647) | 40.47 (16.5-80.35) | -0.4 (-0.56--0.24) |
| Albania | 1799 (852-3458) | 52.34 (26.07-98.38) | 3299 (1727-5862) | 120.18 (60.64-216.45) | 2.96 (2.81-3.11) |
| Algeria | 12090 (5484-24456) | 50.34 (24.63-97.67) | 22514 (10809-44218) | 50.68 (24.01-99.48) | 0.2 (0.03-0.37) |
| American Samoa | 12 (7-22) | 28.72 (18.62-45.89) | 16 (10-26) | 29.85 (19.2-49.31) | 0.24 (0.16-0.31) |
| Andorra | 36 (21-63) | 59.28 (35.47-104.68) | 116 (70-198) | 105.94 (59.49-186.74) | 2.1 (2-2.19) |
| Angola | 3063 (1451-5781) | 35.72 (18.57-61.44) | 10667 (4950-21512) | 38.97 (20.09-71.06) | 0.46 (0.37-0.56) |
| Antigua and Barbuda | 34 (17-62) | 56.92 (30.21-101.67) | 60 (33-106) | 61.59 (32.46-109.41) | 0.72 (0.61-0.83) |
| Argentina | 27914 (13135-54171) | 86.38 (40.72-167.2) | 47542 (22979-90603) | 100.37 (47.6-193.64) | 0.79 (0.7-0.87) |
| Armenia | 2351 (1203-4460) | 67.93 (35.13-126.45) | 3721 (1999-6679) | 118.28 (61.34-219.18) | 2.2 (1.93-2.48) |
| Australia | 17921 (8595-35396) | 99.02 (47.33-196.32) | 51450 (26623-97942) | 187.3 (92.21-361.07) | 2.23 (2.1-2.35) |
| Austria | 7226 (4246-12950) | 81.86 (46.5-153.51) | 15621 (9416-27232) | 147.14 (83.44-273.53) | 2.17 (2.07-2.27) |
| Azerbaijan | 6190 (3025-11263) | 86.44 (45.12-156.07) | 14319 (7844-25113) | 125.39 (67.3-226.01) | 1.47 (1.24-1.71) |
| Bahamas | 118 (59-217) | 45.02 (23.68-79.09) | 199 (108-341) | 47.12 (25.05-83.12) | 0.53 (0.42-0.63) |
| Bahrain | 286 (137-580) | 50.96 (25.02-102.93) | 976 (492-1889) | 55.71 (27.08-112.57) | 0.44 (0.28-0.61) |
| Bangladesh | 50820 (24532-101565) | 53.48 (27.94-101.57) | 100960 (49990-202608) | 59.39 (29.71-116.58) | 0.58 (0.49-0.68) |
| Barbados | 157 (78-299) | 59.99 (30.69-112.43) | 199 (107-362) | 63.63 (32.3-118.63) | 0.65 (0.53-0.77) |
| Belarus | 9529 (4757-19192) | 87.28 (42.38-177.45) | 16628 (8581-32945) | 176.84 (82.77-354.56) | 2.9 (2.63-3.16) |
| Belgium | 5784 (3385-9868) | 51.29 (28.97-87.91) | 11346 (6532-19627) | 85.85 (46.63-155.63) | 2.02 (1.9-2.14) |
| Belize | 91 (44-190) | 55.92 (30.33-105.94) | 261 (133-526) | 57.84 (30.67-112.12) | 0.51 (0.38-0.64) |
| Benin | 1378 (604-2862) | 36.05 (17.82-70.3) | 3852 (1729-8072) | 33.57 (16.74-64.49) | 0.11 (-0.03-0.25) |
| Bermuda | 35 (20-63) | 52.37 (30-96.54) | 39 (24-68) | 54.58 (31.07-99.76) | 0.59 (0.46-0.71) |
| Bhutan | 260 (115-527) | 44.74 (22.51-82.17) | 505 (240-965) | 62.48 (30.39-117.47) | 1.33 (1.22-1.44) |
| Bolivia (Plurinational State of) | 2115 (919-4323) | 35.47 (17.56-68.23) | 5247 (2455-10425) | 42.73 (20.3-83.57) | 0.97 (0.85-1.08) |
| Bosnia and Herzegovina | 3466 (1615-6813) | 70.63 (32.78-138.72) | 3855 (1948-6974) | 126.81 (59.3-241.68) | 2.04 (1.9-2.17) |
| Botswana | 747 (389-1413) | 73.75 (41.15-131.04) | 2094 (1128-3825) | 83.95 (46.49-149.45) | 0.78 (0.68-0.89) |
| Brazil | 87279 (67556-112620) | 60.39 (47.66-76.16) | 255062 (196750-325787) | 107.07 (81.94-136.04) | 2.33 (2.19-2.46) |
| Brunei Darussalam | 281 (142-535) | 108.52 (61.23-196.61) | 481 (279-843) | 94.84 (54.06-166.56) | -0.24 (-0.41--0.07) |
| Bulgaria | 6222 (3040-11856) | 72.41 (34.56-141.93) | 8250 (4158-14673) | 137.11 (65.47-256.34) | 2.29 (2.14-2.45) |
| Burkina Faso | 3608 (1763-6711) | 50.41 (27.18-90.86) | 8913 (4227-16955) | 48.28 (25.59-85.08) | 0.07 (-0.07-0.21) |
| Burundi | 2990 (1514-5820) | 69.01 (37-129.23) | 9545 (4513-19153) | 85 (43.78-163.42) | 1.14 (1.01-1.27) |
| C?te d'Ivoire | 96 (45-199) | 33.21 (17.46-61.4) | 198 (104-380) | 33.8 (18.29-64.25) | 0.19 (0.06-0.33) |
| Cabo Verde | 8653 (3938-17392) | 99.97 (48.55-190.42) | 18095 (8379-36343) | 101.69 (48.1-200.27) | 0.53 (0.42-0.64) |
| Cambodia | 3954 (1970-7588) | 48.39 (26.99-85.85) | 14196 (7046-27188) | 53.35 (28.72-97.62) | 0.6 (0.51-0.69) |
| Cameroon | 77331 (36943-142848) | 252.73 (120.46-464.46) | 125672 (62925-233442) | 322.46 (155.61-601.8) | 1.13 (1.04-1.23) |
| Canada | 841 (411-1576) | 36.82 (20.18-62.63) | 1807 (873-3409) | 36.81 (19.53-65.03) | 0.22 (0.15-0.29) |
| Central African Republic | 1740 (779-3294) | 37.57 (19.19-67.27) | 5364 (2386-10304) | 39.35 (20.35-71.47) | 0.41 (0.27-0.56) |
| Chad | 8729 (3895-18103) | 61.73 (28.95-124.85) | 16635 (7833-32848) | 85.78 (39-172.18) | 1.32 (1.21-1.43) |
| Chile | 249863 (208019-294978) | 22.11 (18.47-26.04) | 493149 (411923-583500) | 28.32 (23.95-33.23) | 1.23 (1.03-1.44) |
| China | 5952 (2745-12647) | 18.49 (9.62-35.49) | 12903 (7347-23326) | 25.18 (14.07-45.98) | 1.56 (1.34-1.78) |
| Colombia | 198 (94-385) | 53.65 (27.42-97.21) | 433 (216-838) | 58.68 (29.72-108.29) | 0.56 (0.49-0.64) |
| Comoros | 1017 (501-1936) | 51.47 (28.38-93.55) | 2668 (1341-5319) | 51.69 (27.55-99.25) | 0.43 (0.34-0.52) |
| Congo | 5 (3-9) | 28.58 (19.29-45.65) | 6 (4-9) | 30.63 (20.49-49.72) | 0.41 (0.35-0.48) |
| Cook Islands | 590 (281-1254) | 20.02 (10.8-39.04) | 1110 (621-2105) | 22.51 (12.13-44.72) | 0.66 (0.55-0.77) |
| Costa Rica | 3165 (1529-5973) | 62.99 (29.23-121.11) | 5900 (2986-10406) | 148.64 (69.84-266.67) | 3.07 (2.92-3.22) |
| Croatia | 3509 (1826-6410) | 31.22 (16.47-56.85) | 3702 (2146-6459) | 30.77 (16.21-56.95) | 0.55 (0.36-0.74) |
| Cuba | 389 (202-726) | 48.71 (25.34-90.84) | 1899 (997-3472) | 123.76 (61.5-229.89) | 3.29 (3.13-3.45) |
| Cyprus | 6443 (3157-11956) | 62.08 (29.57-116.41) | 12109 (6271-21738) | 127.32 (60.26-234.45) | 2.69 (2.54-2.84) |
| Czechia | 3993 (2026-7864) | 41.09 (23.07-72.51) | 10691 (5538-20790) | 43.71 (23.94-80.34) | 0.48 (0.39-0.57) |
| Democratic People's Republic of Korea | 6683 (4268-10548) | 32.68 (20.83-51.37) | 11403 (7478-17754) | 37.02 (23.55-59.65) | 0.53 (0.5-0.57) |
| Democratic Republic of the Congo | 13232 (6373-24725) | 43 (23.58-76.64) | 35447 (17470-66959) | 45.89 (25.26-82.39) | 0.52 (0.43-0.6) |
| Denmark | 5230 (3058-8862) | 87.52 (50.55-152.71) | 9047 (5246-15085) | 133.29 (75.41-230.84) | 1.74 (1.56-1.93) |
| Djibouti | 259 (129-473) | 74.25 (40.4-129.03) | 1084 (549-1955) | 84.66 (44.11-151.78) | 0.77 (0.68-0.86) |
| Dominica | 31 (17-59) | 45.9 (26.07-82.78) | 36 (20-66) | 50.64 (27.34-92.62) | 0.7 (0.6-0.81) |
| Dominican Republic | 2675 (1356-5056) | 39.88 (21.94-72.41) | 5038 (2796-9144) | 44.23 (24.76-79.88) | 0.44 (0.33-0.55) |
| Ecuador | 2608 (1196-5394) | 26.68 (13.52-52.07) | 5855 (2882-11355) | 31.39 (15.62-60.66) | 0.84 (0.74-0.93) |
| Egypt | 43523 (19499-82750) | 85.44 (40.19-160.4) | 82698 (35145-165630) | 77.51 (34.83-156.04) | -0.32 (-0.36--0.27) |
| El Salvador | 1283 (574-2587) | 25.86 (13.21-47.89) | 2317 (1135-4437) | 35.35 (17.82-67.17) | 1.52 (1.38-1.67) |
| Equatorial Guinea | 125 (64-241) | 36.19 (19.75-64.44) | 574 (277-1147) | 42.16 (22.77-78.51) | 0.81 (0.71-0.92) |
| Eritrea | 1581 (766-3198) | 57.76 (30.4-111.44) | 3869 (1902-8068) | 62.71 (32.32-128.71) | 0.57 (0.49-0.65) |
| Estonia | 1974 (1000-3963) | 120.61 (58.52-251.18) | 2658 (1431-4934) | 200.48 (101.28-391.91) | 2.04 (1.89-2.18) |
| Eswatini | 438 (212-857) | 72.53 (40.13-126.88) | 949 (472-1852) | 86.38 (45.52-156.83) | 0.79 (0.59-0.98) |
| Ethiopia | 19084 (13376-26531) | 47.69 (34.61-64.53) | 69257 (47035-101817) | 72.2 (50.55-102.13) | 1.94 (1.78-2.11) |
| Fiji | 353 (199-671) | 49.36 (30.24-86.31) | 480 (282-852) | 50.7 (29.9-90.09) | 0.43 (0.35-0.51) |
| Finland | 6195 (3380-10637) | 108.02 (57.68-190.35) | 12183 (7008-19930) | 182.14 (100.67-310.64) | 1.86 (1.72-2) |
| France | 32584 (18475-59197) | 52.56 (29.44-97.82) | 69807 (40815-124869) | 93.55 (51.65-175.81) | 1.81 (1.74-1.89) |
| Gabon | 373 (199-666) | 44.32 (25.39-78.76) | 829 (430-1518) | 47.92 (26.01-85.13) | 0.58 (0.49-0.67) |
| Gambia | 322 (152-610) | 41.98 (22.91-75.39) | 934 (445-1753) | 45.28 (23.78-82.2) | 0.48 (0.4-0.56) |
| Georgia | 4697 (2502-8322) | 81.17 (42.79-143.64) | 4019 (2228-6907) | 108.4 (57.56-191.01) | 0.89 (0.62-1.16) |
| Germany | 55777 (30770-100998) | 60.17 (32-109.54) | 89230 (51813-152679) | 89.92 (47.94-162.27) | 1.49 (1.34-1.64) |
| Ghana | 3968 (1981-7701) | 32.11 (16.88-60.34) | 11761 (5774-23239) | 37.08 (18.88-71.58) | 0.58 (0.51-0.65) |
| Greece | 4061 (2353-6642) | 35.41 (19.41-58.95) | 8308 (4741-13836) | 67.17 (36.15-114.97) | 2.4 (2.29-2.5) |
| Greenland | 138 (69-269) | 222.85 (117.99-426.59) | 101 (50-195) | 168.86 (82.15-338.05) | -0.82 (-0.94--0.69) |
| Grenada | 37 (19-72) | 46.07 (26.05-83.48) | 56 (31-101) | 51.21 (27.81-97.34) | 0.71 (0.62-0.8) |
| Guam | 36 (22-62) | 28.86 (18.61-46.86) | 59 (38-93) | 34.8 (21.42-57.58) | 0.8 (0.74-0.87) |
| Guatemala | 2245 (992-4740) | 29.92 (15.6-55.29) | 7633 (3547-15498) | 46.18 (22.94-90.3) | 1.37 (1.22-1.51) |
| Guinea | 2222 (1095-4233) | 46.18 (24.27-83.74) | 5652 (2656-10862) | 51.38 (26.65-93.9) | 0.62 (0.45-0.78) |
| Guinea-Bissau | 336 (153-651) | 41.7 (21.33-77.14) | 796 (357-1561) | 44.97 (22.83-84.24) | 0.53 (0.45-0.61) |
| Guyana | 319 (154-667) | 41.71 (22.07-83.36) | 341 (177-703) | 43.01 (22.61-87.87) | 0.4 (0.27-0.53) |
| Haiti | 2798 (1310-5430) | 48.28 (24.59-92.6) | 7209 (3321-14381) | 55.14 (26.77-107.93) | 0.77 (0.69-0.86) |
| Honduras | 820 (325-1743) | 17.87 (8.5-33.91) | 2324 (1029-4660) | 22.4 (10.65-43.19) | 0.96 (0.84-1.08) |
| Hungary | 5518 (2794-10871) | 53.82 (25.91-110.79) | 10340 (5502-18420) | 119.44 (58.47-230.34) | 3 (2.82-3.18) |
| Iceland | 118 (61-209) | 45.12 (23.54-79.87) | 259 (142-453) | 66.52 (34.7-121.04) | 1.48 (1.37-1.59) |
| India | 430767 (343418-546566) | 53.21 (43.11-66.75) | 1023379 (814479-1308816) | 68.59 (54.74-86.8) | 0.9 (0.8-1) |
| Indonesia | 204561 (143589-274096) | 116.03 (83.91-153.14) | 509620 (379337-674139) | 166.66 (123.83-220.01) | 2.76 (2.12-3.41) |
| Iran (Islamic Republic of) | 35542 (28194-44194) | 70.26 (56.84-86.52) | 69704 (55954-89028) | 76.18 (60.69-97.42) | 0.62 (0.41-0.83) |
| Iraq | 6106 (2520-12361) | 33.86 (16.01-66.06) | 15575 (6849-31882) | 36.18 (16.79-72.89) | 0.37 (0.2-0.55) |
| Ireland | 2706 (1585-4791) | 74.28 (43.33-132.28) | 11234 (6718-18507) | 199.16 (115.79-337.22) | 3.42 (3.33-3.51) |
| Israel | 2282 (1338-3875) | 48.13 (28.42-81.48) | 9277 (5209-15541) | 97.37 (53.05-166.94) | 2.79 (2.65-2.94) |
| Italy | 51267 (40815-64751) | 79.47 (62.96-100.92) | 129675 (103720-163392) | 185.1 (145.31-236.31) | 2.44 (2.14-2.74) |
| Jamaica | 1270 (674-2305) | 56.93 (32.67-100.73) | 1674 (911-2959) | 54.77 (29.72-96.2) | 0.47 (0.34-0.61) |
| Japan | 82252 (67424-98974) | 58.59 (47.83-70.71) | 105387 (86745-130630) | 76.45 (62.74-96.34) | 1.17 (1.03-1.32) |
| Jordan | 2182 (845-5082) | 57.25 (25.44-124.35) | 6695 (3010-14673) | 50.04 (23.29-106.24) | -0.26 (-0.38--0.14) |
| Kazakhstan | 8747 (4437-16682) | 53.65 (28.35-99.3) | 13294 (7262-24173) | 69.89 (37.54-128.24) | 1.16 (0.93-1.39) |
| Kenya | 12917 (10639-15425) | 77.21 (64.46-90.76) | 52629 (42332-62948) | 114.19 (93.17-135.96) | 1.55 (1.46-1.64) |
| Kiribati | 21 (12-45) | 31.69 (19.78-60.09) | 42 (23-87) | 35.15 (21.09-69.07) | 0.51 (0.44-0.58) |
| Kuwait | 825 (375-1607) | 45.58 (22.67-84.7) | 2893 (1403-5789) | 54.9 (26.18-109.52) | 0.61 (0.53-0.69) |
| Kyrgyzstan | 2273 (1105-4207) | 55.47 (28.87-97.23) | 5457 (2796-9819) | 81.31 (42.34-144.68) | 1.38 (1.12-1.65) |
| Lao People's Democratic Republic | 3502 (1571-6986) | 97.26 (47.35-185.74) | 7467 (3311-14822) | 96.31 (44.86-185.82) | 0.56 (0.4-0.71) |
| Latvia | 2265 (1191-4455) | 82.59 (41.21-167.64) | 2580 (1367-4755) | 146 (69.99-291.88) | 2.15 (1.95-2.35) |
| Lebanon | 1369 (610-2772) | 47 (21.78-90.8) | 2922 (1304-5704) | 49.97 (22.24-96.61) | 0.47 (0.36-0.58) |
| Lesotho | 1028 (538-1968) | 86.02 (47.84-165.04) | 1623 (845-3185) | 90.78 (50.64-171.94) | 0.19 (0.12-0.26) |
| Liberia | 730 (349-1467) | 36.55 (19.69-67.93) | 1857 (884-3830) | 37.78 (19.43-72.55) | 0.21 (0.14-0.28) |
| Libya | 1953 (780-4200) | 49.45 (22.51-99.49) | 4132 (1888-8662) | 54.33 (24.8-114.62) | 0.31 (0.23-0.38) |
| Lithuania | 3796 (1880-7600) | 98.89 (47.15-199.58) | 4605 (2377-8665) | 169.2 (79.58-320.42) | 2.17 (2.03-2.31) |
| Luxembourg | 251 (143-436) | 57.54 (32.01-101.01) | 772 (439-1321) | 101.72 (54.66-179.7) | 2.16 (2.02-2.3) |
| Madagascar | 5939 (2906-11052) | 63.68 (34.15-112.76) | 18840 (8938-36631) | 74.27 (37.35-141.55) | 0.88 (0.77-0.98) |
| Malawi | 7167 (3508-13541) | 94.96 (49.64-170.79) | 21552 (10157-40722) | 130.48 (67.97-241.41) | 1.49 (1.34-1.63) |
| Malaysia | 12701 (5992-26296) | 76.06 (37.92-148.6) | 31522 (15165-64089) | 91.04 (44.57-184.15) | 1.11 (0.93-1.29) |
| Maldives | 173 (81-355) | 94.92 (48.7-182.86) | 636 (331-1248) | 101.85 (53.91-194.22) | 0.71 (0.49-0.92) |
| Mali | 1826 (802-3679) | 24.46 (11.82-45.52) | 5217 (2149-10724) | 25.27 (11.88-47.61) | 0.18 (0.04-0.32) |
| Malta | 348 (192-636) | 87.24 (47.84-161.44) | 590 (329-1058) | 117.63 (62.37-222.1) | 1.13 (1.07-1.2) |
| Marshall Islands | 11 (5-21) | 27.01 (16.58-43.6) | 15 (9-25) | 27.37 (16.82-43.41) | 0.12 (0.06-0.17) |
| Mauritania | 797 (384-1466) | 48.63 (25.76-86.76) | 2342 (1128-4311) | 63.71 (33.02-111.63) | 1.25 (1.12-1.37) |
| Mauritius | 1281 (598-2503) | 108.61 (53.47-203.06) | 1659 (902-2879) | 121.13 (62.28-222.53) | 0.28 (0.21-0.36) |
| Mexico | 24692 (19242-31151) | 30.31 (24.35-37.83) | 56832 (46584-69856) | 41.87 (34.25-51.49) | 1.7 (1.49-1.91) |
| Micronesia (Federated States of) | 32 (17-63) | 35.64 (21.72-57.02) | 39 (23-66) | 37.44 (22.58-60.34) | 0.24 (0.14-0.35) |
| Monaco | 25 (15-43) | 63.55 (34.7-106.71) | 48 (28-78) | 100.57 (53.35-172.65) | 1.69 (1.58-1.8) |
| Mongolia | 1174 (598-2367) | 62.36 (35.95-114.37) | 2588 (1426-4876) | 79.57 (44.72-149.09) | 0.96 (0.86-1.07) |
| Montenegro | 476 (221-896) | 72.75 (33.66-137.36) | 882 (443-1550) | 146.51 (70.21-267.77) | 2.6 (2.44-2.75) |
| Morocco | 11148 (4602-24580) | 44.84 (19.79-93.44) | 16063 (7106-33477) | 41.89 (18.27-87.8) | -0.27 (-0.38--0.15) |
| Mozambique | 9265 (4713-17001) | 87.42 (46.68-161.42) | 23254 (10943-44517) | 89.77 (45.3-173.02) | 0.22 (0.12-0.31) |
| Myanmar | 37238 (17086-76314) | 95.32 (47.2-182.02) | 61612 (29007-119987) | 104.87 (49.63-202.37) | 1.01 (0.82-1.21) |
| Namibia | 760 (416-1377) | 68.7 (38.88-115.13) | 1933 (1064-3450) | 83.7 (48.18-144.42) | 1.03 (0.91-1.14) |
| Nauru | 3 (2-6) | 30.94 (19.51-54.33) | 3 (2-7) | 32.5 (20.22-56.45) | 0.46 (0.36-0.56) |
| Nepal | 5702 (2616-11821) | 31.22 (15.76-57.05) | 11320 (5497-21554) | 36.29 (18.12-66.13) | 0.41 (0.25-0.57) |
| Netherlands | 14742 (7561-26945) | 88.81 (45-161.38) | 29832 (16592-53031) | 150.93 (79.82-274.16) | 1.93 (1.77-2.1) |
| New Zealand | 2609 (1420-4619) | 72.18 (39.17-127.89) | 4960 (2853-8549) | 88.15 (49.96-153.01) | 0.59 (0.24-0.95) |
| Nicaragua | 921 (369-2074) | 23.72 (11.6-47.14) | 2045 (1044-4050) | 29.68 (15.51-57.94) | 1 (0.91-1.08) |
| Niger | 1968 (848-4061) | 31.49 (15.68-60.28) | 6544 (2678-13785) | 34.48 (16.36-69.96) | 0.55 (0.47-0.63) |
| Nigeria | 39367 (32638-47084) | 55.28 (46.02-66.04) | 141603 (113120-178098) | 76.79 (62-94.22) | 1.37 (1.26-1.49) |
| Niue | 1 (0-1) | 30.94 (19.58-49.16) | 1 (0-1) | 31.26 (19.24-50.22) | 0.18 (0.13-0.23) |
| North Macedonia | 1660 (803-3310) | 79.65 (38.49-158.75) | 3173 (1680-6046) | 144.48 (71.76-279.89) | 2.24 (2.09-2.39) |
| Northern Mariana Islands | 15 (9-24) | 36.07 (23.48-56.45) | 22 (14-34) | 39.76 (24.52-64.1) | 0.47 (0.42-0.52) |
| Norway | 3498 (2762-4335) | 73.49 (57.41-91.88) | 10151 (7865-12932) | 159.86 (121.13-207.91) | 2.97 (2.8-3.15) |
| Oman | 1855 (957-3642) | 102.28 (55.65-188.33) | 6857 (3625-12812) | 128.33 (68.14-239.32) | 1.54 (1.26-1.81) |
| Pakistan | 53448 (32336-85363) | 54.04 (33.77-83.38) | 160243 (99723-250211) | 68.33 (43.34-104.56) | 0.77 (0.58-0.96) |
| Palau | 5 (3-9) | 32.47 (20.49-56.36) | 7 (5-11) | 33.95 (21.56-58.72) | 0.26 (0.22-0.3) |
| Palestine | 1207 (535-2549) | 68.12 (32.74-136.17) | 4322 (1927-9434) | 82.72 (38.1-170.61) | 0.73 (0.6-0.87) |
| Panama | 626 (304-1215) | 27.3 (14.47-50.35) | 1609 (838-2973) | 36.83 (19.11-67.97) | 1.22 (1.13-1.3) |
| Papua New Guinea | 1140 (641-2069) | 32.34 (20.56-51.65) | 3346 (1913-5899) | 34.52 (21.06-55.65) | 0.43 (0.38-0.49) |
| Paraguay | 1426 (644-3124) | 38.17 (18.43-80.39) | 4440 (2051-9671) | 58.68 (27.71-125.72) | 1.56 (1.43-1.7) |
| Peru | 5947 (2753-12191) | 28.87 (14.83-55.37) | 16365 (8046-32289) | 43.24 (21.4-84.86) | 1.61 (1.49-1.72) |
| Philippines | 54156 (44062-66304) | 98.66 (81.42-120.18) | 175968 (141237-221543) | 150.47 (121.41-188.88) | 2 (1.77-2.23) |
| Poland | 27773 (21183-35628) | 74.76 (56.69-96.98) | 44440 (34232-56341) | 126.8 (95.11-162.26) | 1.95 (1.83-2.06) |
| Portugal | 4452 (2734-7559) | 40.5 (24.1-71) | 7893 (4827-13660) | 63.69 (35.47-114.18) | 1.93 (1.79-2.07) |
| Puerto Rico | 2282 (1269-3857) | 62.62 (35.39-105.18) | 2424 (1471-3913) | 67.93 (38.16-115.59) | 0.55 (0.44-0.66) |
| Qatar | 269 (139-506) | 53.17 (28.52-97.39) | 2239 (1189-4096) | 60.59 (31.66-113.85) | 0.41 (0.24-0.57) |
| Republic of Korea | 24368 (11324-51270) | 50.07 (23.78-102.75) | 27904 (16244-47521) | 45.73 (24.92-83.15) | -0.22 (-0.58-0.15) |
| Republic of Moldova | 2793 (1474-5339) | 61.96 (32.27-118.11) | 4300 (2252-8202) | 118.56 (56.54-231.3) | 2.5 (2.23-2.77) |
| Romania | 10342 (4691-21350) | 43.92 (19.41-91.87) | 16958 (8315-31279) | 99.7 (46.9-196.49) | 2.87 (2.78-2.96) |
| Russian Federation | 140986 (121408-162865) | 87.67 (75.21-100.88) | 277267 (234533-330687) | 190.33 (158.8-226.28) | 2.9 (2.69-3.1) |
| Rwanda | 4527 (2172-9199) | 81.75 (42.07-157.05) | 14222 (6639-29145) | 116.27 (56.21-223.53) | 1.83 (1.62-2.03) |
| Saint Kitts and Nevis | 21 (10-40) | 52.11 (28.82-94.57) | 36 (21-67) | 55.88 (30.49-106.05) | 0.78 (0.62-0.94) |
| Saint Lucia | 51 (25-101) | 38.95 (21.73-70.31) | 83 (48-140) | 43.03 (23.77-77.38) | 0.74 (0.64-0.84) |
| Saint Vincent and the Grenadines | 49 (24-96) | 47.02 (26.12-84.41) | 59 (33-105) | 49.7 (26.03-90.69) | 0.62 (0.5-0.73) |
| Samoa | 45 (24-93) | 30.41 (18.56-52.3) | 62 (35-113) | 30.71 (18.59-51.8) | 0.12 (0.06-0.17) |
| San Marino | 16 (9-27) | 58.2 (33.78-101.51) | 40 (23-67) | 98.15 (55.72-180) | 1.95 (1.81-2.09) |
| Sao Tome and Principe | 42 (20-87) | 42.9 (23.04-78.36) | 90 (46-173) | 44.9 (24.56-79.33) | 0.34 (0.27-0.41) |
| Saudi Arabia | 9642 (4267-19912) | 63.53 (30.86-124.55) | 37082 (18303-79696) | 85.17 (41.96-176.95) | 1.09 (0.94-1.25) |
| Senegal | 1992 (942-4101) | 31.87 (16.71-58.39) | 5176 (2382-10037) | 36 (18.04-68.99) | 0.33 (0.24-0.42) |
| Serbia | 6628 (3267-12523) | 67.58 (32.43-129.01) | 11824 (5951-20925) | 137.27 (66.26-254.09) | 2.74 (2.57-2.92) |
| Seychelles | 57 (30-115) | 82.5 (43.97-158.52) | 73 (40-135) | 64.8 (34.53-125.46) | -0.54 (-0.66--0.42) |
| Sierra Leone | 1378 (671-2482) | 40.28 (21.72-68.89) | 3308 (1575-6255) | 42.58 (21.98-76.19) | 0.32 (0.24-0.4) |
| Singapore | 1733 (823-3738) | 50.79 (25.28-106.89) | 4629 (2828-8146) | 70.89 (39.08-137.85) | 1.47 (1.14-1.8) |
| Slovakia | 3228 (1461-6107) | 60.83 (27.15-116.6) | 6310 (3226-11130) | 123.15 (57.54-231.28) | 2.61 (2.48-2.74) |
| Slovenia | 1248 (583-2545) | 60.94 (27.64-125.28) | 2233 (1127-4086) | 122.33 (57.45-237.33) | 2.78 (2.63-2.93) |
| Solomon Islands | 127 (69-258) | 45.51 (28.52-79) | 329 (185-644) | 51.52 (31.36-92.17) | 0.51 (0.45-0.57) |
| Somalia | 4135 (2067-8006) | 67.55 (36.18-120.81) | 13603 (6388-28772) | 77.91 (39.8-146.74) | 0.77 (0.68-0.85) |
| South Africa | 19186 (14347-25080) | 59.54 (45.43-76.87) | 45040 (33495-60638) | 74.81 (55.97-99.81) | 1.08 (0.93-1.22) |
| South Sudan | 3237 (1674-6301) | 69.26 (38.7-123.91) | 6530 (3125-12514) | 80.31 (42.15-147.44) | 0.79 (0.69-0.9) |
| Spain | 18235 (9787-33137) | 43.88 (23.33-81.65) | 41728 (24155-70742) | 79.53 (44.1-143.72) | 2.78 (2.39-3.17) |
| Sri Lanka | 13165 (6346-25197) | 77.01 (38.74-150.06) | 19492 (9717-37055) | 84.27 (40.22-164.65) | 0.6 (0.4-0.79) |
| Sudan | 8860 (3735-18925) | 48.03 (22.25-95.75) | 24537 (10025-53058) | 56.3 (25.51-113.81) | 0.68 (0.56-0.79) |
| Suriname | 122 (64-225) | 31.87 (18.11-56.51) | 191 (108-335) | 32.08 (17.6-56.95) | 0.18 (0.12-0.24) |
| Sweden | 9265 (6103-14269) | 93.95 (60.49-148.46) | 17664 (11601-27056) | 151.94 (95.53-241.96) | 1.99 (1.87-2.11) |
| Switzerland | 3589 (2007-6292) | 46.26 (25.3-79.6) | 7716 (4499-13356) | 74.46 (40.67-133.63) | 1.73 (1.5-1.97) |
| Syrian Arab Republic | 7611 (3424-15050) | 69.57 (35.24-126.89) | 12198 (5702-22713) | 85.01 (39.05-160.8) | 1 (0.92-1.09) |
| Taiwan (Province of China) | 12372 (8364-19546) | 61.2 (42.05-94.41) | 24725 (16927-38329) | 81.29 (53.02-131.11) | 1.33 (1.08-1.59) |
| Tajikistan | 3287 (1633-6016) | 72.41 (39.04-128.63) | 10355 (5022-19132) | 104.46 (53.08-191.33) | 1.37 (1.19-1.55) |
| Thailand | 53215 (25110-106824) | 90.08 (43.75-177.02) | 67706 (34655-130976) | 92.46 (44.32-182.14) | 0.63 (0.47-0.8) |
| Timor-Leste | 599 (260-1295) | 86.29 (42.68-171.12) | 1109 (452-2493) | 82.77 (37.2-178.45) | 0.1 (0.02-0.19) |
| Togo | 1468 (720-2943) | 53.88 (29.3-98.48) | 4600 (2321-9244) | 62.2 (32.71-118.29) | 0.69 (0.59-0.79) |
| Tokelau | 0 (0-1) | 26.76 (16.78-44.25) | 0 (0-1) | 29.66 (18.39-49.69) | 0.5 (0.45-0.56) |
| Tonga | 29 (15-52) | 32.77 (20.74-51.39) | 36 (21-64) | 35.82 (21.84-59.85) | 0.45 (0.4-0.51) |
| Trinidad and Tobago | 517 (275-1067) | 42.55 (24.04-83.48) | 625 (365-1122) | 43.32 (24.03-83.8) | 0.54 (0.41-0.67) |
| Tunisia | 4261 (1897-8949) | 52.08 (25.25-101.55) | 6506 (3214-12841) | 53.37 (24.96-106.52) | 0.21 (0.13-0.3) |
| Türkiye | 3061 (1468-6225) | 93.1 (48.3-174.12) | 6309 (3343-11702) | 120.47 (64.1-221.05) | 0.89 (0.7-1.07) |
| Turkmenistan | 3 (2-6) | 38.08 (22.53-64.99) | 5 (3-8) | 39.42 (23.9-67.52) | 0.36 (0.3-0.42) |
| Tuvalu | 48561 (23267-93407) | 88.13 (44.71-162.53) | 81713 (40164-152988) | 92.28 (44.21-175.21) | 0.67 (0.41-0.94) |
| Uganda | 9986 (4468-18630) | 77.6 (39.06-134.41) | 32246 (13584-64247) | 91.15 (44.11-168.06) | 0.88 (0.78-0.97) |
| Ukraine | 45492 (23036-88829) | 81.33 (39.87-160.08) | 68941 (35572-130514) | 152.89 (74.31-291.3) | 2.46 (2.17-2.74) |
| United Arab Emirates | 1738 (909-3173) | 84.43 (45.74-150.21) | 12284 (7444-21199) | 103.23 (56.17-183.61) | 0.89 (0.62-1.17) |
| United Kingdom | 35075 (30796-39991) | 54.69 (48-62.47) | 42132 (36396-49189) | 51.38 (43.98-60.39) | 0.01 (-0.15-0.17) |
| United Republic of Tanzania | 18765 (9031-35480) | 95.18 (50.63-172.45) | 62093 (29323-119816) | 121.57 (60.86-229.79) | 1.12 (0.98-1.25) |
| United States of America | 278273 (234865-330298) | 101.97 (85.8-121.31) | 357029 (307717-418643) | 101.59 (86.37-120.91) | 0.15 (0.09-0.21) |
| United States Virgin Islands | 41 (23-74) | 38.63 (21.65-69.83) | 36 (21-68) | 42.45 (23.01-81.66) | 0.48 (0.37-0.59) |
| Uruguay | 2796 (1325-5443) | 89.78 (41.77-176.06) | 3719 (1810-7127) | 106.88 (49.04-203.99) | 0.63 (0.55-0.71) |
| Uzbekistan | 17139 (9087-31157) | 93.4 (51.4-164.05) | 49016 (26164-89290) | 139.17 (74.02-251.92) | 1.45 (1.16-1.73) |
| Vanuatu | 49 (26-92) | 38.53 (23.72-64.15) | 114 (64-206) | 38.81 (23.12-66.7) | 0.21 (0.16-0.26) |
| Venezuela (Bolivarian Republic of) | 4213 (2020-8510) | 23.18 (12.8-42.33) | 7196 (4045-13092) | 26.29 (14.25-48.95) | 0.58 (0.49-0.67) |
| Viet Nam | 59897 (27263-124072) | 97.46 (46.64-186.76) | 145839 (69769-275431) | 135.5 (63.33-260.66) | 1.54 (1.39-1.69) |
| Yemen | 5478 (2336-12441) | 48.87 (23.55-100.63) | 20345 (8499-45053) | 62.77 (29.22-130.83) | 1.65 (1.3-2) |
| Zambia | 3034 (1468-5742) | 48.47 (26.25-86.98) | 8942 (4284-17277) | 52.09 (27.58-98.6) | 0.68 (0.57-0.78) |
| Zimbabwe | 4210 (2374-7528) | 55.04 (32.58-89.85) | 8944 (4814-16352) | 65.6 (36.96-112.66) | 0.63 (0.52-0.74) |

| **TableS12: National Burden of chronic kidney disease due to diabetes mellitus type 1: deaths, ASDR, and EAPC (1990–2021).** | | | | | |
| --- | --- | --- | --- | --- | --- |
| Country | **1990** | | **2021** | | **EAPC_95%CI** |
|  | Number_95%UI | ASR | Number_95%UI | ASR |  |
| Afghanistan | 77 (46-128) | 1.03 (0.64-1.7) | 160 (82-282) | 0.99 (0.52-1.75) | -0.12 (-0.17--0.07) |
| Albania | 5 (3-7) | 0.21 (0.14-0.31) | 7 (4-10) | 0.17 (0.11-0.25) | -1.35 (-1.99--0.69) |
| Algeria | 56 (36-102) | 0.37 (0.22-0.67) | 193 (129-283) | 0.44 (0.3-0.65) | 0.73 (0.59-0.88) |
| American Samoa | 2 (1-3) | 5.54 (3.7-8.14) | 7 (5-9) | 12.68 (8.99-16.86) | 2.88 (2.63-3.14) |
| Andorra | 0 (0-0) | 0.21 (0.13-0.33) | 0 (0-0) | 0.14 (0.09-0.22) | -0.89 (-1.12--0.67) |
| Angola | 72 (46-106) | 1.33 (0.84-2.01) | 215 (131-326) | 1.27 (0.79-1.93) | -0.26 (-0.38--0.14) |
| Antigua and Barbuda | 1 (1-2) | 2.78 (2.11-3.55) | 4 (3-5) | 3.5 (2.61-4.56) | 1.53 (1.27-1.8) |
| Argentina | 289 (211-400) | 0.89 (0.64-1.22) | 363 (261-508) | 0.69 (0.5-0.96) | -0.72 (-0.96--0.48) |
| Armenia | 1 (1-1) | 0.02 (0.02-0.03) | 7 (4-10) | 0.17 (0.11-0.26) | 6.52 (5.01-8.05) |
| Australia | 12 (10-13) | 0.06 (0.05-0.07) | 23 (16-33) | 0.06 (0.04-0.09) | 1.01 (0.65-1.37) |
| Austria | 15 (10-20) | 0.14 (0.1-0.2) | 20 (16-25) | 0.13 (0.1-0.16) | -0.03 (-0.39-0.35) |
| Azerbaijan | 11 (7-16) | 0.18 (0.12-0.26) | 27 (17-41) | 0.22 (0.14-0.33) | 0.46 (0.23-0.7) |
| Bahamas | 5 (4-6) | 2.63 (2.02-3.35) | 18 (13-24) | 3.87 (2.77-5.19) | 1.79 (1.61-1.98) |
| Bahrain | 1 (1-2) | 0.44 (0.27-0.67) | 7 (4-10) | 0.44 (0.28-0.63) | -0.63 (-0.9--0.37) |
| Bangladesh | 582 (382-828) | 1.02 (0.66-1.47) | 1115 (705-1582) | 0.73 (0.46-1.04) | -0.86 (-1.05--0.67) |
| Barbados | 5 (4-6) | 1.89 (1.47-2.4) | 11 (7-15) | 2.39 (1.66-3.36) | 1.35 (1.08-1.62) |
| Belarus | 9 (7-12) | 0.07 (0.05-0.09) | 36 (25-51) | 0.24 (0.17-0.34) | 3.82 (2.74-4.92) |
| Belgium | 17 (12-24) | 0.12 (0.09-0.17) | 18 (12-25) | 0.09 (0.07-0.13) | -0.63 (-0.94--0.33) |
| Belize | 2 (2-3) | 2.33 (1.81-2.97) | 17 (13-22) | 4.58 (3.48-5.87) | 2.75 (2.29-3.2) |
| Benin | 23 (16-34) | 0.97 (0.65-1.44) | 68 (45-100) | 0.97 (0.62-1.41) | -0.11 (-0.24-0.03) |
| Bermuda | 1 (1-1) | 1.31 (1-1.66) | 1 (1-2) | 1.17 (0.84-1.55) | 0.2 (-0.13-0.53) |
| Bhutan | 4 (2-6) | 1.26 (0.74-1.95) | 8 (5-13) | 1.25 (0.74-1.92) | -0.1 (-0.18--0.02) |
| Bolivia (Plurinational State of) | 107 (75-144) | 2.8 (1.92-3.84) | 330 (212-496) | 3.24 (2.07-4.91) | 0.43 (0.37-0.49) |
| Bosnia and Herzegovina | 22 (16-31) | 0.48 (0.34-0.68) | 27 (18-37) | 0.46 (0.32-0.65) | -0.4 (-0.95-0.16) |
| Botswana | 4 (2-7) | 0.58 (0.35-0.98) | 13 (7-21) | 0.65 (0.38-1.07) | 0.22 (-0.05-0.49) |
| Brazil | 1377 (1073-1771) | 1.26 (0.96-1.62) | 2939 (2192-3833) | 1.13 (0.85-1.47) | -0.56 (-0.75--0.36) |
| Brunei Darussalam | 3 (2-4) | 2.07 (1.46-2.86) | 8 (6-11) | 1.82 (1.34-2.43) | -0.17 (-0.33--0.01) |
| Bulgaria | 21 (15-30) | 0.18 (0.13-0.26) | 49 (32-74) | 0.43 (0.28-0.66) | 3.5 (3.04-3.96) |
| Burkina Faso | 47 (31-69) | 0.89 (0.58-1.32) | 111 (75-158) | 0.91 (0.59-1.34) | 0.1 (0-0.19) |
| Burundi | 56 (38-81) | 2.02 (1.34-3) | 103 (67-150) | 1.55 (0.97-2.29) | -1.33 (-1.51--1.15) |
| C?te d'Ivoire | 1 (1-2) | 0.52 (0.34-0.73) | 4 (2-6) | 0.79 (0.47-1.19) | 1.23 (1.13-1.32) |
| Cabo Verde | 167 (124-226) | 2.52 (1.86-3.44) | 353 (235-512) | 2.24 (1.49-3.24) | -0.55 (-0.69--0.42) |
| Cambodia | 111 (73-160) | 1.89 (1.21-2.76) | 367 (218-572) | 2 (1.2-3.12) | 0.2 (0.02-0.37) |
| Cameroon | 27 (19-37) | 0.08 (0.06-0.11) | 75 (53-105) | 0.13 (0.09-0.17) | 1.83 (1.57-2.08) |
| Canada | 31 (20-44) | 1.98 (1.26-2.9) | 64 (42-102) | 1.95 (1.29-3.09) | -0.11 (-0.17--0.05) |
| Central African Republic | 25 (16-39) | 0.77 (0.49-1.22) | 73 (47-119) | 0.92 (0.58-1.51) | 0.37 (0.15-0.59) |
| Chad | 48 (34-64) | 0.43 (0.31-0.59) | 95 (68-134) | 0.38 (0.27-0.54) | -0.19 (-0.58-0.21) |
| Chile | 19457 (15417-24181) | 1.8 (1.42-2.25) | 20688 (15277-27014) | 1.06 (0.79-1.38) | -1.96 (-2.1--1.82) |
| China | 153 (113-204) | 0.71 (0.52-0.97) | 261 (172-377) | 0.47 (0.31-0.67) | -1.33 (-1.48--1.18) |
| Colombia | 4 (3-6) | 1.71 (1.09-2.49) | 9 (6-14) | 1.61 (1.03-2.31) | -0.5 (-0.71--0.29) |
| Comoros | 30 (18-44) | 2.26 (1.37-3.27) | 79 (45-119) | 2.02 (1.17-2.98) | -0.66 (-0.83--0.49) |
| Congo | 0 (0-1) | 2.55 (1.82-3.53) | 1 (0-1) | 3.17 (2.2-4.35) | 0.94 (0.81-1.08) |
| Cook Islands | 12 (9-16) | 0.63 (0.45-0.86) | 72 (49-99) | 1.29 (0.9-1.78) | 2.32 (1.78-2.87) |
| Costa Rica | 9 (6-13) | 0.15 (0.11-0.21) | 10 (6-15) | 0.13 (0.08-0.2) | -1.48 (-2.06--0.89) |
| Croatia | 92 (75-114) | 0.87 (0.71-1.09) | 250 (178-329) | 1.45 (1.05-1.9) | 1.87 (1.58-2.16) |
| Cuba | 3 (2-5) | 0.37 (0.25-0.57) | 4 (3-6) | 0.21 (0.15-0.31) | -1.95 (-2.08--1.83) |
| Cyprus | 28 (20-40) | 0.22 (0.15-0.31) | 19 (12-29) | 0.1 (0.07-0.16) | -2.38 (-2.54--2.21) |
| Czechia | 53 (35-80) | 0.89 (0.58-1.35) | 153 (95-228) | 0.92 (0.58-1.39) | -0.12 (-0.29-0.05) |
| Democratic People's Republic of Korea | 409 (271-596) | 2.03 (1.36-2.94) | 707 (493-1001) | 2.1 (1.48-2.99) | 0.12 (0.06-0.19) |
| Democratic Republic of the Congo | 332 (223-480) | 1.62 (1.07-2.37) | 843 (528-1274) | 1.65 (1.03-2.52) | -0.05 (-0.15-0.04) |
| Denmark | 10 (7-15) | 0.14 (0.1-0.2) | 22 (17-29) | 0.2 (0.16-0.25) | 0.62 (-0.16-1.41) |
| Djibouti | 3 (2-4) | 1.35 (0.85-2.06) | 16 (10-24) | 1.81 (1.14-2.75) | 0.91 (0.72-1.09) |
| Dominica | 2 (1-2) | 2.69 (1.95-3.49) | 4 (3-5) | 4.46 (3.13-6.28) | 1.99 (1.86-2.11) |
| Dominican Republic | 66 (50-87) | 1.38 (1.02-1.89) | 243 (149-339) | 2.25 (1.38-3.14) | 2.39 (2.17-2.6) |
| Ecuador | 89 (69-114) | 1.41 (1.06-1.84) | 408 (242-678) | 2.4 (1.42-3.98) | 1.49 (0.55-2.44) |
| Egypt | 221 (146-354) | 0.6 (0.39-0.96) | 709 (463-1068) | 0.85 (0.55-1.28) | 1.32 (1.22-1.42) |
| El Salvador | 41 (29-63) | 1.24 (0.86-1.92) | 218 (137-318) | 3.63 (2.28-5.28) | 3.6 (3.17-4.03) |
| Equatorial Guinea | 4 (3-6) | 1.78 (1.14-2.59) | 15 (8-24) | 1.91 (0.98-3.16) | 0.43 (-0.03-0.9) |
| Eritrea | 33 (20-53) | 1.91 (1.15-3.07) | 73 (45-119) | 1.83 (1.09-3.04) | -0.17 (-0.24--0.1) |
| Estonia | 13 (9-18) | 0.65 (0.49-0.87) | 22 (17-29) | 0.97 (0.75-1.25) | 0.25 (-0.18-0.69) |
| Eswatini | 5 (3-7) | 1.19 (0.74-1.83) | 15 (7-23) | 1.9 (0.95-2.98) | 1.74 (0.89-2.6) |
| Ethiopia | 1554 (1076-2005) | 5.7 (3.97-7.48) | 1630 (1226-2139) | 2.68 (1.9-3.61) | -2.96 (-3.14--2.79) |
| Fiji | 26 (17-37) | 4.72 (3.15-6.9) | 68 (47-96) | 7.3 (5.09-10.29) | 1.08 (0.82-1.35) |
| Finland | 7 (5-9) | 0.1 (0.08-0.14) | 12 (10-16) | 0.12 (0.1-0.14) | 1.01 (0.07-1.96) |
| France | 81 (58-114) | 0.11 (0.08-0.15) | 106 (78-140) | 0.09 (0.07-0.12) | -0.13 (-0.42-0.16) |
| Gabon | 12 (8-17) | 1.85 (1.21-2.76) | 33 (16-50) | 2.48 (1.18-3.75) | 0.88 (0.67-1.09) |
| Gambia | 4 (3-6) | 0.92 (0.57-1.36) | 15 (10-22) | 1.16 (0.72-1.68) | 0.47 (0.26-0.67) |
| Georgia | 7 (5-10) | 0.12 (0.08-0.18) | 12 (8-19) | 0.27 (0.18-0.4) | 2.7 (2.13-3.27) |
| Germany | 178 (125-249) | 0.15 (0.11-0.22) | 189 (128-272) | 0.11 (0.08-0.16) | 0.05 (-0.56-0.67) |
| Ghana | 82 (54-124) | 0.98 (0.64-1.51) | 355 (236-523) | 1.57 (1.04-2.27) | 2.08 (1.84-2.33) |
| Greece | 36 (26-51) | 0.24 (0.18-0.34) | 40 (31-51) | 0.22 (0.17-0.27) | -2.23 (-3.67--0.77) |
| Greenland | 0 (0-0) | 0.21 (0.14-0.3) | 0 (0-0) | 0.22 (0.15-0.33) | 0.76 (0.59-0.92) |
| Grenada | 2 (2-3) | 3.88 (2.91-4.9) | 7 (5-9) | 5.32 (4-6.94) | 1.74 (1.48-2) |
| Guam | 2 (2-3) | 2.05 (1.55-2.81) | 8 (6-10) | 4.29 (3.33-5.38) | 3.25 (2.94-3.56) |
| Guatemala | 54 (40-72) | 1.21 (0.89-1.64) | 287 (194-400) | 2.35 (1.56-3.28) | 3.07 (2.57-3.57) |
| Guinea | 23 (14-36) | 0.6 (0.37-0.97) | 46 (29-72) | 0.63 (0.4-1.02) | 0.18 (0.1-0.27) |
| Guinea-Bissau | 8 (6-12) | 1.62 (1.05-2.38) | 16 (10-23) | 1.41 (0.89-2.17) | -0.52 (-0.58--0.46) |
| Guyana | 15 (11-19) | 3.02 (2.26-3.95) | 46 (30-65) | 6.08 (4.11-8.63) | 3.37 (2.99-3.75) |
| Haiti | 121 (79-200) | 2.88 (1.83-4.88) | 288 (153-612) | 2.95 (1.55-6.54) | 0.36 (0.26-0.46) |
| Honduras | 14 (10-20) | 0.58 (0.39-0.83) | 73 (45-113) | 1 (0.61-1.56) | 1.97 (1.77-2.17) |
| Hungary | 28 (20-40) | 0.21 (0.15-0.28) | 31 (21-46) | 0.18 (0.12-0.27) | -0.55 (-1.36-0.27) |
| Iceland | 0 (0-0) | 0.05 (0.03-0.07) | 0 (0-0) | 0.07 (0.05-0.08) | 1.99 (1.66-2.32) |
| India | 5716 (4063-7551) | 0.97 (0.69-1.3) | 15464 (10796-21436) | 1.15 (0.8-1.6) | 0.49 (0.37-0.61) |
| Indonesia | 3125 (2359-3981) | 2.13 (1.63-2.74) | 7564 (5597-10260) | 2.45 (1.82-3.31) | 0.58 (0.51-0.65) |
| Iran (Islamic Republic of) | 109 (71-162) | 0.32 (0.2-0.48) | 283 (199-389) | 0.3 (0.21-0.41) | -0.04 (-0.14-0.06) |
| Iraq | 84 (52-128) | 0.84 (0.51-1.28) | 237 (120-386) | 0.73 (0.37-1.17) | -0.64 (-0.71--0.57) |
| Ireland | 6 (4-8) | 0.14 (0.1-0.2) | 8 (6-12) | 0.11 (0.08-0.16) | -0.14 (-0.31-0.02) |
| Israel | 23 (16-32) | 0.48 (0.35-0.65) | 47 (33-65) | 0.42 (0.3-0.57) | 1.34 (0.29-2.39) |
| Italy | 133 (93-189) | 0.16 (0.11-0.22) | 124 (84-185) | 0.1 (0.07-0.15) | -1.79 (-1.99--1.59) |
| Jamaica | 29 (22-38) | 1.71 (1.3-2.2) | 86 (57-125) | 2.78 (1.86-4.03) | 1.14 (0.48-1.81) |
| Japan | 632 (478-807) | 0.37 (0.28-0.48) | 611 (444-829) | 0.26 (0.19-0.33) | -1.44 (-1.63--1.25) |
| Jordan | 15 (10-21) | 0.75 (0.5-1.09) | 70 (47-100) | 0.66 (0.45-0.93) | -0.69 (-1.06--0.32) |
| Kazakhstan | 28 (20-40) | 0.19 (0.13-0.27) | 47 (31-67) | 0.23 (0.15-0.33) | -0.3 (-0.88-0.28) |
| Kenya | 112 (76-174) | 1.07 (0.71-1.65) | 504 (339-734) | 1.65 (1.08-2.44) | 1.68 (1.49-1.87) |
| Kiribati | 3 (2-4) | 5.72 (4.31-7.37) | 8 (5-13) | 7.84 (4.58-12.4) | 0.8 (0.5-1.1) |
| Kuwait | 6 (4-8) | 0.53 (0.37-0.73) | 10 (6-15) | 0.2 (0.12-0.28) | -2.95 (-3.16--2.73) |
| Kyrgyzstan | 6 (4-9) | 0.18 (0.12-0.25) | 16 (11-23) | 0.25 (0.17-0.36) | -0.42 (-1.31-0.47) |
| Lao People's Democratic Republic | 141 (98-203) | 5.14 (3.52-7.48) | 258 (169-385) | 4.07 (2.63-6.05) | -0.92 (-0.99--0.85) |
| Latvia | 9 (7-12) | 0.27 (0.2-0.36) | 18 (13-26) | 0.56 (0.39-0.8) | 1.84 (1.53-2.15) |
| Lebanon | 12 (7-19) | 0.49 (0.3-0.75) | 21 (14-30) | 0.35 (0.23-0.52) | -0.99 (-1.15--0.83) |
| Lesotho | 4 (2-6) | 0.39 (0.24-0.59) | 14 (8-21) | 1.03 (0.57-1.61) | 4 (3.37-4.63) |
| Liberia | 17 (11-26) | 1.2 (0.79-1.86) | 42 (26-62) | 1.29 (0.8-1.91) | 0.45 (0.14-0.77) |
| Libya | 10 (6-15) | 0.42 (0.26-0.63) | 51 (26-80) | 0.69 (0.35-1.05) | 2.17 (2.01-2.32) |
| Lithuania | 9 (7-12) | 0.21 (0.15-0.28) | 21 (15-31) | 0.45 (0.32-0.66) | 1.2 (0.72-1.69) |
| Luxembourg | 1 (1-1) | 0.18 (0.13-0.25) | 2 (1-2) | 0.16 (0.11-0.23) | 0.08 (-0.11-0.28) |
| Madagascar | 82 (54-121) | 1.3 (0.85-1.98) | 205 (131-311) | 1.27 (0.81-1.95) | -0.06 (-0.14-0.02) |
| Malawi | 85 (58-123) | 1.73 (1.16-2.49) | 205 (141-284) | 2.05 (1.35-2.93) | 0.3 (0.03-0.58) |
| Malaysia | 283 (221-353) | 2.29 (1.73-2.9) | 847 (624-1093) | 2.61 (1.91-3.38) | 0.32 (0.14-0.5) |
| Maldives | 6 (4-8) | 4.75 (3.4-6.29) | 10 (7-14) | 1.9 (1.39-2.58) | -3.22 (-3.45--2.99) |
| Mali | 54 (36-80) | 1.06 (0.69-1.58) | 114 (74-177) | 0.96 (0.61-1.52) | -0.25 (-0.39--0.12) |
| Malta | 1 (1-1) | 0.22 (0.15-0.31) | 2 (1-3) | 0.23 (0.16-0.32) | 0.3 (0.05-0.54) |
| Marshall Islands | 1 (1-2) | 5.87 (3.44-9.91) | 5 (2-14) | 10.3 (3.23-26.91) | 1.78 (1.51-2.06) |
| Mauritania | 13 (8-20) | 1.16 (0.71-1.78) | 25 (15-41) | 0.96 (0.55-1.58) | -0.97 (-1.13--0.81) |
| Mauritius | 42 (33-52) | 4.6 (3.57-5.8) | 155 (120-198) | 8.89 (7.04-11.06) | 2.38 (1.96-2.79) |
| Mexico | 682 (514-901) | 1.32 (0.97-1.78) | 3933 (2726-5534) | 2.85 (1.99-4) | 2.89 (2.13-3.65) |
| Micronesia (Federated States of) | 4 (2-6) | 6.01 (3.82-9.25) | 10 (7-15) | 10.31 (6.95-15.04) | 1.73 (1.32-2.14) |
| Monaco | 0 (0-0) | 0.12 (0.08-0.17) | 0 (0-0) | 0.16 (0.11-0.23) | 1.24 (0.97-1.51) |
| Mongolia | 4 (3-6) | 0.33 (0.22-0.5) | 9 (6-14) | 0.27 (0.18-0.41) | -1.01 (-1.28--0.74) |
| Montenegro | 2 (1-3) | 0.31 (0.21-0.45) | 3 (2-5) | 0.35 (0.23-0.52) | 1.19 (0.53-1.86) |
| Morocco | 49 (29-79) | 0.29 (0.17-0.47) | 169 (105-265) | 0.44 (0.27-0.68) | 1.79 (1.62-1.96) |
| Mozambique | 86 (57-133) | 1.12 (0.73-1.76) | 259 (169-389) | 1.64 (1.05-2.52) | 1.79 (1.59-1.99) |
| Myanmar | 1131 (777-1552) | 3.53 (2.41-4.94) | 1527 (1079-2099) | 2.67 (1.89-3.66) | -1.21 (-1.33--1.08) |
| Namibia | 4 (3-7) | 0.55 (0.34-0.93) | 12 (7-18) | 0.66 (0.4-1.03) | 0.26 (-0.16-0.68) |
| Nauru | 1 (0-1) | 7.35 (4.42-10.96) | 1 (1-1) | 11.44 (7.53-15.98) | 1.27 (1.1-1.45) |
| Nepal | 116 (74-172) | 0.98 (0.62-1.45) | 336 (206-506) | 1.28 (0.78-1.93) | 1.09 (0.69-1.48) |
| Netherlands | 17 (12-24) | 0.09 (0.07-0.13) | 27 (21-34) | 0.09 (0.07-0.11) | -1.68 (-2.36--1.01) |
| New Zealand | 4 (3-6) | 0.11 (0.07-0.15) | 9 (6-13) | 0.13 (0.09-0.18) | 1.24 (0.88-1.61) |
| Nicaragua | 26 (18-38) | 1.36 (0.94-2.02) | 162 (108-226) | 2.87 (1.89-4.05) | 2.99 (2.53-3.46) |
| Niger | 23 (15-35) | 0.59 (0.37-0.93) | 52 (32-93) | 0.47 (0.29-0.84) | -0.82 (-0.98--0.67) |
| Nigeria | 362 (236-542) | 0.67 (0.44-1.03) | 746 (431-1154) | 0.58 (0.33-0.9) | -1 (-1.26--0.75) |
| Niue | 0 (0-0) | 4.76 (3.23-6.71) | 0 (0-0) | 8.82 (4.86-14.11) | 1.69 (1.51-1.87) |
| North Macedonia | 4 (2-5) | 0.17 (0.11-0.25) | 6 (4-10) | 0.18 (0.11-0.3) | -0.83 (-1.37--0.28) |
| Northern Mariana Islands | 2 (1-3) | 5.92 (3.95-8.35) | 6 (4-7) | 8.82 (6.64-11.08) | 1.46 (1.25-1.67) |
| Norway | 4 (3-6) | 0.07 (0.05-0.09) | 7 (5-10) | 0.08 (0.05-0.11) | -1.18 (-1.87--0.49) |
| Oman | 4 (2-7) | 0.38 (0.22-0.64) | 19 (12-28) | 0.53 (0.33-0.87) | 1.54 (1.34-1.75) |
| Pakistan | 835 (559-1187) | 1.27 (0.84-1.82) | 2934 (1888-4073) | 1.8 (1.15-2.52) | 0.89 (0.64-1.15) |
| Palau | 1 (0-1) | 4.79 (3.05-6.73) | 2 (1-3) | 8.03 (5.11-11.43) | 1.79 (1.58-2.01) |
| Palestine | 6 (4-9) | 0.56 (0.34-0.86) | 17 (12-26) | 0.5 (0.33-0.75) | -0.41 (-0.56--0.26) |
| Panama | 9 (7-12) | 0.54 (0.39-0.74) | 54 (36-80) | 1.22 (0.82-1.81) | 2.79 (2.33-3.25) |
| Papua New Guinea | 65 (37-96) | 2.31 (1.34-3.38) | 216 (157-289) | 2.62 (1.88-3.54) | 0.35 (0.28-0.41) |
| Paraguay | 24 (17-33) | 0.96 (0.66-1.33) | 93 (60-138) | 1.47 (0.93-2.18) | 1.36 (1.17-1.54) |
| Peru | 220 (160-290) | 1.58 (1.14-2.11) | 593 (365-890) | 1.69 (1.03-2.54) | -0.01 (-0.23-0.21) |
| Philippines | 1295 (1051-1587) | 2.89 (2.32-3.61) | 4612 (3467-6061) | 4.42 (3.31-5.82) | 1.86 (1.68-2.05) |
| Poland | 157 (114-218) | 0.36 (0.26-0.5) | 90 (60-137) | 0.14 (0.1-0.22) | -3.34 (-3.86--2.82) |
| Portugal | 40 (28-55) | 0.3 (0.21-0.41) | 47 (33-67) | 0.23 (0.17-0.31) | -0.87 (-1.33--0.4) |
| Puerto Rico | 87 (66-109) | 2.42 (1.83-3.06) | 142 (102-188) | 2.82 (2.1-3.68) | 1.18 (0.75-1.61) |
| Qatar | 1 (1-2) | 0.41 (0.21-0.68) | 8 (5-12) | 0.34 (0.21-0.53) | -0.97 (-1.33--0.61) |
| Republic of Korea | 304 (232-383) | 0.8 (0.61-1.02) | 309 (219-416) | 0.35 (0.25-0.46) | -2.32 (-2.49--2.14) |
| Republic of Moldova | 7 (6-10) | 0.16 (0.12-0.21) | 20 (14-27) | 0.34 (0.24-0.48) | 1.62 (1.16-2.09) |
| Romania | 25 (17-36) | 0.09 (0.06-0.13) | 41 (29-56) | 0.13 (0.09-0.18) | 1.97 (1.44-2.5) |
| Russian Federation | 1196 (927-1537) | 0.66 (0.52-0.85) | 1431 (1025-1990) | 0.62 (0.45-0.84) | -1.16 (-1.52--0.79) |
| Rwanda | 76 (54-107) | 2.12 (1.45-3.04) | 107 (68-159) | 1.3 (0.81-1.97) | -2.69 (-3.12--2.25) |
| Saint Kitts and Nevis | 1 (1-2) | 4.11 (3.2-5.26) | 4 (3-5) | 4.48 (3.2-6.19) | 0.95 (0.58-1.33) |
| Saint Lucia | 3 (2-4) | 3.21 (2.46-4.09) | 9 (6-12) | 3.7 (2.72-5.03) | 1.08 (0.82-1.34) |
| Saint Vincent and the Grenadines | 2 (1-2) | 2.4 (1.84-3.04) | 5 (4-7) | 3.87 (2.91-5.01) | 2.21 (1.91-2.52) |
| Samoa | 5 (3-7) | 4.75 (3.07-6.92) | 12 (8-17) | 7.13 (4.95-9.93) | 1.28 (1.19-1.37) |
| San Marino | 0 (0-0) | 0.08 (0.05-0.12) | 0 (0-0) | 0.06 (0.03-0.1) | 0.34 (-0.03-0.72) |
| Sao Tome and Principe | 1 (1-1) | 1.47 (0.99-2.04) | 3 (2-4) | 1.97 (1.2-2.89) | 0.74 (0.5-0.98) |
| Saudi Arabia | 79 (49-121) | 0.9 (0.54-1.4) | 643 (378-1001) | 1.64 (0.97-2.42) | 1.83 (1.67-1.99) |
| Senegal | 52 (35-77) | 1.28 (0.85-1.93) | 124 (82-198) | 1.27 (0.84-2.02) | -0.06 (-0.18-0.05) |
| Serbia | 48 (32-71) | 0.4 (0.27-0.58) | 54 (36-77) | 0.38 (0.26-0.52) | -0.26 (-0.44--0.07) |
| Seychelles | 2 (1-2) | 3.29 (2.47-4.3) | 6 (4-7) | 4.37 (3.26-5.7) | 1.13 (0.94-1.31) |
| Sierra Leone | 18 (11-27) | 0.77 (0.46-1.15) | 39 (24-59) | 0.77 (0.47-1.17) | 0.1 (0.04-0.16) |
| Singapore | 22 (17-28) | 0.84 (0.64-1.07) | 33 (24-44) | 0.38 (0.28-0.51) | -0.35 (-1.04-0.36) |
| Slovakia | 20 (14-29) | 0.35 (0.24-0.5) | 19 (13-29) | 0.22 (0.15-0.33) | -1.42 (-1.63--1.21) |
| Slovenia | 2 (2-3) | 0.1 (0.07-0.13) | 2 (1-3) | 0.06 (0.04-0.09) | -1.81 (-2.11--1.5) |
| Solomon Islands | 10 (3-17) | 4.86 (1.65-8.4) | 31 (20-42) | 5.67 (3.84-7.69) | 0.47 (0.25-0.68) |
| Somalia | 87 (54-132) | 2.41 (1.49-3.75) | 230 (141-384) | 2.53 (1.54-4.29) | 0.18 (0.08-0.28) |
| South Africa | 167 (122-233) | 0.64 (0.46-0.92) | 447 (312-629) | 0.81 (0.56-1.13) | 1.41 (1.03-1.78) |
| South Sudan | 57 (37-87) | 1.93 (1.21-2.92) | 141 (88-205) | 2.65 (1.59-3.95) | 0.92 (0.64-1.2) |
| Spain | 125 (87-176) | 0.24 (0.17-0.33) | 106 (75-150) | 0.13 (0.09-0.18) | -2.03 (-2.29--1.78) |
| Sri Lanka | 296 (219-390) | 2.1 (1.53-2.8) | 481 (293-719) | 1.82 (1.12-2.69) | -0.65 (-0.89--0.4) |
| Sudan | 30 (18-55) | 0.26 (0.15-0.48) | 93 (55-152) | 0.33 (0.19-0.54) | 0.72 (0.53-0.9) |
| Suriname | 9 (7-12) | 2.97 (2.21-3.81) | 30 (20-42) | 4.52 (3.11-6.27) | 1.55 (1.35-1.76) |
| Sweden | 12 (8-17) | 0.09 (0.06-0.12) | 25 (17-36) | 0.13 (0.09-0.19) | 1.81 (1.25-2.37) |
| Switzerland | 7 (5-10) | 0.08 (0.06-0.11) | 10 (7-13) | 0.06 (0.05-0.08) | -2 (-2.74--1.26) |
| Syrian Arab Republic | 47 (31-71) | 0.67 (0.44-1.03) | 99 (61-153) | 0.64 (0.41-0.96) | -0.61 (-0.98--0.24) |
| Taiwan (Province of China) | 352 (294-428) | 1.85 (1.52-2.28) | 497 (383-628) | 1.33 (1.06-1.63) | -0.35 (-0.69--0.02) |
| Tajikistan | 2 (1-3) | 0.05 (0.04-0.08) | 5 (3-8) | 0.06 (0.03-0.09) | -0.48 (-0.79--0.17) |
| Thailand | 1075 (805-1477) | 2.21 (1.65-3.06) | 2558 (1772-3584) | 2.65 (1.88-3.71) | 0.02 (-0.3-0.34) |
| Timor-Leste | 12 (8-17) | 2.42 (1.59-3.6) | 25 (16-37) | 2.51 (1.63-3.76) | 0.26 (-0.06-0.59) |
| Togo | 16 (10-23) | 0.9 (0.59-1.35) | 55 (35-84) | 1 (0.63-1.58) | 0.25 (0.15-0.36) |
| Tokelau | 0 (0-0) | 4.07 (2.55-7.22) | 0 (0-0) | 6.23 (4.47-8.72) | 1.16 (1.03-1.28) |
| Tonga | 2 (1-2) | 2.39 (1.56-3.69) | 3 (2-4) | 3.19 (2.05-4.54) | 0.57 (0.34-0.81) |
| Trinidad and Tobago | 21 (17-26) | 2.24 (1.75-2.81) | 77 (49-114) | 4.13 (2.71-5.99) | 2.42 (2.07-2.78) |
| Tunisia | 16 (10-26) | 0.28 (0.17-0.45) | 54 (33-87) | 0.38 (0.23-0.61) | 1.01 (0.85-1.18) |
| Türkiye | 7 (5-10) | 0.29 (0.21-0.4) | 25 (17-38) | 0.49 (0.32-0.74) | 1.61 (1.19-2.03) |
| Turkmenistan | 0 (0-1) | 5.11 (3.58-7.9) | 1 (1-1) | 7.11 (4.87-10.32) | 1.13 (1.05-1.22) |
| Tuvalu | 192 (126-285) | 0.45 (0.29-0.68) | 351 (229-542) | 0.36 (0.23-0.55) | -0.82 (-1.06--0.57) |
| Uganda | 115 (74-172) | 1.46 (0.93-2.23) | 354 (230-509) | 1.77 (1.12-2.58) | 0.09 (-0.16-0.34) |
| Ukraine | 8 (6-11) | 0.01 (0.01-0.02) | 182 (117-257) | 0.27 (0.18-0.39) | 13.78 (11.66-15.94) |
| United Arab Emirates | 4 (2-6) | 0.38 (0.21-0.62) | 33 (17-52) | 0.37 (0.2-0.57) | 0.88 (0.41-1.35) |
| United Kingdom | 95 (68-133) | 0.12 (0.08-0.16) | 140 (103-189) | 0.13 (0.1-0.17) | 0.92 (0.55-1.28) |
| United Republic of Tanzania | 94 (62-141) | 0.72 (0.47-1.08) | 238 (156-367) | 0.73 (0.47-1.12) | -0.21 (-0.31--0.12) |
| United States of America | 662 (494-863) | 0.23 (0.17-0.3) | 3116 (2298-4118) | 0.63 (0.47-0.8) | 3.55 (3.18-3.93) |
| United States Virgin Islands | 2 (1-3) | 1.91 (1.38-2.5) | 3 (2-4) | 2.35 (1.59-3.29) | 1.29 (1.09-1.49) |
| Uruguay | 12 (9-16) | 0.33 (0.25-0.43) | 17 (12-24) | 0.38 (0.26-0.52) | 1.02 (0.69-1.36) |
| Uzbekistan | 13 (9-19) | 0.09 (0.07-0.14) | 105 (72-152) | 0.3 (0.21-0.44) | 2.43 (1.55-3.31) |
| Vanuatu | 4 (2-6) | 3.85 (2.13-6.65) | 15 (10-24) | 6.29 (4.14-9.68) | 1.55 (1.47-1.62) |
| Venezuela (Bolivarian Republic of) | 80 (58-109) | 0.69 (0.5-0.94) | 533 (325-790) | 1.67 (1.02-2.46) | 2.26 (1.72-2.8) |
| Viet Nam | 998 (648-1384) | 2.11 (1.38-2.99) | 2338 (1530-3244) | 2.06 (1.34-2.83) | 0.17 (-0.02-0.37) |
| Yemen | 23 (13-45) | 0.35 (0.19-0.67) | 60 (33-108) | 0.3 (0.17-0.53) | -0.67 (-0.79--0.56) |
| Zambia | 84 (57-117) | 2.24 (1.49-3.18) | 266 (162-447) | 2.64 (1.61-4.44) | 0.16 (0-0.33) |
| Zimbabwe | 28 (18-46) | 0.56 (0.36-0.92) | 102 (65-153) | 1.07 (0.67-1.63) | 2.47 (1.79-3.14) |

| **TableS13: National Burden of chronic kidney disease due to diabetes mellitus type 1: DALYs cases, ASDAR, and EAPC (1990–2021).** | | | | | |
| --- | --- | --- | --- | --- | --- |
| Country | **1990** | | **2021** | | **EAPC_95%CI** |
|  | Number_95%UI | ASR | Number_95%UI | ASR |  |
| Afghanistan | 3062 (1898-5002) | 41.93 (26.09-68.18) | 7127 (3811-12202) | 39.22 (21.02-68.6) | -0.22 (-0.28--0.16) |
| Albania | 231 (168-322) | 8.8 (6.43-12.54) | 283 (197-403) | 8.04 (5.62-11.27) | -0.72 (-1.21--0.23) |
| Algeria | 2547 (1664-4200) | 15.49 (10.03-27.04) | 8177 (5678-11785) | 18.03 (12.52-25.98) | 0.65 (0.52-0.78) |
| American Samoa | 82 (57-116) | 227.88 (155.17-324.51) | 275 (199-365) | 524.72 (381.25-695.37) | 2.9 (2.64-3.16) |
| Andorra | 6 (4-8) | 9.43 (6.53-13.07) | 13 (8-17) | 8.9 (6.04-12.12) | 0.03 (-0.09-0.15) |
| Angola | 2965 (1963-4242) | 49.15 (31.62-71.83) | 8866 (5667-13243) | 46.8 (28.77-70.44) | -0.23 (-0.35--0.11) |
| Antigua and Barbuda | 53 (43-66) | 106.3 (84.02-133.8) | 144 (110-186) | 126.58 (98.45-161.75) | 1.48 (1.21-1.75) |
| Argentina | 10907 (8074-14475) | 33.81 (25.11-44.96) | 13507 (10084-18111) | 26.23 (19.46-34.95) | -0.65 (-0.85--0.44) |
| Armenia | 102 (68-149) | 3.17 (2.1-4.65) | 362 (248-514) | 9.83 (6.72-13.81) | 4 (3.23-4.78) |
| Australia | 683 (530-936) | 3.66 (2.82-5.01) | 1772 (1205-2537) | 5.4 (3.69-7.78) | 1.53 (1.33-1.72) |
| Austria | 775 (555-1029) | 8.09 (5.76-10.69) | 1239 (888-1724) | 9.3 (6.63-12.95) | 0.61 (0.41-0.81) |
| Azerbaijan | 662 (465-915) | 10.57 (7.44-14.55) | 1574 (1111-2306) | 12.65 (9.01-18.24) | 0.45 (0.27-0.63) |
| Bahamas | 215 (174-267) | 104.31 (83.46-131.79) | 706 (511-922) | 153.89 (111.88-200.88) | 1.76 (1.59-1.93) |
| Bahrain | 58 (39-84) | 16.63 (10.69-25.02) | 299 (204-415) | 16.97 (11.5-23.57) | -0.49 (-0.72--0.25) |
| Bangladesh | 24002 (16508-32729) | 38.64 (25.65-54.39) | 42864 (28010-60462) | 27.62 (17.97-38.88) | -0.84 (-1.04--0.63) |
| Barbados | 181 (148-224) | 75.47 (60.61-94.27) | 379 (265-534) | 92.15 (65.22-127.29) | 1.2 (0.94-1.45) |
| Belarus | 486 (367-651) | 3.95 (2.96-5.27) | 1448 (1046-1991) | 10.32 (7.45-14.22) | 3.29 (2.47-4.11) |
| Belgium | 851 (619-1145) | 6.63 (4.85-8.8) | 1148 (828-1556) | 7.05 (5.11-9.57) | 0.44 (0.24-0.65) |
| Belize | 105 (87-130) | 90.87 (72.55-115.19) | 697 (552-873) | 178.19 (139.79-225.44) | 2.74 (2.3-3.18) |
| Benin | 956 (667-1331) | 36.46 (25-52.74) | 2892 (1937-4180) | 36.44 (23.64-53.66) | -0.1 (-0.24-0.03) |
| Bermuda | 34 (27-43) | 51.11 (40.63-64.4) | 45 (33-59) | 46.87 (35.58-60.7) | 0.25 (-0.06-0.56) |
| Bhutan | 161 (99-245) | 46.79 (28.23-70.89) | 320 (201-490) | 45.75 (28.21-69.04) | -0.15 (-0.23--0.08) |
| Bolivia (Plurinational State of) | 4142 (2895-5617) | 99.92 (68.84-136.58) | 11542 (7615-17073) | 109.53 (71.39-163.07) | 0.21 (0.16-0.27) |
| Bosnia and Herzegovina | 911 (666-1224) | 18.84 (13.97-25.06) | 947 (669-1298) | 18.14 (12.97-24.66) | -0.34 (-0.74-0.06) |
| Botswana | 176 (109-291) | 23.49 (14.4-38.51) | 544 (328-860) | 25.55 (15.26-40.67) | 0.18 (-0.07-0.44) |
| Brazil | 58928 (47202-74801) | 50.92 (40.47-65.28) | 113391 (87886-143895) | 43.74 (34.09-55.45) | -0.67 (-0.84--0.51) |
| Brunei Darussalam | 142 (108-182) | 82.07 (60.84-107.21) | 373 (282-478) | 74.19 (55.9-95.67) | -0.14 (-0.3-0.02) |
| Bulgaria | 936 (699-1258) | 8.49 (6.39-11.26) | 1808 (1236-2626) | 17.8 (12.35-25.5) | 3.08 (2.7-3.46) |
| Burkina Faso | 1896 (1299-2736) | 33.82 (22.98-49.54) | 4655 (3201-6560) | 34.54 (23.13-48.77) | 0.1 (0-0.19) |
| Burundi | 2228 (1517-3089) | 73.12 (49.3-105.13) | 4208 (2812-6082) | 55.49 (35.86-80.33) | -1.35 (-1.54--1.17) |
| C?te d'Ivoire | 46 (32-61) | 21.14 (14.58-29.03) | 159 (101-235) | 29.18 (18.46-43.41) | 0.99 (0.95-1.03) |
| Cabo Verde | 8123 (5894-10887) | 111.66 (82.52-150.87) | 15773 (10658-22726) | 96.03 (64.93-138.56) | -0.66 (-0.79--0.53) |
| Cambodia | 4674 (3130-6823) | 72.96 (48.61-105.68) | 16012 (10021-24803) | 77.51 (46.69-120.15) | 0.18 (0.02-0.34) |
| Cameroon | 1980 (1352-2873) | 6.35 (4.35-9.18) | 4609 (3277-6139) | 9.36 (6.55-12.83) | 1.7 (1.55-1.85) |
| Canada | 1219 (816-1725) | 73.11 (48.34-105.67) | 2645 (1757-4232) | 72.76 (48.15-115) | -0.08 (-0.15--0.02) |
| Central African Republic | 1024 (685-1525) | 29.39 (19.48-45.31) | 3094 (2057-4959) | 34.43 (22.37-56.51) | 0.36 (0.15-0.57) |
| Chad | 2086 (1602-2714) | 18.2 (13.63-23.69) | 3743 (2763-5077) | 15.78 (11.9-21.29) | -0.08 (-0.4-0.23) |
| Chile | 916754 (739349-1115671) | 80.92 (65.12-98.39) | 886025 (666773-1127677) | 47.95 (36.9-60.73) | -1.96 (-2.11--1.81) |
| China | 6219 (4716-8176) | 26.63 (19.86-35.2) | 9612 (6821-13354) | 17.43 (12.45-24.03) | -1.24 (-1.39--1.09) |
| Colombia | 165 (106-238) | 61.7 (40.19-89.19) | 360 (240-516) | 57.48 (38.44-81.64) | -0.57 (-0.81--0.32) |
| Comoros | 1202 (757-1718) | 84.4 (52.41-121.46) | 3217 (1951-4864) | 75.36 (43.62-112.68) | -0.66 (-0.84--0.48) |
| Congo | 16 (12-22) | 105.28 (76.06-144.04) | 28 (20-38) | 132.09 (95.47-178.93) | 1 (0.85-1.15) |
| Cook Islands | 474 (360-631) | 22.92 (16.75-30.92) | 2536 (1798-3474) | 46.14 (32.92-62.96) | 2.29 (1.78-2.8) |
| Costa Rica | 412 (303-557) | 6.79 (5.04-9.02) | 419 (289-595) | 6.67 (4.61-9.31) | -0.78 (-1.15--0.4) |
| Croatia | 3915 (3264-4761) | 36.71 (30.33-44.9) | 8900 (6506-11520) | 54.88 (41.76-70.88) | 1.52 (1.28-1.77) |
| Cuba | 104 (74-150) | 12.72 (9.1-18.41) | 207 (148-292) | 11.01 (7.89-15.54) | -0.43 (-0.54--0.32) |
| Cyprus | 1173 (860-1580) | 9.46 (6.86-12.84) | 892 (633-1231) | 5.75 (4.09-7.95) | -1.38 (-1.51--1.25) |
| Czechia | 2314 (1548-3402) | 34.04 (22.72-50.75) | 6601 (4161-9814) | 35.26 (22.31-52.57) | -0.07 (-0.24-0.09) |
| Democratic People's Republic of Korea | 19152 (13026-27449) | 92.58 (63.31-132.15) | 31241 (22515-43259) | 94.68 (68.4-131.83) | 0.05 (0-0.09) |
| Democratic Republic of the Congo | 13310 (9397-18784) | 59.73 (40.81-86.74) | 34290 (22505-52028) | 60.67 (38.86-90.55) | -0.05 (-0.14-0.05) |
| Denmark | 545 (399-745) | 8.25 (6.03-11.25) | 971 (732-1331) | 10.84 (8.06-15.32) | 0.78 (0.23-1.32) |
| Djibouti | 111 (75-163) | 48.48 (31.8-72.33) | 647 (417-941) | 64.02 (40.91-94.37) | 0.87 (0.67-1.06) |
| Dominica | 57 (43-71) | 100.02 (74.94-126.3) | 137 (97-191) | 169.84 (121.65-232.59) | 2.1 (1.96-2.24) |
| Dominican Republic | 2984 (2321-3795) | 57.21 (43.87-74.65) | 9927 (6255-13544) | 90.04 (56.7-123.61) | 2.29 (2.06-2.52) |
| Ecuador | 3633 (2897-4573) | 52.87 (41.62-67.48) | 14222 (8673-23760) | 82.59 (50.13-138.84) | 1.15 (0.21-2.11) |
| Egypt | 9739 (6733-14991) | 24.37 (16.68-38.3) | 28869 (19817-41687) | 32.15 (22.08-47.31) | 1.09 (1-1.19) |
| El Salvador | 1638 (1189-2425) | 47.11 (33.36-71.01) | 8093 (5204-11662) | 133.94 (85.79-193) | 3.58 (3.17-4) |
| Equatorial Guinea | 172 (114-248) | 65.74 (42.91-95.65) | 629 (341-1025) | 70.46 (37.25-114.52) | 0.43 (-0.02-0.88) |
| Eritrea | 1341 (828-2115) | 70.44 (43.08-112.34) | 2999 (1880-4822) | 66.75 (41.23-108.67) | -0.21 (-0.27--0.14) |
| Estonia | 492 (370-644) | 25.87 (19.66-33.65) | 674 (531-851) | 33.72 (26.89-42.05) | -0.11 (-0.5-0.29) |
| Eswatini | 187 (128-270) | 45.2 (29.72-66.39) | 607 (322-939) | 73.29 (38.23-113.73) | 1.81 (0.96-2.67) |
| Ethiopia | 65386 (45759-83242) | 215.92 (150.86-278.32) | 70548 (53139-92414) | 100.39 (74.08-131.72) | -3.01 (-3.19--2.83) |
| Fiji | 1178 (804-1663) | 195.86 (131.48-281.18) | 2820 (1963-3946) | 298.45 (208.27-416.32) | 1.05 (0.8-1.3) |
| Finland | 442 (311-610) | 6.94 (4.9-9.67) | 802 (558-1119) | 9.58 (6.68-13.35) | 1.44 (0.86-2.03) |
| France | 3750 (2764-4906) | 5.42 (4.04-7.11) | 5874 (4240-7884) | 6.2 (4.58-8.36) | 0.59 (0.46-0.72) |
| Gabon | 445 (301-640) | 68.14 (45.4-97.62) | 1296 (634-1918) | 90.48 (43.42-133.74) | 0.84 (0.62-1.06) |
| Gambia | 187 (123-267) | 34.89 (22.53-50.39) | 644 (431-949) | 43.77 (28.12-63.72) | 0.45 (0.24-0.67) |
| Georgia | 468 (343-627) | 7.99 (5.9-10.72) | 621 (425-877) | 14.18 (9.77-20.1) | 2.02 (1.59-2.44) |
| Germany | 8678 (6341-11593) | 8.08 (5.96-10.76) | 9962 (6906-13724) | 7.5 (5.25-10.24) | 0.15 (-0.21-0.51) |
| Ghana | 3433 (2321-5108) | 36.89 (24.19-55.79) | 14564 (9944-21182) | 58.18 (38.59-85.35) | 2.02 (1.78-2.26) |
| Greece | 1330 (1002-1760) | 9.72 (7.41-12.63) | 1634 (1258-2044) | 10.29 (8.01-12.89) | -0.75 (-1.6-0.1) |
| Greenland | 6 (4-8) | 11.6 (8.58-16.16) | 8 (6-11) | 10.92 (7.79-15.02) | 0.13 (0.05-0.22) |
| Grenada | 95 (74-116) | 152.04 (117.91-188.9) | 243 (182-318) | 201.26 (154.49-262.04) | 1.67 (1.39-1.94) |
| Guam | 96 (75-128) | 82.03 (63.23-111.15) | 326 (255-407) | 179.41 (142.19-222.64) | 3.36 (3.09-3.63) |
| Guatemala | 2183 (1688-2894) | 45.13 (34.25-60.27) | 10893 (7747-14677) | 85.77 (59.75-117.91) | 2.93 (2.46-3.4) |
| Guinea | 933 (619-1416) | 23.34 (14.92-36.31) | 1959 (1299-2958) | 24.39 (15.7-38.13) | 0.2 (0.1-0.29) |
| Guinea-Bissau | 354 (242-504) | 61.95 (41.76-90.48) | 681 (457-1019) | 54.23 (35.39-80.55) | -0.5 (-0.56--0.45) |
| Guyana | 631 (495-800) | 117.39 (91-151.38) | 1756 (1204-2445) | 231.14 (158.32-321.38) | 3.25 (2.88-3.62) |
| Haiti | 5176 (3530-8226) | 113.15 (75.08-185.96) | 12049 (6537-25021) | 113.43 (61.24-235.82) | 0.3 (0.2-0.41) |
| Honduras | 592 (418-796) | 22.08 (15.4-30.62) | 2651 (1691-4032) | 34.89 (21.88-53.59) | 1.6 (1.41-1.8) |
| Hungary | 1176 (876-1568) | 9.03 (6.85-11.8) | 1176 (863-1619) | 7.94 (5.77-10.84) | -0.41 (-0.99-0.17) |
| Iceland | 7 (5-11) | 2.9 (2.07-4.14) | 20 (15-28) | 4.3 (3.12-6.02) | 1.73 (1.56-1.9) |
| India | 229917 (170593-298188) | 36.88 (26.78-48.11) | 595003 (428352-823441) | 43.29 (31.02-59.74) | 0.47 (0.37-0.57) |
| Indonesia | 153478 (116283-192100) | 96.95 (74.21-122.27) | 337733 (251527-450065) | 107.89 (80.31-143.87) | 0.49 (0.41-0.57) |
| Iran (Islamic Republic of) | 5085 (3564-7191) | 13.67 (9.43-19.65) | 12764 (9413-17042) | 12.95 (9.61-17.3) | 0.08 (-0.03-0.18) |
| Iraq | 3635 (2308-5480) | 33.52 (21.02-51) | 9764 (5202-15505) | 28 (14.75-44.47) | -0.73 (-0.79--0.67) |
| Ireland | 281 (199-383) | 7.56 (5.36-10.43) | 762 (500-1130) | 11.41 (7.4-17.08) | 1.73 (1.62-1.85) |
| Israel | 831 (634-1066) | 18.15 (13.81-23.46) | 1889 (1441-2425) | 17.94 (13.85-22.95) | 1.42 (0.62-2.23) |
| Italy | 6458 (4879-8337) | 8.54 (6.54-10.86) | 8909 (6448-11793) | 9.74 (6.96-12.86) | -0.03 (-0.25-0.18) |
| Jamaica | 1136 (910-1403) | 65.33 (51.07-81.58) | 3299 (2296-4713) | 107.23 (74.96-153.04) | 1.19 (0.56-1.83) |
| Japan | 32657 (24972-40784) | 19.89 (15.37-24.72) | 29501 (21783-38428) | 15.35 (11.49-19.63) | -0.72 (-0.87--0.57) |
| Jordan | 654 (457-914) | 30.15 (20.9-42.66) | 3021 (2088-4308) | 26.57 (18.2-37.57) | -0.7 (-1.04--0.36) |
| Kazakhstan | 1543 (1147-2065) | 10.2 (7.53-13.84) | 2308 (1649-3153) | 11.26 (8.04-15.28) | -0.51 (-1.04-0.03) |
| Kenya | 4573 (3254-6815) | 39.07 (26.75-60.05) | 20329 (14074-28827) | 60.23 (41.16-86.82) | 1.66 (1.46-1.86) |
| Kiribati | 137 (107-173) | 240.48 (186.26-307.5) | 358 (215-568) | 330.91 (197.78-519.98) | 0.81 (0.51-1.11) |
| Kuwait | 278 (208-379) | 20.86 (14.88-28.75) | 528 (357-731) | 8.89 (5.89-12.26) | -2.55 (-2.76--2.35) |
| Kyrgyzstan | 382 (285-511) | 10.48 (7.84-13.95) | 864 (633-1191) | 13.34 (9.73-18.15) | -0.52 (-1.24-0.21) |
| Lao People's Democratic Republic | 6362 (4463-8978) | 214.24 (149.32-305.22) | 11398 (7584-17051) | 167.92 (110.93-250.34) | -0.96 (-1.03--0.88) |
| Latvia | 372 (283-488) | 11.27 (8.7-14.73) | 588 (420-820) | 20.17 (14.4-27.91) | 1.34 (1.07-1.61) |
| Lebanon | 509 (318-775) | 19.88 (12.44-30.24) | 836 (587-1205) | 13.99 (9.78-20.37) | -1.02 (-1.19--0.85) |
| Lesotho | 162 (106-241) | 16.24 (10.6-24.2) | 559 (325-840) | 40.32 (23.21-62.07) | 3.72 (3.13-4.31) |
| Liberia | 687 (462-1048) | 45.6 (30.34-70.34) | 1808 (1171-2629) | 48.96 (30.77-71.18) | 0.48 (0.15-0.8) |
| Libya | 446 (291-660) | 16.91 (10.86-25.29) | 2196 (1152-3402) | 27.63 (14.6-42.08) | 2.12 (1.95-2.29) |
| Lithuania | 408 (315-525) | 9.58 (7.36-12.38) | 746 (537-1026) | 17.76 (12.8-24.34) | 1.15 (0.79-1.51) |
| Luxembourg | 41 (31-55) | 8.4 (6.32-10.96) | 85 (62-118) | 9.06 (6.62-12.66) | 0.52 (0.4-0.65) |
| Madagascar | 3302 (2271-4680) | 47.93 (31.94-70.26) | 8492 (5577-12822) | 46.45 (30.9-70.4) | -0.06 (-0.14-0.02) |
| Malawi | 3445 (2431-4809) | 63.42 (44.09-91.17) | 8452 (5961-11614) | 75.38 (51.26-104.65) | 0.34 (0.07-0.61) |
| Malaysia | 12523 (9968-15412) | 92.21 (72.99-115.63) | 34278 (26568-43642) | 103.06 (78.22-131.48) | 0.31 (0.12-0.49) |
| Maldives | 275 (203-354) | 193.38 (143.24-251.82) | 470 (345-631) | 78.71 (58.94-106.25) | -3.09 (-3.3--2.87) |
| Mali | 2175 (1526-3209) | 40.02 (27.08-60.02) | 4747 (3179-7122) | 35.88 (23.36-55.33) | -0.3 (-0.44--0.17) |
| Malta | 43 (31-57) | 10.08 (7.23-13.53) | 79 (58-105) | 11.65 (8.6-15.35) | 0.69 (0.53-0.85) |
| Marshall Islands | 65 (40-110) | 243.35 (146.7-399.83) | 234 (77-625) | 432.28 (140.9-1145.68) | 1.82 (1.53-2.1) |
| Mauritania | 538 (351-776) | 43.2 (27.34-64) | 1013 (639-1591) | 35.85 (21.92-56.55) | -0.94 (-1.08--0.8) |
| Mauritius | 1799 (1478-2169) | 186.26 (150.4-228.02) | 5955 (4741-7336) | 361.58 (295.85-438.99) | 2.34 (1.96-2.72) |
| Mexico | 28216 (21997-36640) | 50.55 (37.96-66.49) | 148046 (105794-208940) | 106.06 (75.93-149.08) | 2.88 (2.18-3.58) |
| Micronesia (Federated States of) | 170 (108-256) | 250.35 (158.19-373.27) | 423 (284-618) | 428.33 (289.44-622.31) | 1.73 (1.32-2.14) |
| Monaco | 3 (2-4) | 6.47 (4.65-8.82) | 6 (4-8) | 9.08 (6.52-12.53) | 1.25 (1.06-1.45) |
| Mongolia | 223 (160-319) | 15.86 (11.25-22.8) | 470 (328-683) | 13.73 (9.67-19.76) | -0.73 (-0.98--0.49) |
| Montenegro | 83 (57-114) | 12.5 (8.77-17.11) | 121 (83-172) | 14.1 (9.8-19.7) | 1.07 (0.52-1.62) |
| Morocco | 2105 (1331-3303) | 11.59 (7.12-18.38) | 6560 (4205-10123) | 16.61 (10.68-25.49) | 1.58 (1.41-1.75) |
| Mozambique | 3529 (2458-5268) | 41.87 (28.71-63.54) | 10989 (7264-16707) | 61.82 (40.33-91.9) | 1.83 (1.63-2.04) |
| Myanmar | 55413 (37793-74872) | 160.75 (111.22-217.81) | 68616 (49476-93316) | 118.28 (85.79-160.64) | -1.29 (-1.43--1.16) |
| Namibia | 180 (118-296) | 21.73 (14.16-35.87) | 487 (308-771) | 25.98 (16.47-39.8) | 0.29 (-0.12-0.7) |
| Nauru | 23 (14-33) | 303.69 (182.08-433.79) | 43 (29-58) | 474.04 (318.63-640.09) | 1.28 (1.1-1.45) |
| Nepal | 4642 (3052-6632) | 36.9 (24.08-53.37) | 12723 (8054-18890) | 47.31 (29.74-70.26) | 0.99 (0.62-1.37) |
| Netherlands | 1028 (748-1391) | 5.8 (4.22-7.89) | 1793 (1272-2566) | 7.1 (4.96-10.06) | -0.01 (-0.38-0.36) |
| New Zealand | 184 (136-247) | 5.14 (3.75-6.91) | 428 (313-570) | 6.72 (5.03-8.99) | 1.2 (0.96-1.44) |
| Nicaragua | 1055 (770-1515) | 50.82 (35.91-74.28) | 6190 (4175-8680) | 105.2 (70.89-147.25) | 2.92 (2.47-3.38) |
| Niger | 983 (673-1461) | 22.42 (14.68-33.89) | 2223 (1415-3928) | 17.9 (11.23-31.5) | -0.84 (-0.99--0.68) |
| Nigeria | 15336 (10630-22055) | 26.25 (17.64-38.66) | 34079 (21258-51403) | 23.72 (14.69-34.98) | -0.79 (-1.03--0.56) |
| Niue | 4 (3-6) | 195.87 (136.57-276.69) | 7 (4-11) | 374.71 (210.84-599.52) | 1.68 (1.49-1.87) |
| North Macedonia | 175 (129-234) | 8.4 (6.2-11.2) | 279 (187-409) | 9.05 (6.09-13.12) | -0.29 (-0.64-0.06) |
| Northern Mariana Islands | 100 (68-139) | 239.74 (160.65-331.59) | 215 (160-275) | 348.26 (263.75-435.63) | 1.35 (1.14-1.55) |
| Norway | 250 (185-322) | 4.79 (3.58-6.2) | 523 (378-692) | 6.84 (4.93-9.14) | 0.59 (0.29-0.89) |
| Oman | 195 (127-307) | 15.8 (9.84-25.44) | 893 (605-1267) | 20.87 (13.77-31.95) | 1.42 (1.2-1.64) |
| Pakistan | 33067 (22983-47069) | 47.9 (32.58-68.18) | 119530 (79663-165541) | 68.2 (44.61-94.2) | 0.92 (0.67-1.17) |
| Palau | 27 (18-37) | 199.3 (130.76-277.13) | 81 (53-116) | 346.33 (222.02-491.36) | 1.94 (1.73-2.15) |
| Palestine | 247 (164-361) | 22.17 (14.31-33) | 760 (537-1099) | 19.9 (13.76-29.36) | -0.41 (-0.51--0.31) |
| Panama | 365 (275-481) | 20.76 (15.39-27.92) | 1952 (1337-2796) | 44.04 (30.34-62.92) | 2.64 (2.2-3.09) |
| Papua New Guinea | 3167 (1796-4531) | 103.07 (59.71-150.34) | 10458 (7826-13861) | 116.7 (87.2-154.45) | 0.33 (0.26-0.4) |
| Paraguay | 948 (703-1260) | 35.3 (25.36-47.86) | 3444 (2326-4908) | 52.4 (35.1-75.35) | 1.27 (1.1-1.45) |
| Peru | 8739 (6507-11315) | 58.06 (42.75-76.68) | 21593 (13828-31354) | 59.95 (38.19-87.13) | -0.08 (-0.29-0.13) |
| Philippines | 63326 (52275-77010) | 128.41 (105.12-156.72) | 202862 (155140-260531) | 186.78 (142.41-241.14) | 1.72 (1.53-1.92) |
| Poland | 6256 (4650-8226) | 14.84 (11.08-19.71) | 3782 (2802-5322) | 6.86 (5.11-9.67) | -2.54 (-2.91--2.17) |
| Portugal | 1514 (1137-1986) | 12.11 (9.24-15.6) | 1786 (1308-2374) | 10.38 (7.83-13.67) | -0.27 (-0.63-0.09) |
| Puerto Rico | 3349 (2647-4162) | 94.22 (74.17-118.16) | 4910 (3689-6358) | 108.26 (83.92-137.1) | 1.06 (0.7-1.43) |
| Qatar | 52 (30-83) | 15.59 (8.99-24.63) | 398 (272-582) | 12.94 (8.58-19.25) | -0.92 (-1.22--0.62) |
| Republic of Korea | 12380 (9650-15362) | 30.56 (23.8-38.13) | 12875 (9390-16687) | 15.54 (11.68-19.84) | -1.54 (-1.81--1.27) |
| Republic of Moldova | 339 (265-424) | 7.28 (5.68-9.17) | 730 (541-973) | 13.43 (10.05-17.95) | 1.25 (0.86-1.64) |
| Romania | 1083 (795-1485) | 4.02 (2.94-5.53) | 1742 (1300-2318) | 6.22 (4.64-8.47) | 2.45 (1.99-2.92) |
| Russian Federation | 46504 (37315-59002) | 26.34 (21.17-33.39) | 46661 (34761-62628) | 21.3 (16.37-28.32) | -1.63 (-1.97--1.3) |
| Rwanda | 3059 (2186-4150) | 77.76 (54.51-106.81) | 4261 (2781-6178) | 47.07 (30.27-69.09) | -2.73 (-3.17--2.3) |
| Saint Kitts and Nevis | 49 (40-61) | 158.98 (126.93-199.7) | 129 (93-178) | 161.26 (118.28-219.85) | 0.59 (0.18-0.99) |
| Saint Lucia | 117 (95-144) | 123.86 (97.73-154.09) | 339 (254-452) | 146.7 (111.84-193.05) | 1.16 (0.92-1.41) |
| Saint Vincent and the Grenadines | 73 (59-90) | 95.96 (76.56-117.84) | 207 (157-268) | 152.77 (117.46-196.06) | 2.17 (1.88-2.47) |
| Samoa | 215 (144-313) | 192.61 (127.75-279.23) | 517 (359-709) | 292.56 (203.64-401.96) | 1.34 (1.25-1.42) |
| San Marino | 2 (1-2) | 5.1 (3.67-6.8) | 3 (2-4) | 5.79 (3.74-8.44) | 0.98 (0.8-1.16) |
| Sao Tome and Principe | 41 (28-56) | 55.81 (37.81-76.69) | 119 (74-170) | 72.69 (44.63-102.94) | 0.62 (0.36-0.89) |
| Saudi Arabia | 3558 (2267-5341) | 35.61 (22.53-54.19) | 28758 (17375-44548) | 65.31 (38.87-98.45) | 1.91 (1.77-2.05) |
| Senegal | 2134 (1476-3077) | 48.45 (32.64-72.25) | 5031 (3302-7988) | 47.11 (30.8-75.35) | -0.1 (-0.22-0.03) |
| Serbia | 1871 (1337-2638) | 15.95 (11.47-22.11) | 1929 (1389-2570) | 14.89 (11.12-19.46) | -0.27 (-0.43--0.1) |
| Seychelles | 82 (65-104) | 137.27 (105.67-174.93) | 229 (172-295) | 178.75 (136.02-227.62) | 1.09 (0.92-1.25) |
| Sierra Leone | 758 (482-1092) | 28.98 (18.08-42.02) | 1683 (1076-2558) | 29.45 (18.26-44.61) | 0.16 (0.1-0.22) |
| Singapore | 905 (727-1121) | 31.36 (24.4-39.14) | 1514 (1131-1952) | 17.52 (13.13-22.39) | 0.22 (-0.4-0.85) |
| Slovakia | 811 (579-1110) | 14.27 (10.16-19.74) | 794 (571-1132) | 10.08 (7.27-14.05) | -0.96 (-1.1--0.83) |
| Slovenia | 114 (89-143) | 4.89 (3.83-6.12) | 120 (82-167) | 3.99 (2.74-5.7) | -0.72 (-0.94--0.5) |
| Solomon Islands | 467 (145-808) | 212.36 (70.21-362.35) | 1445 (967-1934) | 251.63 (168.91-337.97) | 0.51 (0.29-0.73) |
| Somalia | 3592 (2340-5431) | 87.32 (55.52-134.18) | 9534 (5914-15854) | 90.9 (55.97-152.78) | 0.15 (0.05-0.25) |
| South Africa | 7653 (5720-10502) | 27.81 (20.52-38.36) | 18798 (13672-25662) | 32.82 (23.55-45.13) | 1.13 (0.73-1.52) |
| South Sudan | 2165 (1436-3192) | 67.67 (44.06-100.15) | 5619 (3605-8152) | 94.65 (59.36-137.07) | 0.99 (0.7-1.28) |
| Spain | 4488 (3387-5949) | 9.42 (7.21-12.24) | 4971 (3658-6663) | 6.88 (5.09-9.24) | -0.31 (-0.6--0.01) |
| Sri Lanka | 13246 (10124-17162) | 87.77 (66.3-114.28) | 19494 (12452-27986) | 76.56 (49.07-109.3) | -0.65 (-0.88--0.42) |
| Sudan | 1382 (855-2405) | 10.82 (6.64-19.27) | 4212 (2536-6786) | 13.59 (8.24-21.68) | 0.7 (0.53-0.87) |
| Suriname | 373 (281-474) | 118.04 (88.63-150.48) | 1165 (815-1598) | 177.6 (126.18-240.81) | 1.5 (1.3-1.7) |
| Sweden | 647 (483-854) | 5.62 (4.16-7.4) | 1150 (857-1537) | 7.62 (5.64-10.16) | 1.53 (1.18-1.88) |
| Switzerland | 388 (286-521) | 4.47 (3.3-6.02) | 608 (417-887) | 4.61 (3.14-6.86) | -0.32 (-0.61--0.03) |
| Syrian Arab Republic | 2174 (1487-3130) | 28.5 (19.24-42.36) | 4006 (2580-6040) | 26.11 (17.23-38.89) | -0.67 (-1.05--0.3) |
| Taiwan (Province of China) | 16710 (14268-19680) | 83.68 (70.49-99.31) | 22606 (17764-27616) | 65.02 (52.83-77.23) | -0.16 (-0.4-0.09) |
| Tajikistan | 157 (115-215) | 4.37 (3.12-5.93) | 458 (307-649) | 5.13 (3.46-7.34) | 0.05 (-0.16-0.26) |
| Thailand | 50061 (37875-65883) | 95.73 (72.37-129.77) | 100321 (71264-137923) | 111.42 (82.6-151.3) | -0.18 (-0.56-0.21) |
| Timor-Leste | 561 (388-790) | 100.42 (68.96-144.47) | 1062 (713-1550) | 101.08 (67.44-149.04) | 0.12 (-0.24-0.48) |
| Togo | 681 (466-995) | 34.74 (23.24-51.82) | 2308 (1552-3497) | 38.52 (25.22-58.96) | 0.27 (0.17-0.36) |
| Tokelau | 2 (1-4) | 167.89 (107.47-290.75) | 4 (3-5) | 272.77 (204.05-376.19) | 1.23 (1.07-1.38) |
| Tonga | 68 (46-100) | 99.97 (67.58-149.63) | 118 (76-164) | 132.25 (86.3-184.52) | 0.54 (0.32-0.76) |
| Trinidad and Tobago | 867 (707-1065) | 88.09 (70.38-109.84) | 2816 (1900-4049) | 158.53 (108.81-224.53) | 2.28 (1.97-2.6) |
| Tunisia | 737 (495-1101) | 11.9 (7.8-18.11) | 2225 (1379-3495) | 15.8 (9.9-24.56) | 0.89 (0.67-1.11) |
| Türkiye | 410 (301-543) | 15.52 (11.53-20.29) | 1304 (884-1876) | 25.04 (17-36.06) | 1.51 (1.19-1.84) |
| Turkmenistan | 18 (12-26) | 212.83 (150.06-321.78) | 35 (24-49) | 295.04 (205.58-416.22) | 1.12 (1.04-1.2) |
| Tuvalu | 8710 (6028-12118) | 19.16 (13.1-26.87) | 14319 (9724-20791) | 14.61 (9.96-21.13) | -0.69 (-0.87--0.51) |
| Uganda | 4460 (2988-6638) | 51.96 (33.91-77.56) | 14555 (9883-20769) | 63.82 (41.59-91.96) | 0.12 (-0.14-0.39) |
| Ukraine | 1232 (835-1815) | 1.87 (1.28-2.76) | 7723 (5334-10497) | 12.37 (8.54-17.01) | 8.45 (7.21-9.7) |
| United Arab Emirates | 204 (136-306) | 15.51 (9.46-23.89) | 1794 (1047-2621) | 14.67 (8.85-21.48) | 0.45 (0.09-0.82) |
| United Kingdom | 4917 (3793-6205) | 6.8 (5.31-8.48) | 7150 (5643-9017) | 7.41 (5.85-9.23) | 0.79 (0.52-1.06) |
| United Republic of Tanzania | 3803 (2629-5370) | 26.68 (17.84-39.23) | 9842 (6498-14607) | 27.17 (17.64-40.75) | -0.15 (-0.22--0.08) |
| United States of America | 36267 (28595-45040) | 12.85 (10.13-16.04) | 116845 (90220-147674) | 26.06 (20.55-32.36) | 2.22 (1.9-2.55) |
| United States Virgin Islands | 85 (65-109) | 79.76 (59.78-100.69) | 106 (72-149) | 107.19 (75.97-147.68) | 1.66 (1.48-1.85) |
| Uruguay | 447 (350-552) | 12.94 (10.3-15.82) | 648 (478-853) | 15.25 (11.35-19.95) | 1.12 (0.77-1.48) |
| Uzbekistan | 1082 (778-1580) | 7.26 (5.15-10.14) | 5960 (4418-8239) | 16.81 (12.47-23.1) | 1.95 (1.36-2.54) |
| Vanuatu | 163 (92-277) | 160.65 (89.9-273.91) | 685 (450-1034) | 264.08 (173.55-400.46) | 1.54 (1.47-1.62) |
| Venezuela (Bolivarian Republic of) | 3329 (2524-4421) | 26.31 (19.27-35.19) | 18943 (11945-27767) | 59.71 (37.92-86.83) | 2.08 (1.59-2.57) |
| Viet Nam | 44781 (28276-61029) | 88.89 (57.58-121.51) | 96786 (64872-133791) | 84.56 (56.61-116.31) | 0.09 (-0.12-0.29) |
| Yemen | 1026 (596-1902) | 14.36 (8.35-26.95) | 2742 (1668-4659) | 12.39 (7.36-21.63) | -0.6 (-0.68--0.51) |
| Zambia | 3407 (2351-4621) | 81.59 (56.21-112.86) | 11077 (6782-18478) | 96.5 (59.16-162.57) | 0.2 (0.04-0.36) |
| Zimbabwe | 1153 (771-1789) | 21.48 (14.33-33.38) | 4322 (2790-6355) | 41.6 (27.2-61.51) | 2.53 (1.85-3.22) |

| **TableS14: Trends in the burden of chronic kidney disease due to diabetes mellitus type 1 by SDI: incidence, prevalence, deaths, and disability-adjusted life years (1990–2021).** | | | | | |
| --- | --- | --- | --- | --- | --- |
| SDI | 1990 | | 2021 | | **EAPC_95%CI** |
|  | Number_95%UI | ASR | Number_95%UI | ASR |  |
| **Incidence** |  |  |  |  |  |
| High-middle SDI | 10075 (7028-14791) | 1.06 (0.73-1.58) | 13073 (10659-16532) | 1.26 (0.95-1.73) | 0.69 (0.63-0.76) |
| High SDI | 9585 (7663-12567) | 1.25 (0.97-1.71) | 14678 (12302-17480) | 1.46 (1.15-1.91) | 0.55 (0.49-0.61) |
| Low-middle SDI | 15572 (10034-22823) | 1.03 (0.7-1.46) | 23675 (16376-34384) | 1.24 (0.86-1.78) | 0.65 (0.57-0.73) |
| Low SDI | 7356 (4677-11522) | 0.95 (0.65-1.42) | 15682 (10518-23236) | 1.1 (0.78-1.56) | 0.53 (0.45-0.61) |
| Middle SDI | 20958 (15491-28413) | 1.1 (0.83-1.47) | 27946 (22863-34711) | 1.35 (1.06-1.72) | 0.82 (0.77-0.87) |
| **Prevalence** |  |  |  |  |  |
| High-middle SDI | 567123 (495242-651656) | 51.55 (44.84-58.94) | 1145979 (996133-1307724) | 81.65 (69.82-94.12) | 1.89 (1.76-2.02) |
| High SDI | 745897 (633891-863641) | 78.69 (66.74-91.02) | 1177232 (1018653-1365833) | 98.23 (83.87-115.4) | 0.89 (0.84-0.95) |
| Low-middle SDI | 624409 (510724-751927) | 58.96 (48.6-69.74) | 1416617 (1150430-1728664) | 72.34 (58.95-87.45) | 1 (0.88-1.11) |
| Low SDI | 223627 (180323-270425) | 53.74 (44.6-64.35) | 660372 (529853-799807) | 66.26 (53.74-79.88) | 0.97 (0.88-1.05) |
| Middle SDI | 803841 (681184-951016) | 48.03 (41.25-56.34) | 1889960 (1613277-2238020) | 72.79 (61.69-86.38) | 1.98 (1.82-2.14) |
| **Deaths** |  |  |  |  |  |
| High-middle SDI | 9483 (7646-11752) | 0.89 (0.71-1.11) | 12785 (9874-16518) | 0.71 (0.56-0.92) | -1.01 (-1.19--0.83) |
| High SDI | 3419 (2666-4285) | 0.33 (0.26-0.42) | 6999 (5300-8951) | 0.42 (0.33-0.53) | 0.92 (0.75-1.09) |
| Low-middle SDI | 9211 (6858-11814) | 1.18 (0.86-1.52) | 22730 (16853-30310) | 1.35 (0.99-1.79) | 0.43 (0.39-0.47) |
| Low SDI | 4377 (3228-5718) | 1.51 (1.1-2.02) | 8707 (6391-11568) | 1.31 (0.95-1.79) | -0.67 (-0.78--0.57) |
| Middle SDI | 22764 (18253-28000) | 1.66 (1.31-2.08) | 42695 (32435-54427) | 1.49 (1.15-1.89) | -0.39 (-0.45--0.33) |
| **DALYs** |  |  |  |  |  |
| High-middle SDI | 430914 (355801-521099) | 39.82 (32.99-48.24) | 536774 (425929-686928) | 31.25 (24.96-39.27) | -1.05 (-1.22--0.87) |
| High SDI | 165889 (136965-201017) | 16.64 (13.78-20.17) | 297421 (236784-365573) | 19.82 (16.01-24.29) | 0.7 (0.59-0.82) |
| Low-middle SDI | 396626 (310559-498330) | 47.32 (35.97-59.9) | 918175 (705470-1183415) | 52.63 (40.03-68.72) | 0.33 (0.29-0.37) |
| Low SDI | 180555 (138988-228863) | 57.26 (42.78-73.83) | 362228 (274222-467222) | 49.48 (36.58-65.09) | -0.7 (-0.8--0.6) |
| Middle SDI | 1051467 (848724-1259151) | 71.88 (58.07-86.85) | 1756700 (1392097-2179224) | 61.89 (49.76-76.37) | -0.52 (-0.58--0.46) |

**TableS15: Trends in the burden of chronic kidney disease due to diabetes mellitus type 1 by sex: incidence, prevalence, deaths, and disability-adjusted life years (1990–2021).**

| Sex | 1990 | | 2021 | | **EAPC_95%CI** |
| --- | --- | --- | --- | --- | --- |
|  | Number_95%UI | ASR | Number_95%UI | ASR |  |
| **Incidence** |  |  |  |  |  |
| Global | 63601 (52476-76375) | 1.1 (0.92-1.31) | 95140 (82237-111471) | 1.31 (1.12-1.55) | 0.69 (0.64-0.74) |
| Female | 26773 (21783-33119) | 0.94 (0.77-1.15) | 38228 (32027-45888) | 1.08 (0.89-1.33) | 0.55 (0.51-0.58) |
| Male | 36829 (30520-43841) | 1.25 (1.06-1.48) | 56911 (49528-65500) | 1.53 (1.32-1.78) | 0.79 (0.73-0.86) |
| **Prevalence** |  |  |  |  |  |
| Global | 2967857 (2607069-3328285) | 57.54 (50.87-64.11) | 6295711 (5459693-7114345) | 77.31 (66.91-87.58) | 1.36 (1.26-1.47) |
| Female | 1728367 (1482021-1974137) | 66.85 (57.8-75.9) | 3668687 (3109394-4232810) | 90.71 (76.97-104.6) | 1.38 (1.28-1.48) |
| Male | 1239490 (1113261-1373486) | 48.37 (43.74-53.53) | 2627023 (2318287-2923705) | 64.19 (56.78-71.74) | 1.34 (1.23-1.46) |
| **Deaths** |  |  |  |  |  |
| Global | 49300 (39088-61208) | 1.08 (0.84-1.35) | 94020 (71457-119984) | 1.08 (0.83-1.38) | -0.07 (-0.13--0.01) |
| Female | 22377 (17833-28820) | 0.97 (0.77-1.26) | 39416 (30272-51111) | 0.89 (0.69-1.15) | -0.42 (-0.51--0.33) |
| Male | 26924 (20680-34089) | 1.19 (0.9-1.53) | 54604 (40656-69965) | 1.28 (0.96-1.63) | 0.2 (0.16-0.25) |
| **DALYs** |  |  |  |  |  |
| Global | 2227518 (1835373-2679208) | 47.05 (38.4-57.32) | 3875628 (3062396-4845503) | 45.2 (36.01-56.35) | -0.21 (-0.28--0.15) |
| Female | 1010830 (828455-1248632) | 42.69 (34.68-53.34) | 1609247 (1278069-2036183) | 37.27 (29.68-47.09) | -0.59 (-0.68--0.5) |
| Male | 1216689 (978326-1486172) | 51.44 (40.65-63.6) | 2266380 (1739578-2862625) | 53.21 (40.96-67.05) | 0.08 (0.03-0.12) |

| **TableS16: Trends in the burden of chronic kidney disease due to diabetes mellitus type 2 across different age groups: incidence, prevalence, deaths, and disability-adjusted life years (1990–2021).** | | | | | |
| --- | --- | --- | --- | --- | --- |
| Age | 1990 | | 2021 | | **EAPC_95%CI** |
|  | Number_95%UI | ASR | Number_95%UI | ASR |  |
| **Incidence** |  |  |  |  |  |
| <15 years | 0 (0-0) | 0 (0-0) | 0 (0-0) | 0 (0-0) | 0 (0-0) |
| 15–19 years | 813 (399-1302) | 0.16 (0.08-0.25) | 790 (477-1171) | 0.13 (0.08-0.19) | -1.07 (-1.23--0.9) |
| 20–24 years | 1608 (764-2613) | 0.33 (0.16-0.53) | 1454 (834-2161) | 0.24 (0.14-0.36) | -1.22 (-1.29--1.15) |
| 25–29 years | 2798 (1292-4851) | 0.63 (0.29-1.1) | 2861 (1467-4729) | 0.49 (0.25-0.8) | -0.94 (-1--0.87) |
| 30–34 years | 4952 (2734-7838) | 1.28 (0.71-2.03) | 7238 (4350-10488) | 1.2 (0.72-1.74) | -0.23 (-0.34--0.12) |
| 35–39 years | 10491 (6355-15081) | 2.98 (1.8-4.28) | 18095 (11764-24970) | 3.23 (2.1-4.45) | 0.21 (0.09-0.34) |
| 40–44 years | 18536 (12495-26398) | 6.47 (4.36-9.21) | 37542 (26903-49944) | 7.5 (5.38-9.98) | 0.4 (0.32-0.49) |
| 45–49 years | 29777 (19639-39904) | 12.82 (8.46-17.19) | 72165 (50332-92102) | 15.24 (10.63-19.45) | 0.53 (0.48-0.58) |
| 50–54 years | 51986 (34155-71714) | 24.46 (16.07-33.74) | 135800 (96388-180655) | 30.52 (21.66-40.6) | 0.76 (0.7-0.81) |
| 55–59 years | 80809 (54578-106712) | 43.63 (29.47-57.62) | 213195 (158254-265330) | 53.87 (39.99-67.05) | 0.76 (0.72-0.81) |
| 60–64 years | 115496 (80035-154171) | 71.91 (49.83-95.99) | 289436 (216095-370252) | 90.44 (67.52-115.69) | 0.7 (0.67-0.72) |
| 65–69 years | 135546 (94051-177872) | 109.66 (76.09-143.9) | 364163 (272571-475468) | 132.02 (98.81-172.37) | 0.65 (0.63-0.66) |
| 70–74 years | 122188 (90555-157155) | 144.33 (106.96-185.63) | 366045 (286728-459891) | 177.83 (139.3-223.42) | 0.68 (0.65-0.7) |
| 75–79 years | 99139 (70787-131032) | 161.06 (115-212.87) | 262636 (198412-330053) | 199.14 (150.44-250.26) | 0.63 (0.6-0.67) |
| 80–84 years | 56199 (39806-75730) | 158.86 (112.52-214.07) | 164993 (120205-213601) | 188.38 (137.25-243.88) | 0.51 (0.47-0.55) |
| 85–89 years | 18992 (13227-25918) | 125.68 (87.53-171.52) | 59338 (43133-77779) | 129.78 (94.34-170.11) | 0.08 (0.05-0.12) |
| 90–94 years | 3613 (2534-4925) | 84.31 (59.14-114.94) | 15315 (11417-19471) | 85.61 (63.82-108.84) | 0.08 (-0.02-0.19) |
| 95+ years | 164 (95-251) | 16.16 (9.33-24.64) | 959 (585-1386) | 17.59 (10.73-25.43) | 0.27 (0.11-0.44) |
| **Prevalence** |  |  |  |  |  |
| <15 years | 0 (0-0) | 0 (0-0) | 0 (0-0) | 0 (0-0) | 0 (0-0) |
| 15–19 years | 955952 (698908-1272239) | 184.04 (134.55-244.93) | 743681 (536946-1010427) | 119.18 (86.05-161.93) | -1.72 (-1.91--1.53) |
| 20–24 years | 2760306 (1970056-3681881) | 560.94 (400.35-748.22) | 2309501 (1681845-3138684) | 386.75 (281.64-525.6) | -1.48 (-1.58--1.37) |
| 25–29 years | 4181575 (3001551-5682710) | 944.73 (678.13-1283.88) | 4244851 (3117057-5579761) | 721.49 (529.8-948.39) | -1.01 (-1.06--0.97) |
| 30–34 years | 4631195 (3411194-6140863) | 1201.59 (885.05-1593.28) | 6262885 (4684973-8091950) | 1036.08 (775.04-1338.66) | -0.53 (-0.6--0.46) |
| 35–39 years | 4925670 (3777130-6348469) | 1398.37 (1072.3-1802.29) | 7338835 (5579241-9452895) | 1308.48 (994.75-1685.41) | -0.24 (-0.32--0.16) |
| 40–44 years | 4481657 (3409885-5631851) | 1564.38 (1190.26-1965.87) | 7737302 (5865062-9736446) | 1546.68 (1172.42-1946.31) | -0.07 (-0.13-0) |
| 45–49 years | 4267920 (3383829-5464690) | 1838.07 (1457.32-2353.49) | 8817241 (6980290-11362319) | 1862.12 (1474.18-2399.62) | 0.05 (0.01-0.1) |
| 50–54 years | 4621564 (3631504-5738209) | 2174.12 (1708.37-2699.43) | 9793156 (7606982-12252252) | 2201.09 (1709.73-2753.79) | 0.1 (0.05-0.15) |
| 55–59 years | 4949248 (4020579-6046430) | 2672.38 (2170.94-3264.81) | 10574813 (8500170-12983805) | 2672.24 (2147.98-3280.99) | 0.07 (0.02-0.12) |
| 60–64 years | 5368226 (4491585-6357470) | 3342.41 (2796.59-3958.35) | 10637096 (8889779-12663398) | 3323.6 (2777.64-3956.72) | 0 (-0.04-0.04) |
| 65–69 years | 5109357 (4240409-6012972) | 4133.47 (3430.49-4864.49) | 11157898 (9201803-13205782) | 4045.03 (3335.89-4787.44) | -0.06 (-0.07--0.04) |
| 70–74 years | 4423985 (3780232-5086458) | 5225.52 (4465.13-6008.02) | 10348990 (8802271-11958241) | 5027.71 (4276.28-5809.51) | -0.06 (-0.08--0.03) |
| 75–79 years | 3804127 (3266263-4301310) | 6179.99 (5306.21-6987.69) | 7945254 (6793043-9069419) | 6024.41 (5150.76-6876.79) | -0.05 (-0.08--0.03) |
| 80–84 years | 2360381 (2061586-2667656) | 6672.28 (5827.65-7540.88) | 5656107 (4923524-6382072) | 6457.99 (5621.55-7286.88) | -0.07 (-0.09--0.06) |
| 85–89 years | 988786 (869001-1104985) | 6543.45 (5750.76-7312.42) | 2868340 (2531428-3231741) | 6273.46 (5536.59-7068.27) | -0.11 (-0.13--0.09) |
| 90–94 years | 253654 (223549-286715) | 5919.32 (5216.78-6690.83) | 1010565 (892969-1146080) | 5648.97 (4991.62-6406.49) | -0.14 (-0.16--0.12) |
| 95+ years | 21665 (16875-27689) | 2128.02 (1657.51-2719.69) | 113439 (88087-145596) | 2081.34 (1616.18-2671.34) | -0.05 (-0.08--0.03) |
| **Deaths** |  |  |  |  |  |
| <15 years | 0 (0-0) | 0 (0-0) | 0 (0-0) | 0 (0-0) | 0 (0-0) |
| 15–19 years | 2 (0-4) | 0 (0-0) | 1 (0-3) | 0 (0-0) | -1.81 (-1.91--1.72) |
| 20–24 years | 19 (5-51) | 0 (0-0.01) | 16 (4-43) | 0 (0-0.01) | -1.44 (-1.52--1.36) |
| 25–29 years | 102 (34-232) | 0.02 (0.01-0.05) | 107 (35-247) | 0.02 (0.01-0.04) | -1.07 (-1.21--0.93) |
| 30–34 years | 395 (159-742) | 0.1 (0.04-0.19) | 523 (211-998) | 0.09 (0.03-0.17) | -0.82 (-1--0.63) |
| 35–39 years | 1174 (579-2036) | 0.33 (0.16-0.58) | 1580 (778-2815) | 0.28 (0.14-0.5) | -0.64 (-0.79--0.49) |
| 40–44 years | 2253 (1138-3857) | 0.79 (0.4-1.35) | 3833 (1904-6520) | 0.77 (0.38-1.3) | -0.18 (-0.26--0.11) |
| 45–49 years | 3634 (2023-5703) | 1.56 (0.87-2.46) | 7868 (4517-12411) | 1.66 (0.95-2.62) | 0.29 (0.23-0.36) |
| 50–54 years | 7045 (4221-10287) | 3.31 (1.99-4.84) | 16351 (9764-24203) | 3.67 (2.19-5.44) | 0.35 (0.3-0.39) |
| 55–59 years | 11855 (7875-16246) | 6.4 (4.25-8.77) | 29671 (20234-40687) | 7.5 (5.11-10.28) | 0.56 (0.47-0.64) |
| 60–64 years | 16951 (11799-22918) | 10.55 (7.35-14.27) | 42240 (30388-57038) | 13.2 (9.49-17.82) | 0.71 (0.64-0.77) |
| 65–69 years | 21358 (15063-27884) | 17.28 (12.19-22.56) | 60583 (43299-77859) | 21.96 (15.7-28.23) | 0.85 (0.75-0.96) |
| 70–74 years | 23891 (17110-31132) | 28.22 (20.21-36.77) | 72407 (52958-92625) | 35.18 (25.73-45) | 0.9 (0.82-0.99) |
| 75–79 years | 23780 (17431-31078) | 38.63 (28.32-50.49) | 70973 (54369-91240) | 53.81 (41.22-69.18) | 1.04 (0.99-1.08) |
| 80–84 years | 18038 (12595-23854) | 50.99 (35.6-67.43) | 66234 (48013-85415) | 75.62 (54.82-97.53) | 1.44 (1.33-1.56) |
| 85–89 years | 11707 (8009-15899) | 77.47 (53-105.21) | 56580 (39295-75294) | 123.75 (85.94-164.68) | 1.81 (1.66-1.96) |
| 90–94 years | 4482 (3018-6413) | 104.59 (70.44-149.66) | 33566 (23302-46179) | 187.63 (130.26-258.14) | 2.25 (2.11-2.39) |
| 95+ years | 1284 (694-2011) | 126.15 (68.13-197.5) | 14739 (8328-22354) | 270.43 (152.8-410.14) | 2.81 (2.68-2.93) |
| **DALYs** |  |  |  |  |  |
| <15 years | 0 (0-0) | 0 (0-0) | 0 (0-0) | 0 (0-0) | 0 (0-0) |
| 15–19 years | 791 (471-1203) | 0.15 (0.09-0.23) | 487 (298-740) | 0.08 (0.05-0.12) | -2.59 (-2.75--2.44) |
| 20–24 years | 3466 (2063-5940) | 0.7 (0.42-1.21) | 2481 (1414-4386) | 0.42 (0.24-0.73) | -2 (-2.08--1.93) |
| 25–29 years | 12106 (6937-20715) | 2.74 (1.57-4.68) | 11653 (6505-20359) | 1.98 (1.11-3.46) | -1.28 (-1.35--1.21) |
| 30–34 years | 34262 (19230-54161) | 8.89 (4.99-14.05) | 44497 (24425-71801) | 7.36 (4.04-11.88) | -0.79 (-0.9--0.69) |
| 35–39 years | 83820 (49582-135925) | 23.8 (14.08-38.59) | 115202 (68900-188341) | 20.54 (12.28-33.58) | -0.53 (-0.63--0.43) |
| 40–44 years | 141987 (82169-221528) | 49.56 (28.68-77.33) | 242718 (140296-379032) | 48.52 (28.05-75.77) | -0.13 (-0.18--0.07) |
| 45–49 years | 204766 (130658-303106) | 88.19 (56.27-130.54) | 442246 (285732-641535) | 93.4 (60.34-135.49) | 0.29 (0.24-0.35) |
| 50–54 years | 341709 (224878-471492) | 160.75 (105.79-221.8) | 792060 (532495-1106857) | 178.02 (119.68-248.78) | 0.37 (0.33-0.41) |
| 55–59 years | 498709 (358159-660028) | 269.28 (193.39-356.39) | 1230675 (896212-1650236) | 310.99 (226.47-417.01) | 0.55 (0.48-0.62) |
| 60–64 years | 612908 (452777-791975) | 381.61 (281.91-493.11) | 1482394 (1122053-1926614) | 463.18 (350.59-601.98) | 0.67 (0.6-0.73) |
| 65–69 years | 643437 (480160-809688) | 520.54 (388.45-655.04) | 1766685 (1312344-2183659) | 640.47 (475.76-791.63) | 0.78 (0.68-0.88) |
| 70–74 years | 584586 (439599-727174) | 690.5 (519.25-858.92) | 1724464 (1298834-2136450) | 837.77 (630.99-1037.92) | 0.82 (0.74-0.91) |
| 75–79 years | 472975 (369715-597334) | 768.37 (600.62-970.4) | 1350725 (1073379-1657141) | 1024.17 (813.88-1256.51) | 0.93 (0.88-0.98) |
| 80–84 years | 286220 (211048-362879) | 809.08 (596.59-1025.78) | 985577 (748307-1232719) | 1125.31 (854.4-1407.49) | 1.24 (1.13-1.35) |
| 85–89 years | 143789 (104973-187355) | 951.55 (694.68-1239.85) | 644461 (466911-831152) | 1409.53 (1021.2-1817.85) | 1.54 (1.41-1.67) |
| 90–94 years | 46612 (33397-63422) | 1087.75 (779.35-1480.03) | 322079 (230107-433415) | 1800.39 (1286.28-2422.75) | 1.95 (1.84-2.07) |
| 95+ years | 10776 (5968-16707) | 1058.44 (586.17-1641.03) | 120532 (68992-182007) | 2211.46 (1265.83-3339.39) | 2.71 (2.59-2.82) |

**TableS17: Trends in the burden of chronic kidney disease due to diabetes mellitus type 2 by sex: incidence, prevalence, deaths, and disability-adjusted life years (1990–2021).**

| Sex | 1990 | | 2021 | | **EAPC_95%CI** |
| --- | --- | --- | --- | --- | --- |
|  | Number_95%UI | ASR | Number_95%UI | ASR |  |
| **Incidence** |  |  |  |  |  |
| Global | 753106 (680930-826928) | 19.07 (17.28-20.83) | 2012025 (1857800-2154288) | 23.07 (21.4-24.72) | 0.61 (0.6-0.63) |
| Female | 376968 (341204-413084) | 17.62 (15.96-19.26) | 990074 (915628-1063729) | 21.42 (19.84-22.97) | 0.64 (0.62-0.65) |
| Male | 376138 (339359-412959) | 20.88 (18.91-22.8) | 1021951 (943498-1098453) | 25.01 (23.14-26.82) | 0.57 (0.56-0.59) |
| **Prevalence** |  |  |  |  |  |
| Global | 58105268 (53056992-63286818) | 1327.22 (1223.26-1439.42) | 107559955 (99170797-115994732) | 1259.63 (1161.99-1359.92) | -0.17 (-0.2--0.14) |
| Female | 28360560 (25996835-30841479) | 1250.08 (1152.38-1357.8) | 52837532 (48769234-56931920) | 1191.96 (1100.99-1286.63) | -0.14 (-0.18--0.1) |
| Male | 29744708 (27057208-32317657) | 1414.09 (1298.44-1528.78) | 54722423 (50395175-59068229) | 1333.1 (1229.35-1437.39) | -0.21 (-0.23--0.18) |
| **Deaths** |  |  |  |  |  |
| Global | 147970 (124179-176413) | 4.15 (3.5-4.94) | 477273 (401541-565951) | 5.72 (4.83-6.79) | 1.17 (1.1-1.24) |
| Female | 72522 (60489-86024) | 3.6 (3-4.29) | 230668 (193896-274409) | 4.93 (4.15-5.86) | 1.1 (1.02-1.18) |
| Male | 75448 (61702-93510) | 4.98 (4.09-6.13) | 246605 (204152-294508) | 6.77 (5.57-8.14) | 1.17 (1.1-1.24) |
| **DALYs** |  |  |  |  |  |
| Global | 4122919 (3498980-4818958) | 105.71 (90.68-122.67) | 11278935 (9682785-13103871) | 131.08 (112.75-152.49) | 0.81 (0.75-0.87) |
| Female | 1975646 (1674230-2307158) | 93.54 (79.27-108.89) | 5262931 (4502103-6118770) | 113.67 (97.44-132.05) | 0.7 (0.63-0.76) |
| Male | 2147273 (1750418-2594132) | 121.65 (100.4-145.91) | 6016004 (5067209-7070255) | 151.81 (129.54-177.25) | 0.86 (0.81-0.92) |

| **TableS18: Trends in the burden of chronic kidney disease due to diabetes mellitus type 2 by SDI: incidence, prevalence, deaths, and disability-adjusted life years (1990–2021).** | | | | | |
| --- | --- | --- | --- | --- | --- |
| SDI | 1990 | | 2021 | | **EAPC_95%CI** |
|  | Number_95%UI | ASR | Number_95%UI | ASR |  |
| **Incidence** |  |  |  |  |  |
| High-middle SDI | 156966 (140079-172677) | 15.72 (14.12-17.28) | 404154 (369300-435464) | 20.09 (18.43-21.59) | 0.91 (0.88-0.94) |
| High SDI | 289954 (263980-316807) | 25.59 (23.3-27.81) | 595271 (547943-636879) | 28.34 (26.19-30.3) | 0.28 (0.24-0.32) |
| Low-middle SDI | 96145 (85820-107293) | 15.13 (13.54-16.71) | 298099 (270254-326985) | 20.2 (18.31-22.05) | 0.77 (0.7-0.84) |
| Low SDI | 27500 (24486-30720) | 11.9 (10.68-13.16) | 77298 (69537-84889) | 15.09 (13.65-16.6) | 0.65 (0.56-0.74) |
| Middle SDI | 181844 (161584-203076) | 17.13 (15.38-18.97) | 635310 (582063-685433) | 22.95 (21.15-24.58) | 0.99 (0.97-1.02) |
| **Prevalence** |  |  |  |  |  |
| High-middle SDI | 13233847 (12089212-14479468) | 1271.32 (1167.77-1384.68) | 21060241 (19314069-22837225) | 1157 (1062.14-1253.04) | -0.25 (-0.31--0.19) |
| High SDI | 11068895 (10251403-11847690) | 1037.34 (959.27-1110.52) | 18097946 (16763917-19359376) | 997.07 (918.7-1066.28) | -0.09 (-0.14--0.05) |
| Low-middle SDI | 11948110 (10785488-13071618) | 1586.44 (1452.35-1722.23) | 23752428 (21773065-25995904) | 1474.59 (1357.94-1606.52) | -0.31 (-0.35--0.27) |
| Low SDI | 3827766 (3462237-4236705) | 1355.23 (1238.78-1484.31) | 8155174 (7427183-9026803) | 1269.37 (1167.02-1378.81) | -0.28 (-0.31--0.25) |
| Middle SDI | 17975998 (16224751-19689647) | 1431.89 (1315.46-1554.26) | 36412911 (33598848-39348243) | 1331.6 (1232.11-1433.53) | -0.2 (-0.25--0.16) |
| **Deaths** |  |  |  |  |  |
| High-middle SDI | 26410 (21805-32380) | 3.01 (2.48-3.66) | 71227 (57909-86609) | 3.65 (2.96-4.43) | 0.73 (0.58-0.89) |
| High SDI | 26431 (21843-31511) | 2.36 (1.95-2.84) | 111565 (93260-132404) | 4.62 (3.92-5.4) | 2.51 (2.38-2.65) |
| Low-middle SDI | 25469 (20399-32174) | 4.9 (3.97-6.04) | 82399 (66443-102844) | 6.43 (5.2-7.98) | 0.89 (0.85-0.93) |
| Low SDI | 13362 (10622-16840) | 7.16 (5.8-8.95) | 29491 (23284-37041) | 7.36 (5.88-9.3) | 0.02 (-0.1-0.15) |
| Middle SDI | 56152 (47412-66286) | 6.77 (5.73-7.95) | 182160 (151835-216847) | 7.51 (6.21-8.97) | 0.44 (0.37-0.51) |
| **DALYs** |  |  |  |  |  |
| High-middle SDI | 757547 (633490-918717) | 78.21 (65.53-93.87) | 1678679 (1404999-2018747) | 84.71 (71.1-102.2) | 0.37 (0.24-0.51) |
| High SDI | 692418 (588252-796294) | 62.47 (53.01-71.88) | 2202413 (1928998-2486233) | 102.65 (90.04-114.51) | 1.9 (1.78-2.02) |
| Low-middle SDI | 747142 (612242-907224) | 125.03 (102.51-150.97) | 2202184 (1821393-2694852) | 155.33 (128.61-189.41) | 0.71 (0.68-0.74) |
| Low SDI | 360510 (290076-441002) | 166.43 (136.8-206.65) | 761198 (622328-942049) | 160.88 (131.69-199.61) | -0.21 (-0.31--0.12) |
| Middle SDI | 1561397 (1328370-1842152) | 157.87 (136.06-183.14) | 4424359 (3759638-5160626) | 167.06 (141.25-193.92) | 0.29 (0.22-0.36) |

| **TableS19: National Burden of chronic kidney disease due to diabetes mellitus type 2: incidence cases, ASIR, and EAPC (1990–2021).** | | | | | |
| --- | --- | --- | --- | --- | --- |
| Country | **1990** | | **2021** | | **EAPC_95%CI** |
|  | Number_95%UI | ASR | Number_95%UI | ASR |  |
| Afghanistan | 1859 (1575-2205) | 26.07 (22.32-30.25) | 3501 (3005-4113) | 36.73 (31.96-42.4) | 1.01 (0.8-1.22) |
| Albania | 253 (213-296) | 11.96 (10.16-13.86) | 925 (806-1065) | 20.34 (17.79-23.27) | 2.04 (1.85-2.22) |
| Algeria | 3391 (2902-3947) | 26.43 (22.8-30.68) | 15600 (13633-17933) | 42.47 (37.4-48.3) | 1.45 (1.38-1.52) |
| American Samoa | 5 (4-6) | 21.68 (18.75-25) | 16 (14-18) | 30.72 (26.75-35.19) | 1.11 (1.03-1.19) |
| Andorra | 13 (11-15) | 22.87 (19.7-26.12) | 34 (30-39) | 22.25 (19.3-25.44) | 0.01 (-0.06-0.08) |
| Angola | 341 (293-399) | 8.82 (7.74-10.11) | 1474 (1251-1712) | 12.51 (10.92-14.33) | 1.1 (0.91-1.28) |
| Antigua and Barbuda | 11 (10-13) | 21.92 (18.81-25.16) | 39 (34-44) | 34.49 (30.09-38.89) | 1.52 (1.41-1.63) |
| Argentina | 7205 (6168-8365) | 22.05 (18.95-25.53) | 15868 (13881-17970) | 27.76 (24.32-31.5) | 0.87 (0.76-0.98) |
| Armenia | 234 (194-282) | 8.19 (6.96-9.81) | 767 (657-900) | 17.31 (15.08-20.05) | 2.79 (2.64-2.95) |
| Australia | 5451 (4883-6089) | 26.55 (23.74-29.61) | 14077 (12352-15833) | 30.14 (26.42-34.04) | 0.41 (0.36-0.46) |
| Austria | 2721 (2341-3153) | 21.78 (18.86-25.49) | 5071 (4453-5782) | 27.49 (24.18-31.36) | 0.89 (0.81-0.97) |
| Azerbaijan | 479 (396-573) | 9.03 (7.55-10.61) | 2054 (1705-2438) | 17.96 (15.23-20.86) | 2.59 (2.38-2.79) |
| Bahamas | 58 (50-68) | 31.85 (27.59-36.53) | 517 (438-612) | 50.04 (43.58-56.97) | 1.41 (1.35-1.47) |
| Bahrain | 4286 (3670-5006) | 9.02 (7.76-10.41) | 18391 (15656-21277) | 12.97 (11.11-14.99) | 1.05 (0.94-1.17) |
| Bangladesh | 53 (46-61) | 19.17 (16.57-22.19) | 157 (138-180) | 30.45 (26.59-34.91) | 1.53 (1.43-1.62) |
| Barbados | 973 (831-1124) | 7.5 (6.4-8.7) | 1941 (1671-2229) | 12.27 (10.64-14.03) | 1.63 (1.41-1.86) |
| Belarus | 3947 (3417-4547) | 24.46 (21.19-28.28) | 5952 (5216-6676) | 25.08 (22.11-28.12) | 0.19 (0.14-0.23) |
| Belgium | 18 (15-21) | 18.98 (16.09-22.13) | 96 (82-110) | 30.15 (26.05-34.49) | 1.46 (1.38-1.55) |
| Belize | 265 (227-306) | 13.14 (11.37-15.05) | 889 (759-1031) | 17.14 (14.76-19.71) | 0.81 (0.72-0.9) |
| Benin | 12 (11-14) | 19.54 (16.74-22.65) | 43 (37-49) | 32.98 (28.47-37.4) | 1.91 (1.7-2.11) |
| Bermuda | 31 (26-37) | 11.98 (10.15-13.97) | 111 (94-129) | 17.89 (15.23-20.75) | 1.44 (1.38-1.51) |
| Bhutan | 2594 (2230-3047) | 25.63 (22.04-29.82) | 11929 (10453-13491) | 38.12 (33.47-42.87) | 1.44 (1.33-1.55) |
| Bolivia (Plurinational State of) | 513 (430-605) | 11.99 (10.24-13.95) | 1483 (1297-1695) | 23.38 (20.45-26.46) | 2.42 (2.24-2.61) |
| Bosnia and Herzegovina | 86 (74-100) | 15.05 (13.13-17.21) | 323 (280-374) | 21.59 (18.95-24.79) | 1.07 (0.87-1.27) |
| Botswana | 17679 (15797-19736) | 19.22 (17.21-21.41) | 64619 (59022-70003) | 25.4 (23.21-27.46) | 0.85 (0.8-0.9) |
| Brazil | 31 (26-35) | 31.79 (27.67-36.11) | 117 (101-135) | 34.5 (30.14-39.12) | 0.31 (0.22-0.39) |
| Brunei Darussalam | 1639 (1402-1925) | 12.6 (10.85-14.77) | 3163 (2771-3638) | 22.34 (19.73-25.77) | 1.88 (1.82-1.94) |
| Bulgaria | 532 (463-610) | 12.06 (10.55-13.7) | 1481 (1286-1675) | 15.86 (13.94-17.95) | 0.95 (0.87-1.02) |
| Burkina Faso | 209 (180-239) | 9.01 (7.8-10.27) | 521 (445-596) | 10.9 (9.47-12.4) | 0.54 (0.44-0.65) |
| Burundi | 535 (449-624) | 11.46 (9.64-13.17) | 2230 (1884-2604) | 17.19 (14.67-19.98) | 1.3 (1.15-1.45) |
| C?te d'Ivoire | 771 (655-906) | 17.04 (14.9-19.43) | 2960 (2545-3407) | 22.84 (20.14-26.05) | 0.87 (0.75-0.98) |
| Cabo Verde | 8206 (7165-9256) | 24.46 (21.39-27.59) | 18560 (16177-21497) | 24.88 (21.69-28.69) | 0.35 (0.24-0.45) |
| Cambodia | 101 (86-120) | 8.78 (7.63-10.09) | 248 (211-288) | 11.1 (9.76-12.54) | 0.68 (0.54-0.82) |
| Cameroon | 357 (304-415) | 12.36 (10.58-14.32) | 881 (759-1029) | 15.18 (13.17-17.39) | 0.6 (0.43-0.77) |
| Canada | 2228 (1913-2576) | 22.5 (19.35-26) | 8699 (7609-9893) | 33.42 (29.2-37.97) | 1.54 (1.38-1.69) |
| Central African Republic | 127561 (112718-142654) | 15.11 (13.45-16.8) | 354157 (321265-382784) | 16.29 (14.92-17.53) | 0.42 (0.34-0.5) |
| Chad | 4334 (3680-5101) | 23.77 (20.4-27.68) | 17991 (15655-20822) | 32.5 (28.26-37.41) | 1.18 (1.14-1.22) |
| Chile | 31 (27-36) | 19.51 (17.05-22.44) | 125 (109-144) | 28.82 (25.27-32.66) | 1.41 (1.31-1.51) |
| China | 19 (16-22) | 9.87 (8.47-11.17) | 59 (52-68) | 12.15 (10.61-13.9) | 0.55 (0.47-0.63) |
| Colombia | 105 (89-123) | 9.68 (8.38-11.1) | 389 (330-453) | 14.21 (12.43-16.18) | 1.19 (1.07-1.32) |
| Comoros | 2 (2-3) | 17.87 (15.58-20.58) | 7 (6-9) | 28.46 (24.68-32.55) | 1.51 (1.41-1.61) |
| Congo | 837 (758-925) | 47.42 (43.17-52.04) | 2603 (2352-2862) | 46.56 (42.08-51.12) | -0.07 (-0.08--0.06) |
| Cook Islands | 993 (840-1154) | 15.64 (13.44-18.02) | 2405 (2082-2701) | 27.12 (23.51-30.55) | 2.04 (1.88-2.2) |
| Costa Rica | 1488 (1272-1735) | 14.48 (12.42-16.93) | 5240 (4467-6051) | 27.23 (23.19-31.36) | 2.23 (2.13-2.34) |
| Croatia | 261 (223-303) | 29.71 (26.14-34) | 688 (602-780) | 31.08 (27.15-35.14) | 0.18 (0.05-0.31) |
| Cuba | 1815 (1542-2105) | 13.06 (11.14-15.12) | 4867 (4226-5610) | 22.4 (19.45-25.85) | 1.81 (1.62-1.99) |
| Cyprus | 2158 (1817-2530) | 13.4 (11.43-15.4) | 5914 (5119-6786) | 17.84 (15.42-20.5) | 0.95 (0.88-1.03) |
| Czechia | 1448 (1214-1694) | 9.23 (7.96-10.61) | 4496 (3791-5196) | 12.35 (10.72-14.26) | 0.87 (0.7-1.04) |
| Democratic People's Republic of Korea | 1683 (1454-1953) | 19.9 (17.25-23.04) | 3015 (2640-3426) | 24.15 (21.23-27.3) | 0.77 (0.72-0.83) |
| Democratic Republic of the Congo | 12 (11-15) | 9.46 (8.24-10.85) | 80 (69-93) | 12.63 (11.02-14.49) | 0.81 (0.65-0.97) |
| Denmark | 13 (11-15) | 22.26 (19.31-25.62) | 27 (23-31) | 30.98 (26.82-35.42) | 1.06 (0.99-1.14) |
| Djibouti | 523 (448-616) | 13.57 (11.75-15.83) | 2377 (2029-2700) | 23.53 (20.17-26.8) | 1.9 (1.82-1.98) |
| Dominica | 976 (829-1123) | 18.38 (15.55-21.14) | 5930 (5193-6750) | 35.84 (31.39-40.66) | 2.49 (2.36-2.62) |
| Dominican Republic | 7386 (6285-8696) | 26.69 (22.94-30.81) | 30039 (25450-35153) | 45.02 (39.2-51.43) | 1.61 (1.54-1.69) |
| Ecuador | 708 (598-830) | 23.35 (19.78-27.21) | 2379 (2090-2714) | 39.51 (34.63-45.09) | 2.1 (1.95-2.25) |
| Egypt | 17 (15-20) | 9.07 (7.92-10.38) | 82 (71-95) | 16.28 (14.18-18.5) | 2.2 (2.04-2.35) |
| El Salvador | 93 (78-110) | 8.47 (7.35-9.82) | 303 (256-356) | 11.01 (9.48-12.84) | 0.71 (0.57-0.85) |
| Equatorial Guinea | 187 (157-224) | 9.12 (7.67-10.91) | 501 (429-585) | 20.42 (17.51-23.86) | 2.9 (2.8-3.01) |
| Eritrea | 1764 (1557-1996) | 8.97 (8-9.97) | 4768 (4275-5327) | 11.22 (10.03-12.53) | 0.56 (0.41-0.71) |
| Estonia | 9 (8-11) | 17.72 (15.23-20.82) | 22 (19-26) | 27.23 (23.36-31.2) | 1.36 (1.31-1.42) |
| Eswatini | 74 (62-87) | 19.42 (16.66-22.27) | 212 (179-240) | 25.73 (22.29-29.18) | 0.81 (0.76-0.86) |
| Ethiopia | 1162 (983-1360) | 15.53 (13.19-18.04) | 2407 (2097-2774) | 17.82 (15.63-20.39) | 0.47 (0.4-0.54) |
| Fiji | 18189 (15690-21061) | 20.76 (17.98-23.88) | 32306 (28365-36778) | 22.74 (19.99-25.75) | 0.33 (0.26-0.4) |
| Finland | 66 (57-77) | 11.38 (9.93-13.03) | 191 (166-219) | 17.97 (15.85-20.45) | 1.42 (1.29-1.56) |
| France | 573 (471-680) | 8.94 (7.46-10.58) | 911 (776-1074) | 15.83 (13.55-18.49) | 2.11 (1.96-2.26) |
| Gabon | 31809 (27863-35858) | 24.26 (21.27-27.63) | 54022 (48788-60284) | 27.67 (24.96-30.56) | 0.44 (0.42-0.46) |
| Gambia | 719 (613-845) | 11.39 (9.85-13.02) | 2728 (2369-3161) | 16.11 (14.06-18.35) | 1.12 (1.01-1.23) |
| Georgia | 4827 (4198-5529) | 30.36 (26.58-34.83) | 6696 (6009-7571) | 28.36 (25.26-32.23) | -0.13 (-0.22--0.03) |
| Germany | 5 (4-6) | 18.09 (15.66-21.03) | 16 (14-18) | 23.61 (20.64-26.62) | 1.04 (0.97-1.11) |
| Ghana | 14 (12-16) | 20.95 (18.07-24.12) | 43 (37-48) | 35.49 (31.09-40.2) | 1.65 (1.55-1.75) |
| Greece | 15 (13-18) | 18.2 (15.7-20.86) | 54 (46-62) | 25.36 (22.16-29.07) | 1.09 (1.01-1.16) |
| Greenland | 926 (776-1085) | 25.23 (21.6-29.15) | 4391 (3794-5066) | 38.86 (33.66-44.87) | 1.54 (1.46-1.63) |
| Grenada | 432 (369-501) | 12.78 (11.06-14.69) | 929 (810-1074) | 16.4 (14.41-18.85) | 0.75 (0.65-0.84) |
| Guam | 55 (48-64) | 13.61 (11.95-15.54) | 118 (101-137) | 15.92 (13.88-18.26) | 0.44 (0.32-0.57) |
| Guatemala | 70 (60-81) | 17.4 (15.01-19.99) | 197 (168-228) | 28.56 (24.6-32.63) | 1.57 (1.52-1.62) |
| Guinea | 566 (475-678) | 16.54 (14.03-19.42) | 1671 (1409-1939) | 21.78 (18.63-25.16) | 0.92 (0.87-0.98) |
| Guinea-Bissau | 521 (441-612) | 24.25 (20.6-28.13) | 2141 (1851-2494) | 32.01 (27.9-37.21) | 1.02 (0.98-1.06) |
| Guyana | 1665 (1407-1952) | 11.1 (9.37-12.94) | 4530 (3950-5207) | 23.08 (20.1-26.54) | 2.53 (2.37-2.68) |
| Haiti | 56 (49-66) | 19.28 (16.86-22.38) | 119 (104-136) | 20.29 (17.71-23.06) | 0.17 (0.05-0.28) |
| Honduras | 75874 (66994-84751) | 15.28 (13.67-16.92) | 222793 (199078-245039) | 18.16 (16.32-19.97) | 0.27 (0.16-0.37) |
| Hungary | 11995 (10583-13517) | 11.8 (10.54-13.17) | 43242 (38382-48503) | 17.04 (15.27-18.87) | 1 (0.83-1.17) |
| Iceland | 2398 (2048-2798) | 30.18 (26.01-34.98) | 11405 (9809-13191) | 45.78 (39.99-52.57) | 1.33 (1.19-1.47) |
| India | 1204 (1074-1325) | 28.16 (25.06-30.96) | 1919 (1763-2072) | 23.89 (21.89-25.79) | -0.33 (-0.39--0.27) |
| Indonesia | 8653 (7577-9925) | 31.19 (27.84-34.8) | 32939 (30164-35878) | 41.45 (37.83-44.91) | 0.86 (0.81-0.92) |
| Iran (Islamic Republic of) | 1479 (1281-1716) | 29.19 (25.39-33.76) | 4028 (3503-4629) | 32.25 (28.08-36.56) | 0.43 (0.3-0.55) |
| Iraq | 20289 (17952-22778) | 21.71 (19.14-24.37) | 31474 (28467-34606) | 20.64 (18.63-22.58) | 0.01 (-0.04-0.06) |
| Ireland | 352 (304-406) | 20.29 (17.54-23.48) | 889 (781-1018) | 28.97 (25.45-33.18) | 1.23 (1.11-1.35) |
| Israel | 52456 (47493-57437) | 30.2 (27.39-33.06) | 105932 (95554-115892) | 30.63 (27.88-33.16) | 0.07 (0.01-0.13) |
| Italy | 414 (356-477) | 30.21 (26.44-34.55) | 3765 (3316-4252) | 47.76 (42.64-53.43) | 1.54 (1.48-1.6) |
| Jamaica | 1095 (914-1290) | 8.21 (6.91-9.63) | 3222 (2684-3758) | 16.65 (14-19.32) | 2.48 (2.27-2.68) |
| Japan | 722 (647-804) | 8.93 (8.03-9.83) | 2579 (2296-2864) | 11.29 (10.14-12.48) | 0.49 (0.3-0.67) |
| Jordan | 53 (45-61) | 18.12 (15.71-20.79) | 131 (111-149) | 22.31 (19.27-25.33) | 0.48 (0.26-0.7) |
| Kazakhstan | 6 (5-7) | 13.94 (11.95-16.24) | 15 (13-18) | 19.16 (16.55-21.93) | 0.99 (0.93-1.06) |
| Kenya | 204 (174-239) | 31.56 (27.23-35.67) | 1286 (1107-1491) | 42.56 (37.03-48.62) | 1.1 (1-1.19) |
| Kiribati | 246 (205-296) | 7.75 (6.54-9.14) | 657 (545-784) | 12.24 (10.24-14.38) | 1.52 (1.36-1.68) |
| Kuwait | 347 (296-408) | 16.02 (13.84-18.63) | 1066 (905-1230) | 21.89 (18.82-25.17) | 1.02 (0.91-1.12) |
| Kyrgyzstan | 272 (233-322) | 7.63 (6.54-9.04) | 547 (472-626) | 15.11 (13.02-17.54) | 2.21 (2.06-2.35) |
| Lao People's Democratic Republic | 616 (530-717) | 27.68 (24.26-31.75) | 2642 (2329-2967) | 45.53 (39.95-51.6) | 1.62 (1.55-1.69) |
| Latvia | 122 (104-140) | 14.3 (12.23-16.26) | 213 (182-244) | 19.05 (16.48-21.54) | 0.74 (0.5-0.98) |
| Lebanon | 152 (129-177) | 12.74 (10.94-14.71) | 364 (309-422) | 17.31 (14.98-19.57) | 1.16 (1.07-1.25) |
| Lesotho | 520 (443-604) | 27.32 (23.44-31.69) | 2289 (1967-2656) | 43.22 (37.37-49.49) | 1.29 (1.15-1.43) |
| Liberia | 391 (333-457) | 8.66 (7.38-10.13) | 694 (604-792) | 13.18 (11.51-15.07) | 1.43 (1.26-1.6) |
| Libya | 141 (123-163) | 24.7 (21.39-28.28) | 284 (246-318) | 26.87 (23.19-30.25) | 0.4 (0.28-0.52) |
| Lithuania | 410 (353-478) | 8.16 (7.15-9.34) | 1141 (984-1337) | 10.26 (8.95-11.74) | 0.68 (0.53-0.82) |
| Luxembourg | 346 (297-400) | 9.18 (7.97-10.55) | 856 (738-979) | 11.81 (10.26-13.45) | 0.77 (0.63-0.9) |
| Madagascar | 1675 (1430-1942) | 17.76 (15.36-20.4) | 7811 (6815-8936) | 26.25 (23.01-29.97) | 1.29 (1.24-1.34) |
| Malawi | 18 (15-21) | 19.18 (16.55-22.29) | 104 (90-121) | 30.23 (26.24-34.91) | 1.58 (1.46-1.7) |
| Malaysia | 486 (413-567) | 12 (10.34-13.67) | 1380 (1178-1601) | 15.45 (13.34-17.74) | 0.8 (0.72-0.89) |
| Maldives | 113 (98-130) | 25.84 (22.5-29.69) | 279 (244-315) | 27.06 (23.72-30.27) | 0.18 (0.1-0.25) |
| Mali | 3 (2-3) | 16.07 (13.77-18.55) | 9 (8-10) | 23.36 (20.26-26.43) | 1.12 (1.06-1.19) |
| Malta | 144 (123-168) | 13.93 (12.01-16.16) | 423 (362-490) | 19.23 (16.58-22.2) | 1.04 (0.94-1.15) |
| Marshall Islands | 196 (165-230) | 25.44 (21.61-29.44) | 788 (682-900) | 40.12 (35.01-45.5) | 1.57 (1.5-1.64) |
| Mauritania | 13927 (12391-15711) | 31.25 (27.98-35.06) | 61210 (56686-65363) | 46.24 (42.93-49.43) | 1.36 (1.28-1.44) |
| Mauritius | 135 (112-159) | 12.02 (10.03-14.11) | 425 (349-493) | 16.3 (13.72-18.66) | 0.94 (0.86-1.01) |
| Mexico | 116 (100-136) | 18.43 (15.94-21.51) | 285 (246-330) | 27.71 (24.05-31.73) | 1.51 (1.44-1.58) |
| Micronesia (Federated States of) | 3082 (2676-3557) | 21.54 (18.68-24.75) | 14044 (12221-16083) | 39.84 (34.81-45.23) | 1.97 (1.82-2.13) |
| Monaco | 516 (439-597) | 8.87 (7.62-10.23) | 1223 (1046-1428) | 11.22 (9.76-12.95) | 0.65 (0.49-0.81) |
| Mongolia | 3523 (3020-4123) | 14.66 (12.7-16.84) | 10655 (9207-12091) | 21.04 (18.28-23.87) | 1.18 (1.03-1.33) |
| Montenegro | 101 (87-120) | 15.28 (13.39-17.63) | 277 (240-324) | 19.79 (17.33-22.88) | 0.75 (0.56-0.95) |
| Morocco | 1222 (1042-1449) | 12.26 (10.56-14.27) | 5030 (4314-5817) | 20.45 (17.67-23.42) | 1.47 (1.4-1.55) |
| Mozambique | 4458 (3940-5037) | 21.23 (18.89-23.95) | 9025 (7866-10061) | 24.21 (21.16-26.9) | 0.59 (0.53-0.65) |
| Myanmar | 1112 (981-1284) | 27.16 (24.08-31.3) | 2695 (2380-3046) | 31.1 (27.6-35.22) | 0.48 (0.42-0.53) |
| Namibia | 471 (400-559) | 28.35 (24.27-33.05) | 2231 (1916-2565) | 42.59 (36.71-48.62) | 1.34 (1.28-1.39) |
| Nauru | 339 (284-394) | 12.14 (10.48-13.81) | 1238 (1054-1443) | 14.75 (12.7-16.99) | 0.64 (0.57-0.71) |
| Nepal | 5779 (5144-6466) | 12.98 (11.61-14.46) | 15856 (14146-17503) | 17.43 (15.54-19.26) | 0.93 (0.79-1.08) |
| Netherlands | 5 (4-6) | 24.52 (21.04-28.63) | 20 (17-24) | 33.77 (28.89-39.77) | 0.85 (0.72-0.99) |
| New Zealand | 1309 (1153-1492) | 17.9 (15.93-20.09) | 2126 (1912-2349) | 20.36 (18.45-22.37) | 0.51 (0.48-0.54) |
| Nicaragua | 151 (131-173) | 22.7 (19.73-25.91) | 865 (745-986) | 42.61 (37.37-48.21) | 1.97 (1.78-2.16) |
| Niger | 7999 (7094-9017) | 13.68 (12.14-15.4) | 21609 (19073-24238) | 16.82 (14.96-18.78) | 0.8 (0.77-0.84) |
| Nigeria | 262 (224-307) | 29.58 (25.76-34.39) | 1189 (1030-1372) | 45.19 (39.94-51.33) | 1.26 (1.14-1.37) |
| Niue | 387 (329-453) | 25.45 (21.65-29.69) | 1644 (1434-1862) | 37.35 (32.54-42.32) | 1.33 (1.28-1.37) |
| North Macedonia | 176 (145-215) | 9.02 (7.66-10.58) | 646 (537-760) | 11.93 (10.11-13.87) | 0.88 (0.7-1.05) |
| Northern Mariana Islands | 444 (384-509) | 19.7 (17.14-22.55) | 1864 (1639-2127) | 31.22 (27.64-35.47) | 1.55 (1.48-1.61) |
| Norway | 1781 (1513-2057) | 15.01 (12.78-17.38) | 9585 (8189-10937) | 28.91 (24.66-33.15) | 2.44 (2.34-2.54) |
| Oman | 5490 (4947-6021) | 17.99 (16.27-19.76) | 22328 (20228-24217) | 25.73 (23.55-27.82) | 1.11 (1.07-1.15) |
| Pakistan | 592 (516-681) | 18.29 (16.02-20.93) | 2493 (2182-2862) | 26.91 (23.73-30.83) | 1.42 (1.37-1.47) |
| Palau | 6544 (5684-7484) | 14.69 (12.78-16.8) | 14663 (12969-16660) | 20.19 (18.05-22.63) | 0.36 (0.14-0.57) |
| Palestine | 3176 (2692-3739) | 21.74 (18.53-25.28) | 6151 (5351-7184) | 24.15 (20.89-28.01) | 0.46 (0.4-0.52) |
| Panama | 15 (13-18) | 20.65 (17.79-23.87) | 23 (20-26) | 22.65 (19.89-25.73) | 0.4 (0.36-0.43) |
| Papua New Guinea | 848 (734-986) | 23.3 (20.09-27.12) | 2271 (1987-2587) | 35.84 (31.61-40.52) | 1.5 (1.31-1.69) |
| Paraguay | 40 (34-48) | 32.96 (28.12-38.55) | 587 (497-689) | 53.08 (46.26-60.12) | 1.39 (1.31-1.46) |
| Peru | 25 (22-29) | 10.96 (9.47-12.52) | 75 (64-87) | 17.32 (14.85-19.93) | 1.54 (1.39-1.68) |
| Philippines | 597 (489-700) | 14.78 (12.72-16.86) | 2116 (1782-2464) | 18.81 (16.27-21.34) | 0.66 (0.55-0.77) |
| Poland | 6234 (5344-7363) | 22 (18.99-25.77) | 24487 (21866-27473) | 25.36 (22.69-28.38) | 0.54 (0.44-0.65) |
| Portugal | 269 (230-318) | 6.05 (5.22-7.09) | 640 (538-763) | 10.9 (9.27-12.97) | 1.77 (1.55-1.99) |
| Puerto Rico | 1 (1-1) | 17.74 (15.3-20.55) | 1 (1-2) | 24.02 (20.76-27.69) | 0.91 (0.82-1.01) |
| Qatar | 0 (0-0) | 17.98 (15.26-20.76) | 1 (1-1) | 27.07 (22.93-31.28) | 1.29 (1.23-1.35) |
| Republic of Korea | 2 (2-3) | 20.91 (17.55-24.56) | 9 (7-10) | 34.19 (29.89-39.45) | 1.58 (1.44-1.72) |
| Republic of Moldova | 7 (6-8) | 19.27 (16.75-22.01) | 15 (13-18) | 20.47 (17.7-23.58) | 0.25 (0.2-0.3) |
| Romania | 47 (41-55) | 13.14 (11.63-14.88) | 167 (146-193) | 17.09 (14.96-19.61) | 0.8 (0.67-0.94) |
| Russian Federation | 3174 (2710-3712) | 11.04 (9.51-12.84) | 7845 (6893-8837) | 21.36 (18.93-23.89) | 2.22 (2.03-2.41) |
| Rwanda | 17142 (15089-19302) | 9.41 (8.37-10.51) | 40190 (36171-44622) | 17.17 (15.51-18.82) | 2 (1.94-2.06) |
| Saint Kitts and Nevis | 251 (214-297) | 8.99 (7.8-10.44) | 717 (615-837) | 11.51 (10.02-13.3) | 0.8 (0.66-0.93) |
| Saint Lucia | 8 (7-9) | 22.24 (18.98-25.3) | 27 (23-31) | 34.48 (30.14-39.79) | 1.49 (1.34-1.65) |
| Saint Vincent and the Grenadines | 18 (15-21) | 19.95 (17.32-22.91) | 76 (66-88) | 30.98 (26.97-35.69) | 1.39 (1.27-1.52) |
| Samoa | 13 (12-15) | 18.51 (15.97-21.28) | 42 (37-48) | 28.66 (24.98-32.44) | 1.43 (1.34-1.53) |
| San Marino | 17 (14-20) | 18.83 (16.07-22.04) | 40 (34-46) | 26.31 (22.88-30.39) | 1.02 (0.98-1.06) |
| Sao Tome and Principe | 10 (9-12) | 15.23 (13.08-17.79) | 26 (22-30) | 22.57 (19.8-25.82) | 1.33 (1.23-1.42) |
| Saudi Arabia | 1897 (1658-2204) | 31.37 (27.52-35.6) | 11475 (9985-13127) | 52.8 (47.08-58.92) | 1.59 (1.46-1.71) |
| Senegal | 435 (377-502) | 13.27 (11.61-15) | 1303 (1128-1497) | 16.68 (14.64-18.97) | 0.67 (0.63-0.7) |
| Serbia | 1698 (1455-1954) | 14.01 (12.24-15.91) | 4314 (3741-4964) | 25.78 (22.45-29.38) | 2.28 (2.15-2.42) |
| Seychelles | 13 (11-14) | 22.04 (19.08-25.04) | 40 (34-46) | 32.25 (28.2-36.81) | 1.2 (1.09-1.32) |
| Sierra Leone | 255 (217-294) | 12.22 (10.45-14) | 593 (508-669) | 15.64 (13.58-17.62) | 0.78 (0.61-0.94) |
| Singapore | 597 (538-660) | 27.39 (24.78-30.14) | 2776 (2453-3158) | 31.83 (28.4-36.05) | 0.61 (0.55-0.66) |
| Slovakia | 862 (736-996) | 14.24 (12.19-16.45) | 2243 (1945-2549) | 23 (19.96-26.03) | 1.55 (1.47-1.62) |
| Slovenia | 318 (273-369) | 12.87 (11.05-14.95) | 1000 (854-1145) | 22.91 (19.64-26.11) | 1.98 (1.81-2.14) |
| Solomon Islands | 5308 (4548-6108) | 13.04 (11.27-14.98) | 21256 (18212-24657) | 20.9 (18.16-24.02) | 1.76 (1.69-1.82) |
| Somalia | 21 (17-26) | 13.88 (11.82-16.18) | 57 (48-67) | 15.17 (13.07-17.39) | 0.04 (-0.11-0.19) |
| South Africa | 208 (176-242) | 9.18 (7.98-10.46) | 635 (539-739) | 10.61 (9.16-12.05) | 0.36 (0.25-0.47) |
| South Sudan | 3492 (3122-3891) | 16.83 (15.11-18.69) | 11519 (10409-12561) | 24.33 (21.97-26.45) | 1.12 (0.99-1.25) |
| Spain | 252 (219-292) | 9.8 (8.57-11.26) | 441 (379-511) | 11.82 (10.21-13.62) | 0.49 (0.38-0.6) |
| Sri Lanka | 14143 (12448-16111) | 24.71 (21.86-27.98) | 23658 (20872-26718) | 23.64 (20.9-26.67) | -0.03 (-0.1-0.05) |
| Sudan | 2128 (1849-2471) | 19.22 (16.76-22.18) | 8187 (7002-9322) | 28.58 (24.54-32.4) | 1.35 (1.3-1.41) |
| Suriname | 2112 (1797-2461) | 22.47 (19.24-25.88) | 6793 (5910-7802) | 34.98 (30.58-39.62) | 1.36 (1.13-1.59) |
| Sweden | 50 (43-58) | 18.75 (16.05-21.49) | 195 (168-225) | 29.71 (25.78-34.16) | 1.63 (1.55-1.71) |
| Switzerland | 2856 (2477-3295) | 17.5 (15.2-20.09) | 3891 (3355-4466) | 17.3 (15.1-19.57) | 0.09 (0.04-0.13) |
| Syrian Arab Republic | 2619 (2284-2986) | 24.2 (21.17-27.53) | 4891 (4217-5594) | 26.4 (22.58-30.4) | 0.38 (0.31-0.44) |
| Taiwan (Province of China) | 1431 (1207-1673) | 27.06 (23.25-31.37) | 5883 (5170-6684) | 42.1 (37.44-47.25) | 1.38 (1.3-1.46) |
| Tajikistan | 4574 (4087-5121) | 28 (25.17-31.08) | 13540 (12194-15028) | 31.39 (28.35-34.68) | 0.34 (0.26-0.42) |
| Thailand | 186 (156-223) | 6.49 (5.49-7.72) | 711 (596-846) | 11.41 (9.76-13.14) | 2.17 (1.93-2.41) |
| Timor-Leste | 7608 (6472-8795) | 20.68 (17.77-23.73) | 34553 (30207-39095) | 30.95 (27.18-34.85) | 1.24 (1.12-1.35) |
| Togo | 263 (228-305) | 13.65 (11.9-15.7) | 959 (830-1097) | 26.83 (23.31-30.44) | 2.36 (2.13-2.58) |
| Tokelau | 43 (36-51) | 14.44 (12.3-16.68) | 176 (153-205) | 19.83 (17.25-22.93) | 1.12 (1-1.24) |
| Tonga | 163 (140-193) | 13.08 (11.29-15.12) | 648 (557-755) | 16.73 (14.5-19.43) | 0.67 (0.52-0.81) |
| Trinidad and Tobago | 0 (0-0) | 15.2 (13.07-17.75) | 0 (0-0) | 24.11 (20.84-27.96) | 1.44 (1.36-1.51) |
| Tunisia | 11 (9-13) | 18.28 (15.8-21.16) | 21 (18-24) | 25.96 (22.58-29.66) | 1.08 (1.04-1.12) |
| Türkiye | 158 (137-181) | 18.31 (15.88-21.06) | 611 (536-697) | 30.69 (27.1-34.73) | 1.81 (1.72-1.9) |
| Turkmenistan | 1360 (1176-1566) | 26.28 (22.96-30.17) | 5852 (5121-6677) | 42.62 (37.57-48.49) | 1.48 (1.39-1.57) |
| Tuvalu | 7820 (6938-8868) | 22.92 (20.5-25.77) | 41374 (36219-47068) | 42.69 (37.59-48.08) | 1.98 (1.91-2.05) |
| Uganda | 190 (160-229) | 9.12 (7.78-10.83) | 643 (548-757) | 14.43 (12.37-16.78) | 1.64 (1.47-1.82) |
| Ukraine | 1 (1-1) | 14.1 (12.05-16.58) | 3 (2-3) | 23.05 (19.78-26.49) | 1.55 (1.5-1.59) |
| United Arab Emirates | 543 (473-624) | 8.6 (7.53-9.85) | 1480 (1258-1690) | 10.43 (9.01-11.87) | 0.5 (0.33-0.68) |
| United Kingdom | 5039 (4249-5980) | 7.03 (5.98-8.36) | 7825 (6796-9053) | 10.31 (8.93-11.87) | 1.23 (1.14-1.33) |
| United Republic of Tanzania | 172 (146-206) | 35.2 (30.73-40.61) | 3008 (2555-3635) | 51.84 (45.85-58.7) | 1.06 (0.94-1.17) |
| United States of America | 20115 (18086-22687) | 20.86 (18.81-23.19) | 30215 (27275-33284) | 22.43 (20.29-24.64) | -0.01 (-0.1-0.09) |
| United States Virgin Islands | 1070 (917-1235) | 9.89 (8.54-11.39) | 3092 (2687-3531) | 12.44 (10.83-14.12) | 0.63 (0.53-0.73) |
| Uruguay | 99641 (89218-110736) | 30.61 (27.37-33.79) | 198284 (180729-216564) | 33.28 (30.52-36.21) | 0.15 (0.06-0.24) |
| Uzbekistan | 18 (15-21) | 20.11 (17.26-23.34) | 56 (48-64) | 31.28 (26.96-36.03) | 1.49 (1.37-1.61) |
| Vanuatu | 811 (702-925) | 20.03 (17.44-22.77) | 1359 (1197-1535) | 24.09 (21.28-27.26) | 0.7 (0.64-0.75) |
| Venezuela (Bolivarian Republic of) | 1419 (1171-1693) | 11.69 (9.76-13.92) | 5138 (4246-6105) | 17.65 (14.82-20.4) | 1.36 (1.22-1.49) |
| Viet Nam | 9 (8-11) | 13.9 (11.91-15.98) | 38 (31-43) | 20.4 (17.43-23.26) | 1.15 (1.08-1.23) |
| Yemen | 1068 (895-1252) | 21.59 (18.24-25.08) | 4384 (3763-5034) | 31.24 (27.06-35.8) | 1.18 (0.96-1.41) |
| Zambia | 300 (257-345) | 10.65 (9.3-12.06) | 915 (780-1054) | 13.38 (11.56-15.3) | 0.67 (0.49-0.84) |
| Zimbabwe | 668 (576-777) | 16.09 (13.95-18.5) | 1312 (1140-1503) | 18.6 (16.2-21.1) | 0.18 (-0.03-0.39) |

| **TableS20: National Burden of chronic kidney disease due to diabetes mellitus type 2: prevalence cases, ASPR, and EAPC (1990–2021).** | | | | | |
| --- | --- | --- | --- | --- | --- |
| Country | **1990** | | **2021** | | **EAPC_95%CI** |
|  | Number_95%UI | ASR | Number_95%UI | ASR |  |
| Afghanistan | 106060 (92495-120612) | 1527.26 (1336.6-1741.08) | 214601 (183938-251640) | 1462.58 (1289.83-1673.58) | -0.17 (-0.19--0.15) |
| Albania | 22626 (19721-25915) | 946.12 (832.47-1064.77) | 34278 (30597-38222) | 860.61 (762.91-967.87) | -0.32 (-0.33--0.3) |
| Algeria | 242410 (206940-282845) | 1571.96 (1368.63-1786.42) | 588745 (510342-683237) | 1460.31 (1277.31-1669.32) | -0.34 (-0.37--0.3) |
| American Samoa | 500 (425-588) | 1619.74 (1418.16-1847.34) | 766 (680-865) | 1548.45 (1376.28-1745.57) | -0.17 (-0.18--0.16) |
| Andorra | 532 (472-598) | 877.76 (778.3-981.51) | 1119 (991-1245) | 793.41 (701.75-885.03) | -0.35 (-0.38--0.32) |
| Angola | 75844 (65278-88276) | 1462.14 (1295.7-1644.01) | 212646 (184953-245636) | 1364.72 (1204.62-1528.33) | -0.26 (-0.28--0.25) |
| Antigua and Barbuda | 604 (524-681) | 1109.82 (964.4-1252.91) | 1184 (1039-1338) | 1097.83 (967.51-1235.89) | -0.06 (-0.07--0.04) |
| Argentina | 268633 (236956-303879) | 837.17 (737.74-948.96) | 481398 (423884-547350) | 897.55 (786.43-1023.94) | 0.29 (0.25-0.32) |
| Armenia | 45864 (40691-52572) | 1554.46 (1387.33-1752.39) | 58886 (53199-65282) | 1447.73 (1303.86-1610.26) | -0.29 (-0.32--0.27) |
| Australia | 158986 (142280-176265) | 815.65 (727.37-906.37) | 312048 (281621-344763) | 749.65 (672.16-829.52) | -0.23 (-0.3--0.16) |
| Austria | 89410 (79677-99743) | 813.7 (722.32-916.08) | 123312 (111328-136850) | 772.27 (692.94-863.75) | -0.16 (-0.18--0.13) |
| Azerbaijan | 85045 (74642-96919) | 1532.98 (1367.22-1729.86) | 161988 (143523-183902) | 1479 (1323.28-1670.53) | -0.15 (-0.17--0.14) |
| Bahamas | 5588 (4583-6718) | 1644.64 (1432.46-1853.23) | 22073 (18890-25321) | 1550.82 (1357.11-1743.69) | -0.23 (-0.24--0.21) |
| Bahrain | 887498 (756971-1033070) | 1427.82 (1254.76-1607.64) | 2044421 (1783301-2320118) | 1363.34 (1195.84-1539.45) | -0.25 (-0.3--0.21) |
| Bangladesh | 2962 (2642-3350) | 1070.41 (954.61-1211.74) | 4831 (4324-5401) | 1061.62 (945.01-1197.3) | -0.05 (-0.07--0.03) |
| Barbados | 193028 (169461-219718) | 1582.47 (1383.11-1812.72) | 204588 (181795-229298) | 1437.81 (1265.2-1616.98) | -0.4 (-0.43--0.38) |
| Belarus | 124797 (110837-137751) | 882.46 (776.7-987.39) | 163817 (147075-181564) | 827.75 (738.8-930.03) | -0.18 (-0.2--0.17) |
| Belgium | 1199 (1032-1381) | 1100.16 (963.91-1241.78) | 3783 (3311-4314) | 1099.54 (966.06-1238.32) | -0.03 (-0.04--0.02) |
| Belize | 30425 (26125-35346) | 1221.22 (1088.94-1378.54) | 81151 (70923-94351) | 1184.36 (1054.37-1333.71) | -0.12 (-0.15--0.09) |
| Benin | 691 (606-790) | 1048.2 (927.25-1191.11) | 1105 (979-1244) | 987.49 (874.41-1117.7) | -0.23 (-0.26--0.21) |
| Bermuda | 5188 (4378-6086) | 1505.5 (1322.18-1692.57) | 9679 (8440-10998) | 1412.36 (1252.96-1592.37) | -0.24 (-0.26--0.23) |
| Bhutan | 172690 (148690-196251) | 1429.66 (1264.7-1585.74) | 387706 (344126-427869) | 1296.2 (1146.84-1433.51) | -0.36 (-0.41--0.3) |
| Bolivia (Plurinational State of) | 38235 (34000-42667) | 889.29 (795.18-991.11) | 48775 (43269-54152) | 873.28 (775.32-972.3) | -0.05 (-0.06--0.04) |
| Bosnia and Herzegovina | 9239 (7950-10742) | 1343.28 (1182.34-1519.18) | 24032 (20704-27490) | 1328.26 (1168.01-1488.99) | -0.06 (-0.07--0.05) |
| Botswana | 1325115 (1196378-1451363) | 1267.25 (1154.24-1372.74) | 2886922 (2647320-3121112) | 1144.93 (1052.7-1235.54) | -0.37 (-0.38--0.35) |
| Brazil | 2310 (1999-2725) | 1549.2 (1381.2-1734.12) | 6293 (5526-7171) | 1496.78 (1332.34-1665.65) | -0.16 (-0.18--0.14) |
| Brunei Darussalam | 103847 (93230-116362) | 894.5 (801.6-1003.62) | 105861 (96670-116902) | 837.63 (761.04-931.52) | -0.23 (-0.24--0.22) |
| Bulgaria | 58352 (50415-66955) | 1153.36 (1012.88-1301.65) | 131701 (114141-152116) | 1123.57 (996.51-1260.84) | -0.11 (-0.13--0.1) |
| Burkina Faso | 30022 (25421-35236) | 992.47 (869.74-1131.82) | 67199 (57413-78402) | 971.13 (855.83-1106.14) | -0.11 (-0.13--0.1) |
| Burundi | 93484 (79535-108855) | 1623.9 (1405.74-1846.69) | 226660 (194730-260659) | 1577.4 (1373.25-1785.21) | -0.14 (-0.17--0.12) |
| C?te d'Ivoire | 72149 (62636-82791) | 1324.56 (1174.93-1483.38) | 226315 (199403-257037) | 1384.21 (1243.24-1537.69) | 0.18 (0.07-0.29) |
| Cabo Verde | 241220 (215821-274588) | 746.5 (667.88-850.26) | 524389 (467798-587911) | 812.44 (717.15-918.79) | 0.35 (0.29-0.41) |
| Cambodia | 21276 (18346-24703) | 1468.18 (1297.48-1644.71) | 39619 (34410-46082) | 1372.12 (1220.51-1536.72) | -0.25 (-0.26--0.24) |
| Cameroon | 39844 (34552-44909) | 1202.7 (1069.2-1340.74) | 88096 (76611-100190) | 1152.97 (1026.52-1280.1) | -0.17 (-0.18--0.16) |
| Canada | 111415 (97397-127938) | 1000.77 (888.5-1130.99) | 227450 (202147-257341) | 939.35 (833.88-1071.88) | -0.22 (-0.23--0.21) |
| Central African Republic | 11890522 (10790476-13078708) | 1214.76 (1109.34-1320.7) | 20911520 (19184463-22605470) | 1053.92 (971.11-1139.64) | -0.24 (-0.39--0.1) |
| Chad | 287027 (249464-330939) | 1327.25 (1178.71-1490.32) | 664030 (594165-740653) | 1212.7 (1084.65-1352.83) | -0.28 (-0.3--0.25) |
| Chile | 2114 (1803-2461) | 1107.53 (965.44-1253.22) | 4595 (4001-5172) | 1081.37 (948-1219.4) | -0.09 (-0.1--0.08) |
| China | 2608 (2194-3069) | 1025.12 (892.7-1170.69) | 5686 (4899-6565) | 976.48 (852.8-1108.36) | -0.2 (-0.22--0.18) |
| Colombia | 19252 (16638-22391) | 1476.58 (1314.16-1655.43) | 48738 (42611-56085) | 1412.08 (1258.88-1584.74) | -0.19 (-0.2--0.17) |
| Comoros | 214 (185-244) | 1478.8 (1296.92-1661) | 324 (286-364) | 1395.4 (1229.2-1563.54) | -0.22 (-0.23--0.21) |
| Congo | 32279 (28194-36476) | 1568.16 (1399.69-1737.37) | 78947 (70882-87440) | 1448.79 (1299.65-1603.6) | -0.2 (-0.23--0.18) |
| Cook Islands | 55134 (49187-61951) | 932.16 (825.46-1045.65) | 64856 (58203-72356) | 836 (744.2-940.11) | -0.38 (-0.39--0.37) |
| Costa Rica | 118805 (103057-136162) | 1104.96 (963.7-1262.12) | 172152 (153766-192179) | 1005.72 (888.55-1134.4) | -0.35 (-0.39--0.32) |
| Croatia | 7915 (7053-8887) | 954.4 (853.61-1073.39) | 16601 (14899-18612) | 826.6 (738.92-929.61) | -0.51 (-0.53--0.5) |
| Cuba | 119541 (107519-132879) | 922.36 (826.25-1031.35) | 152955 (138276-168818) | 811.6 (727.48-900.62) | -0.39 (-0.43--0.36) |
| Cyprus | 204638 (176799-233204) | 1139.93 (991.86-1289.01) | 342607 (300189-386124) | 1045.53 (918.41-1177.73) | -0.38 (-0.41--0.35) |
| Czechia | 292383 (252460-337905) | 1487.82 (1319.24-1657.78) | 647909 (564592-740765) | 1377.98 (1241.27-1530.46) | -0.35 (-0.38--0.32) |
| Democratic People's Republic of Korea | 60096 (53340-66836) | 809.75 (713.4-909.06) | 79965 (71881-88163) | 775.24 (686.3-866.96) | -0.22 (-0.25--0.18) |
| Democratic Republic of the Congo | 1993 (1616-2375) | 924.74 (789.19-1045.69) | 8466 (7062-9849) | 942.61 (805.58-1065.71) | 0.03 (0.02-0.05) |
| Denmark | 701 (612-796) | 1163.29 (1018.39-1315.51) | 928 (821-1037) | 1144.46 (1010.56-1282.74) | -0.07 (-0.09--0.05) |
| Djibouti | 50710 (43332-59439) | 1097.88 (965.58-1247.92) | 113995 (99857-129505) | 1082.8 (952.82-1226.45) | -0.03 (-0.05-0) |
| Dominica | 66333 (57940-77374) | 1016.76 (911.88-1155.79) | 170654 (152197-192979) | 998.85 (891.46-1126.47) | -0.01 (-0.05-0.03) |
| Dominican Republic | 490136 (420423-570364) | 1425.87 (1261.72-1626.34) | 1168674 (1018840-1342175) | 1509.01 (1339.24-1721.09) | 0.24 (0.2-0.28) |
| Ecuador | 40245 (34877-46605) | 1178.97 (1039.31-1334.48) | 71168 (63261-80227) | 1150.89 (1022.75-1295.2) | 0.03 (-0.01-0.08) |
| Egypt | 3354 (2914-3844) | 1456.42 (1289.37-1634.95) | 10750 (9231-12451) | 1431.1 (1271.21-1607.75) | -0.07 (-0.08--0.06) |
| El Salvador | 15971 (13457-18991) | 941.21 (824.16-1073.2) | 37145 (31613-43731) | 932.27 (815.69-1064.56) | -0.05 (-0.07--0.04) |
| Equatorial Guinea | 28882 (25412-32727) | 1506.25 (1315.78-1712.79) | 31279 (27830-34625) | 1414.68 (1250.29-1575.51) | -0.22 (-0.25--0.19) |
| Eritrea | 251707 (225092-284392) | 955.38 (865.4-1054.02) | 557970 (502100-632388) | 930.85 (848.74-1030.01) | -0.08 (-0.11--0.06) |
| Estonia | 880 (763-1033) | 1457.08 (1298.03-1650.49) | 1195 (1059-1356) | 1415 (1272.39-1597.4) | -0.11 (-0.12--0.09) |
| Eswatini | 7576 (6328-8767) | 1524.53 (1315.5-1725.12) | 12510 (10731-14230) | 1478.78 (1274.26-1670.46) | -0.1 (-0.12--0.08) |
| Ethiopia | 52539 (46728-59310) | 779.64 (689.51-886.59) | 73979 (67050-82023) | 697.82 (623.11-779.07) | -0.29 (-0.35--0.24) |
| Fiji | 500524 (444365-555709) | 655.4 (575.76-733.11) | 734737 (660803-815653) | 622.22 (554.3-695.53) | -0.17 (-0.26--0.08) |
| Finland | 9167 (8002-10475) | 1442.41 (1269.73-1621.8) | 17153 (15041-19460) | 1388.03 (1232.81-1549.73) | -0.15 (-0.17--0.14) |
| France | 96241 (85492-107644) | 1588.85 (1413.19-1774.79) | 78720 (71076-86675) | 1493.45 (1344.48-1657.6) | -0.23 (-0.25--0.2) |
| Gabon | 935922 (842335-1030960) | 799.13 (715.16-888.45) | 1245910 (1137851-1359608) | 741.09 (674.06-816.57) | -0.3 (-0.42--0.19) |
| Gambia | 90845 (76632-106143) | 1062.95 (926.57-1201.94) | 223569 (192823-258569) | 1002.46 (880.73-1128.62) | -0.22 (-0.24--0.21) |
| Georgia | 130011 (115904-145269) | 926.53 (817.43-1036.43) | 165047 (149279-183060) | 827.61 (740.86-923.43) | -0.41 (-0.43--0.39) |
| Germany | 269 (227-309) | 766.25 (666.27-857.75) | 672 (593-745) | 993.45 (879.15-1097.62) | 0.89 (0.86-0.92) |
| Ghana | 827 (725-934) | 1180.29 (1031.49-1333.51) | 1341 (1174-1511) | 1161.03 (1014.55-1305.33) | -0.07 (-0.09--0.05) |
| Greece | 1528 (1313-1808) | 1454.62 (1271.71-1661.04) | 2733 (2398-3099) | 1411.94 (1232.69-1601.47) | -0.1 (-0.11--0.08) |
| Greenland | 54808 (47927-62544) | 1301.03 (1155.68-1452.8) | 163036 (143806-183693) | 1347.35 (1196.96-1509.38) | 0.19 (0.15-0.22) |
| Grenada | 44656 (39099-50836) | 1199.04 (1063.85-1347.37) | 83062 (71865-95451) | 1175.85 (1041.27-1320.01) | -0.06 (-0.09--0.04) |
| Guam | 6407 (5453-7439) | 1256.67 (1107.39-1405.66) | 12266 (10491-14132) | 1195.69 (1054.05-1342.7) | -0.19 (-0.2--0.18) |
| Guatemala | 5699 (4936-6704) | 1163.23 (1029.52-1312.63) | 7803 (6929-8813) | 1126.01 (1006.41-1260.23) | -0.12 (-0.13--0.11) |
| Guinea | 45587 (39402-52314) | 1169.9 (1028.85-1313.11) | 102643 (88945-117366) | 1142.09 (1010.16-1288.74) | -0.09 (-0.1--0.08) |
| Guinea-Bissau | 35031 (30258-40128) | 1404.06 (1243.42-1560.53) | 96889 (85710-108728) | 1334 (1191.21-1484.62) | -0.17 (-0.18--0.15) |
| Guyana | 129770 (115269-145763) | 953.72 (844.54-1083.46) | 142228 (126988-157141) | 831.7 (742.63-930.43) | -0.49 (-0.51--0.46) |
| Haiti | 2043 (1836-2301) | 731.81 (655.16-823.33) | 3439 (3105-3863) | 672.2 (604.7-760.69) | -0.33 (-0.37--0.29) |
| Honduras | 10452093 (9353027-11529934) | 1777.93 (1615.96-1940.55) | 20825525 (18968515-22827604) | 1586.69 (1450.73-1726.7) | -0.48 (-0.57--0.4) |
| Hungary | 2287824 (2034136-2559184) | 1815.91 (1644.47-1998.01) | 4884349 (4426931-5419195) | 1774.63 (1619.37-1945.78) | -0.25 (-0.34--0.16) |
| Iceland | 178843 (151774-208204) | 1711.61 (1500.13-1941.35) | 482687 (419822-558918) | 1585.59 (1395.3-1795.76) | -0.29 (-0.31--0.27) |
| India | 42816 (37843-48308) | 1079.44 (953.07-1223.87) | 67783 (61352-75418) | 921.82 (829.32-1032.29) | -0.51 (-0.54--0.48) |
| Indonesia | 495894 (447704-543796) | 1537.9 (1408.15-1670.43) | 1268663 (1166386-1386761) | 1466.48 (1345.09-1586.75) | -0.16 (-0.19--0.13) |
| Iran (Islamic Republic of) | 46014 (40798-51512) | 950.34 (842.99-1066.94) | 97935 (87645-109483) | 851.27 (761.97-949.74) | -0.41 (-0.43--0.39) |
| Iraq | 665908 (607390-716988) | 815.03 (740.6-882.61) | 789150 (722681-853244) | 646.37 (585.94-704.15) | -0.6 (-0.68--0.52) |
| Ireland | 19625 (17140-22258) | 1062.83 (932.15-1186.48) | 32935 (29228-37033) | 1067.52 (947.28-1201.06) | 0 (-0.03-0.03) |
| Israel | 2409618 (2218436-2607887) | 1490.11 (1367.35-1613.04) | 3529802 (3243067-3800842) | 1326.15 (1221.21-1429.23) | -0.39 (-0.48--0.31) |
| Italy | 31163 (26516-36873) | 1623.2 (1413.45-1850.99) | 144069 (124295-165022) | 1501.73 (1310.04-1700.95) | -0.32 (-0.35--0.29) |
| Jamaica | 225747 (197993-256476) | 1633.29 (1447.67-1824.75) | 289296 (256707-322890) | 1542.49 (1364.54-1715.7) | -0.22 (-0.23--0.21) |
| Japan | 110296 (97469-124012) | 969.44 (880.32-1056.91) | 285172 (258001-315222) | 936.19 (856.73-1016.15) | -0.16 (-0.18--0.14) |
| Jordan | 5001 (4301-5767) | 1345.29 (1185.9-1498.72) | 9390 (8136-10747) | 1335.47 (1182.96-1494.06) | -0.03 (-0.05--0.02) |
| Kazakhstan | 708 (598-818) | 1489.37 (1291.13-1676.61) | 1250 (1078-1419) | 1418.72 (1243.26-1597.71) | -0.16 (-0.18--0.15) |
| Kenya | 18900 (15667-22439) | 1633.38 (1421.88-1846.89) | 65351 (56093-75890) | 1455.36 (1282.27-1647.54) | -0.41 (-0.43--0.39) |
| Kiribati | 50941 (44865-57365) | 1561.02 (1393.04-1738.51) | 76571 (68164-86252) | 1412.98 (1268.96-1579) | -0.38 (-0.41--0.36) |
| Kuwait | 46863 (39905-54446) | 1861.48 (1613.36-2115.7) | 100673 (86119-116587) | 1757.27 (1528.38-1999.86) | -0.23 (-0.26--0.21) |
| Kyrgyzstan | 52181 (45142-59317) | 1586.53 (1363.07-1812.08) | 46903 (40964-52334) | 1469.31 (1280.82-1669.21) | -0.27 (-0.28--0.25) |
| Lao People's Democratic Republic | 37300 (32050-42967) | 1554.03 (1353.54-1785.02) | 90584 (79133-103802) | 1496.72 (1305.25-1718.14) | -0.12 (-0.16--0.08) |
| Latvia | 11647 (10279-13150) | 1247.89 (1107.58-1403.73) | 16175 (14227-18303) | 1290.77 (1155.94-1443.64) | 0.13 (0.12-0.14) |
| Lebanon | 17929 (15534-20456) | 1273.16 (1132.22-1423.23) | 36350 (31440-41752) | 1204.31 (1077.3-1340.91) | -0.18 (-0.19--0.17) |
| Lesotho | 39733 (34187-46928) | 1619.23 (1420.22-1835.86) | 101249 (88811-117423) | 1555.01 (1378.98-1755.88) | -0.13 (-0.15--0.11) |
| Liberia | 67594 (59496-76800) | 1578.91 (1384.61-1815.5) | 67896 (60625-76546) | 1465.08 (1297.5-1655.79) | -0.27 (-0.29--0.24) |
| Libya | 4811 (4295-5369) | 934.51 (830.82-1046.69) | 8219 (7365-9201) | 849.21 (756.51-952.34) | -0.31 (-0.34--0.28) |
| Lithuania | 63150 (52984-73150) | 960.56 (837.36-1092.12) | 148500 (125776-172950) | 932.08 (811.71-1064.3) | -0.13 (-0.16--0.11) |
| Luxembourg | 48353 (41020-57400) | 953.86 (832.34-1103.84) | 96505 (82839-113466) | 957.56 (833.45-1096.35) | -0.04 (-0.06--0.02) |
| Madagascar | 231262 (196008-271639) | 1923.06 (1679.6-2205.23) | 578484 (496849-662324) | 1852.54 (1601.54-2110.34) | -0.01 (-0.07-0.05) |
| Malawi | 2160 (1801-2510) | 1847.91 (1583.14-2090.12) | 8624 (7288-10147) | 1747.03 (1495.03-1981.55) | -0.23 (-0.27--0.2) |
| Malaysia | 58770 (50983-67651) | 1222.66 (1087.96-1369.54) | 132144 (115493-151918) | 1164.9 (1044.04-1308.84) | -0.19 (-0.21--0.17) |
| Maldives | 3630 (3228-4040) | 859.21 (763.4-958.07) | 6731 (6005-7473) | 800.78 (716.36-894.65) | -0.25 (-0.29--0.21) |
| Mali | 337 (283-397) | 1471.72 (1285.32-1674.77) | 612 (524-698) | 1407.96 (1235.7-1586.33) | -0.16 (-0.17--0.15) |
| Malta | 14626 (12732-17029) | 1223.38 (1088.73-1395.86) | 29193 (25726-33519) | 1135.44 (1017.33-1287.87) | -0.3 (-0.33--0.27) |
| Marshall Islands | 16238 (13657-18775) | 1850.04 (1595.03-2097.27) | 32684 (28705-36888) | 1884.21 (1644.51-2141.81) | 0.08 (0.04-0.12) |
| Mauritania | 753539 (679699-827535) | 1449.96 (1332.22-1569.46) | 1802771 (1666162-1944522) | 1375.47 (1273.87-1478.82) | -0.15 (-0.17--0.13) |
| Mauritius | 21014 (18111-24046) | 1654.56 (1454.99-1847.86) | 40710 (35658-46184) | 1504.21 (1336.68-1680.88) | -0.35 (-0.37--0.33) |
| Mexico | 5875 (5214-6587) | 929.16 (827.09-1041.57) | 7985 (7123-8920) | 875.22 (779.37-983.23) | -0.22 (-0.23--0.2) |
| Micronesia (Federated States of) | 273424 (230180-322011) | 1564.76 (1354.81-1803.18) | 539970 (467286-625059) | 1462.29 (1273.02-1682.17) | -0.28 (-0.3--0.26) |
| Monaco | 73161 (62534-84402) | 973.69 (840.82-1101.47) | 153797 (131651-178657) | 998.91 (868.11-1137.61) | 0.06 (0.05-0.08) |
| Mongolia | 508236 (435109-589452) | 1811.82 (1583.77-2057.08) | 900462 (771782-1034310) | 1701.86 (1471.22-1937.95) | -0.22 (-0.23--0.2) |
| Montenegro | 10550 (9206-12067) | 1353.26 (1201.77-1511.38) | 20939 (18289-23926) | 1270.78 (1127.34-1421.12) | -0.24 (-0.25--0.22) |
| Morocco | 179066 (157602-206356) | 1594.71 (1429.64-1785.91) | 380852 (339136-433980) | 1514.44 (1358.39-1709.26) | -0.35 (-0.42--0.29) |
| Mozambique | 176475 (157812-196981) | 926.5 (823.88-1040.39) | 258815 (234764-286854) | 862.54 (771.06-965.12) | -0.43 (-0.51--0.35) |
| Myanmar | 34281 (30854-38236) | 888.48 (799.64-990.42) | 65967 (59664-72630) | 863.25 (779.38-956.87) | -0.06 (-0.13-0) |
| Namibia | 30330 (26011-35131) | 1573.66 (1396.62-1764.17) | 84954 (76030-95325) | 1575.13 (1415.29-1749.26) | -0.05 (-0.07--0.03) |
| Nauru | 42980 (36698-49720) | 1144.4 (1015.01-1272.53) | 115358 (100111-131142) | 1085.38 (972.74-1204.22) | -0.2 (-0.22--0.18) |
| Nepal | 749899 (688160-814042) | 1447.37 (1335.47-1551.84) | 1625850 (1503433-1770172) | 1413 (1304.58-1523.2) | -0.01 (-0.04-0.01) |
| Netherlands | 505 (420-596) | 1562.72 (1384.12-1743.79) | 819 (725-921) | 1494.21 (1329.81-1666.96) | -0.17 (-0.18--0.16) |
| New Zealand | 49434 (44882-53346) | 829.01 (748.84-901.17) | 66311 (60666-71917) | 767.27 (695.14-835.43) | -0.26 (-0.31--0.22) |
| Nicaragua | 15155 (12639-17831) | 1393.51 (1219.12-1580.56) | 52147 (44421-61120) | 1460.55 (1289.28-1662.14) | 0.14 (0.1-0.19) |
| Niger | 979609 (870675-1097996) | 1444.57 (1294.71-1594.58) | 2202472 (1971550-2458814) | 1411.97 (1273.91-1547.37) | -0.1 (-0.14--0.07) |
| Nigeria | 16920 (14432-19752) | 1559.7 (1368.44-1765.86) | 47853 (41154-55360) | 1441.06 (1272.97-1620.28) | -0.28 (-0.33--0.23) |
| Niue | 24002 (21039-27593) | 1393.98 (1232.76-1580.76) | 58149 (51577-65624) | 1322.53 (1173.44-1492.5) | -0.18 (-0.19--0.16) |
| North Macedonia | 32844 (27299-37972) | 1343.59 (1168.59-1509.8) | 88097 (74958-101091) | 1299.94 (1144.16-1456.93) | -0.11 (-0.13--0.09) |
| Northern Mariana Islands | 31817 (27636-36522) | 1232.38 (1088.69-1391.43) | 73846 (65263-83666) | 1176.18 (1042.53-1326.23) | -0.17 (-0.18--0.16) |
| Norway | 137703 (118675-160375) | 956.32 (840.89-1089.62) | 326874 (286519-374730) | 928.05 (816.9-1057.35) | -0.07 (-0.11--0.04) |
| Oman | 727091 (655237-806428) | 1849.56 (1691.95-2013) | 1613687 (1475734-1760668) | 1707.82 (1565.25-1847.94) | -0.35 (-0.41--0.3) |
| Pakistan | 40055 (34582-45904) | 1026.84 (904.57-1149.46) | 99610 (86945-113088) | 988.53 (871.87-1110.12) | -0.13 (-0.16--0.1) |
| Palau | 424168 (389294-457803) | 982.9 (902.46-1063.04) | 569201 (525192-617150) | 890.85 (815.48-963.21) | -0.4 (-0.43--0.36) |
| Palestine | 107655 (95619-119186) | 840.38 (748.52-936.79) | 145815 (130602-162176) | 733.89 (653.84-817.34) | -0.44 (-0.48--0.39) |
| Panama | 492 (441-551) | 853.85 (749.85-965.52) | 627 (566-697) | 792.05 (703.71-890.27) | -0.27 (-0.3--0.23) |
| Papua New Guinea | 40856 (36338-46172) | 1124.53 (1000.51-1270.46) | 62496 (56099-69389) | 1102.01 (978.2-1245.51) | -0.07 (-0.08--0.06) |
| Paraguay | 5132 (4185-6220) | 1678.66 (1486.21-1901.53) | 38763 (32760-46198) | 1553.98 (1375.01-1749.48) | -0.32 (-0.36--0.29) |
| Peru | 2925 (2536-3405) | 1239.68 (1096.72-1426.42) | 6007 (5244-6977) | 1188.63 (1049.96-1357.81) | -0.15 (-0.16--0.14) |
| Philippines | 75528 (64588-89489) | 1271.72 (1135.88-1431.65) | 186055 (161218-213429) | 1209.15 (1071.58-1353.5) | -0.2 (-0.21--0.18) |
| Poland | 444222 (381841-508892) | 1249.57 (1091.69-1402.51) | 941916 (836464-1053706) | 1110.95 (975.04-1243.34) | -0.59 (-0.65--0.52) |
| Portugal | 77160 (67991-87678) | 1723.77 (1521.97-1950.96) | 91045 (81294-101551) | 1669.11 (1482.46-1876.11) | -0.13 (-0.18--0.08) |
| Puerto Rico | 98 (83-115) | 1563.44 (1376.7-1777.7) | 111 (95-128) | 1506.14 (1324.17-1718.83) | -0.15 (-0.16--0.13) |
| Qatar | 33 (29-37) | 1560.7 (1359.07-1772.71) | 30 (26-34) | 1491.36 (1299.7-1684.5) | -0.17 (-0.18--0.16) |
| Republic of Korea | 184 (158-212) | 1522.33 (1325.79-1727.44) | 343 (298-391) | 1478.61 (1296.22-1677.24) | -0.11 (-0.12--0.1) |
| Republic of Moldova | 275 (243-306) | 853.03 (749.99-953.92) | 481 (428-539) | 778.8 (691.62-874.33) | -0.34 (-0.38--0.3) |
| Romania | 5990 (5072-7056) | 1223.87 (1068.76-1376.92) | 15263 (13096-17832) | 1184.16 (1049-1337.41) | -0.13 (-0.14--0.11) |
| Russian Federation | 243158 (215859-274030) | 907.18 (803.55-1025.1) | 279159 (253619-307034) | 840.29 (757.92-931.86) | -0.48 (-0.55--0.41) |
| Rwanda | 2598710 (2374569-2847906) | 1498.59 (1368.23-1637.44) | 2866519 (2611271-3133121) | 1347.69 (1238.41-1470.6) | -0.39 (-0.41--0.37) |
| Saint Kitts and Nevis | 36281 (30400-42924) | 964.34 (832.77-1090.82) | 73577 (62542-84246) | 892.54 (771.28-1001.61) | -0.34 (-0.39--0.29) |
| Saint Lucia | 427 (383-478) | 1170.53 (1050.16-1301.59) | 807 (723-903) | 1118.33 (1006.03-1246.94) | -0.2 (-0.22--0.18) |
| Saint Vincent and the Grenadines | 1151 (1001-1310) | 1183.14 (1047.77-1321.76) | 2593 (2322-2889) | 1115.29 (996.48-1244.66) | -0.18 (-0.21--0.16) |
| Samoa | 858 (744-985) | 1100.93 (969.67-1258.25) | 1525 (1351-1719) | 1104.34 (975.51-1248.06) | -0.02 (-0.04--0.01) |
| San Marino | 1536 (1294-1771) | 1496.21 (1306.52-1691.18) | 2293 (1996-2606) | 1436.94 (1264.6-1619.43) | -0.15 (-0.17--0.13) |
| Sao Tome and Principe | 944 (828-1077) | 1319.46 (1173.74-1490.63) | 1824 (1585-2083) | 1284.27 (1145.56-1449.8) | -0.1 (-0.11--0.08) |
| Saudi Arabia | 143281 (119113-168334) | 1613.96 (1401.65-1829.97) | 481885 (410390-563775) | 1518.12 (1323.67-1710.96) | -0.28 (-0.31--0.25) |
| Senegal | 50246 (42952-59386) | 1222.32 (1079.59-1389.67) | 105730 (91722-122701) | 1090.9 (964.19-1231.47) | -0.41 (-0.43--0.39) |
| Serbia | 100487 (89186-113374) | 906.21 (802.4-1023.52) | 120345 (108395-134903) | 831.09 (740.74-934.04) | -0.34 (-0.36--0.31) |
| Seychelles | 1157 (989-1336) | 1909.93 (1650.99-2176.66) | 2266 (1970-2583) | 1869.16 (1632.37-2123.41) | -0.1 (-0.12--0.09) |
| Sierra Leone | 31854 (27387-36552) | 1270.64 (1126.72-1414.45) | 60488 (52201-69068) | 1216.22 (1070.79-1353.33) | -0.15 (-0.17--0.14) |
| Singapore | 49144 (41736-56676) | 1780.26 (1550.89-2015.92) | 129829 (114283-145764) | 1558.79 (1358.48-1762.23) | -0.46 (-0.52--0.4) |
| Slovakia | 53699 (47572-60249) | 916.11 (811.57-1032.47) | 71632 (64218-80028) | 818.53 (727.4-919.55) | -0.39 (-0.4--0.38) |
| Slovenia | 21875 (19554-24822) | 916.68 (817.31-1047.91) | 31800 (28471-35398) | 842.39 (747.47-953.26) | -0.36 (-0.39--0.34) |
| Solomon Islands | 767384 (655595-894590) | 1629.42 (1403.23-1861.07) | 1670779 (1451353-1914266) | 1555.76 (1353.38-1773.43) | -0.06 (-0.12--0.01) |
| Somalia | 2502 (2101-2931) | 1399.73 (1220.28-1587.98) | 5875 (5020-6742) | 1332.36 (1163.76-1501.06) | -0.18 (-0.2--0.16) |
| South Africa | 34318 (28665-40987) | 918.77 (803.86-1054.37) | 84441 (70826-99772) | 891.45 (780.48-1024.26) | -0.12 (-0.14--0.11) |
| South Sudan | 355297 (323801-391232) | 1441.05 (1321.31-1558.13) | 703915 (644159-765503) | 1373.59 (1261.89-1486.64) | -0.21 (-0.23--0.2) |
| Spain | 31098 (26513-36249) | 956.02 (844.27-1075.83) | 48233 (41547-55858) | 936.06 (823.66-1062.07) | -0.09 (-0.1--0.07) |
| Sri Lanka | 424742 (381227-466607) | 830.74 (741.15-917.27) | 602895 (543453-669488) | 715.11 (638.78-797.82) | -0.53 (-0.61--0.45) |
| Sudan | 251062 (215390-289866) | 1874.91 (1644.44-2128.64) | 469003 (411399-527948) | 1793 (1580.56-2019.76) | -0.13 (-0.15--0.11) |
| Suriname | 184684 (159147-215022) | 1579.51 (1380.56-1781.12) | 409483 (350940-475295) | 1555.6 (1368.49-1763.13) | -0.09 (-0.1--0.07) |
| Sweden | 3356 (2920-3868) | 1149.47 (1015.42-1309.2) | 6940 (6136-7863) | 1096.53 (969.48-1246.18) | -0.17 (-0.19--0.16) |
| Switzerland | 132517 (117815-148281) | 1042.03 (922.57-1184.57) | 169334 (150919-187782) | 957.28 (850.13-1075.25) | -0.26 (-0.28--0.24) |
| Syrian Arab Republic | 99957 (89433-110689) | 1043.2 (925.25-1165.42) | 145267 (130672-160703) | 940.03 (839.51-1049.48) | -0.38 (-0.43--0.32) |
| Taiwan (Province of China) | 105201 (90055-121967) | 1557.06 (1361.36-1752.72) | 202272 (176303-229787) | 1456.12 (1273.77-1648.11) | -0.27 (-0.29--0.26) |
| Tajikistan | 215333 (189072-247960) | 1208.4 (1076.04-1365.28) | 407911 (368363-454214) | 1068.88 (961.81-1203.68) | -0.34 (-0.37--0.31) |
| Thailand | 47413 (40748-54696) | 1491.64 (1312.99-1692.61) | 99085 (86548-113296) | 1415.22 (1260.85-1597.48) | -0.2 (-0.22--0.19) |
| Timor-Leste | 804657 (693860-922896) | 1821 (1603.78-2052.11) | 1811543 (1611823-2013537) | 1819.72 (1613.55-2034.2) | -0.07 (-0.1--0.05) |
| Togo | 19468 (17305-21655) | 1019.87 (909.09-1129.55) | 31578 (28315-34972) | 978.01 (880.53-1082.45) | -0.17 (-0.2--0.15) |
| Tokelau | 7677 (6461-9079) | 1760.88 (1537.67-1985.37) | 17119 (14877-19651) | 1777.68 (1554.62-2016.01) | 0.03 (0.02-0.04) |
| Tonga | 20881 (17559-24216) | 1207.94 (1050.21-1347.92) | 56519 (48238-64779) | 1134.5 (990.11-1274.72) | -0.29 (-0.32--0.26) |
| Trinidad and Tobago | 19 (17-22) | 1473.58 (1291.24-1667.51) | 20 (18-23) | 1426.4 (1263.76-1607.55) | -0.12 (-0.12--0.11) |
| Tunisia | 969 (832-1110) | 1528.68 (1336.22-1730.93) | 1237 (1077-1390) | 1465.23 (1277.04-1644.07) | -0.13 (-0.15--0.11) |
| Türkiye | 10934 (9515-12449) | 1145.1 (1007.18-1285.1) | 20826 (18364-23310) | 1135.22 (1001.84-1275.31) | -0.06 (-0.07--0.05) |
| Turkmenistan | 90983 (77906-105656) | 1531.62 (1332.68-1753.26) | 194311 (169201-224186) | 1441.34 (1263.44-1660.06) | -0.25 (-0.26--0.23) |
| Tuvalu | 702989 (604696-812195) | 1696.37 (1483.81-1933.07) | 1494520 (1319956-1700293) | 1571.77 (1385.96-1789.36) | -0.23 (-0.26--0.19) |
| Uganda | 35916 (31188-41440) | 1593.17 (1419.7-1785.38) | 66556 (59250-74668) | 1510.54 (1356.16-1680.22) | -0.18 (-0.19--0.17) |
| Ukraine | 109 (93-126) | 1458.81 (1261.18-1670.86) | 158 (137-180) | 1428.88 (1254.22-1625.06) | -0.09 (-0.1--0.08) |
| United Arab Emirates | 86425 (72776-101071) | 1017.02 (886.98-1151.6) | 200739 (168812-233804) | 972.79 (846.19-1111.77) | -0.21 (-0.24--0.19) |
| United Kingdom | 1049913 (921148-1187629) | 1612.42 (1401.04-1853.12) | 1007294 (896599-1127662) | 1477.94 (1311.33-1689.43) | -0.35 (-0.37--0.33) |
| United Republic of Tanzania | 19935 (16452-24019) | 1703.59 (1508.57-1908.09) | 153684 (131038-177705) | 1578.93 (1397.94-1758.6) | -0.3 (-0.32--0.27) |
| United States of America | 751631 (693504-806116) | 902.22 (835.75-970.27) | 906634 (839184-970610) | 795.21 (739.1-850.73) | -0.62 (-0.75--0.48) |
| United States Virgin Islands | 119259 (100659-141850) | 870.95 (760.93-1002.92) | 314753 (266576-368606) | 942.3 (820.6-1073.95) | 0.3 (0.27-0.34) |
| Uruguay | 3190573 (2957899-3415721) | 1035.31 (959.55-1111.33) | 5677976 (5252216-6091645) | 1085.39 (1005.76-1165.45) | 0.36 (0.29-0.43) |
| Uzbekistan | 1068 (928-1217) | 1128.68 (981.62-1276.53) | 1659 (1471-1875) | 1099.9 (966.86-1242.28) | -0.11 (-0.11--0.1) |
| Vanuatu | 33217 (29417-36888) | 906.48 (798.39-1011.13) | 45015 (40208-50063) | 929.38 (826.09-1046.66) | 0.11 (0.09-0.14) |
| Venezuela (Bolivarian Republic of) | 197833 (171126-226520) | 1508.14 (1337.21-1683.02) | 443793 (389039-499988) | 1509.93 (1345.74-1683.75) | 0.02 (0-0.04) |
| Viet Nam | 1327 (1130-1555) | 1546.26 (1359.86-1755.26) | 3166 (2746-3656) | 1479.04 (1307.56-1670.54) | -0.16 (-0.18--0.15) |
| Yemen | 94920 (80868-110530) | 1448.11 (1263.11-1633.92) | 270536 (232375-312505) | 1406.85 (1232.25-1580.68) | -0.17 (-0.19--0.14) |
| Zambia | 37633 (31239-44289) | 954.81 (824.96-1082.41) | 99250 (82808-117537) | 958.8 (829.18-1099.68) | 0.02 (-0.01-0.05) |
| Zimbabwe | 69969 (61017-79589) | 1363.72 (1213.98-1523.23) | 115919 (101556-131129) | 1331.53 (1184.2-1482.33) | -0.07 (-0.08--0.06) |

| **TableS21: National Burden of chronic kidney disease due to diabetes mellitus type 2: deaths, ASDR, and EAPC (1990–2021).** | | | | | |
| --- | --- | --- | --- | --- | --- |
| Country | **1990** | | **2021** | | **EAPC_95%CI** |
|  | Number_95%UI | ASR | Number_95%UI | ASR |  |
| Afghanistan | 595 (397-945) | 9.37 (6.27-15.06) | 851 (446-1547) | 10.87 (5.78-19.82) | 0.53 (0.49-0.58) |
| Albania | 21 (15-30) | 1.23 (0.86-1.76) | 49 (33-74) | 1.15 (0.79-1.68) | 0.3 (0.05-0.56) |
| Algeria | 492 (303-872) | 5.84 (3.65-10.84) | 2182 (1585-2941) | 8.12 (5.99-10.9) | 1.52 (1.32-1.72) |
| American Samoa | 4 (3-6) | 23.7 (17.05-34.35) | 21 (16-26) | 51.89 (39.96-64.7) | 2.75 (2.51-2.98) |
| Andorra | 1 (1-1) | 2.07 (1.38-3.12) | 3 (2-4) | 1.64 (1.07-2.45) | -0.41 (-0.64--0.19) |
| Angola | 188 (127-272) | 6.53 (4.57-9.11) | 597 (396-859) | 7.15 (4.81-10.35) | 0.16 (0.04-0.28) |
| Antigua and Barbuda | 8 (7-9) | 13.43 (11.57-15.82) | 21 (19-25) | 22.18 (19.09-25.85) | 2.19 (1.93-2.45) |
| Argentina | 1893 (1467-2366) | 6.17 (4.84-7.64) | 3321 (2595-4174) | 5.69 (4.45-7.11) | -0.07 (-0.39-0.25) |
| Armenia | 3 (2-4) | 0.12 (0.09-0.16) | 69 (49-93) | 1.58 (1.13-2.13) | 7.8 (6.55-9.07) |
| Australia | 80 (63-103) | 0.45 (0.34-0.58) | 390 (278-541) | 0.73 (0.53-1) | 2.67 (2.16-3.18) |
| Austria | 146 (110-189) | 1.16 (0.9-1.48) | 662 (509-817) | 2.78 (2.2-3.41) | 4.18 (3.48-4.88) |
| Azerbaijan | 40 (28-57) | 0.84 (0.58-1.19) | 141 (96-199) | 1.53 (1.04-2.15) | 2.36 (2.05-2.68) |
| Bahamas | 10 (7-14) | 9.09 (6.09-13) | 55 (40-77) | 11.25 (8.02-15.92) | 0.27 (-0.13-0.67) |
| Bahrain | 1661 (1271-2396) | 3.96 (3.06-5.62) | 4499 (3293-6446) | 3.71 (2.74-5.15) | -0.17 (-0.45-0.12) |
| Bangladesh | 29 (25-33) | 9.32 (8.15-10.57) | 73 (56-93) | 13.85 (10.53-17.52) | 1.79 (1.48-2.1) |
| Barbados | 9 (7-12) | 0.08 (0.06-0.1) | 42 (30-58) | 0.25 (0.18-0.35) | 3.95 (3.16-4.74) |
| Belarus | 240 (178-317) | 1.48 (1.11-1.92) | 470 (331-650) | 1.51 (1.08-2.02) | 0.9 (0.51-1.3) |
| Belgium | 10 (8-11) | 10.72 (9.33-12.37) | 54 (45-64) | 20.27 (16.99-23.8) | 2.54 (2.04-3.04) |
| Belize | 91 (66-123) | 5.21 (3.88-6.99) | 234 (168-325) | 5.76 (4.15-8.13) | 0.26 (0.15-0.37) |
| Benin | 4 (4-5) | 7.18 (6.26-8.29) | 11 (9-14) | 7.4 (5.9-9.4) | 0.57 (0.25-0.88) |
| Bermuda | 11 (7-16) | 5.25 (3.63-7.87) | 37 (23-54) | 6.59 (4.24-9.55) | 0.81 (0.74-0.88) |
| Bhutan | 297 (231-371) | 3.47 (2.74-4.35) | 2615 (1867-3786) | 9.1 (6.48-13.07) | 2.9 (2.45-3.34) |
| Bolivia (Plurinational State of) | 54 (39-75) | 1.58 (1.15-2.16) | 130 (94-174) | 2 (1.43-2.67) | 0.84 (0.58-1.11) |
| Bosnia and Herzegovina | 13 (8-19) | 2.96 (2.01-4.36) | 43 (29-62) | 3.85 (2.65-5.42) | 0.97 (0.78-1.17) |
| Botswana | 4643 (3919-5437) | 6.21 (5.25-7.31) | 17370 (14533-20606) | 7.17 (5.96-8.5) | 0.58 (0.41-0.75) |
| Brazil | 13 (11-18) | 17.59 (14.01-22.78) | 38 (30-47) | 17.12 (13.61-21.12) | 0.54 (0.29-0.79) |
| Brunei Darussalam | 76 (55-101) | 0.75 (0.57-0.97) | 281 (203-379) | 1.86 (1.36-2.51) | 3.89 (3.38-4.4) |
| Bulgaria | 167 (118-231) | 4.85 (3.57-6.64) | 395 (283-550) | 5.39 (3.9-7.42) | 0.32 (0.26-0.38) |
| Burkina Faso | 211 (153-289) | 10.76 (7.87-14.78) | 355 (235-523) | 10.03 (6.73-14.96) | -0.61 (-0.75--0.47) |
| Burundi | 267 (200-349) | 6.73 (5.07-8.75) | 801 (561-1100) | 7.58 (5.37-10.23) | 0.32 (0.16-0.48) |
| C?te d'Ivoire | 336 (225-474) | 9.67 (6.6-13.36) | 1009 (653-1496) | 10.57 (6.93-15.31) | 0.43 (0.21-0.65) |
| Cabo Verde | 562 (441-697) | 1.74 (1.37-2.16) | 1953 (1499-2563) | 2.39 (1.85-3.08) | 1.42 (1.21-1.63) |
| Cambodia | 78 (54-110) | 8.95 (6.39-12.27) | 144 (94-209) | 9.13 (6.31-13.18) | 0.08 (0.03-0.12) |
| Cameroon | 101 (70-146) | 4.09 (2.87-5.75) | 226 (153-342) | 4.98 (3.44-7.47) | 0.48 (0.31-0.65) |
| Canada | 273 (213-348) | 3.02 (2.4-3.82) | 1058 (807-1349) | 4 (3.06-5.08) | 1.36 (0.92-1.79) |
| Central African Republic | 43537 (35988-53065) | 6.83 (5.74-8.34) | 107652 (84626-134047) | 5.64 (4.46-7) | -0.57 (-0.66--0.49) |
| Chad | 619 (491-767) | 4.15 (3.34-5.13) | 1971 (1457-2617) | 3.53 (2.61-4.7) | -0.28 (-0.43--0.14) |
| Chile | 14 (12-16) | 9.74 (8.34-11.27) | 60 (48-76) | 16.38 (13.29-20.37) | 2.28 (2.02-2.54) |
| China | 15 (11-21) | 9.79 (7.13-13.21) | 42 (29-61) | 10.68 (7.19-15.04) | 0.12 (0.03-0.2) |
| Colombia | 89 (61-122) | 10.88 (7.25-14.7) | 214 (134-300) | 11.1 (6.91-15.79) | -0.12 (-0.23--0.01) |
| Comoros | 1 (1-2) | 11.84 (9.28-15.93) | 4 (3-5) | 15.05 (11.37-18.4) | 0.82 (0.72-0.92) |
| Congo | 63 (50-78) | 3.87 (3.09-4.82) | 422 (330-536) | 7.61 (5.93-9.7) | 2.39 (2.02-2.76) |
| Cook Islands | 59 (43-78) | 1.1 (0.81-1.43) | 174 (122-242) | 1.71 (1.22-2.34) | 1.23 (0.88-1.59) |
| Costa Rica | 357 (314-406) | 3.61 (3.18-4.1) | 1583 (1327-1896) | 7.69 (6.47-9.26) | 3.09 (2.77-3.41) |
| Croatia | 34 (23-48) | 7.04 (4.96-10.2) | 61 (43-85) | 3.65 (2.6-5.1) | -2.32 (-2.59--2.05) |
| Cuba | 127 (92-169) | 0.91 (0.67-1.18) | 202 (145-275) | 0.84 (0.61-1.15) | -0.19 (-0.39-0.01) |
| Cyprus | 930 (661-1255) | 7.18 (5.06-10.05) | 2183 (1674-2857) | 7.14 (5.49-9.56) | 0.13 (0-0.27) |
| Czechia | 998 (711-1383) | 8.84 (6.62-11.93) | 2445 (1604-3595) | 9.15 (6-13.68) | -0.02 (-0.13-0.09) |
| Democratic People's Republic of Korea | 69 (53-90) | 0.77 (0.59-0.99) | 303 (242-377) | 2.13 (1.71-2.63) | 3.44 (3.12-3.77) |
| Democratic Republic of the Congo | 7 (5-11) | 8.23 (5.81-11.4) | 53 (36-76) | 12.33 (8.76-17.26) | 1.31 (1.2-1.42) |
| Denmark | 9 (7-11) | 14.96 (12.5-17.83) | 19 (14-23) | 23.33 (18.18-28.99) | 1.63 (1.54-1.71) |
| Djibouti | 192 (152-252) | 6.18 (4.91-8.1) | 911 (591-1233) | 9.43 (6.11-12.72) | 2.14 (1.86-2.41) |
| Dominica | 395 (328-466) | 8.59 (7.18-10.25) | 2525 (1848-3558) | 16.46 (12.32-22.96) | 2.28 (1.36-3.21) |
| Dominican Republic | 1812 (1226-2850) | 9.34 (6.23-14.77) | 6533 (4671-9047) | 14.02 (10.14-19.48) | 1.64 (1.53-1.75) |
| Ecuador | 148 (112-226) | 5.22 (3.92-7.98) | 986 (658-1344) | 15.47 (10.33-21.27) | 3.58 (3.08-4.08) |
| Egypt | 13 (9-18) | 8.34 (6.05-11.32) | 42 (23-64) | 11.53 (6.12-17.33) | 1.41 (1.03-1.8) |
| El Salvador | 69 (47-102) | 8.76 (5.98-12.75) | 205 (132-338) | 10.86 (7.06-17.68) | 0.69 (0.59-0.79) |
| Equatorial Guinea | 10 (7-13) | 0.49 (0.37-0.65) | 85 (65-110) | 2.47 (1.89-3.14) | 5.06 (4.62-5.5) |
| Eritrea | 4142 (3245-5142) | 27.39 (21.92-33.02) | 5989 (4758-7316) | 17.53 (13.91-21.44) | -1.82 (-1.95--1.68) |
| Estonia | 9 (6-14) | 21.58 (14.52-35.88) | 22 (15-31) | 37.66 (27.24-54.15) | 1.76 (1.35-2.19) |
| Eswatini | 50 (34-74) | 17.9 (12.54-27.18) | 191 (141-252) | 32.07 (24.03-41.32) | 1.41 (1.05-1.78) |
| Ethiopia | 56 (43-71) | 0.77 (0.59-0.97) | 214 (164-265) | 1.25 (0.98-1.53) | 2.14 (1.94-2.34) |
| Fiji | 1138 (843-1512) | 1.25 (0.94-1.65) | 2439 (1845-3081) | 1.18 (0.92-1.45) | 0.3 (-0.06-0.66) |
| Finland | 50 (36-69) | 10.18 (7.45-13.91) | 121 (61-180) | 15.39 (7.37-22.77) | 1.3 (1.13-1.47) |
| France | 25 (17-36) | 0.4 (0.27-0.57) | 115 (82-162) | 1.86 (1.33-2.61) | 5.46 (4.69-6.23) |
| Gabon | 1882 (1392-2510) | 1.37 (1.03-1.79) | 6662 (4623-9475) | 2.57 (1.81-3.58) | 2.95 (2.46-3.45) |
| Gambia | 255 (176-375) | 5.45 (3.86-8.08) | 1199 (871-1678) | 9.58 (6.95-13.48) | 2.27 (2.09-2.45) |
| Georgia | 628 (467-825) | 4.34 (3.27-5.65) | 1437 (1138-1726) | 4.36 (3.56-5.13) | -0.75 (-1.69-0.2) |
| Germany | 1 (1-1) | 3.39 (2.6-4.35) | 2 (1-3) | 3.71 (2.62-5.03) | 0.76 (0.56-0.97) |
| Ghana | 11 (10-13) | 14.9 (12.56-17.67) | 28 (23-34) | 26.7 (21.99-31.76) | 2.18 (1.9-2.46) |
| Greece | 6 (5-7) | 10.66 (9.02-13.25) | 24 (20-28) | 11.15 (9.12-13.08) | 1.13 (0.62-1.64) |
| Greenland | 192 (148-241) | 7.37 (5.83-9.23) | 1133 (841-1528) | 11.18 (8.36-14.91) | 2.16 (1.72-2.59) |
| Grenada | 94 (65-134) | 3.31 (2.3-4.69) | 171 (115-255) | 3.7 (2.51-5.5) | 0.24 (0.17-0.32) |
| Guam | 23 (16-32) | 7.16 (5.13-9.78) | 36 (26-51) | 6.84 (4.93-9.54) | -0.2 (-0.25--0.16) |
| Guatemala | 44 (37-52) | 13.14 (11.21-15.47) | 143 (111-184) | 24.59 (19.07-31.12) | 3.05 (2.69-3.42) |
| Guinea | 317 (218-550) | 11.63 (7.92-20.66) | 777 (415-1713) | 13.08 (7.15-28.35) | 0.61 (0.53-0.69) |
| Guinea-Bissau | 47 (34-65) | 2.61 (1.93-3.65) | 324 (222-448) | 5.85 (4.09-7.99) | 2.9 (2.62-3.18) |
| Guyana | 83 (63-109) | 0.58 (0.44-0.76) | 247 (180-338) | 1.11 (0.82-1.51) | 3.3 (2.91-3.68) |
| Haiti | 1 (1-2) | 0.38 (0.28-0.52) | 4 (3-6) | 0.63 (0.48-0.82) | 2.21 (1.94-2.47) |
| Honduras | 15340 (12049-19023) | 3.88 (3.09-4.77) | 56202 (42578-70450) | 5.16 (3.97-6.52) | 0.86 (0.71-1.02) |
| Hungary | 4692 (3753-6231) | 5.51 (4.41-7.48) | 15860 (12207-20634) | 7.85 (6.09-10.63) | 1.2 (1.09-1.3) |
| Iceland | 655 (471-907) | 8.94 (6.43-12.52) | 2079 (1298-2912) | 11.11 (7.08-15.46) | 0.43 (0.23-0.63) |
| India | 55 (42-73) | 1.41 (1.08-1.85) | 126 (88-173) | 1.45 (1.02-1.97) | 0.79 (0.58-1.01) |
| Indonesia | 869 (612-1219) | 4.5 (3.15-6.4) | 3433 (2550-4342) | 5.15 (3.77-6.57) | 0.53 (0.39-0.67) |
| Iran (Islamic Republic of) | 211 (159-278) | 4.67 (3.53-6.03) | 604 (446-802) | 4.25 (3.15-5.63) | 0.44 (-0.19-1.08) |
| Iraq | 1203 (906-1594) | 1.34 (1.02-1.74) | 2874 (1974-4075) | 1.39 (0.99-1.92) | 0.18 (0.05-0.31) |
| Ireland | 189 (159-220) | 10.15 (8.57-11.79) | 380 (278-504) | 11.87 (8.67-15.85) | -0.09 (-0.71-0.53) |
| Israel | 6431 (5189-7844) | 4.15 (3.33-5.08) | 18934 (13475-24887) | 3.38 (2.54-4.26) | -0.67 (-0.81--0.54) |
| Italy | 108 (81-142) | 10.66 (7.94-14.27) | 635 (471-844) | 11.59 (8.59-15.52) | 0.08 (-0.27-0.43) |
| Jamaica | 85 (62-112) | 0.67 (0.49-0.89) | 311 (225-430) | 1.95 (1.43-2.7) | 3.21 (2.86-3.56) |
| Japan | 460 (329-724) | 7.06 (5.11-11.21) | 1856 (1331-2497) | 10.99 (7.95-14.7) | 1.64 (1.57-1.72) |
| Jordan | 13 (9-19) | 5.94 (4.27-8.29) | 37 (22-56) | 8.26 (5.03-12.01) | 1.32 (0.66-1.97) |
| Kazakhstan | 6 (5-7) | 19.07 (15.21-23.99) | 16 (10-23) | 28.02 (18.31-40.41) | 1 (0.68-1.31) |
| Kenya | 32 (25-40) | 7.1 (5.44-9.08) | 83 (57-113) | 3.78 (2.52-5.32) | -1.98 (-2.24--1.71) |
| Kiribati | 13 (9-17) | 0.44 (0.32-0.58) | 60 (43-81) | 1.34 (0.99-1.79) | 2.13 (1.22-3.04) |
| Kuwait | 286 (210-390) | 15.87 (11.77-21.33) | 600 (407-840) | 15.45 (10.54-21.21) | -0.21 (-0.25--0.16) |
| Kyrgyzstan | 8 (6-10) | 0.23 (0.17-0.3) | 49 (35-67) | 1.02 (0.74-1.39) | 5.61 (5.13-6.09) |
| Lao People's Democratic Republic | 123 (81-171) | 6.74 (4.59-9.59) | 432 (319-582) | 6.61 (4.95-8.84) | 0.1 (-0.01-0.22) |
| Latvia | 16 (11-23) | 2.12 (1.43-3.01) | 44 (27-67) | 4.8 (3.03-7.18) | 3.51 (3.06-3.97) |
| Lebanon | 64 (45-89) | 6.66 (4.84-9.14) | 116 (79-167) | 7.37 (5.01-10.37) | 0.55 (0.3-0.81) |
| Lesotho | 87 (58-129) | 5.21 (3.49-7.61) | 395 (234-562) | 9 (5.41-12.71) | 2.52 (2.28-2.76) |
| Liberia | 8 (6-10) | 0.17 (0.13-0.23) | 45 (33-62) | 0.66 (0.49-0.9) | 3.21 (2.66-3.77) |
| Libya | 9 (7-12) | 1.72 (1.31-2.24) | 27 (19-38) | 2.16 (1.53-2.99) | 1.14 (0.91-1.37) |
| Lithuania | 297 (209-447) | 7.31 (5.17-11.02) | 592 (396-843) | 7.68 (5.25-10.62) | 0.19 (0.11-0.28) |
| Luxembourg | 294 (211-397) | 10.01 (7.45-13.23) | 688 (505-916) | 12.18 (9.11-16.11) | 0.46 (0.27-0.66) |
| Madagascar | 757 (628-908) | 9.1 (7.51-10.85) | 3041 (2446-3665) | 12.06 (9.75-14.44) | 0.67 (0.44-0.91) |
| Malawi | 13 (10-17) | 18.1 (13.94-26.65) | 31 (24-39) | 11.44 (8.79-14.54) | -1.78 (-1.97--1.59) |
| Malaysia | 167 (116-234) | 5.49 (3.99-7.39) | 377 (269-551) | 5.63 (4.11-8.21) | 0.21 (0.09-0.34) |
| Maldives | 8 (6-10) | 2.08 (1.59-2.73) | 25 (18-34) | 2.16 (1.56-2.89) | 0.18 (-0.06-0.42) |
| Mali | 3 (2-5) | 20.84 (12.84-38.62) | 9 (3-27) | 34.84 (10.9-104.71) | 1.67 (1.45-1.88) |
| Malta | 54 (36-77) | 6.36 (4.43-9) | 112 (68-170) | 6.28 (3.89-9.33) | -0.4 (-0.58--0.23) |
| Marshall Islands | 112 (96-129) | 17.65 (15.32-20.29) | 585 (507-662) | 32.5 (28.46-36.73) | 2.18 (1.78-2.58) |
| Mauritania | 3174 (2565-3831) | 9.41 (7.63-11.46) | 16033 (12633-19982) | 13.35 (10.59-16.53) | 1.78 (1.15-2.41) |
| Mauritius | 20 (14-28) | 2.04 (1.43-2.85) | 35 (24-47) | 1.8 (1.26-2.43) | -0.83 (-0.99--0.67) |
| Mexico | 8 (5-11) | 1.32 (0.95-1.85) | 19 (13-28) | 2.12 (1.38-3.08) | 1.88 (1.68-2.07) |
| Micronesia (Federated States of) | 459 (274-858) | 3.62 (2.18-7.01) | 1995 (1403-2777) | 6.6 (4.67-9.13) | 2.41 (2.22-2.6) |
| Monaco | 301 (216-459) | 7.1 (5.2-10.5) | 770 (546-1202) | 10.01 (6.91-14.81) | 1.53 (1.39-1.67) |
| Mongolia | 1775 (1290-2431) | 8.76 (6.51-11.89) | 3794 (2911-5014) | 8.92 (6.87-11.97) | -0.17 (-0.24--0.09) |
| Montenegro | 15 (10-22) | 2.86 (1.93-4.14) | 40 (27-58) | 3.59 (2.47-5.22) | 0.56 (0.26-0.86) |
| Morocco | 324 (230-456) | 4.06 (2.92-5.67) | 1283 (885-1818) | 6.21 (4.35-8.81) | 1.61 (1.24-1.98) |
| Mozambique | 188 (138-256) | 0.91 (0.67-1.23) | 641 (487-809) | 1.55 (1.19-1.95) | 1.68 (1.16-2.2) |
| Myanmar | 30 (23-39) | 0.8 (0.61-1.03) | 116 (88-149) | 1.28 (0.98-1.65) | 1.82 (1.22-2.42) |
| Namibia | 84 (64-120) | 6.22 (4.74-8.82) | 552 (409-758) | 12.17 (9.12-16.81) | 2.75 (2.32-3.17) |
| Nauru | 64 (43-94) | 3.09 (2.07-4.36) | 176 (117-292) | 2.88 (1.94-4.53) | -0.28 (-0.37--0.19) |
| Nepal | 1566 (1106-2108) | 4.45 (3.19-6.04) | 2823 (1948-3854) | 4.24 (2.95-5.77) | -0.82 (-1.14--0.5) |
| Netherlands | 3 (2-4) | 27 (21.91-34.24) | 17 (14-21) | 42.52 (34.32-51) | 1.6 (1.42-1.78) |
| New Zealand | 38 (29-51) | 0.49 (0.37-0.64) | 109 (77-149) | 0.87 (0.63-1.16) | 1.98 (1.56-2.41) |
| Nicaragua | 29 (20-43) | 5.27 (3.55-7.73) | 138 (99-192) | 9.85 (7.1-13.3) | 2.67 (2.37-2.98) |
| Niger | 2836 (2073-3862) | 5.66 (4.13-7.69) | 8300 (6160-11022) | 8.27 (6.19-10.97) | 1.04 (0.81-1.27) |
| Nigeria | 67 (46-97) | 9.12 (6.2-13.22) | 171 (129-231) | 9.01 (6.67-11.94) | -0.06 (-0.33-0.21) |
| Niue | 41 (32-52) | 2.98 (2.35-3.71) | 322 (234-435) | 7.16 (5.2-9.72) | 3.16 (2.69-3.63) |
| North Macedonia | 96 (63-140) | 6.66 (4.63-9.66) | 327 (240-463) | 8.19 (6.09-12.15) | 0.66 (0.62-0.7) |
| Northern Mariana Islands | 117 (92-149) | 5.82 (4.58-7.39) | 513 (361-706) | 9.51 (6.69-13.07) | 1.81 (1.69-1.93) |
| Norway | 1039 (824-1298) | 9.69 (7.7-12.14) | 3970 (2659-5529) | 12.05 (8.04-16.83) | 0.48 (0.27-0.69) |
| Oman | 2482 (2039-3026) | 11.36 (9.51-13.47) | 10925 (8758-13662) | 15.18 (12.29-18.71) | 1.41 (1.27-1.55) |
| Pakistan | 443 (342-591) | 16.52 (12.89-21.56) | 1883 (1352-2602) | 23.99 (17.54-32.62) | 1.35 (1.24-1.46) |
| Palau | 669 (499-871) | 1.58 (1.19-2.03) | 725 (548-966) | 0.93 (0.71-1.23) | -2.29 (-2.9--1.68) |
| Palestine | 305 (232-396) | 2.38 (1.83-3.08) | 940 (661-1288) | 2.83 (2.03-3.8) | 0.6 (0.1-1.11) |
| Panama | 1 (1-1) | 1.07 (0.75-1.52) | 2 (2-3) | 1.73 (1.21-2.42) | 1.78 (1.5-2.06) |
| Papua New Guinea | 520 (451-594) | 15.06 (13.15-17.14) | 1383 (1144-1635) | 16.24 (13.49-19.25) | 0.99 (0.52-1.46) |
| Paraguay | 5 (3-8) | 9.08 (5.04-15.12) | 40 (29-58) | 9 (6.61-12.45) | -0.46 (-1.09-0.16) |
| Peru | 6 (4-8) | 2.49 (1.86-3.34) | 21 (13-30) | 5.24 (3.18-7.33) | 2.12 (1.83-2.41) |
| Philippines | 132 (95-185) | 4.65 (3.39-6.31) | 412 (279-583) | 4.98 (3.51-6.94) | 0.03 (-0.07-0.13) |
| Poland | 1263 (1069-1461) | 6.03 (5.07-7.13) | 4209 (3263-5250) | 4.58 (3.53-5.73) | -0.57 (-0.74--0.4) |
| Portugal | 6 (4-8) | 0.17 (0.13-0.22) | 21 (15-30) | 0.34 (0.25-0.48) | 1.86 (1.36-2.36) |
| Puerto Rico | 1 (1-2) | 26.32 (16.68-45.68) | 2 (1-3) | 40.64 (27.35-72.11) | 1.26 (1.15-1.38) |
| Qatar | 0 (0-1) | 19.81 (15.1-26.56) | 1 (0-1) | 36.32 (21.28-55.84) | 1.94 (1.75-2.12) |
| Republic of Korea | 2 (1-2) | 19.43 (14.08-25.62) | 6 (4-8) | 33.16 (23.66-44.3) | 1.93 (1.72-2.13) |
| Republic of Moldova | 0 (0-1) | 0.95 (0.67-1.32) | 1 (0-1) | 0.69 (0.41-1.05) | 0.26 (-0.17-0.68) |
| Romania | 13 (10-19) | 4.94 (3.58-6.88) | 52 (35-74) | 6.53 (4.42-9.59) | 0.77 (0.67-0.86) |
| Russian Federation | 89 (64-131) | 0.36 (0.27-0.52) | 272 (214-344) | 0.67 (0.52-0.84) | 3.07 (2.39-3.75) |
| Rwanda | 806 (597-1061) | 0.47 (0.36-0.61) | 2404 (1848-3135) | 0.98 (0.76-1.28) | 2.4 (1.84-2.95) |
| Saint Kitts and Nevis | 239 (173-326) | 10.87 (8.07-14.55) | 414 (286-608) | 8.94 (6.29-13.1) | -1.43 (-1.76--1.1) |
| Saint Lucia | 7 (6-8) | 19.02 (16.4-22.02) | 16 (12-19) | 27.4 (22.33-32.83) | 2.17 (1.87-2.48) |
| Saint Vincent and the Grenadines | 13 (11-15) | 16.4 (14.21-18.85) | 42 (34-51) | 17.91 (14.46-21.92) | 0.51 (0.21-0.8) |
| Samoa | 8 (7-9) | 11.61 (9.93-13.52) | 24 (20-29) | 17.94 (15.05-21.24) | 2.06 (1.73-2.39) |
| San Marino | 14 (10-20) | 20.42 (14.74-27.58) | 35 (27-46) | 28.43 (21.55-36.36) | 0.96 (0.83-1.1) |
| Sao Tome and Principe | 5 (4-6) | 8.6 (6.69-11.38) | 11 (7-14) | 12.35 (7.96-17) | 1.28 (1.21-1.35) |
| Saudi Arabia | 538 (367-775) | 11.14 (7.69-16.02) | 2815 (1952-3814) | 20.07 (14.02-26.79) | 1.67 (1.48-1.87) |
| Senegal | 181 (130-240) | 6.82 (4.97-8.93) | 479 (336-697) | 7.65 (5.39-11.04) | 0.25 (0.17-0.33) |
| Serbia | 201 (144-276) | 2.27 (1.63-3.11) | 421 (314-535) | 2.37 (1.77-3.01) | 0.37 (0.19-0.54) |
| Seychelles | 6 (5-8) | 11.35 (9.3-13.99) | 20 (15-25) | 19.19 (15.02-23.89) | 1.81 (1.52-2.1) |
| Sierra Leone | 76 (54-107) | 4.29 (3.12-6.04) | 130 (87-188) | 4.29 (2.95-6.16) | -0.01 (-0.07-0.04) |
| Singapore | 118 (102-136) | 6.61 (5.66-7.55) | 416 (344-491) | 5.09 (4.19-6.01) | 0.94 (0.37-1.51) |
| Slovakia | 81 (58-108) | 1.36 (0.98-1.84) | 120 (86-168) | 1.23 (0.87-1.7) | -0.15 (-0.34-0.05) |
| Slovenia | 15 (12-18) | 0.61 (0.49-0.75) | 37 (25-56) | 0.68 (0.45-1.02) | 1.17 (0.79-1.54) |
| Solomon Islands | 3018 (2156-4080) | 8.27 (5.86-11.12) | 8537 (6083-11205) | 9.99 (7.03-13.03) | 0.8 (0.7-0.9) |
| Somalia | 15 (7-23) | 13.36 (7.48-20.49) | 42 (33-54) | 14.93 (12.01-18.87) | 0.32 (0.12-0.53) |
| South Africa | 208 (139-312) | 12.51 (8.62-18.59) | 583 (371-886) | 13.51 (9.06-20.11) | 0.4 (0.31-0.5) |
| South Sudan | 493 (363-710) | 2.8 (2.07-4.04) | 1626 (1233-2176) | 4.19 (3.2-5.59) | 2.02 (1.69-2.35) |
| Spain | 251 (169-385) | 11.56 (8.09-17.31) | 452 (296-648) | 15.88 (10.91-22.55) | 0.96 (0.81-1.11) |
| Sri Lanka | 1241 (934-1643) | 2.31 (1.73-3.01) | 2584 (1797-3648) | 1.83 (1.29-2.52) | -0.35 (-0.59--0.1) |
| Sudan | 832 (638-1060) | 9.55 (7.26-12.09) | 2007 (1321-2852) | 7.99 (5.33-11.14) | -0.55 (-0.74--0.37) |
| Suriname | 229 (138-423) | 2.81 (1.7-5.33) | 700 (469-1037) | 4.31 (2.94-6.38) | 1.19 (0.94-1.43) |
| Sweden | 28 (24-35) | 12.1 (10.16-14.85) | 111 (79-147) | 18.37 (13.16-24.01) | 1.79 (1.57-2.01) |
| Switzerland | 119 (89-158) | 0.68 (0.51-0.89) | 475 (337-653) | 1.64 (1.17-2.22) | 3.45 (3.26-3.64) |
| Syrian Arab Republic | 119 (88-157) | 1.02 (0.77-1.34) | 395 (289-505) | 1.57 (1.19-1.98) | 2.37 (2.04-2.71) |
| Taiwan (Province of China) | 346 (247-479) | 7.89 (5.6-10.87) | 1012 (654-1479) | 9.63 (6.19-14.05) | 0.23 (-0.01-0.46) |
| Tajikistan | 1620 (1444-1808) | 13.96 (12.37-15.64) | 5228 (4422-6041) | 11.68 (9.95-13.47) | -0.18 (-0.51-0.16) |
| Thailand | 5 (4-8) | 0.2 (0.14-0.32) | 15 (10-22) | 0.29 (0.19-0.43) | 0.81 (0.35-1.28) |
| Timor-Leste | 2616 (2105-3407) | 9.02 (7.25-11.57) | 12608 (9428-16658) | 11.53 (8.62-15.21) | 0.5 (0.41-0.59) |
| Togo | 17 (12-24) | 1.03 (0.74-1.4) | 43 (29-64) | 1.52 (1.03-2.16) | 1.23 (0.61-1.86) |
| Tokelau | 19 (14-28) | 8.93 (6.67-12.55) | 80 (55-118) | 10.47 (7.24-15.19) | 0.72 (0.53-0.91) |
| Tonga | 45 (32-61) | 4.74 (3.48-6.49) | 153 (104-218) | 5.5 (3.87-7.91) | 0.34 (0.26-0.42) |
| Trinidad and Tobago | 0 (0-0) | 16.09 (11.15-28.14) | 0 (0-1) | 23.58 (16.73-35.96) | 1.3 (1.18-1.43) |
| Tunisia | 4 (3-6) | 8.84 (6.16-12.2) | 11 (7-14) | 14.02 (9.82-18.34) | 1.42 (1.17-1.68) |
| Türkiye | 80 (69-91) | 10.78 (9.37-12.25) | 342 (251-468) | 17.69 (13.07-24.18) | 2.35 (1.97-2.73) |
| Turkmenistan | 179 (120-279) | 4.39 (2.98-6.73) | 814 (537-1176) | 6.83 (4.55-9.87) | 1.47 (1.29-1.65) |
| Tuvalu | 1858 (1339-2690) | 6.43 (4.62-9.3) | 5925 (4408-7995) | 6.93 (5.14-9.4) | 0.91 (0.5-1.32) |
| Uganda | 20 (15-27) | 1.09 (0.81-1.42) | 90 (62-126) | 2.42 (1.72-3.34) | 2.1 (1.48-2.72) |
| Ukraine | 1 (1-2) | 17.64 (13-28.89) | 2 (2-4) | 26.56 (18.98-39.55) | 1.4 (1.28-1.52) |
| United Arab Emirates | 475 (334-667) | 9.27 (6.66-12.96) | 1302 (919-1845) | 11.79 (8.55-16.48) | 0.53 (0.39-0.66) |
| United Kingdom | 8 (6-10) | 0.01 (0.01-0.01) | 151 (104-209) | 0.19 (0.13-0.26) | 13.37 (11.39-15.39) |
| United Republic of Tanzania | 18 (11-27) | 5.88 (3.69-8.93) | 165 (105-241) | 10.14 (5.34-14.68) | 4.22 (3.43-5.01) |
| United States of America | 759 (571-985) | 0.78 (0.59-1.01) | 1704 (1250-2304) | 1.06 (0.79-1.41) | 1.96 (1.65-2.28) |
| United States Virgin Islands | 380 (263-548) | 4.45 (3.21-6.31) | 993 (722-1424) | 4.94 (3.58-7.08) | 0.18 (0.11-0.24) |
| Uruguay | 7648 (6090-9495) | 2.3 (1.84-2.81) | 55206 (47673-61989) | 8.8 (7.67-9.88) | 4.93 (4.67-5.19) |
| Uzbekistan | 6 (5-7) | 8.56 (7.07-10.46) | 16 (11-22) | 8.71 (6-11.8) | 0.38 (0.07-0.69) |
| Vanuatu | 101 (92-110) | 2.56 (2.32-2.77) | 224 (172-294) | 3.43 (2.67-4.41) | 1.62 (1.26-1.98) |
| Venezuela (Bolivarian Republic of) | 74 (43-131) | 0.7 (0.41-1.26) | 453 (337-605) | 1.99 (1.49-2.67) | 2.35 (1.5-3.2) |
| Viet Nam | 7 (4-12) | 14.33 (9.6-24.78) | 30 (22-46) | 22.18 (16.07-33.51) | 1.47 (1.4-1.54) |
| Yemen | 163 (100-314) | 3.98 (2.44-7.99) | 472 (297-769) | 4.14 (2.65-6.83) | 0.02 (-0.1-0.15) |
| Zambia | 278 (208-370) | 12.73 (9.59-16.7) | 771 (541-1109) | 14.93 (10.74-20.71) | 0.2 (0.04-0.35) |
| Zimbabwe | 108 (74-155) | 3.41 (2.44-4.85) | 279 (186-402) | 5.31 (3.56-7.75) | 1.78 (1.34-2.22) |

| **TableS22: National Burden of chronic kidney disease due to diabetes mellitus type 2: DALYs Cases, ASDAR, and EAPC (1990–2021).** | | | | | |
| --- | --- | --- | --- | --- | --- |
| Country | **1990** | | **2021** | | **EAPC_95%CI** |
|  | Number_95%UI | ASR | Number_95%UI | ASR |  |
| Afghanistan | 15863 (10785-23986) | 225.02 (155.79-347.36) | 23173 (12683-39344) | 249.37 (136.39-445.53) | 0.35 (0.31-0.39) |
| Albania | 699 (536-887) | 35.74 (27.61-45.73) | 1407 (1059-1812) | 32.02 (24.2-41.29) | -0.01 (-0.17-0.15) |
| Algeria | 12415 (8298-20904) | 114.93 (76.68-196.84) | 48027 (36161-63204) | 148.52 (112.11-194.1) | 1.08 (0.93-1.23) |
| American Samoa | 105 (77-145) | 498.45 (366.8-698.81) | 487 (372-608) | 1048.35 (811.35-1297.79) | 2.61 (2.39-2.83) |
| Andorra | 25 (19-33) | 47.33 (35.08-62.65) | 65 (49-84) | 40.14 (30.39-51.93) | -0.35 (-0.5--0.21) |
| Angola | 5548 (3920-7624) | 154.68 (112.07-210.67) | 16939 (11751-23826) | 159.1 (112.24-220.16) | -0.04 (-0.15-0.06) |
| Antigua and Barbuda | 154 (132-180) | 286.6 (246.36-335.85) | 454 (388-527) | 432.63 (375.06-499.17) | 1.81 (1.57-2.05) |
| Argentina | 40689 (32317-50618) | 126.9 (101.75-157.17) | 64073 (51182-78135) | 112.58 (90.2-137.15) | -0.22 (-0.48-0.05) |
| Armenia | 797 (541-1070) | 29.77 (20.28-40.73) | 2567 (1989-3266) | 58.95 (45.86-74.8) | 2.35 (2.07-2.63) |
| Australia | 3200 (2463-4041) | 16.63 (12.79-20.86) | 10021 (7701-12683) | 21.37 (16.41-27.18) | 1.19 (0.93-1.44) |
| Austria | 4347 (3425-5345) | 35.95 (28.29-43.99) | 11243 (9447-13212) | 54.61 (46.12-64.02) | 2.17 (1.74-2.6) |
| Azerbaijan | 2442 (1844-3153) | 48.77 (37.17-63.25) | 6588 (4947-8446) | 63.96 (48.68-80.93) | 0.99 (0.85-1.13) |
| Bahamas | 265 (182-374) | 179.31 (125.04-250.74) | 1443 (1075-1952) | 201.16 (148.13-271.18) | -0.14 (-0.48-0.2) |
| Bahrain | 48789 (38167-65761) | 105.55 (83.51-141.13) | 122172 (92990-169227) | 90.66 (69.48-123.71) | -0.33 (-0.55--0.1) |
| Bangladesh | 576 (505-652) | 196.78 (171.47-222.64) | 1428 (1088-1793) | 272.49 (208.05-342.52) | 1.55 (1.28-1.82) |
| Barbados | 2280 (1552-3095) | 17.72 (12.08-23.93) | 3448 (2512-4624) | 21.24 (15.44-28.24) | 0.53 (0.39-0.68) |
| Belarus | 6754 (5243-8262) | 42.9 (33.35-52.33) | 10262 (7826-12785) | 40.59 (31.1-50.26) | 0.39 (0.15-0.63) |
| Belgium | 218 (190-251) | 237.16 (206.3-273.55) | 1265 (1034-1506) | 430.21 (356.15-509.41) | 2.38 (1.94-2.83) |
| Belize | 2248 (1757-2955) | 118.16 (91.83-152.42) | 5992 (4469-8244) | 125.61 (93.7-169.86) | 0.12 (0.03-0.22) |
| Benin | 95 (83-110) | 153.74 (134.18-178.1) | 205 (167-256) | 146.17 (119.77-181.06) | 0.26 (-0.02-0.54) |
| Bermuda | 323 (225-462) | 135.41 (95.93-193.77) | 897 (627-1280) | 151.42 (105.68-215.48) | 0.38 (0.32-0.44) |
| Bhutan | 8994 (7252-10932) | 95.78 (77.57-116.19) | 62271 (45106-87562) | 206.73 (151.24-289.69) | 2.2 (1.82-2.59) |
| Bolivia (Plurinational State of) | 1725 (1321-2199) | 44.37 (34.27-56.17) | 3137 (2412-3888) | 48.81 (37.65-60.31) | 0.4 (0.12-0.68) |
| Bosnia and Herzegovina | 437 (310-622) | 82.83 (60.97-116.5) | 1346 (959-1838) | 98.04 (71.09-130.23) | 0.58 (0.43-0.73) |
| Botswana | 122841 (103531-142764) | 143.93 (121.6-165.84) | 386540 (326554-444653) | 154.88 (130.72-177.55) | 0.24 (0.06-0.41) |
| Brazil | 301 (243-376) | 333.18 (271.01-419.71) | 907 (746-1108) | 308.44 (253.55-372.92) | 0.14 (-0.03-0.3) |
| Brunei Darussalam | 3268 (2528-4128) | 27.36 (21.84-34.17) | 7243 (5559-9370) | 50.29 (38.45-64.9) | 2.52 (2.22-2.81) |
| Bulgaria | 4484 (3297-5904) | 111.08 (83.73-145.16) | 10274 (7754-14027) | 119.78 (90.2-161.44) | 0.23 (0.17-0.28) |
| Burkina Faso | 4948 (3589-6692) | 225.19 (165.91-306.95) | 8487 (5802-12207) | 197.75 (133.72-287.07) | -0.81 (-0.95--0.66) |
| Burundi | 8261 (6221-10636) | 180.12 (137.35-226.58) | 23012 (16878-30137) | 186.4 (138-241.24) | 0.02 (-0.12-0.15) |
| C?te d'Ivoire | 8716 (6153-12000) | 211.51 (150.44-288.97) | 26909 (18415-38375) | 231.05 (161.07-328.25) | 0.42 (0.22-0.62) |
| Cabo Verde | 13319 (11002-15753) | 40.67 (33.6-48.06) | 38750 (31821-46474) | 52.26 (43.41-61.64) | 1.22 (1.02-1.41) |
| Cambodia | 2252 (1634-3137) | 209.4 (154.67-283.1) | 4303 (3001-6163) | 208.98 (150.51-291.8) | -0.03 (-0.07-0.01) |
| Cameroon | 2631 (1930-3672) | 96.99 (72.42-133) | 6183 (4359-8922) | 114.85 (83.32-162.97) | 0.39 (0.24-0.55) |
| Canada | 6762 (5433-8404) | 69.24 (55.96-85.76) | 20393 (16422-25032) | 78.12 (62.81-96.02) | 0.78 (0.4-1.17) |
| Central African Republic | 1231518 (1022061-1492725) | 155.94 (131.16-188.26) | 2537070 (2044338-3072897) | 122.15 (99.62-146.99) | -0.7 (-0.8--0.6) |
| Chad | 16991 (13775-20574) | 101.51 (83.57-122.1) | 46902 (37145-59083) | 84.85 (67.04-107.5) | -0.46 (-0.59--0.34) |
| Chile | 351 (300-411) | 229.83 (195.99-266.61) | 1412 (1121-1777) | 348.85 (281.73-435.73) | 1.9 (1.69-2.12) |
| China | 363 (258-496) | 200.54 (146.57-269.04) | 933 (647-1334) | 207.13 (144.6-295.54) | -0.07 (-0.18-0.03) |
| Colombia | 2392 (1701-3236) | 240.47 (171.27-320.29) | 5738 (3761-7877) | 233.63 (152.76-320.62) | -0.29 (-0.41--0.18) |
| Comoros | 31 (25-41) | 261.39 (209.26-341.2) | 82 (64-101) | 315.58 (248.56-388.02) | 0.67 (0.59-0.75) |
| Congo | 1738 (1403-2077) | 101.84 (82.6-121.37) | 9424 (7603-11557) | 170.52 (137.55-209.26) | 1.79 (1.49-2.08) |
| Cook Islands | 1938 (1522-2367) | 32.99 (25.76-40.01) | 3814 (2918-4906) | 40.27 (30.85-51.53) | 0.52 (0.3-0.74) |
| Costa Rica | 8965 (7862-10179) | 88.2 (77.38-100.03) | 32301 (27395-38537) | 163.32 (139.29-194.97) | 2.5 (2.22-2.79) |
| Croatia | 688 (513-931) | 110.84 (83.34-148.57) | 1255 (962-1616) | 65.23 (50.41-82.82) | -1.86 (-2.04--1.67) |
| Cuba | 4307 (3376-5370) | 31.06 (24.64-38.55) | 5831 (4484-7373) | 26.27 (20.38-32.99) | -0.51 (-0.62--0.41) |
| Cyprus | 27580 (20671-35602) | 177.94 (134.37-229.99) | 59437 (46855-75619) | 181.29 (143.3-229.39) | 0.16 (0.06-0.26) |
| Czechia | 27599 (20371-37376) | 194.58 (149.07-260.2) | 66682 (46002-95341) | 199.95 (139.57-283.06) | -0.04 (-0.13-0.04) |
| Democratic People's Republic of Korea | 2410 (1804-3030) | 28.82 (21.65-36.2) | 5961 (4950-7076) | 46.18 (38.24-55.1) | 1.52 (1.38-1.67) |
| Democratic Republic of the Congo | 196 (136-283) | 166.84 (118.97-237.52) | 1295 (901-1868) | 236.91 (168.52-334.59) | 1.13 (1.01-1.25) |
| Denmark | 183 (154-220) | 307.82 (259.29-366.85) | 393 (298-500) | 469.96 (361.26-592.11) | 1.58 (1.5-1.66) |
| Djibouti | 4813 (3838-6178) | 135.77 (109.23-174.57) | 20647 (13773-27778) | 207.83 (139.1-278.74) | 2.01 (1.82-2.21) |
| Dominica | 8544 (7174-9981) | 170.63 (144.2-198.93) | 50343 (36300-72170) | 314.54 (229.48-447.7) | 2.06 (1.2-2.93) |
| Dominican Republic | 45996 (32278-70286) | 189.75 (133.89-291.06) | 162106 (118195-219966) | 281.34 (206.2-377.51) | 1.51 (1.42-1.6) |
| Ecuador | 3790 (2937-5389) | 129.44 (99.81-184.53) | 21261 (14585-28952) | 346.48 (237.44-473.54) | 3.33 (2.88-3.78) |
| Egypt | 354 (259-491) | 193.28 (142.03-259.68) | 1073 (641-1585) | 236.23 (135.11-341.58) | 0.91 (0.54-1.27) |
| El Salvador | 2006 (1401-2980) | 193.49 (137.07-280.68) | 5219 (3488-8383) | 216.32 (143.82-349.72) | 0.35 (0.27-0.43) |
| Equatorial Guinea | 566 (413-722) | 27.54 (20.14-34.99) | 1779 (1467-2180) | 59.07 (48.53-71.64) | 2.45 (2.23-2.66) |
| Eritrea | 103428 (80074-127616) | 563.31 (449.88-690.9) | 127071 (101302-155533) | 326.02 (261.55-393.64) | -2.18 (-2.32--2.03) |
| Estonia | 225 (155-343) | 479.4 (328.63-748.34) | 577 (407-824) | 803.05 (581.84-1141.94) | 1.65 (1.26-2.03) |
| Eswatini | 1383 (974-1982) | 400.9 (287.73-581.71) | 4790 (3510-6395) | 656.72 (493.13-858.82) | 1.21 (0.91-1.52) |
| Ethiopia | 1870 (1436-2308) | 25.77 (19.73-31.79) | 4260 (3513-5133) | 29.85 (24.29-36.1) | 0.93 (0.75-1.11) |
| Fiji | 25175 (19773-31858) | 28.95 (22.79-36.21) | 45905 (37302-55191) | 28.25 (22.94-34.48) | -0.05 (-0.22-0.12) |
| Finland | 1193 (878-1605) | 219.19 (162.68-291.45) | 2907 (1589-4131) | 306.17 (164.28-442.77) | 1.05 (0.89-1.2) |
| France | 2390 (1812-3092) | 38.39 (28.86-49.52) | 4087 (3156-5198) | 69.13 (53.49-87.89) | 2.36 (1.99-2.72) |
| Gabon | 53682 (41747-65529) | 41.17 (32.42-50.09) | 115380 (88837-146701) | 51.87 (40.85-64.94) | 1.02 (0.81-1.24) |
| Gambia | 6378 (4510-9318) | 113.2 (81.8-161.8) | 28051 (20404-38587) | 186.47 (137.09-257.94) | 2.08 (1.89-2.27) |
| Georgia | 12478 (9978-15487) | 82.37 (66.67-102.49) | 23490 (19694-26872) | 85.83 (72.84-97.41) | -0.26 (-0.86-0.34) |
| Germany | 23 (19-29) | 77.98 (63.3-96.86) | 56 (42-72) | 85.05 (65.36-106.95) | 0.67 (0.52-0.82) |
| Ghana | 237 (200-282) | 335.76 (280.35-395.27) | 624 (515-758) | 548.59 (455.63-660.56) | 1.93 (1.74-2.12) |
| Greece | 163 (138-203) | 231.52 (197.78-285.96) | 645 (544-744) | 302.94 (256.13-349.56) | 1.65 (1.25-2.04) |
| Greenland | 5033 (4044-6137) | 158.95 (128.56-194.56) | 27323 (20646-35909) | 252.33 (191.26-332.72) | 2.29 (1.89-2.7) |
| Grenada | 2569 (1905-3482) | 81.18 (60.7-109.27) | 4683 (3348-6607) | 88.7 (63.56-124.63) | 0.21 (0.17-0.26) |
| Guam | 627 (457-852) | 166.85 (125.08-224.95) | 1034 (760-1452) | 154.58 (115.09-209.27) | -0.31 (-0.35--0.27) |
| Guatemala | 1067 (889-1285) | 290.3 (244.74-346.25) | 3535 (2693-4616) | 544.36 (422.96-704.55) | 3.05 (2.7-3.4) |
| Guinea | 8416 (5928-13814) | 266.3 (188.68-446.93) | 20307 (11562-42729) | 290.87 (165.41-614.2) | 0.51 (0.44-0.59) |
| Guinea-Bissau | 1612 (1235-2057) | 80.45 (62.26-102.54) | 8839 (6519-11689) | 143.28 (105.67-189.72) | 2.15 (1.98-2.32) |
| Guyana | 3438 (2688-4361) | 23.42 (18.21-29.57) | 6072 (4729-7777) | 29.61 (23.17-37.59) | 1.34 (1.07-1.6) |
| Haiti | 52 (39-66) | 17.76 (13.38-22.47) | 128 (97-158) | 21.17 (15.88-26.35) | 0.59 (0.48-0.7) |
| Honduras | 491273 (396488-598466) | 106.74 (86.92-129.16) | 1592176 (1286480-1952847) | 133.63 (108.33-164.01) | 0.71 (0.62-0.8) |
| Hungary | 151287 (123167-190237) | 151.56 (124.57-191.36) | 472034 (375409-592078) | 194.82 (155.78-244.68) | 0.84 (0.78-0.89) |
| Iceland | 16050 (11840-22025) | 206.12 (151.01-283.15) | 50450 (31992-69022) | 225.51 (145.2-308.3) | 0.05 (-0.1-0.2) |
| India | 1953 (1491-2464) | 47.15 (35.96-58.66) | 3681 (2818-4600) | 44.87 (34.55-56.72) | 0.09 (0.01-0.17) |
| Indonesia | 24635 (18257-33174) | 102.1 (76.39-136.28) | 79344 (61425-96709) | 107.81 (83.56-132.6) | 0.34 (0.24-0.44) |
| Iran (Islamic Republic of) | 4443 (3581-5435) | 92.45 (75.39-113.62) | 10919 (8730-13517) | 83.34 (67.23-102.34) | 0.11 (-0.36-0.59) |
| Iraq | 33918 (27154-41662) | 37.59 (30.03-45.82) | 52794 (40916-67565) | 31.66 (24.85-39.27) | -0.53 (-0.6--0.47) |
| Ireland | 3783 (3238-4383) | 209.15 (179.05-241.82) | 8269 (6134-10964) | 265.55 (195.97-353.34) | 0.28 (-0.3-0.86) |
| Israel | 146074 (123219-168923) | 87.95 (73.98-101.6) | 307816 (242832-371080) | 73.52 (60.41-85.93) | -0.51 (-0.63--0.38) |
| Italy | 2725 (2076-3558) | 221.42 (167.7-285.37) | 14984 (11302-19351) | 221.03 (166.13-289.49) | -0.24 (-0.59-0.12) |
| Jamaica | 6025 (4552-7690) | 47.17 (35.86-59.56) | 12257 (9434-15299) | 69.08 (53.4-86.44) | 1.12 (0.96-1.29) |
| Japan | 10375 (7697-16239) | 139.26 (103.48-214.01) | 42905 (31509-58832) | 210.7 (154.3-279.17) | 1.55 (1.47-1.64) |
| Jordan | 388 (289-521) | 143.94 (108.48-193.16) | 1073 (672-1518) | 198.16 (125.95-276.77) | 1.2 (0.59-1.82) |
| Kazakhstan | 159 (128-196) | 441.38 (357.71-543.33) | 425 (280-629) | 608.02 (403.18-886.57) | 0.82 (0.54-1.1) |
| Kenya | 883 (690-1111) | 152.48 (120.99-189.87) | 2166 (1627-2837) | 78.32 (56.75-103.4) | -2.05 (-2.28--1.81) |
| Kiribati | 1218 (922-1552) | 40.73 (31.09-52.22) | 2889 (2248-3660) | 58.55 (45.51-73.78) | 0.56 (0.1-1.01) |
| Kuwait | 8072 (5918-10908) | 387.74 (294.32-514.81) | 16045 (11524-22186) | 352.12 (253.22-480.14) | -0.43 (-0.47--0.39) |
| Kyrgyzstan | 761 (544-1010) | 21.14 (15.14-28.08) | 1444 (1074-1844) | 34.08 (25.43-43.43) | 1.8 (1.66-1.95) |
| Lao People's Democratic Republic | 2906 (2005-4124) | 140.7 (97.94-196.53) | 7757 (5934-10050) | 125.43 (95.9-163.28) | -0.21 (-0.33--0.1) |
| Latvia | 533 (393-711) | 65.09 (48.52-86.27) | 1306 (909-1874) | 124.82 (88.9-178.04) | 2.73 (2.38-3.07) |
| Lebanon | 1568 (1149-2126) | 144.35 (107.29-191.01) | 3027 (2136-4131) | 157 (112.3-218.43) | 0.45 (0.21-0.68) |
| Lesotho | 2147 (1498-3097) | 117.23 (82.52-169.03) | 9732 (5951-13686) | 191.62 (117.57-267.81) | 2.17 (1.98-2.36) |
| Liberia | 921 (663-1205) | 20.32 (14.6-26.53) | 1710 (1310-2196) | 28.38 (21.68-36.43) | 0.87 (0.74-0.99) |
| Libya | 238 (188-298) | 43.62 (34.4-54.44) | 538 (419-684) | 47.6 (37.14-60.31) | 0.51 (0.38-0.63) |
| Lithuania | 7126 (5105-10464) | 151.74 (109.5-226.24) | 15263 (10485-21093) | 155.1 (108.04-216.35) | 0.1 (0.02-0.18) |
| Luxembourg | 7141 (5095-9649) | 203.94 (151.31-268.79) | 16263 (11824-21690) | 242.87 (180.9-318.94) | 0.36 (0.15-0.58) |
| Madagascar | 20635 (17394-24266) | 227.65 (193.32-265.45) | 76738 (63050-91299) | 274.36 (225.51-323.23) | 0.41 (0.25-0.58) |
| Malawi | 354 (278-459) | 407.37 (321.8-549.83) | 725 (572-908) | 229.6 (181.91-282.48) | -2.19 (-2.4--1.98) |
| Malaysia | 4637 (3366-6212) | 125.71 (94.52-165.1) | 10175 (7532-14321) | 125.63 (95.33-175.96) | 0.09 (-0.02-0.2) |
| Maldives | 209 (165-259) | 50.25 (40.01-62.05) | 528 (413-652) | 50.62 (39.7-62.53) | -0.02 (-0.18-0.13) |
| Mali | 74 (48-133) | 470.45 (302.1-844.64) | 262 (90-741) | 759.89 (264.01-2194.26) | 1.56 (1.35-1.77) |
| Malta | 1339 (942-1859) | 140.97 (100.01-192.54) | 2662 (1759-3930) | 132.25 (86.82-195.68) | -0.53 (-0.68--0.38) |
| Marshall Islands | 2905 (2492-3342) | 405.49 (353.01-465.28) | 13623 (11679-15634) | 726.57 (630.29-830.36) | 2.14 (1.73-2.55) |
| Mauritania | 74466 (61009-88424) | 190.73 (157.77-225.84) | 385119 (296154-481773) | 304.1 (236.78-379.24) | 2.06 (1.4-2.72) |
| Mauritius | 805 (610-1051) | 76.54 (58.5-99.26) | 1514 (1136-1926) | 65.73 (50.22-82.17) | -0.8 (-0.92--0.69) |
| Mexico | 230 (175-294) | 37.5 (28.65-47.96) | 468 (343-617) | 48.49 (35.88-63.57) | 0.98 (0.9-1.06) |
| Micronesia (Federated States of) | 11399 (7439-19128) | 82.2 (53.19-141.39) | 45810 (32574-62271) | 136.87 (96.96-183.21) | 2.12 (1.94-2.3) |
| Monaco | 7252 (5208-10715) | 138.44 (103.83-203.52) | 18639 (13397-28605) | 192.16 (138.88-293.24) | 1.47 (1.32-1.62) |
| Mongolia | 53313 (39221-71348) | 227.89 (169.94-297.8) | 102103 (79801-132671) | 211.83 (167.73-273.01) | -0.46 (-0.54--0.39) |
| Montenegro | 500 (364-695) | 81.41 (60.24-110.69) | 1221 (885-1650) | 93.54 (68.08-126.32) | 0.27 (0.04-0.51) |
| Morocco | 11132 (8241-14841) | 119.56 (90.67-158.31) | 36971 (27229-50287) | 160.44 (117.7-215.41) | 0.96 (0.62-1.29) |
| Mozambique | 6029 (4607-7579) | 29.76 (22.78-37.43) | 13682 (10976-16691) | 36.66 (29.16-45.22) | 0.57 (0.32-0.82) |
| Myanmar | 1026 (802-1256) | 26.71 (20.87-32.52) | 2960 (2354-3582) | 35.13 (27.79-42.56) | 0.97 (0.55-1.39) |
| Namibia | 2384 (1881-3170) | 158.8 (125.44-210.58) | 14389 (10865-19099) | 295.37 (224.92-391.68) | 2.5 (2.12-2.87) |
| Nauru | 1991 (1446-2748) | 78.12 (56.43-105.73) | 5397 (3841-8021) | 71.64 (52.1-103.77) | -0.33 (-0.42--0.24) |
| Nepal | 44700 (34161-56887) | 109.32 (84.61-138.43) | 85603 (64812-109889) | 104.22 (80.15-134.15) | -0.62 (-0.85--0.4) |
| Netherlands | 89 (69-120) | 557.14 (453.99-696.14) | 428 (345-536) | 850.92 (706.61-1023.86) | 1.54 (1.38-1.7) |
| New Zealand | 1611 (1226-2039) | 23.14 (17.56-29.19) | 2699 (2075-3374) | 25.44 (19.46-31.74) | 0.34 (0.17-0.51) |
| Nicaragua | 792 (559-1149) | 123.06 (86.03-176.7) | 3570 (2636-5034) | 199.53 (147.47-271.83) | 2.08 (1.86-2.3) |
| Niger | 75133 (56065-98474) | 136.2 (102.38-178.55) | 224593 (171262-295484) | 189.61 (145.78-248.34) | 0.85 (0.64-1.06) |
| Nigeria | 1509 (1077-2125) | 184.37 (132.46-258.33) | 4082 (3153-5429) | 176.37 (135.79-235.15) | -0.16 (-0.4-0.07) |
| Niue | 1216 (975-1501) | 83.34 (67.12-102.08) | 7141 (5383-9421) | 161.34 (121.75-213.66) | 2.39 (2.05-2.74) |
| North Macedonia | 3191 (2263-4437) | 175.43 (127.37-241.47) | 10399 (7931-14118) | 203.52 (156.86-278.22) | 0.47 (0.43-0.5) |
| Northern Mariana Islands | 2754 (2158-3391) | 128.91 (101.14-158.89) | 11274 (8083-15123) | 198.97 (143.8-266.8) | 1.49 (1.38-1.6) |
| Norway | 22077 (17491-27457) | 194.16 (153.06-240.8) | 75915 (51852-105252) | 229.73 (156.89-320.57) | 0.33 (0.13-0.53) |
| Oman | 67220 (56022-81325) | 243.11 (205.41-288.46) | 288085 (232045-358895) | 350.69 (285.58-429.65) | 1.58 (1.44-1.72) |
| Pakistan | 10061 (7812-13252) | 334.91 (262.66-439.1) | 39242 (27837-54433) | 453.96 (329.56-620.26) | 1.07 (0.97-1.17) |
| Palau | 19390 (15535-23975) | 44.49 (35.84-54.64) | 21452 (16875-26512) | 29.29 (22.99-36.38) | -1.67 (-2--1.33) |
| Palestine | 7320 (5890-9044) | 53.35 (43.37-66.91) | 15125 (11645-19179) | 53.17 (42.38-66.17) | 0.16 (-0.27-0.59) |
| Panama | 24 (19-32) | 31.94 (24.81-41.37) | 44 (35-57) | 41 (31.85-52.4) | 0.92 (0.79-1.05) |
| Papua New Guinea | 10497 (9183-11850) | 292.8 (257.13-331.53) | 23930 (19912-28145) | 323.42 (266.7-379.13) | 1.02 (0.62-1.43) |
| Paraguay | 160 (100-238) | 177.57 (108.74-277.42) | 1241 (918-1745) | 167.9 (125.48-228.71) | -0.47 (-1-0.06) |
| Peru | 148 (117-188) | 63.78 (50.9-80.79) | 472 (313-638) | 110.48 (72.06-148.62) | 1.51 (1.29-1.74) |
| Philippines | 3979 (2957-5321) | 109.53 (83.35-145.63) | 11837 (8338-16025) | 114.89 (82.7-154.11) | -0.03 (-0.14-0.07) |
| Poland | 30721 (26136-35118) | 117.06 (100.85-135.19) | 77180 (63601-92172) | 82.17 (67.76-98.27) | -0.71 (-0.93--0.5) |
| Portugal | 1101 (769-1462) | 25.84 (18.12-34.05) | 1911 (1375-2558) | 31.82 (22.92-42.73) | 0.51 (0.41-0.62) |
| Puerto Rico | 26 (16-40) | 579.75 (368.37-937.62) | 49 (35-70) | 876.15 (608.8-1347.34) | 1.21 (1.07-1.34) |
| Qatar | 10 (7-13) | 427.28 (328.15-574) | 16 (9-24) | 738.08 (444.21-1110.5) | 1.72 (1.55-1.89) |
| Republic of Korea | 39 (29-52) | 420.8 (313.43-547.41) | 140 (99-189) | 662.69 (481.37-885.25) | 1.61 (1.42-1.8) |
| Republic of Moldova | 11 (8-13) | 29.38 (22.65-36.92) | 21 (15-28) | 26.44 (18.53-34.31) | 0.16 (0-0.33) |
| Romania | 365 (270-499) | 112.86 (84.31-154.28) | 1291 (922-1812) | 141.79 (100.54-200.16) | 0.58 (0.47-0.69) |
| Russian Federation | 5635 (4186-7470) | 20.68 (15.28-26.91) | 10492 (8223-12820) | 28.05 (21.76-34.04) | 1.56 (1.22-1.9) |
| Rwanda | 41389 (32208-51655) | 23.24 (18.14-28.95) | 68671 (54630-85188) | 28.15 (22.52-34.59) | 0.41 (0.22-0.59) |
| Saint Kitts and Nevis | 5988 (4340-8042) | 228.33 (168.64-306.13) | 9690 (6801-14207) | 173.21 (121.43-249.59) | -1.75 (-2.09--1.4) |
| Saint Lucia | 151 (129-177) | 397.06 (340.28-463.63) | 357 (276-447) | 534.53 (426.45-651.33) | 1.86 (1.57-2.15) |
| Saint Vincent and the Grenadines | 280 (240-320) | 329.78 (285.16-375.88) | 856 (680-1050) | 356.77 (286.03-436.66) | 0.6 (0.34-0.87) |
| Samoa | 170 (147-197) | 240.72 (208.9-279.21) | 513 (429-611) | 363.11 (304.23-429.91) | 1.89 (1.6-2.19) |
| San Marino | 356 (260-492) | 438.28 (323.24-594.69) | 845 (646-1109) | 602.1 (460.61-777.71) | 0.94 (0.83-1.06) |
| Sao Tome and Principe | 110 (85-143) | 177.97 (138.02-229.39) | 251 (175-332) | 246.31 (170.54-324.43) | 1.05 (0.95-1.15) |
| Saudi Arabia | 13836 (9623-19790) | 245.92 (171.11-347.85) | 81716 (56876-110119) | 420.05 (296.57-555.49) | 1.52 (1.33-1.71) |
| Senegal | 4409 (3256-5888) | 145.6 (107.83-188.41) | 11112 (8043-16022) | 154.68 (111.58-220.46) | 0.1 (0.01-0.18) |
| Serbia | 5272 (3993-6840) | 51.04 (38.97-66.71) | 9049 (7144-11169) | 52.74 (41.64-64.73) | 0.21 (0.07-0.35) |
| Seychelles | 153 (129-186) | 271.65 (228.78-329.66) | 462 (365-565) | 403.7 (318.54-493.82) | 1.4 (1.16-1.64) |
| Sierra Leone | 1942 (1434-2642) | 99.32 (74.09-134.06) | 3536 (2529-4922) | 99.9 (72.19-138.55) | 0.03 (-0.02-0.07) |
| Singapore | 2809 (2453-3191) | 137.29 (120.96-155.54) | 8557 (7273-9872) | 101.34 (85.65-116.99) | 0.73 (0.22-1.25) |
| Slovakia | 2473 (1877-3154) | 41.16 (31.52-52.29) | 3395 (2597-4280) | 35.18 (27.16-44.45) | -0.44 (-0.56--0.32) |
| Slovenia | 580 (462-701) | 23.52 (18.78-28.51) | 1037 (776-1354) | 22.14 (16.43-28.93) | -0.06 (-0.24-0.12) |
| Solomon Islands | 73983 (55001-98693) | 188.08 (140.71-247.68) | 199251 (143465-256308) | 208.09 (152.58-267.4) | 0.55 (0.44-0.67) |
| Somalia | 453 (212-712) | 327.42 (176.78-506.34) | 1291 (995-1682) | 360.05 (286.72-456.79) | 0.28 (0.1-0.46) |
| South Africa | 5523 (3722-8085) | 262.56 (178.43-391.2) | 15405 (9959-23537) | 282.27 (187.8-421.04) | 0.34 (0.25-0.43) |
| South Sudan | 15398 (11786-19932) | 77.1 (59.83-100.79) | 46790 (36941-60270) | 104.9 (83.91-134.6) | 1.44 (1.2-1.68) |
| Spain | 5696 (3858-8651) | 234.75 (161.44-346.89) | 10836 (7008-15460) | 315.04 (211.8-445.28) | 0.86 (0.68-1.05) |
| Sri Lanka | 28909 (22932-35792) | 52.56 (41.91-64.83) | 44729 (34598-57356) | 39.15 (30.55-49.17) | -0.58 (-0.76--0.4) |
| Sudan | 21804 (17226-27336) | 213.35 (169.14-267.16) | 50397 (34955-69334) | 186.65 (131.11-255.41) | -0.48 (-0.66--0.3) |
| Suriname | 6596 (4386-10890) | 72.16 (48.52-121.73) | 18703 (12989-25856) | 99.28 (70.26-139.43) | 0.87 (0.67-1.07) |
| Sweden | 684 (561-832) | 272.36 (226.42-330.05) | 2549 (1864-3371) | 399.72 (292.37-526.07) | 1.58 (1.37-1.79) |
| Switzerland | 3810 (2884-4789) | 23.9 (18.17-29.74) | 8373 (6496-10652) | 33.61 (26.56-42.39) | 1.64 (1.48-1.81) |
| Syrian Arab Republic | 3635 (2850-4523) | 33.92 (26.29-42.4) | 7807 (6312-9512) | 38.52 (30.92-47.14) | 0.83 (0.65-1.02) |
| Taiwan (Province of China) | 8567 (6308-11885) | 170.28 (124.05-230.8) | 24174 (15997-34100) | 191.93 (128.41-271.42) | -0.04 (-0.28-0.21) |
| Tajikistan | 38464 (34198-43008) | 272.8 (243.93-300.29) | 100771 (88270-115124) | 232.11 (203.76-263.98) | 0.08 (-0.19-0.36) |
| Thailand | 821 (579-1104) | 30.41 (21.45-41.12) | 1932 (1378-2599) | 33.73 (24.08-45.19) | 0.21 (0.13-0.3) |
| Timor-Leste | 72070 (58655-90946) | 211.98 (173.48-267.15) | 288711 (222565-367494) | 263.7 (203.89-335.88) | 0.51 (0.44-0.58) |
| Togo | 704 (543-899) | 38.61 (29.82-48.78) | 1473 (1087-1945) | 45.41 (33.79-58.77) | 0.61 (0.2-1.02) |
| Tokelau | 594 (437-852) | 217.59 (165.08-302.8) | 2065 (1466-2958) | 244.41 (177.12-348.9) | 0.54 (0.36-0.72) |
| Tonga | 1239 (926-1623) | 109.17 (81.57-141.61) | 4307 (3121-5896) | 123.09 (89.64-167.63) | 0.26 (0.19-0.34) |
| Trinidad and Tobago | 5 (3-8) | 358.16 (250.88-594.15) | 7 (5-11) | 492.74 (355.49-701.4) | 1.06 (0.94-1.18) |
| Tunisia | 120 (88-161) | 218.87 (162.26-288.2) | 245 (178-318) | 308.87 (224.03-398.88) | 0.98 (0.77-1.19) |
| Türkiye | 1852 (1603-2130) | 228.36 (199.59-261.17) | 7615 (5633-10351) | 385.68 (285.74-522.43) | 2.29 (1.94-2.64) |
| Turkmenistan | 4546 (3261-6717) | 95.71 (69.12-141.49) | 17391 (11983-24083) | 134.86 (92.12-187.17) | 1.12 (0.94-1.3) |
| Tuvalu | 45658 (34029-62554) | 139.36 (103.8-194.3) | 124432 (96227-167026) | 136.11 (105.26-179.89) | 0.51 (0.21-0.82) |
| Uganda | 1117 (874-1392) | 57.42 (45.23-71.58) | 3554 (2700-4641) | 86.39 (65.9-111.27) | 1.12 (0.77-1.47) |
| Ukraine | 27 (20-41) | 407.01 (299.89-637.74) | 58 (42-85) | 572.64 (417.16-838.12) | 1.17 (1.08-1.27) |
| United Arab Emirates | 11014 (7848-15257) | 186.83 (135.31-254.98) | 29618 (21325-41720) | 227.29 (165.89-314.63) | 0.32 (0.16-0.48) |
| United Kingdom | 11568 (7684-16179) | 16.4 (10.93-22.77) | 16823 (12153-22956) | 21.4 (15.41-29.08) | 1 (0.89-1.11) |
| United Republic of Tanzania | 578 (401-818) | 137.82 (94.36-203.3) | 5793 (4101-7780) | 198.97 (121.58-279.67) | 3.07 (2.46-3.68) |
| United States of America | 29811 (22914-37592) | 32.63 (25.08-40.73) | 49366 (38555-60219) | 36.59 (28.1-44.94) | 0.73 (0.59-0.87) |
| United States Virgin Islands | 9416 (6734-13604) | 94.12 (68.88-133.11) | 23498 (17188-33129) | 101.39 (75.34-142.09) | 0.12 (0.07-0.17) |
| Uruguay | 213560 (178505-248197) | 67.48 (56.41-77.99) | 1114832 (1005493-1218803) | 188.98 (171.11-207.31) | 3.8 (3.58-4.03) |
| Uzbekistan | 150 (122-188) | 184.48 (151.45-226.69) | 328 (229-441) | 183.21 (129.35-245.87) | 0.28 (0.02-0.53) |
| Vanuatu | 2262 (2037-2475) | 57.1 (51.24-62.38) | 4053 (3269-4917) | 69.44 (56.1-85.02) | 1.18 (0.89-1.48) |
| Venezuela (Bolivarian Republic of) | 5015 (3651-6754) | 44.17 (32-59.58) | 19527 (15091-24922) | 74.55 (58.04-94.82) | 1.25 (0.77-1.72) |
| Viet Nam | 195 (129-336) | 326.21 (220.58-555.51) | 837 (601-1281) | 492.05 (358.57-744.67) | 1.36 (1.3-1.41) |
| Yemen | 4688 (3073-8242) | 97.86 (65.16-178.92) | 12922 (8756-20311) | 95.71 (65.15-147.63) | -0.19 (-0.3--0.08) |
| Zambia | 6621 (4976-8810) | 256.74 (195.51-337.46) | 18667 (13009-27452) | 298.41 (215.11-424.7) | 0.13 (-0.03-0.29) |
| Zimbabwe | 3359 (2484-4538) | 88.67 (66-119.13) | 8533 (5985-11691) | 131.14 (93.52-178.41) | 1.5 (1.13-1.87) |

| **TableS23: Trends in the burden of chronic kidney disease due to glomerulonephritis across different age groups: incidence, prevalence, deaths, and disability-adjusted life years (1990–2021).** | | | | | |
| --- | --- | --- | --- | --- | --- |
| Age | 1990 | | 2021 | | **EAPC_95%CI** |
|  | Number_95%UI | ASR | Number_95%UI | ASR |  |
| **Incidence** |  |  |  |  |  |
| <5 years | 129844 (106489-159004) | 20.94 (17.18-25.65) | 128410 (106448-151069) | 19.51 (16.17-22.95) | -0.19 (-0.21--0.17) |
| 5–9 years | 16687 (8461-27701) | 2.86 (1.45-4.75) | 21822 (11773-34658) | 3.18 (1.71-5.04) | 0.27 (0.16-0.39) |
| 10–14 years | 13526 (6975-22698) | 2.53 (1.3-4.24) | 23995 (14191-36306) | 3.6 (2.13-5.45) | 1.2 (1.16-1.23) |
| 15–19 years | 12101 (6202-19920) | 2.33 (1.19-3.83) | 21934 (13584-32007) | 3.52 (2.18-5.13) | 1.34 (1.3-1.39) |
| 20–24 years | 9124 (4298-14595) | 1.85 (0.87-2.97) | 14975 (8510-21977) | 2.51 (1.43-3.68) | 1 (0.98-1.02) |
| 25–29 years | 7316 (3522-12432) | 1.65 (0.8-2.81) | 11739 (5882-19142) | 2 (1-3.25) | 0.69 (0.65-0.73) |
| 30–34 years | 7069 (3992-10823) | 1.83 (1.04-2.81) | 13422 (8035-19508) | 2.22 (1.33-3.23) | 0.74 (0.66-0.82) |
| 35–39 years | 7915 (4850-11394) | 2.25 (1.38-3.23) | 15978 (10197-21686) | 2.85 (1.82-3.87) | 0.81 (0.72-0.89) |
| 40–44 years | 7425 (4990-10701) | 2.59 (1.74-3.74) | 16439 (11728-21947) | 3.29 (2.34-4.39) | 0.75 (0.68-0.81) |
| 45–49 years | 6541 (4272-8843) | 2.82 (1.84-3.81) | 16520 (11435-21182) | 3.49 (2.41-4.47) | 0.69 (0.65-0.73) |
| 50–54 years | 6451 (4258-8894) | 3.03 (2-4.18) | 17129 (11978-22919) | 3.85 (2.69-5.15) | 0.82 (0.77-0.88) |
| 55–59 years | 5905 (3987-7830) | 3.19 (2.15-4.23) | 15654 (11586-19587) | 3.96 (2.93-4.95) | 0.8 (0.75-0.84) |
| 60–64 years | 5252 (3628-6997) | 3.27 (2.26-4.36) | 13200 (9729-16965) | 4.12 (3.04-5.3) | 0.71 (0.69-0.74) |
| 65–69 years | 4060 (2813-5363) | 3.28 (2.28-4.34) | 11033 (8262-14260) | 4 (3-5.17) | 0.68 (0.66-0.71) |
| 70–74 years | 2567 (1882-3306) | 3.03 (2.22-3.91) | 7865 (6132-9911) | 3.82 (2.98-4.82) | 0.76 (0.75-0.78) |
| 75–79 years | 1533 (1085-2027) | 2.49 (1.76-3.29) | 4224 (3161-5306) | 3.2 (2.4-4.02) | 0.78 (0.73-0.82) |
| 80–84 years | 682 (487-921) | 1.93 (1.38-2.6) | 2125 (1524-2769) | 2.43 (1.74-3.16) | 0.73 (0.69-0.77) |
| 85–89 years | 196 (132-266) | 1.3 (0.88-1.76) | 654 (466-860) | 1.43 (1.02-1.88) | 0.33 (0.3-0.36) |
| 90–94 years | 34 (24-46) | 0.8 (0.55-1.08) | 157 (117-201) | 0.88 (0.66-1.12) | 0.39 (0.27-0.5) |
| 95+ years | 2 (1-3) | 0.22 (0.13-0.34) | 14 (8-20) | 0.26 (0.15-0.36) | 0.55 (0.39-0.71) |
| **Prevalence** |  |  |  |  |  |
| <5 years | 71528 (62362-82020) | 11.54 (10.06-13.23) | 64005 (54272-75502) | 9.72 (8.25-11.47) | -0.69 (-0.76--0.63) |
| 5–9 years | 195923 (174875-221267) | 33.58 (29.97-37.92) | 182223 (158011-211451) | 26.52 (23-30.78) | -0.86 (-0.92--0.81) |
| 10–14 years | 296681 (268488-330371) | 55.38 (50.12-61.67) | 309199 (275155-349482) | 46.38 (41.28-52.42) | -0.64 (-0.68--0.61) |
| 15–19 years | 447621 (398345-503934) | 86.18 (76.69-97.02) | 501865 (433421-572101) | 80.43 (69.46-91.69) | -0.27 (-0.31--0.24) |
| 20–24 years | 606963 (522080-705004) | 123.34 (106.09-143.27) | 753494 (628477-901498) | 126.18 (105.24-150.96) | 0.03 (0-0.07) |
| 25–29 years | 689861 (577703-817185) | 155.86 (130.52-184.62) | 958016 (801292-1155670) | 162.83 (136.19-196.43) | 0.14 (0.12-0.15) |
| 30–34 years | 705935 (596170-829922) | 183.16 (154.68-215.33) | 1134716 (951917-1358034) | 187.72 (157.48-224.66) | 0.12 (0.09-0.16) |
| 35–39 years | 682652 (575789-805543) | 193.8 (163.46-228.69) | 1129811 (952010-1342939) | 201.44 (169.74-239.44) | 0.15 (0.11-0.19) |
| 40–44 years | 558493 (461427-663757) | 194.95 (161.07-231.69) | 1019759 (832412-1210134) | 203.85 (166.4-241.91) | 0.14 (0.11-0.18) |
| 45–49 years | 458303 (383052-545587) | 197.38 (164.97-234.97) | 959685 (795892-1154245) | 202.68 (168.09-243.77) | 0.12 (0.09-0.16) |
| 50–54 years | 407608 (335558-483413) | 191.75 (157.86-227.41) | 867596 (706717-1046616) | 195 (158.84-235.24) | 0.14 (0.09-0.19) |
| 55–59 years | 347209 (287906-413282) | 187.48 (155.46-223.15) | 749072 (615621-905287) | 189.29 (155.57-228.76) | 0.15 (0.11-0.19) |
| 60–64 years | 296878 (248183-360123) | 184.84 (154.53-224.22) | 601532 (500390-724805) | 187.95 (156.35-226.47) | 0.12 (0.09-0.16) |
| 65–69 years | 224235 (186763-264725) | 181.41 (151.09-214.16) | 506824 (421329-601737) | 183.74 (152.74-218.15) | 0.14 (0.09-0.19) |
| 70–74 years | 153821 (129030-182643) | 181.69 (152.41-215.73) | 387161 (322672-459357) | 188.09 (156.76-223.16) | 0.24 (0.18-0.31) |
| 75–79 years | 114567 (94018-138897) | 186.12 (152.74-225.64) | 263550 (213940-323411) | 199.83 (162.22-245.22) | 0.32 (0.26-0.37) |
| 80–84 years | 67866 (55215-83780) | 191.84 (156.08-236.83) | 181549 (146214-229713) | 207.29 (166.94-262.28) | 0.36 (0.3-0.42) |
| 85–89 years | 31246 (24257-40135) | 206.77 (160.53-265.6) | 101986 (78837-132244) | 223.06 (172.43-289.24) | 0.32 (0.28-0.35) |
| 90–94 years | 11177 (8029-15652) | 260.82 (187.37-365.26) | 50737 (35573-71523) | 283.62 (198.85-399.81) | 0.3 (0.27-0.32) |
| 95+ years | 2316 (1440-3868) | 227.52 (141.47-379.93) | 13027 (8189-21917) | 239.01 (150.25-402.12) | 0.27 (0.19-0.35) |
| **Deaths** |  |  |  |  |  |
| <5 years | 5300 (3499-6901) | 0.85 (0.56-1.11) | 2984 (2031-4015) | 0.45 (0.31-0.61) | -1.8 (-1.88--1.73) |
| 5–9 years | 1849 (1128-2633) | 0.32 (0.19-0.45) | 1478 (951-2058) | 0.22 (0.14-0.3) | -0.94 (-1.04--0.84) |
| 10–14 years | 1743 (1115-2294) | 0.33 (0.21-0.43) | 1928 (1284-2554) | 0.29 (0.19-0.38) | -0.17 (-0.24--0.1) |
| 15–19 years | 3213 (2204-4292) | 0.62 (0.42-0.83) | 4440 (3088-5925) | 0.71 (0.49-0.95) | 0.32 (0.23-0.42) |
| 20–24 years | 3378 (2387-4589) | 0.69 (0.49-0.93) | 5172 (3689-6947) | 0.87 (0.62-1.16) | 0.51 (0.38-0.64) |
| 25–29 years | 3191 (2251-4396) | 0.72 (0.51-0.99) | 5343 (3854-7181) | 0.91 (0.66-1.22) | 0.62 (0.5-0.74) |
| 30–34 years | 3080 (2164-4254) | 0.8 (0.56-1.1) | 5440 (3776-7366) | 0.9 (0.62-1.22) | 0.41 (0.34-0.48) |
| 35–39 years | 3344 (2233-4624) | 0.95 (0.63-1.31) | 6265 (4211-8565) | 1.12 (0.75-1.53) | 0.5 (0.4-0.6) |
| 40–44 years | 3486 (2257-4948) | 1.22 (0.79-1.73) | 7478 (4947-10405) | 1.49 (0.99-2.08) | 0.41 (0.24-0.57) |
| 45–49 years | 3537 (2192-5180) | 1.52 (0.94-2.23) | 8401 (5344-12167) | 1.77 (1.13-2.57) | 0.35 (0.26-0.44) |
| 50–54 years | 4787 (2927-6963) | 2.25 (1.38-3.28) | 11303 (7063-16274) | 2.54 (1.59-3.66) | 0.35 (0.29-0.41) |
| 55–59 years | 5728 (3707-8426) | 3.09 (2-4.55) | 14504 (9398-21364) | 3.67 (2.37-5.4) | 0.55 (0.44-0.65) |
| 60–64 years | 6789 (4221-9916) | 4.23 (2.63-6.17) | 16785 (10659-24551) | 5.24 (3.33-7.67) | 0.53 (0.44-0.63) |
| 65–69 years | 7248 (4400-11059) | 5.86 (3.56-8.95) | 18948 (11628-28176) | 6.87 (4.22-10.21) | 0.55 (0.44-0.65) |
| 70–74 years | 7256 (4284-10953) | 8.57 (5.06-12.94) | 20004 (12239-29802) | 9.72 (5.95-14.48) | 0.54 (0.48-0.6) |
| 75–79 years | 7238 (4398-10602) | 11.76 (7.14-17.22) | 17993 (11086-26078) | 13.64 (8.41-19.77) | 0.59 (0.55-0.64) |
| 80–84 years | 5970 (3687-9091) | 16.88 (10.42-25.7) | 17649 (10911-25716) | 20.15 (12.46-29.36) | 0.77 (0.7-0.83) |
| 85–89 years | 4010 (2477-6109) | 26.54 (16.39-40.43) | 15115 (9641-22219) | 33.06 (21.09-48.6) | 1.06 (0.95-1.17) |
| 90–94 years | 1538 (894-2523) | 35.89 (20.86-58.87) | 8832 (5289-14225) | 49.37 (29.56-79.52) | 1.38 (1.21-1.54) |
| 95+ years | 483 (248-899) | 47.43 (24.39-88.28) | 3935 (2093-7149) | 72.2 (38.41-131.17) | 1.54 (1.38-1.69) |
| **DALYs** |  |  |  |  |  |
| <5 years | 472484 (314109-612887) | 76.21 (50.67-98.86) | 268711 (185371-360348) | 40.83 (28.16-54.75) | -1.77 (-1.85--1.7) |
| 5–9 years | 162062 (101295-227529) | 27.77 (17.36-38.99) | 132106 (87837-180884) | 19.23 (12.78-26.33) | -0.9 (-0.99--0.81) |
| 10–14 years | 146814 (97721-191040) | 27.41 (18.24-35.66) | 161954 (111299-210430) | 24.29 (16.7-31.57) | -0.21 (-0.27--0.15) |
| 15–19 years | 251862 (177058-331332) | 48.49 (34.09-63.79) | 342584 (244079-447110) | 54.9 (39.12-71.65) | 0.27 (0.18-0.36) |
| 20–24 years | 257977 (190783-341459) | 52.42 (38.77-69.39) | 384865 (285657-505395) | 64.45 (47.84-84.63) | 0.44 (0.32-0.56) |
| 25–29 years | 242279 (185132-316792) | 54.74 (41.83-71.57) | 392557 (301182-512481) | 66.72 (51.19-87.11) | 0.53 (0.43-0.64) |
| 30–34 years | 228962 (169294-297479) | 59.41 (43.92-77.18) | 396874 (287167-513587) | 65.66 (47.51-84.96) | 0.36 (0.3-0.43) |
| 35–39 years | 231964 (169142-301869) | 65.85 (48.02-85.7) | 425795 (311508-558437) | 75.92 (55.54-99.57) | 0.46 (0.38-0.53) |
| 40–44 years | 217038 (156906-289734) | 75.76 (54.77-101.14) | 454083 (330627-601045) | 90.77 (66.09-120.15) | 0.39 (0.27-0.51) |
| 45–49 years | 194892 (135192-263525) | 83.93 (58.22-113.49) | 457017 (314859-616011) | 96.52 (66.5-130.1) | 0.36 (0.29-0.43) |
| 50–54 years | 223218 (150969-305331) | 105.01 (71.02-143.64) | 524359 (357354-723125) | 117.85 (80.32-162.53) | 0.37 (0.3-0.43) |
| 55–59 years | 228989 (156533-318101) | 123.64 (84.52-171.76) | 572365 (397106-797757) | 144.64 (100.35-201.59) | 0.55 (0.46-0.63) |
| 60–64 years | 230059 (156402-324761) | 143.24 (97.38-202.21) | 558620 (374732-783667) | 174.54 (117.09-244.86) | 0.53 (0.44-0.62) |
| 65–69 years | 204643 (133169-297640) | 165.56 (107.73-240.79) | 529025 (344665-751115) | 191.79 (124.95-272.3) | 0.54 (0.44-0.64) |
| 70–74 years | 166401 (106028-241017) | 196.55 (125.24-284.68) | 459030 (303564-659186) | 223 (147.48-320.24) | 0.55 (0.48-0.61) |
| 75–79 years | 134350 (86200-190438) | 218.26 (140.04-309.38) | 335154 (221620-462441) | 254.13 (168.04-350.64) | 0.6 (0.56-0.64) |
| 80–84 years | 88169 (57903-127103) | 249.23 (163.68-359.29) | 259591 (175606-363336) | 296.39 (200.5-414.85) | 0.75 (0.68-0.82) |
| 85–89 years | 47472 (32342-68717) | 314.15 (214.03-454.74) | 176177 (121787-248808) | 385.32 (266.37-544.18) | 0.97 (0.88-1.06) |
| 90–94 years | 16677 (10688-25057) | 389.17 (249.41-584.74) | 92194 (60146-139117) | 515.35 (336.21-777.65) | 1.2 (1.07-1.33) |
| 95+ years | 4777 (2737-8071) | 469.19 (268.83-792.77) | 36699 (21455-62637) | 673.33 (393.65-1149.24) | 1.33 (1.21-1.45) |

**TableS24: Trends in the burden of chronic kidney disease due to glomerulonephritis by sex: incidence, prevalence, deaths, and disability-adjusted life years (1990–2021).**

| Sex | 1990 | | 2021 | | **EAPC_95%CI** |
| --- | --- | --- | --- | --- | --- |
|  | Number_95%UI | ASR | Number_95%UI | ASR |  |
| **Incidence** |  |  |  |  |  |
| Global | 244229 (218811-271857) | 4.3 (3.88-4.75) | 357288 (329226-388483) | 4.84 (4.42-5.29) | 0.39 (0.36-0.43) |
| Female | 87235 (77100-99306) | 3.1 (2.75-3.5) | 120654 (109596-133046) | 3.38 (3.04-3.77) | 0.28 (0.25-0.31) |
| Male | 156994 (141222-173481) | 5.48 (4.98-6.01) | 236634 (218787-254836) | 6.28 (5.77-6.78) | 0.45 (0.42-0.49) |
| **Prevalence** |  |  |  |  |  |
| Global | 6370882 (5926723-6848154) | 128.55 (119.33-137.58) | 10735809 (9925500-11520171) | 129.94 (120.25-139.51) | 0.06 (0.04-0.08) |
| Female | 2644789 (2434847-2883624) | 106.92 (98.49-116.09) | 4531261 (4169272-4956553) | 108.5 (99.78-118.56) | 0.09 (0.07-0.11) |
| Male | 3726093 (3484081-3986630) | 150.9 (141.25-160.98) | 6204548 (5774782-6630226) | 151.85 (141.58-162.39) | 0.04 (0.02-0.06) |
| **Deaths** |  |  |  |  |  |
| Global | 83170 (69625-97738) | 2.02 (1.68-2.38) | 193997 (162332-226569) | 2.34 (1.96-2.74) | 0.54 (0.5-0.59) |
| Female | 37141 (31114-43462) | 1.67 (1.38-1.96) | 89022 (73524-105883) | 1.99 (1.66-2.36) | 0.6 (0.55-0.66) |
| Male | 46028 (37748-54972) | 2.5 (2.05-3.01) | 104975 (87280-123438) | 2.78 (2.3-3.26) | 0.43 (0.39-0.47) |
| **DALYs** |  |  |  |  |  |
| Global | 3751088 (3252292-4269460) | 77.78 (67.62-88.41) | 6959758 (6018414-7961673) | 84.47 (73.2-96.13) | 0.28 (0.25-0.31) |
| Female | 1655577 (1429752-1885529) | 67.19 (57.78-76.73) | 3068936 (2640502-3521006) | 72.47 (62.8-82.98) | 0.23 (0.19-0.27) |
| Male | 2095512 (1783982-2409288) | 90.23 (77.22-104.71) | 3890822 (3322946-4499291) | 97.57 (83.48-112.8) | 0.29 (0.27-0.31) |

| **TableS25: National Burden of chronic kidney disease due to glomerulonephritis: incidence Cases, ASIR, and EAPC (1990–2021).** | | | | | |
| --- | --- | --- | --- | --- | --- |
| Country | **1990** | | **2021** | | **EAPC_95%CI** |
|  | Number_95%UI | ASR | Number_95%UI | ASR |  |
| Afghanistan | 631 (235-1204) | 4.97 (2.36-8.7) | 2514 (898-4969) | 6.74 (3.25-11.88) | 0.95 (0.77-1.13) |
| Albania | 146 (51-283) | 4.01 (1.59-7.57) | 114 (63-188) | 5.17 (2.25-9.42) | 1.06 (0.87-1.25) |
| Algeria | 1394 (482-2789) | 4.86 (2.18-8.88) | 3090 (1428-5585) | 6.96 (3.27-12.51) | 1.07 (0.99-1.15) |
| American Samoa | 4 (1-7) | 6.22 (2.91-11.12) | 4 (2-7) | 8.51 (4.1-15.15) | 1 (0.96-1.04) |
| Andorra | 1 (1-2) | 2.45 (1.13-4.39) | 2 (1-3) | 2.24 (1.1-3.96) | -0.27 (-0.3--0.24) |
| Angola | 500 (141-1015) | 3.11 (1.17-5.72) | 1618 (489-3179) | 3.65 (1.47-6.65) | 0.48 (0.37-0.59) |
| Antigua and Barbuda | 3 (2-6) | 5.76 (2.86-10.46) | 7 (4-11) | 8.4 (4.2-14.89) | 1.3 (1.22-1.37) |
| Argentina | 1277 (561-2337) | 3.83 (1.7-6.98) | 1612 (815-2728) | 4.42 (1.93-7.99) | 0.65 (0.49-0.81) |
| Armenia | 138 (53-269) | 3.87 (1.52-7.45) | 134 (73-225) | 5.21 (2.39-9.42) | 1.05 (0.91-1.19) |
| Australia | 435 (242-712) | 2.82 (1.38-4.88) | 804 (505-1221) | 3.22 (1.64-5.57) | 0.43 (0.38-0.48) |
| Austria | 176 (100-268) | 2.56 (1.25-4.29) | 249 (163-370) | 2.76 (1.41-4.52) | 0.31 (0.29-0.33) |
| Azerbaijan | 421 (153-822) | 5.15 (2-9.84) | 682 (311-1230) | 7.5 (3.1-14.25) | 1.42 (1.28-1.55) |
| Bahamas | 13 (6-24) | 5.27 (2.48-9.26) | 26 (14-41) | 7.54 (3.64-13.2) | 1.31 (1.21-1.4) |
| Bahrain | 25 (10-48) | 4.87 (2.41-8.92) | 95 (57-155) | 7.2 (3.91-12.64) | 1.22 (1.13-1.3) |
| Bangladesh | 4257 (1483-8304) | 2.88 (1.3-5.12) | 5693 (2669-9859) | 3.75 (1.72-6.61) | 0.72 (0.66-0.79) |
| Barbados | 11 (6-20) | 5.02 (2.31-9.17) | 18 (11-28) | 7.07 (3.39-12.41) | 1.16 (1.08-1.23) |
| Belarus | 323 (140-611) | 3.63 (1.43-7.14) | 330 (184-535) | 4.58 (2.02-8.44) | 0.69 (0.46-0.91) |
| Belgium | 250 (145-393) | 2.8 (1.32-4.93) | 296 (187-442) | 2.67 (1.35-4.67) | -0.14 (-0.15--0.12) |
| Belize | 12 (4-24) | 5.45 (2.52-9.61) | 35 (16-61) | 8.32 (4.04-14.5) | 1.41 (1.34-1.49) |
| Benin | 343 (89-706) | 4.49 (1.68-8.62) | 929 (281-1849) | 5.11 (2.03-9.58) | 0.38 (0.34-0.41) |
| Bermuda | 2 (1-4) | 4.39 (1.99-7.54) | 4 (2-5) | 6.37 (3.21-10.69) | 1.35 (1.25-1.45) |
| Bhutan | 33 (12-66) | 4.13 (1.87-7.64) | 37 (19-64) | 5.19 (2.55-9.3) | 0.82 (0.78-0.86) |
| Bolivia (Plurinational State of) | 362 (119-733) | 4.5 (1.97-8.35) | 642 (279-1140) | 5.51 (2.48-9.64) | 0.76 (0.72-0.79) |
| Bosnia and Herzegovina | 127 (61-223) | 3.12 (1.37-5.66) | 122 (78-183) | 4.07 (2.06-6.97) | 0.97 (0.89-1.06) |
| Botswana | 62 (23-112) | 3.88 (1.83-6.42) | 111 (53-189) | 4.69 (2.31-7.94) | 0.48 (0.31-0.65) |
| Brazil | 6985 (5431-8903) | 4.72 (3.75-5.87) | 10483 (8742-12382) | 5.11 (4.15-6.21) | 0.14 (0.08-0.19) |
| Brunei Darussalam | 13 (5-23) | 4.69 (2.22-7.94) | 21 (11-35) | 5.54 (2.6-9.5) | 0.79 (0.57-1.01) |
| Bulgaria | 267 (144-464) | 3.65 (1.63-6.79) | 309 (189-483) | 5.5 (2.7-10.14) | 1.43 (1.37-1.48) |
| Burkina Faso | 645 (183-1342) | 4.18 (1.66-8.13) | 1713 (510-3535) | 5.1 (2.01-9.85) | 0.76 (0.7-0.83) |
| Burundi | 254 (83-531) | 3.02 (1.25-5.73) | 520 (179-1082) | 3 (1.28-5.79) | -0.07 (-0.17-0.02) |
| C?te d'Ivoire | 20 (5-43) | 4.11 (1.39-8.17) | 26 (11-48) | 5.13 (2.05-9.74) | 0.7 (0.61-0.8) |
| Cabo Verde | 579 (183-1159) | 3.97 (1.56-7.43) | 794 (339-1413) | 4.62 (2.02-8.11) | 0.43 (0.34-0.53) |
| Cambodia | 888 (264-1901) | 5.73 (2.23-11.36) | 2697 (925-5607) | 6.82 (2.96-13.26) | 0.49 (0.4-0.58) |
| Cameroon | 875 (460-1504) | 3.67 (1.64-6.56) | 1237 (725-1934) | 3.91 (1.68-6.98) | 0.51 (0.34-0.69) |
| Canada | 124 (36-255) | 3.06 (1.24-5.8) | 255 (85-502) | 3.65 (1.55-6.67) | 0.56 (0.53-0.6) |
| Central African Republic | 399 (118-820) | 4.09 (1.65-7.68) | 1280 (393-2607) | 4.73 (2.01-8.66) | 0.45 (0.35-0.55) |
| Chad | 497 (181-980) | 3.68 (1.48-7.11) | 728 (393-1221) | 4.71 (1.98-8.76) | 0.92 (0.8-1.05) |
| Chile | 44054 (35811-53621) | 3.91 (3.18-4.76) | 37893 (33122-43564) | 3.31 (2.71-3.95) | -0.44 (-0.52--0.36) |
| China | 2014 (821-3842) | 5.65 (2.76-9.95) | 2960 (1615-4919) | 6.78 (3.25-11.91) | 0.66 (0.62-0.69) |
| Colombia | 25 (7-50) | 3.6 (1.32-6.89) | 30 (10-57) | 3.79 (1.46-7.12) | 0.04 (-0.02-0.09) |
| Comoros | 111 (35-219) | 3.36 (1.34-6.23) | 224 (87-419) | 3.84 (1.68-6.93) | 0.36 (0.27-0.45) |
| Congo | 1 (0-2) | 4.64 (2.19-8.49) | 1 (1-2) | 6.75 (3.46-11.93) | 1.23 (1.17-1.29) |
| Cook Islands | 256 (109-479) | 8.2 (4.17-14.03) | 402 (229-648) | 9.32 (4.69-15.83) | 0.4 (0.38-0.42) |
| Costa Rica | 134 (74-224) | 3.17 (1.46-5.87) | 155 (101-224) | 3.86 (2-6.61) | 0.71 (0.67-0.76) |
| Croatia | 427 (195-779) | 4.27 (1.88-7.84) | 599 (356-904) | 6.08 (2.93-10.73) | 1.24 (1.18-1.29) |
| Cuba | 21 (11-34) | 2.82 (1.4-4.84) | 38 (24-55) | 2.81 (1.49-4.64) | -0.01 (-0.07-0.05) |
| Cyprus | 276 (137-475) | 3.18 (1.36-5.84) | 358 (215-552) | 3.6 (1.74-6.4) | 0.33 (0.23-0.42) |
| Czechia | 907 (305-1887) | 5.05 (2.13-9.71) | 2043 (771-4158) | 5.82 (2.56-11.01) | 0.39 (0.33-0.45) |
| Democratic People's Republic of Korea | 896 (326-1709) | 3.98 (1.56-7.45) | 985 (523-1681) | 4.48 (1.87-8.29) | 0.37 (0.35-0.38) |
| Democratic Republic of the Congo | 1802 (522-3850) | 2.99 (1.15-5.81) | 3785 (1274-7704) | 3.38 (1.42-6.46) | 0.32 (0.23-0.42) |
| Denmark | 120 (68-195) | 2.65 (1.23-4.89) | 162 (98-248) | 2.88 (1.38-5.29) | 0.4 (0.35-0.44) |
| Djibouti | 19 (6-37) | 3.28 (1.33-6.11) | 51 (20-98) | 3.83 (1.62-7.05) | 0.39 (0.28-0.5) |
| Dominica | 5 (2-9) | 6.4 (2.86-11.49) | 5 (3-9) | 9.44 (4.21-17.4) | 1.33 (1.24-1.41) |
| Dominican Republic | 352 (126-710) | 4.18 (1.92-7.87) | 671 (331-1197) | 6.21 (3.03-11.14) | 1.41 (1.35-1.47) |
| Ecuador | 493 (177-967) | 4.32 (1.91-7.91) | 1126 (547-1949) | 6.52 (3.15-11.3) | 1.54 (1.48-1.61) |
| Egypt | 3062 (1085-5907) | 4.72 (2.15-8.4) | 7784 (3410-14008) | 7.15 (3.49-12.27) | 1.27 (1.17-1.36) |
| El Salvador | 348 (143-668) | 5.85 (2.84-10.34) | 702 (369-1206) | 11.14 (5.81-19.03) | 2.47 (2.33-2.62) |
| Equatorial Guinea | 20 (5-43) | 3.02 (1.09-5.84) | 70 (23-143) | 4.17 (1.7-8) | 1.26 (1.15-1.36) |
| Eritrea | 155 (46-317) | 3.12 (1.2-5.87) | 268 (94-525) | 3.43 (1.43-6.36) | 0.15 (0.04-0.26) |
| Estonia | 53 (25-104) | 3.92 (1.64-8) | 57 (34-95) | 5.23 (2.52-10.01) | 0.96 (0.88-1.05) |
| Eswatini | 50 (16-99) | 4.77 (2.09-8.56) | 77 (32-145) | 6.26 (2.84-11.01) | 0.74 (0.57-0.92) |
| Ethiopia | 2520 (1514-3842) | 3.17 (2.09-4.55) | 4255 (2521-6432) | 3.13 (2.04-4.52) | -0.17 (-0.28--0.06) |
| Fiji | 48 (19-91) | 5.79 (2.68-10.58) | 66 (32-118) | 7.17 (3.47-12.92) | 0.62 (0.58-0.67) |
| Finland | 83 (46-137) | 1.87 (0.87-3.49) | 107 (66-159) | 2 (0.96-3.63) | 0.13 (0.07-0.19) |
| France | 1295 (689-2237) | 2.51 (1.14-4.63) | 1605 (995-2483) | 2.45 (1.22-4.47) | -0.1 (-0.13--0.07) |
| Gabon | 47 (15-97) | 3.59 (1.39-6.92) | 89 (35-174) | 4.53 (2-8.47) | 0.63 (0.52-0.74) |
| Gambia | 63 (19-129) | 4.25 (1.62-8.21) | 147 (51-293) | 4.97 (2.03-9.31) | 0.42 (0.32-0.53) |
| Georgia | 223 (103-422) | 4.39 (1.89-8.6) | 179 (96-317) | 5.85 (2.8-11.06) | 1.05 (0.93-1.17) |
| Germany | 2055 (1192-3299) | 2.89 (1.35-5.19) | 2501 (1614-3700) | 2.83 (1.42-4.94) | -0.09 (-0.11--0.07) |
| Ghana | 748 (220-1510) | 3.58 (1.39-6.68) | 1862 (657-3677) | 4.67 (1.89-8.67) | 0.85 (0.78-0.93) |
| Greece | 289 (178-448) | 3.09 (1.6-5.47) | 297 (195-436) | 2.88 (1.51-5.07) | -0.13 (-0.21--0.05) |
| Greenland | 2 (1-3) | 3.15 (1.27-5.69) | 2 (1-3) | 3.25 (1.44-5.64) | 0.15 (0.05-0.25) |
| Grenada | 5 (2-10) | 5.66 (2.79-10.34) | 8 (5-14) | 8.95 (4.59-15.72) | 1.48 (1.4-1.56) |
| Guam | 8 (3-16) | 5.16 (2.35-9.88) | 12 (6-21) | 7.99 (3.63-14.99) | 1.52 (1.44-1.6) |
| Guatemala | 632 (214-1264) | 5.94 (2.84-10.66) | 1368 (650-2486) | 8.92 (4.49-15.82) | 1.43 (1.36-1.5) |
| Guinea | 399 (117-870) | 4.33 (1.68-8.62) | 891 (299-1851) | 5.01 (2.06-9.61) | 0.42 (0.37-0.47) |
| Guinea-Bissau | 72 (21-142) | 4.89 (1.89-8.92) | 139 (47-266) | 5.31 (2.2-9.5) | 0.2 (0.15-0.25) |
| Guyana | 48 (17-91) | 5.14 (2.34-9.16) | 57 (28-100) | 7.56 (3.72-13.12) | 1.25 (1.21-1.29) |
| Haiti | 382 (138-762) | 4.72 (2.14-8.65) | 755 (329-1414) | 5.46 (2.61-9.77) | 0.49 (0.45-0.53) |
| Honduras | 336 (125-687) | 5.83 (2.92-10.45) | 678 (327-1239) | 6.74 (3.49-11.84) | 0.51 (0.49-0.52) |
| Hungary | 256 (133-435) | 3.03 (1.3-5.56) | 339 (205-517) | 4.01 (2-7.01) | 0.92 (0.87-0.97) |
| Iceland | 5 (3-9) | 2.29 (1.02-4.03) | 7 (4-11) | 2.17 (1.04-3.69) | -0.1 (-0.12--0.07) |
| India | 42127 (34911-50698) | 4.4 (3.74-5.17) | 55734 (47520-64606) | 4.21 (3.57-4.95) | -0.18 (-0.23--0.12) |
| Indonesia | 10001 (7597-12713) | 4.82 (3.73-6.05) | 14693 (11853-17885) | 5.75 (4.56-7.07) | 0.4 (0.31-0.49) |
| Iran (Islamic Republic of) | 4173 (3245-5150) | 6.56 (5.41-7.81) | 5916 (5078-6888) | 7.65 (6.47-9.08) | 0.31 (0.22-0.4) |
| Iraq | 1307 (473-2682) | 5.86 (2.86-10.92) | 3194 (1535-5876) | 8.01 (4.13-14.32) | 0.98 (0.85-1.12) |
| Ireland | 123 (64-214) | 3.71 (1.81-6.58) | 160 (97-246) | 3.5 (1.69-5.94) | -0.2 (-0.21--0.18) |
| Israel | 159 (78-284) | 3.2 (1.63-5.66) | 331 (183-540) | 3.35 (1.77-5.6) | 0.24 (0.12-0.35) |
| Italy | 1362 (1136-1650) | 2.96 (2.33-3.75) | 1408 (1212-1624) | 2.73 (2.16-3.46) | -0.15 (-0.24--0.05) |
| Jamaica | 127 (54-248) | 5.19 (2.53-9.58) | 176 (103-296) | 6.99 (3.71-12.19) | 1.07 (0.97-1.18) |
| Japan | 4621 (4048-5187) | 4.04 (3.4-4.69) | 4872 (4385-5368) | 3.77 (3.18-4.39) | -0.29 (-0.32--0.25) |
| Jordan | 222 (77-459) | 5.15 (2.47-9.42) | 824 (415-1431) | 7.27 (3.77-12.51) | 1.14 (1.08-1.2) |
| Kazakhstan | 889 (352-1795) | 5.08 (2.1-10.07) | 1134 (552-2084) | 6 (2.88-11.11) | 0.54 (0.38-0.7) |
| Kenya | 969 (787-1163) | 2.81 (2.37-3.27) | 1712 (1439-2023) | 3.12 (2.65-3.61) | 0.07 (-0.09-0.23) |
| Kiribati | 6 (2-11) | 5.84 (2.56-11.13) | 10 (4-18) | 7.29 (3.44-13.42) | 0.71 (0.68-0.74) |
| Kuwait | 90 (40-164) | 5.51 (2.81-9.54) | 258 (148-410) | 6.44 (3.3-11.16) | 0.54 (0.45-0.63) |
| Kyrgyzstan | 339 (119-669) | 6.4 (2.45-12.29) | 479 (192-896) | 6.6 (2.74-12.07) | 0.03 (-0.05-0.12) |
| Lao People's Democratic Republic | 288 (92-604) | 5.17 (2.18-10.1) | 473 (196-910) | 6.1 (2.72-11.43) | 0.51 (0.44-0.57) |
| Latvia | 81 (34-149) | 3.61 (1.32-6.89) | 70 (39-112) | 4.81 (2.05-8.6) | 0.84 (0.69-0.99) |
| Lebanon | 171 (67-315) | 5.08 (2.21-8.81) | 358 (188-577) | 7.21 (3.46-12.06) | 1.1 (1.04-1.16) |
| Lesotho | 69 (26-138) | 3.7 (1.68-6.74) | 98 (45-182) | 5.09 (2.55-9.16) | 0.89 (0.72-1.06) |
| Liberia | 156 (49-309) | 4.21 (1.71-7.8) | 324 (121-628) | 4.96 (2.23-8.96) | 0.68 (0.63-0.74) |
| Libya | 230 (89-486) | 4.93 (2.48-9.09) | 423 (246-728) | 6.87 (3.68-12.32) | 0.89 (0.77-1.01) |
| Lithuania | 125 (54-227) | 3.87 (1.53-7.33) | 93 (53-148) | 4.49 (2.02-8.12) | 0.43 (0.28-0.59) |
| Luxembourg | 9 (5-15) | 2.71 (1.21-4.72) | 16 (10-25) | 2.65 (1.29-4.52) | 0.03 (-0.03-0.09) |
| Madagascar | 568 (172-1168) | 3.15 (1.17-6.06) | 1090 (380-2181) | 3.13 (1.28-5.88) | -0.13 (-0.2--0.05) |
| Malawi | 462 (126-975) | 2.95 (1.12-5.77) | 798 (266-1608) | 3.35 (1.35-6.26) | 0.38 (0.27-0.49) |
| Malaysia | 785 (287-1530) | 3.99 (1.69-7.25) | 1497 (723-2621) | 5.17 (2.37-9.33) | 0.78 (0.71-0.84) |
| Maldives | 14 (5-28) | 4.71 (2.11-8.59) | 24 (13-41) | 5.8 (2.8-10.35) | 0.64 (0.6-0.69) |
| Mali | 596 (167-1225) | 4.25 (1.65-8.31) | 1722 (517-3443) | 4.8 (1.93-8.74) | 0.39 (0.35-0.44) |
| Malta | 10 (5-17) | 2.94 (1.44-5.3) | 14 (9-20) | 3.23 (1.58-5.61) | 0.39 (0.3-0.48) |
| Marshall Islands | 3 (1-6) | 5.42 (2.35-10.06) | 4 (2-7) | 6.93 (3.23-12.51) | 0.7 (0.63-0.77) |
| Mauritania | 140 (45-285) | 4.77 (1.87-9.25) | 302 (103-604) | 5.55 (2.24-10.49) | 0.44 (0.36-0.51) |
| Mauritius | 66 (31-117) | 6.08 (2.99-10.65) | 112 (69-173) | 9.97 (5.08-17.26) | 1.71 (1.66-1.76) |
| Mexico | 6321 (4979-7942) | 6.72 (5.52-8.12) | 12894 (10993-15050) | 10.52 (8.82-12.41) | 1.6 (1.51-1.7) |
| Micronesia (Federated States of) | 7 (3-14) | 6.1 (2.86-10.73) | 9 (4-15) | 8.49 (4.11-14.33) | 1.09 (1.05-1.13) |
| Monaco | 1 (0-1) | 2.52 (1.19-4.51) | 1 (1-1) | 2.47 (1.23-4.32) | -0.02 (-0.04-0) |
| Mongolia | 162 (59-326) | 5.98 (2.56-11.34) | 230 (101-417) | 6.69 (3.01-12.04) | 0.25 (0.2-0.29) |
| Montenegro | 27 (13-46) | 4.67 (2.18-8.12) | 29 (18-45) | 5.08 (2.6-8.53) | 0.3 (0.23-0.36) |
| Morocco | 1245 (462-2475) | 4.26 (1.96-7.86) | 2493 (1267-4388) | 7.05 (3.43-12.75) | 1.59 (1.44-1.75) |
| Mozambique | 624 (178-1326) | 3.04 (1.21-5.9) | 1561 (484-3163) | 3.69 (1.55-6.99) | 0.59 (0.53-0.66) |
| Myanmar | 2394 (860-4671) | 5.18 (2.04-9.61) | 3646 (1498-6583) | 6.6 (2.67-12.04) | 0.77 (0.68-0.86) |
| Namibia | 62 (21-121) | 3.66 (1.65-6.53) | 101 (43-186) | 4.09 (1.91-7.24) | 0.25 (0.07-0.43) |
| Nauru | 1 (0-1) | 5.78 (2.62-10.48) | 1 (0-2) | 7.63 (3.53-13.82) | 0.83 (0.74-0.91) |
| Nepal | 1008 (351-2029) | 3.94 (1.76-7.31) | 1799 (834-3330) | 5.73 (2.77-10.35) | 0.99 (0.92-1.06) |
| Netherlands | 311 (176-504) | 2.4 (1.12-4.31) | 439 (288-646) | 2.62 (1.27-4.59) | 0.49 (0.39-0.6) |
| New Zealand | 108 (64-170) | 3.35 (1.88-5.47) | 198 (128-284) | 4.28 (2.37-6.61) | 0.91 (0.81-1.01) |
| Nicaragua | 389 (143-757) | 8.46 (4.15-14.77) | 880 (455-1468) | 13.17 (6.85-21.8) | 1.41 (1.32-1.5) |
| Niger | 594 (171-1251) | 4.37 (1.66-8.43) | 1650 (513-3379) | 4.4 (1.88-8.32) | 0.03 (0-0.06) |
| Nigeria | 6132 (4980-7377) | 4.46 (3.7-5.27) | 14796 (11903-17958) | 4.84 (4-5.7) | 0.25 (0.16-0.34) |
| Niue | 0 (0-0) | 5.45 (2.44-10.36) | 0 (0-0) | 7.6 (3.49-14.33) | 1.08 (1.04-1.12) |
| North Macedonia | 67 (31-123) | 3.61 (1.58-6.78) | 94 (58-141) | 4.77 (2.46-8.52) | 0.98 (0.83-1.13) |
| Northern Mariana Islands | 3 (1-5) | 6.23 (3.03-10.65) | 4 (2-6) | 8.16 (4.07-13.74) | 0.77 (0.68-0.85) |
| Norway | 82 (67-100) | 2.06 (1.59-2.65) | 124 (102-150) | 2.63 (1.98-3.41) | 1.07 (0.96-1.19) |
| Oman | 87 (31-173) | 3.76 (1.88-6.55) | 241 (124-400) | 5.77 (3.08-9.53) | 1.25 (0.99-1.52) |
| Pakistan | 6174 (3468-9779) | 4.38 (2.76-6.54) | 14572 (8850-22135) | 5.56 (3.58-8.19) | 0.78 (0.74-0.82) |
| Palau | 1 (0-2) | 6.66 (3.1-12.02) | 2 (1-3) | 10.77 (5.34-18.58) | 1.62 (1.49-1.75) |
| Palestine | 164 (52-336) | 5.97 (2.71-10.69) | 393 (166-728) | 7.67 (3.64-13.38) | 0.66 (0.54-0.78) |
| Panama | 152 (66-279) | 6.14 (2.99-10.67) | 352 (185-570) | 8.68 (4.36-14.4) | 1.16 (1.12-1.2) |
| Papua New Guinea | 222 (74-442) | 4.09 (1.63-7.65) | 618 (219-1162) | 4.79 (2.03-8.42) | 0.45 (0.36-0.54) |
| Paraguay | 196 (72-385) | 4.22 (2-7.57) | 418 (205-741) | 6.19 (3.03-10.97) | 1.26 (1.2-1.33) |
| Peru | 904 (299-1800) | 3.58 (1.51-6.55) | 1862 (878-3359) | 5.4 (2.46-9.82) | 1.51 (1.44-1.58) |
| Philippines | 3700 (3130-4366) | 4.9 (4.24-5.65) | 7811 (6781-8881) | 6.86 (5.99-7.74) | 1.01 (0.98-1.05) |
| Poland | 1560 (1222-1906) | 4.82 (3.67-5.95) | 1244 (1047-1457) | 4.09 (3.19-5.05) | -0.7 (-0.78--0.63) |
| Portugal | 220 (118-352) | 2.51 (1.12-4.37) | 262 (166-383) | 2.49 (1.17-4.2) | 0.06 (0-0.13) |
| Puerto Rico | 181 (92-315) | 5.3 (2.64-9.33) | 203 (134-298) | 7.89 (3.98-13.7) | 1.42 (1.29-1.54) |
| Qatar | 24 (9-45) | 5.45 (2.56-9.51) | 187 (99-312) | 7.65 (3.78-13.07) | 0.97 (0.84-1.1) |
| Republic of Korea | 1283 (628-2229) | 3.37 (1.63-5.87) | 1389 (961-1980) | 3 (1.53-5.05) | -0.28 (-0.38--0.17) |
| Republic of Moldova | 124 (51-241) | 2.91 (1.15-5.69) | 111 (66-184) | 4.07 (1.83-7.63) | 1 (0.85-1.15) |
| Romania | 678 (341-1228) | 3.31 (1.49-6.37) | 673 (420-1033) | 3.96 (2.04-6.99) | 0.54 (0.45-0.63) |
| Russian Federation | 8448 (7273-9824) | 6.79 (5.83-7.94) | 7938 (7003-9035) | 7.09 (6.04-8.34) | 0.06 (-0.01-0.14) |
| Rwanda | 331 (94-668) | 3.11 (1.2-5.82) | 474 (157-917) | 3.08 (1.21-5.65) | -0.06 (-0.15-0.03) |
| Saint Kitts and Nevis | 2 (1-4) | 5.49 (2.73-9.22) | 4 (3-7) | 8.06 (4.1-13.74) | 1.37 (1.24-1.51) |
| Saint Lucia | 8 (3-15) | 5.28 (2.51-9.5) | 12 (7-19) | 7.77 (3.81-13.8) | 1.27 (1.17-1.37) |
| Saint Vincent and the Grenadines | 6 (2-11) | 5.11 (2.44-9.47) | 8 (4-13) | 7.59 (3.82-13.81) | 1.34 (1.26-1.42) |
| Samoa | 11 (4-22) | 5.52 (2.34-10.01) | 17 (7-32) | 7.03 (3.25-12.84) | 0.73 (0.7-0.76) |
| San Marino | 0 (0-1) | 2.37 (1.07-4.2) | 1 (0-1) | 2.3 (1.09-3.98) | -0.07 (-0.09--0.06) |
| Sao Tome and Principe | 10 (3-20) | 5.72 (2.18-11.1) | 18 (7-35) | 7.47 (3.07-13.95) | 0.91 (0.85-0.97) |
| Saudi Arabia | 965 (374-1899) | 5.6 (2.84-9.83) | 3053 (1730-4976) | 8.89 (4.76-15.37) | 1.41 (1.27-1.55) |
| Senegal | 549 (156-1095) | 4.73 (1.71-8.82) | 995 (331-1914) | 5.13 (2.01-9.32) | 0.2 (0.14-0.25) |
| Serbia | 256 (142-434) | 2.86 (1.38-5.22) | 301 (198-459) | 3.41 (1.88-5.78) | 0.67 (0.63-0.7) |
| Seychelles | 3 (2-7) | 4.77 (2.3-8.59) | 7 (4-11) | 6.55 (3.15-11.61) | 1 (0.96-1.04) |
| Sierra Leone | 290 (79-596) | 4.47 (1.68-8.59) | 594 (186-1194) | 5.17 (2.04-9.8) | 0.48 (0.39-0.57) |
| Singapore | 110 (56-190) | 4.26 (2.07-7.55) | 223 (141-335) | 4.24 (2.14-7.44) | 0.2 (0.11-0.29) |
| Slovakia | 178 (84-305) | 3.81 (1.58-6.85) | 211 (128-331) | 4.4 (2.01-7.7) | 0.42 (0.37-0.48) |
| Slovenia | 46 (24-78) | 2.86 (1.26-5.22) | 66 (42-101) | 3.47 (1.72-5.98) | 0.6 (0.53-0.66) |
| Solomon Islands | 28 (11-57) | 6.59 (2.88-12.24) | 49 (21-96) | 6.32 (2.86-11.57) | -0.33 (-0.44--0.22) |
| Somalia | 390 (111-768) | 3.17 (1.28-5.81) | 1104 (320-2156) | 3.43 (1.4-6.2) | 0.24 (0.2-0.29) |
| South Africa | 1944 (1343-2683) | 4.66 (3.32-6.18) | 2762 (2085-3526) | 5.13 (3.86-6.63) | 0.18 (0.04-0.33) |
| South Sudan | 259 (69-533) | 3.02 (1.13-5.68) | 483 (135-951) | 3.52 (1.32-6.47) | 0.48 (0.41-0.56) |
| Spain | 880 (504-1379) | 2.61 (1.25-4.64) | 948 (625-1336) | 2.03 (1.04-3.51) | -0.6 (-0.68--0.51) |
| Sri Lanka | 772 (339-1480) | 4.52 (2.15-8.34) | 1167 (653-1961) | 5.65 (2.82-10.07) | 0.73 (0.69-0.76) |
| Sudan | 1015 (334-2037) | 4.01 (1.91-7.15) | 2488 (966-4787) | 5.61 (2.71-9.67) | 1.02 (0.8-1.25) |
| Suriname | 23 (9-43) | 5.66 (2.55-10.28) | 45 (23-77) | 8.36 (3.95-15.14) | 1.41 (1.34-1.48) |
| Sweden | 167 (106-253) | 2 (1.11-3.38) | 184 (125-252) | 1.68 (1.05-2.63) | -0.16 (-0.31--0.01) |
| Switzerland | 164 (95-257) | 2.7 (1.3-4.66) | 226 (142-337) | 2.58 (1.31-4.37) | -0.13 (-0.15--0.12) |
| Syrian Arab Republic | 952 (330-2004) | 5.97 (2.74-11.26) | 925 (507-1619) | 6.84 (3.55-12.66) | 0.41 (0.33-0.5) |
| Taiwan (Province of China) | 755 (404-1291) | 4.16 (2.19-7.24) | 1066 (669-1616) | 5.24 (2.61-9.4) | 0.73 (0.63-0.83) |
| Tajikistan | 270 (83-556) | 3.75 (1.43-7.31) | 497 (187-964) | 4.39 (1.79-8.25) | 0.63 (0.46-0.8) |
| Thailand | 3449 (1399-6586) | 6.24 (2.61-11.62) | 3570 (2278-5378) | 6.17 (3.07-10.73) | -0.06 (-0.14-0.02) |
| Timor-Leste | 46 (14-98) | 4.2 (1.73-7.97) | 78 (28-153) | 4.92 (2.12-9.09) | 0.56 (0.48-0.64) |
| Togo | 240 (70-491) | 4.43 (1.72-8.5) | 486 (176-957) | 4.92 (2.09-9.06) | 0.25 (0.15-0.35) |
| Tokelau | 0 (0-0) | 4.61 (1.98-8.84) | 0 (0-0) | 6.5 (3.08-12.13) | 1.06 (0.98-1.14) |
| Tonga | 6 (2-13) | 5.16 (2.31-9.62) | 8 (3-16) | 7.01 (3.29-12.53) | 0.94 (0.9-0.98) |
| Trinidad and Tobago | 61 (26-111) | 5.16 (2.32-9.02) | 95 (55-144) | 7.73 (3.74-12.87) | 1.45 (1.37-1.53) |
| Tunisia | 405 (152-769) | 4.58 (2.05-8.3) | 703 (386-1176) | 6.3 (3.05-11.14) | 0.93 (0.81-1.06) |
| Türkiye | 271 (94-548) | 5.78 (2.35-11.01) | 429 (184-781) | 8.09 (3.48-14.71) | 1.25 (1.17-1.33) |
| Turkmenistan | 1 (0-1) | 4.81 (1.97-8.95) | 1 (0-1) | 6.58 (3.07-11.52) | 0.98 (0.96-1) |
| Tuvalu | 2592 (1031-4887) | 4.29 (1.98-7.68) | 4690 (2808-7258) | 6.14 (3.29-10.36) | 1.18 (1.15-1.21) |
| Uganda | 704 (197-1507) | 2.44 (0.99-4.65) | 1584 (492-3289) | 2.71 (1.15-5.17) | 0.29 (0.14-0.43) |
| Ukraine | 1446 (692-2612) | 3.35 (1.43-6.35) | 1365 (811-2206) | 4.48 (2.16-8.04) | 1.04 (0.98-1.1) |
| United Arab Emirates | 113 (49-210) | 6.11 (3.18-10.5) | 829 (511-1205) | 8.86 (4.86-15.04) | 1.01 (0.87-1.15) |
| United Kingdom | 1549 (1359-1767) | 3.07 (2.62-3.6) | 1843 (1649-2042) | 3.18 (2.74-3.68) | 0.02 (-0.03-0.06) |
| United Republic of Tanzania | 1267 (369-2674) | 3.12 (1.16-6.08) | 2545 (796-5212) | 3.4 (1.32-6.53) | 0.19 (0.03-0.36) |
| United States of America | 12604 (10355-14944) | 5.57 (4.49-6.78) | 15629 (13600-17662) | 5.63 (4.66-6.64) | -0.06 (-0.14-0.03) |
| United States Virgin Islands | 6 (3-11) | 5.41 (2.37-9.84) | 6 (3-8) | 7.53 (3.65-13.38) | 1.11 (1.04-1.18) |
| Uruguay | 105 (46-191) | 3.52 (1.41-6.64) | 109 (58-180) | 4.07 (1.63-7.54) | 0.47 (0.38-0.56) |
| Uzbekistan | 1620 (651-3285) | 6.3 (2.89-12.26) | 2711 (1288-5180) | 7.86 (3.77-14.94) | 0.76 (0.67-0.86) |
| Vanuatu | 9 (3-18) | 4.54 (1.96-8.31) | 21 (8-39) | 5.99 (2.72-10.53) | 0.8 (0.73-0.87) |
| Venezuela (Bolivarian Republic of) | 1127 (498-2073) | 5.61 (2.94-9.48) | 1969 (1128-3164) | 7.39 (3.94-12.48) | 1.04 (0.94-1.14) |
| Viet Nam | 2781 (1002-5750) | 3.49 (1.46-6.74) | 4274 (2138-7541) | 4.63 (2.12-8.62) | 1.01 (0.99-1.03) |
| Yemen | 776 (229-1595) | 4.06 (1.81-7.19) | 1879 (680-3628) | 5.2 (2.39-9.13) | 0.78 (0.6-0.97) |
| Zambia | 421 (121-930) | 3.43 (1.42-6.99) | 907 (311-1848) | 3.76 (1.61-7.26) | 0.23 (0.07-0.38) |
| Zimbabwe | 480 (160-974) | 3.75 (1.76-6.82) | 733 (274-1411) | 4.21 (2.02-7.45) | 0.07 (-0.11-0.26) |

| **TableS26: National Burden of chronic kidney disease due to glomerulonephritis: prevalence cases, ASPR, and EAPC (1990–2021).** | | | | | |
| --- | --- | --- | --- | --- | --- |
| Country | **1990** | | **2021** | | **EAPC_95%CI** |
|  | Number_95%UI | ASR | Number_95%UI | ASR |  |
| Afghanistan | 10906 (7535-15704) | 136.41 (97.61-197.63) | 32215 (21581-48017) | 135.74 (97.12-196.26) | -0.07 (-0.1--0.03) |
| Albania | 3377 (2334-4821) | 109.85 (79.63-152.48) | 3139 (2365-4279) | 101.32 (73.42-141.65) | -0.3 (-0.33--0.27) |
| Algeria | 28397 (18655-42463) | 136.11 (95.45-194.69) | 57654 (40173-84433) | 131.37 (92.18-191.41) | -0.21 (-0.26--0.17) |
| American Samoa | 63 (43-94) | 159.18 (114.52-226.02) | 92 (67-127) | 183.7 (132.83-256.96) | 0.47 (0.45-0.49) |
| Andorra | 59 (44-80) | 96.29 (72.08-128.81) | 107 (81-139) | 92.12 (68.91-127.47) | -0.21 (-0.24--0.18) |
| Angola | 10895 (7794-15117) | 145.59 (109.02-193.8) | 33406 (23427-47746) | 142.88 (104.38-196.38) | -0.09 (-0.1--0.07) |
| Antigua and Barbuda | 72 (51-102) | 123.55 (89.29-171.05) | 131 (96-178) | 132.41 (96.45-180.37) | 0.25 (0.23-0.27) |
| Argentina | 30919 (22052-44318) | 95.49 (68.17-137.04) | 46135 (32282-68511) | 93.39 (64.71-139.5) | 0.12 (0.05-0.18) |
| Armenia | 5524 (3913-7906) | 167.18 (120.3-232.32) | 5719 (4187-7852) | 165.21 (118.35-231.32) | -0.09 (-0.11--0.06) |
| Australia | 15720 (11896-22498) | 85.78 (65.16-122.72) | 28232 (22191-37943) | 89.13 (68.41-124.18) | 0.12 (0.03-0.21) |
| Austria | 8644 (6632-11245) | 93.92 (71.7-123.92) | 11281 (8771-14431) | 95.28 (72.7-128.22) | 0.1 (0.08-0.12) |
| Azerbaijan | 12378 (8725-17284) | 182.6 (130.47-247.45) | 21566 (15601-28992) | 189.27 (136.94-256.62) | 0.12 (0.11-0.13) |
| Bahamas | 280 (195-389) | 116.26 (85.67-158.89) | 520 (385-711) | 123.31 (90.54-170.29) | 0.2 (0.18-0.21) |
| Bahrain | 672 (452-1111) | 139.06 (99.27-207.16) | 2410 (1693-3743) | 140.7 (99.74-214.17) | 0 (-0.03-0.04) |
| Bangladesh | 118071 (79318-173341) | 136.55 (96.81-191.61) | 226633 (157003-330583) | 137.69 (97.38-197.67) | 0.02 (-0.03-0.06) |
| Barbados | 296 (216-410) | 112.48 (81.66-156.34) | 417 (317-559) | 119 (87.94-165.41) | 0.19 (0.18-0.21) |
| Belarus | 15520 (10830-22666) | 136.53 (95-199.86) | 15778 (11151-23013) | 138.05 (96.48-202.86) | 0.05 (0.04-0.07) |
| Belgium | 11584 (8923-15225) | 97.88 (73.98-130.83) | 14171 (10945-18158) | 96.21 (72.1-130.78) | -0.02 (-0.05-0) |
| Belize | 182 (124-258) | 121.55 (88.28-165.96) | 565 (406-789) | 134.88 (99.87-184.51) | 0.37 (0.36-0.39) |
| Benin | 4262 (2913-6404) | 122.63 (87.67-170.5) | 12824 (8869-19137) | 126.85 (91.44-177.15) | 0.1 (0.09-0.11) |
| Bermuda | 69 (50-98) | 105.53 (76.67-148.12) | 81 (60-113) | 102.87 (75.58-145.4) | -0.1 (-0.15--0.06) |
| Bhutan | 750 (485-1102) | 147.41 (104.01-202.23) | 1179 (820-1647) | 150.02 (106.96-206.92) | 0.06 (0.05-0.07) |
| Bolivia (Plurinational State of) | 5833 (4091-8027) | 110.37 (82.52-145.99) | 12221 (8961-16615) | 107.02 (80.04-144.18) | -0.08 (-0.1--0.06) |
| Bosnia and Herzegovina | 4601 (3416-6435) | 98.42 (74.05-136.35) | 4030 (3127-5305) | 98.74 (74.4-135.73) | 0.06 (0.04-0.09) |
| Botswana | 1282 (885-1833) | 137.28 (99.73-182.51) | 3291 (2360-4581) | 144.15 (104.83-193.8) | 0.18 (0.17-0.19) |
| Brazil | 173029 (151443-197916) | 132.72 (116.75-150.88) | 300153 (265054-340456) | 123.33 (109.27-140) | -0.25 (-0.28--0.22) |
| Brunei Darussalam | 325 (217-480) | 142.31 (101.8-202.34) | 715 (511-1025) | 145.73 (104.51-207.21) | 0.01 (-0.01-0.03) |
| Bulgaria | 9825 (7351-13311) | 101.66 (73.48-140.89) | 8872 (6819-11833) | 108.01 (78.35-147.47) | 0.32 (0.28-0.35) |
| Burkina Faso | 7858 (5360-11708) | 114.89 (81.07-163.87) | 20278 (13705-30303) | 119.68 (86.15-169.53) | 0.15 (0.14-0.16) |
| Burundi | 3957 (2626-5851) | 96.08 (65.56-136.91) | 9468 (6232-14507) | 94.33 (64.28-136.11) | -0.06 (-0.07--0.05) |
| C?te d'Ivoire | 325 (225-474) | 118.48 (85.45-163.16) | 696 (502-971) | 122.13 (89.48-167.18) | 0.1 (0.09-0.11) |
| Cabo Verde | 11570 (7611-17071) | 151.63 (104.89-217.02) | 24327 (16311-36047) | 149.27 (103.71-216.83) | -0.09 (-0.11--0.07) |
| Cambodia | 11432 (7995-15965) | 150.3 (113.02-197.34) | 41101 (29903-56600) | 170.13 (132.04-223.51) | 0.52 (0.39-0.66) |
| Cameroon | 30841 (21352-45272) | 102.19 (70.13-149.8) | 46708 (34352-65167) | 107.04 (75.64-153.27) | 0.24 (0.2-0.28) |
| Canada | 2987 (2094-4250) | 148.57 (107.17-199.96) | 6399 (4478-9106) | 150.94 (108.49-203.8) | 0.04 (0.03-0.06) |
| Central African Republic | 4982 (3431-7212) | 116.38 (84.07-163.33) | 14409 (9748-21051) | 122.22 (88.81-169.94) | 0.15 (0.15-0.16) |
| Chad | 12197 (8437-17618) | 95.87 (67.76-135.02) | 20040 (14184-28304) | 93.43 (65.37-133.08) | 0.06 (0.02-0.11) |
| Chile | 1253432 (1126823-1398841) | 109.57 (98.58-121.6) | 1610288 (1424197-1795854) | 94.21 (83.71-104.86) | -0.39 (-0.48--0.31) |
| China | 36903 (25953-52493) | 131.48 (96.48-183.93) | 67639 (49332-94296) | 127.01 (91.9-176.95) | 0 (-0.07-0.07) |
| Colombia | 331 (217-482) | 95.29 (65.3-136.05) | 642 (423-943) | 92.81 (62.69-134.96) | -0.09 (-0.11--0.08) |
| Comoros | 2731 (1879-4008) | 152.01 (110.46-212.11) | 6970 (4855-10058) | 149.62 (109.05-209.46) | -0.07 (-0.08--0.06) |
| Congo | 22 (16-33) | 134.58 (95.54-193.49) | 28 (20-41) | 142.01 (100-206.81) | 0.14 (0.13-0.15) |
| Cook Islands | 3922 (2817-5482) | 151.71 (114.32-204.23) | 8038 (6024-10825) | 153.88 (114.98-207.69) | 0.11 (0.08-0.15) |
| Costa Rica | 5537 (4204-7426) | 101.28 (74.95-137.52) | 5337 (4189-6943) | 99.15 (73.93-134.39) | -0.09 (-0.12--0.06) |
| Croatia | 11607 (8270-16704) | 104.24 (74.72-148.86) | 13854 (10224-19478) | 103.39 (74.21-147.53) | 0 (-0.03-0.03) |
| Cuba | 795 (600-1067) | 97.86 (73.71-130.6) | 1583 (1220-2107) | 92.59 (69.92-126.83) | -0.14 (-0.17--0.12) |
| Cyprus | 11856 (9046-16607) | 104.76 (78.54-147.72) | 12902 (10121-17792) | 97.71 (73.18-137.81) | -0.14 (-0.17--0.11) |
| Czechia | 11366 (7821-15858) | 128.02 (95.76-173.92) | 28783 (20326-40251) | 130.05 (96.83-177.41) | 0.05 (0.04-0.06) |
| Democratic People's Republic of Korea | 22390 (16215-32125) | 111.3 (81.12-156.76) | 32843 (23974-45303) | 107.03 (78.64-149.39) | -0.23 (-0.26--0.2) |
| Democratic Republic of the Congo | 39932 (27987-56158) | 145.29 (108.2-192.06) | 100563 (71958-141023) | 147.8 (111.01-194.6) | -0.04 (-0.08--0.01) |
| Denmark | 5792 (4414-7554) | 94.21 (70.54-125.87) | 7106 (5550-9152) | 95.84 (72.67-129.99) | 0.11 (0.08-0.14) |
| Djibouti | 296 (198-468) | 90.66 (63.77-132.06) | 1121 (759-1718) | 95.12 (66.66-141.36) | 0.18 (0.17-0.19) |
| Dominica | 89 (65-122) | 134.51 (98.66-180.55) | 109 (82-144) | 149.77 (112.52-198.46) | 0.37 (0.36-0.38) |
| Dominican Republic | 6606 (4599-9153) | 108.84 (78.87-150.26) | 13143 (9675-18336) | 117.72 (87.14-163.8) | 0.29 (0.26-0.33) |
| Ecuador | 8768 (6098-12520) | 102.11 (73.69-138.89) | 19750 (14340-26596) | 109.33 (79.92-147.38) | 0.31 (0.27-0.35) |
| Egypt | 62771 (41574-94653) | 133.44 (91.69-190.75) | 134350 (91071-198730) | 139.69 (95.86-201.65) | 0.15 (0.13-0.17) |
| El Salvador | 4943 (3438-6936) | 116.22 (85.67-157.3) | 9039 (6746-12255) | 141.44 (106.83-191.32) | 0.99 (0.82-1.17) |
| Equatorial Guinea | 443 (307-604) | 143.61 (104.15-192.07) | 1752 (1205-2487) | 148.79 (107.02-198.12) | 0.13 (0.12-0.14) |
| Eritrea | 2231 (1476-3268) | 89.3 (63.01-130.57) | 5037 (3396-7688) | 91.15 (63.86-133.91) | 0.08 (0.07-0.09) |
| Estonia | 2447 (1754-3626) | 142.05 (100.73-217.41) | 2485 (1934-3526) | 150.48 (109.97-221.84) | 0.33 (0.28-0.38) |
| Eswatini | 771 (536-1095) | 141.78 (103.67-187.71) | 1492 (1038-2056) | 153.39 (112.66-203.85) | 0.29 (0.27-0.3) |
| Ethiopia | 36366 (29218-45312) | 96.25 (79.54-116.34) | 76074 (60878-96869) | 88.78 (72.67-109.92) | -0.28 (-0.31--0.25) |
| Fiji | 994 (700-1464) | 152.43 (113.61-213.04) | 1521 (1128-2114) | 167.16 (125.06-231.01) | 0.31 (0.29-0.34) |
| Finland | 4998 (3810-6953) | 84.76 (63.75-119.53) | 5983 (4644-7943) | 82.28 (62.26-115.32) | 0.01 (-0.04-0.07) |
| France | 48311 (37203-64947) | 73.12 (55.94-100.16) | 62562 (48667-81182) | 72.31 (54.35-98.82) | -0.04 (-0.12-0.04) |
| Gabon | 1199 (869-1618) | 153.04 (115.06-199.68) | 2456 (1799-3334) | 154.95 (116.27-205.93) | 0.03 (0.01-0.05) |
| Gambia | 843 (566-1228) | 119.6 (85.84-166.41) | 2312 (1554-3392) | 124.57 (88.37-175.72) | 0.14 (0.12-0.16) |
| Georgia | 10012 (7258-13909) | 172.86 (124.81-241.5) | 7292 (5461-9998) | 175.4 (128.87-245.57) | 0.04 (0.01-0.07) |
| Germany | 87825 (68397-114011) | 89.24 (68.24-117.25) | 104447 (82597-130816) | 88.29 (67.66-114.66) | -0.15 (-0.25--0.06) |
| Ghana | 9978 (6410-15612) | 88.32 (59.51-129.6) | 27693 (18046-42685) | 94.08 (63.45-139.13) | 0.22 (0.19-0.26) |
| Greece | 11885 (9263-16000) | 98.05 (74.37-132.88) | 13028 (10328-17066) | 93.08 (70.67-129.15) | -0.12 (-0.17--0.08) |
| Greenland | 60 (42-93) | 106.82 (76.88-159.73) | 67 (50-97) | 105.15 (76.55-159.15) | -0.07 (-0.1--0.04) |
| Grenada | 99 (71-139) | 129.57 (96.37-177.7) | 156 (116-213) | 140.77 (104.35-193.16) | 0.29 (0.27-0.3) |
| Guam | 169 (116-265) | 133.83 (95.59-202.63) | 274 (204-407) | 159.29 (115.18-235.42) | 0.6 (0.58-0.63) |
| Guatemala | 8342 (5688-11625) | 134.73 (100.23-179.32) | 22583 (16206-31136) | 154.66 (114.27-206.7) | 0.53 (0.49-0.56) |
| Guinea | 5683 (3997-8240) | 123.25 (90.93-173.84) | 12810 (8856-19091) | 127.42 (94.24-180.56) | 0.11 (0.1-0.12) |
| Guinea-Bissau | 975 (657-1380) | 132.2 (97.8-176.09) | 2043 (1390-2881) | 130.09 (95.6-171.93) | -0.08 (-0.1--0.07) |
| Guyana | 800 (563-1120) | 121.02 (89.2-164.02) | 999 (743-1366) | 131.75 (99.07-179.16) | 0.3 (0.29-0.32) |
| Haiti | 6126 (4406-8668) | 119.05 (89.03-164.46) | 14135 (10259-19921) | 121.45 (90.16-165.86) | 0.1 (0.09-0.1) |
| Honduras | 4664 (3244-6622) | 136.44 (100.95-182.5) | 12311 (8858-17079) | 137.51 (102.27-183.95) | 0.07 (0.05-0.1) |
| Hungary | 10939 (8151-15224) | 94.76 (69.35-134.32) | 10526 (8020-14529) | 89.43 (65.41-126.72) | -0.15 (-0.17--0.14) |
| Iceland | 191 (144-260) | 71.79 (53.85-96.86) | 315 (241-416) | 74.8 (55.56-101.06) | 0.23 (0.18-0.29) |
| India | 1187299 (1063089-1324639) | 161.39 (145.49-178.76) | 2315370 (2076305-2578409) | 160.42 (144.21-178.41) | 0.01 (-0.04-0.06) |
| Indonesia | 272084 (232021-320973) | 172.01 (148.94-200.65) | 511081 (443631-598316) | 173.75 (151.33-202.08) | -0.05 (-0.09--0.02) |
| Iran (Islamic Republic of) | 68437 (61362-76863) | 146.62 (132.42-163.34) | 124861 (112234-140290) | 135.37 (121.89-150.64) | -0.25 (-0.29--0.21) |
| Iraq | 21894 (14431-33869) | 150 (102.92-217.35) | 55748 (36808-84711) | 144.65 (101.42-211.58) | -0.19 (-0.22--0.15) |
| Ireland | 4364 (3255-5746) | 117.86 (87.89-157.46) | 7072 (5452-9167) | 118.84 (88.79-157.85) | -0.07 (-0.13--0.02) |
| Israel | 4997 (3657-6534) | 104.64 (76.84-136.57) | 10184 (7604-13127) | 100.78 (74.28-133.46) | -0.17 (-0.2--0.15) |
| Italy | 66891 (58918-76272) | 100.44 (88.58-116.56) | 70775 (62839-79125) | 92.48 (82.58-104.27) | -0.25 (-0.28--0.22) |
| Jamaica | 2456 (1700-3496) | 116.06 (83.81-161.44) | 3680 (2735-5149) | 120.74 (89.23-168.34) | 0.19 (0.16-0.22) |
| Japan | 146823 (131588-162679) | 100.42 (90.62-111.8) | 165918 (149670-183122) | 96.44 (86.23-108.23) | -0.16 (-0.23--0.09) |
| Jordan | 4133 (2729-6039) | 141.83 (101.06-199) | 16633 (11481-23754) | 139.1 (98.98-194.6) | -0.14 (-0.19--0.09) |
| Kazakhstan | 28367 (20139-39243) | 181.17 (131.82-247.05) | 33925 (24717-46668) | 175.95 (127.73-241.78) | -0.1 (-0.11--0.08) |
| Kenya | 14174 (12616-15787) | 87.93 (79.07-97.12) | 37654 (33673-42209) | 90.27 (81.06-100.13) | 0.07 (0.06-0.09) |
| Kiribati | 94 (66-135) | 153.53 (110.65-214.03) | 182 (128-257) | 165.51 (118.08-229.27) | 0.25 (0.23-0.26) |
| Kuwait | 2240 (1555-3258) | 136.92 (99.42-189.1) | 6823 (4741-9979) | 126.86 (89.26-180.43) | -0.3 (-0.33--0.26) |
| Kyrgyzstan | 7749 (5319-10773) | 195.74 (140.34-268.15) | 11530 (8153-15983) | 177.61 (128.3-241.12) | -0.37 (-0.39--0.34) |
| Lao People's Democratic Republic | 5821 (4074-8299) | 181.75 (131.28-260.47) | 11922 (8399-17899) | 173.97 (124.6-254.73) | -0.2 (-0.22--0.17) |
| Latvia | 4112 (2932-5916) | 139.24 (97.68-201.51) | 3345 (2485-4662) | 143.51 (102.44-209.58) | 0.18 (0.16-0.2) |
| Lebanon | 3550 (2400-5265) | 130.02 (89.73-190.66) | 7867 (5357-11919) | 130.78 (89.53-195.53) | 0.02 (-0.03-0.07) |
| Lesotho | 1472 (1036-2038) | 128.66 (94.14-173.43) | 2320 (1644-3195) | 144.27 (106.9-192.26) | 0.4 (0.39-0.41) |
| Liberia | 2335 (1601-3298) | 126.25 (93.65-169.41) | 5541 (3882-8033) | 126.52 (93.63-171.72) | 0.02 (0.01-0.03) |
| Libya | 4447 (3048-6746) | 133.02 (96.16-195.35) | 10185 (7257-15456) | 136.01 (97.76-203.83) | 0.08 (0.06-0.1) |
| Lithuania | 5787 (4106-8227) | 145.27 (101.92-208.53) | 4975 (3654-6862) | 146.39 (103.29-207.73) | 0.19 (0.15-0.24) |
| Luxembourg | 459 (345-629) | 101.83 (75.7-143.91) | 781 (590-1066) | 96.45 (71.43-139.05) | -0.23 (-0.27--0.2) |
| Madagascar | 8214 (5274-12179) | 91.28 (61.81-133.38) | 20218 (12744-30980) | 89.08 (58.98-134.43) | -0.09 (-0.12--0.07) |
| Malawi | 7078 (4594-10826) | 96.64 (67.64-141.91) | 14641 (9420-23038) | 96.86 (66.25-148.37) | 0.01 (0-0.02) |
| Malaysia | 25467 (17758-36867) | 171.44 (126.43-240.06) | 58109 (41184-85291) | 175.55 (126.9-251.78) | 0.2 (0.14-0.27) |
| Maldives | 274 (185-419) | 174.55 (122.39-260.14) | 953 (652-1477) | 163.72 (116.16-243.82) | -0.27 (-0.32--0.22) |
| Mali | 8287 (5838-11776) | 127.58 (94.54-176.12) | 22493 (15498-33826) | 130.11 (97.27-181.54) | 0.05 (0.05-0.06) |
| Malta | 401 (312-516) | 99.12 (76.85-128.99) | 574 (454-728) | 97.56 (75.9-126.76) | -0.1 (-0.12--0.08) |
| Marshall Islands | 48 (34-68) | 147.01 (109.47-199.34) | 86 (62-119) | 162.46 (120.89-218.08) | 0.33 (0.31-0.34) |
| Mauritania | 2022 (1405-2845) | 128.4 (94.21-173.71) | 4145 (2933-5802) | 120.67 (88.5-164.11) | -0.26 (-0.28--0.23) |
| Mauritius | 1891 (1274-2714) | 183.07 (128.03-256.92) | 3249 (2384-4467) | 214.56 (151.43-303.4) | 0.55 (0.53-0.58) |
| Mexico | 112228 (102219-124022) | 164.21 (150.61-180.93) | 240945 (220782-264808) | 178.51 (163.58-196.37) | 0.57 (0.45-0.7) |
| Micronesia (Federated States of) | 128 (90-181) | 157.74 (119.5-218.18) | 175 (130-239) | 177.63 (133.99-238.77) | 0.39 (0.36-0.42) |
| Monaco | 37 (29-49) | 88.49 (66.02-121.52) | 47 (37-61) | 87.81 (64.43-121.24) | -0.03 (-0.05--0.02) |
| Mongolia | 3665 (2456-5230) | 205.62 (148.35-278.74) | 5946 (4320-8204) | 184.14 (135.75-252.9) | -0.42 (-0.44--0.39) |
| Montenegro | 707 (520-989) | 109.77 (80.91-153.3) | 735 (553-1029) | 103.89 (76.49-148.11) | -0.2 (-0.23--0.16) |
| Morocco | 28166 (18362-43894) | 127.17 (86.56-195.99) | 48331 (32435-74423) | 126.32 (85.28-194.63) | -0.02 (-0.05-0.02) |
| Mozambique | 9613 (6489-14456) | 93.75 (65.86-137.86) | 23087 (15189-35420) | 98.33 (68.3-146.74) | 0.17 (0.16-0.19) |
| Myanmar | 59765 (40737-87845) | 172.38 (123.08-249.02) | 92270 (63887-137951) | 164.16 (115.01-243.64) | -0.18 (-0.2--0.16) |
| Namibia | 1359 (942-1909) | 134.11 (97.34-181.77) | 2773 (1923-3888) | 132.31 (95.64-178.97) | -0.05 (-0.07--0.03) |
| Nauru | 13 (9-19) | 159.01 (115.24-217.44) | 17 (12-24) | 175.46 (126.12-243.87) | 0.33 (0.31-0.35) |
| Nepal | 29655 (21227-42432) | 186.88 (140.41-248.52) | 59290 (42989-80497) | 197.76 (147.68-262.46) | -0.17 (-0.3--0.03) |
| Netherlands | 17996 (13469-24845) | 106.09 (78.79-145.54) | 21836 (16911-29446) | 100.9 (75.41-141.96) | -0.29 (-0.35--0.24) |
| New Zealand | 3203 (2484-4168) | 88.46 (68.62-115.22) | 5559 (4433-7099) | 92.02 (72.93-118.75) | 0.07 (0.02-0.13) |
| Nicaragua | 5160 (3572-7292) | 178.08 (133.73-230.8) | 13244 (9800-17256) | 206.72 (155.68-263.93) | 0.43 (0.4-0.46) |
| Niger | 6415 (4403-9417) | 115 (84.7-160.96) | 18428 (12601-27599) | 112.51 (82.79-160.66) | -0.09 (-0.11--0.07) |
| Nigeria | 109833 (99301-121160) | 161.34 (145.92-176.94) | 267905 (242266-296487) | 158.88 (142.99-174.73) | 0.01 (-0.03-0.04) |
| Niue | 3 (2-5) | 157.26 (114.58-218.79) | 3 (2-4) | 171.59 (125.59-237.65) | 0.28 (0.26-0.3) |
| North Macedonia | 2468 (1861-3294) | 121.56 (91.92-161.11) | 3045 (2347-4070) | 116.21 (87.18-154.25) | -0.13 (-0.15--0.1) |
| Northern Mariana Islands | 74 (52-103) | 169.36 (126.85-226.08) | 95 (71-129) | 180.36 (134.72-242.88) | 0.18 (0.16-0.19) |
| Norway | 4585 (4056-5220) | 91.36 (80.61-104.19) | 6443 (5693-7325) | 96.06 (84.39-109.98) | 0.3 (0.24-0.35) |
| Oman | 2101 (1397-3273) | 128.17 (91.89-188.41) | 6860 (4673-10348) | 137.97 (98.96-201.27) | 0.26 (0.23-0.29) |
| Pakistan | 126027 (98512-163885) | 142.85 (115.46-179.6) | 340289 (270907-432488) | 160.89 (130.51-198.43) | 0.35 (0.33-0.36) |
| Palau | 23 (17-32) | 161.03 (120.6-213.42) | 38 (29-50) | 180.52 (135.53-238.51) | 0.36 (0.35-0.38) |
| Palestine | 2343 (1431-3519) | 147.74 (98.3-214.23) | 6422 (4170-9600) | 140.04 (93.7-211.5) | -0.24 (-0.29--0.19) |
| Panama | 2925 (2131-3936) | 140.74 (107.11-182.46) | 6622 (5097-8767) | 150.96 (115.71-200.08) | 0.34 (0.28-0.4) |
| Papua New Guinea | 3876 (2674-5606) | 120.52 (86.26-170.73) | 11439 (7792-16680) | 129.38 (92.1-184.1) | 0.23 (0.21-0.25) |
| Paraguay | 3743 (2661-5365) | 115.77 (85.34-160.98) | 8687 (6305-12470) | 123.7 (91.57-175.14) | 0.22 (0.21-0.24) |
| Peru | 19294 (13561-27842) | 101.52 (74.45-141.6) | 35569 (25914-51713) | 95.95 (70.18-138.79) | -0.13 (-0.15--0.11) |
| Philippines | 88497 (79038-97735) | 175.39 (157.32-193.05) | 197202 (177175-217328) | 181.62 (163.43-199.8) | 0.02 (-0.01-0.06) |
| Poland | 49370 (44082-55046) | 123.71 (110.14-138.12) | 48220 (43422-54027) | 102.53 (91.73-115.39) | -0.57 (-0.61--0.54) |
| Portugal | 11321 (8665-15024) | 100.07 (76.36-136.02) | 12715 (9937-17093) | 88.73 (67.2-123.04) | -0.13 (-0.23--0.03) |
| Puerto Rico | 4614 (3491-6295) | 127.15 (96.37-172.29) | 5187 (4002-7041) | 130.57 (98.78-179.23) | 0.12 (0.06-0.18) |
| Qatar | 672 (459-967) | 149.34 (107.6-207.15) | 5024 (3467-7258) | 144.22 (105.65-201.73) | -0.16 (-0.2--0.12) |
| Republic of Korea | 43810 (30638-65263) | 100.27 (72.83-144.56) | 60906 (44830-89738) | 89.03 (63.77-132.12) | -0.68 (-0.78--0.58) |
| Republic of Moldova | 7131 (5123-10145) | 157.89 (113.49-222.43) | 7498 (5549-10415) | 167.44 (120.62-236.7) | 0.18 (0.14-0.22) |
| Romania | 24390 (18125-32878) | 98.61 (72.84-134.73) | 22860 (18118-29273) | 95.92 (72.75-129.4) | 0.04 (-0.04-0.11) |
| Russian Federation | 225367 (205834-245409) | 138.47 (126.53-151.11) | 218669 (198281-240662) | 127.6 (115.07-140.83) | -0.28 (-0.29--0.27) |
| Rwanda | 5216 (3420-8007) | 97.26 (67.15-142.04) | 9992 (6316-15841) | 88.94 (58.89-133.92) | -0.3 (-0.32--0.28) |
| Saint Kitts and Nevis | 51 (36-73) | 134.45 (97.09-189.59) | 91 (66-130) | 134.81 (95.77-193.99) | -0.03 (-0.05--0.01) |
| Saint Lucia | 148 (104-211) | 125.8 (91.82-174.67) | 272 (203-370) | 133.39 (97.62-184.24) | 0.22 (0.2-0.24) |
| Saint Vincent and the Grenadines | 111 (79-155) | 117.65 (87.24-160.3) | 160 (121-217) | 129.12 (96.35-176.08) | 0.32 (0.31-0.32) |
| Samoa | 203 (141-295) | 152.6 (111.43-209.94) | 301 (215-421) | 162.93 (119.57-225.29) | 0.19 (0.18-0.21) |
| San Marino | 25 (19-34) | 90.38 (68.2-123.13) | 39 (31-52) | 88.38 (66.07-121.34) | -0.11 (-0.13--0.08) |
| Sao Tome and Principe | 132 (88-190) | 143.18 (103.01-199.04) | 284 (200-405) | 149.41 (108.4-208.59) | 0.13 (0.1-0.15) |
| Saudi Arabia | 18779 (13129-29248) | 145.32 (108.5-208.63) | 62633 (46096-93673) | 149.44 (110.3-213.07) | 0.02 (-0.02-0.05) |
| Senegal | 6502 (4288-9358) | 115.24 (81.35-159.86) | 13995 (9216-20608) | 107.99 (74.86-154.83) | -0.26 (-0.3--0.22) |
| Serbia | 9813 (7193-13492) | 93.72 (68.02-130.03) | 9553 (7046-13148) | 86.37 (61.53-122.53) | -0.33 (-0.35--0.3) |
| Seychelles | 111 (76-163) | 168.66 (119.33-243.54) | 216 (154-313) | 183.53 (128.65-263.07) | 0.28 (0.27-0.3) |
| Sierra Leone | 4034 (2797-5908) | 124.57 (92.04-172.35) | 8982 (6248-13309) | 127.25 (92.44-178.72) | 0.07 (0.05-0.08) |
| Singapore | 4411 (3096-6551) | 139.06 (99.73-199.24) | 10719 (7939-15367) | 145.87 (106.84-207.66) | -0.34 (-0.5--0.18) |
| Slovakia | 5780 (4293-7823) | 104.98 (76.99-141.98) | 6173 (4659-8199) | 94.68 (70.4-128.1) | -0.33 (-0.38--0.29) |
| Slovenia | 2220 (1704-2982) | 101.34 (77.15-137.93) | 2626 (2059-3428) | 98.52 (74.68-133.53) | -0.26 (-0.32--0.19) |
| Solomon Islands | 380 (262-543) | 152.11 (110.77-207.71) | 924 (642-1294) | 158.74 (115.89-215.98) | 0.12 (0.1-0.13) |
| Somalia | 5208 (3509-7905) | 90.7 (64.98-131.58) | 13940 (9340-22063) | 90.73 (64.59-134) | 0.02 (0.01-0.03) |
| South Africa | 44688 (37928-52222) | 146.38 (125.53-169.03) | 85057 (72335-98737) | 148.29 (126.39-172.53) | 0 (-0.01-0.01) |
| South Sudan | 4017 (2729-6071) | 90.53 (64.58-130.98) | 6650 (4493-10089) | 90.76 (64.64-133.19) | 0.04 (0.03-0.05) |
| Spain | 41695 (31892-54516) | 94.98 (72.39-124.94) | 50244 (38810-64145) | 82.22 (61.97-110.33) | -0.1 (-0.2-0.01) |
| Sri Lanka | 25537 (16881-39119) | 164.1 (113.04-240.34) | 40674 (28453-59807) | 166.66 (114.15-248.81) | 0.06 (0.05-0.08) |
| Sudan | 20240 (13626-31106) | 129.87 (92.18-188.02) | 49567 (33097-78330) | 135.13 (95.48-202.01) | 0.1 (0.08-0.12) |
| Suriname | 445 (317-610) | 125.21 (94.29-167.84) | 812 (612-1092) | 133.87 (99.45-182.72) | 0.24 (0.23-0.25) |
| Sweden | 10620 (8412-14044) | 101.94 (78.79-136.79) | 13039 (10351-16886) | 99.49 (75.82-136.24) | 0.13 (0.07-0.2) |
| Switzerland | 8074 (6017-11189) | 97.81 (72.02-136.98) | 11350 (8633-15291) | 97.14 (71.78-137.88) | -0.03 (-0.07-0.01) |
| Syrian Arab Republic | 15117 (9850-22273) | 149.56 (102.4-214.33) | 18832 (12679-27849) | 133.72 (89.71-198.5) | -0.41 (-0.45--0.36) |
| Taiwan (Province of China) | 23046 (16600-33795) | 114.43 (84.46-163.27) | 32521 (24602-45439) | 106.12 (77.23-154.33) | -0.21 (-0.26--0.16) |
| Tajikistan | 6577 (4580-9406) | 157.71 (113.01-220.31) | 14225 (10032-19947) | 157.46 (114.42-215.27) | -0.02 (-0.04--0.01) |
| Thailand | 93863 (64776-138145) | 177.15 (125.87-245.95) | 155633 (114605-216677) | 182.06 (131-259.4) | -0.01 (-0.08-0.06) |
| Timor-Leste | 952 (654-1419) | 159.84 (113.74-233.44) | 1844 (1264-2789) | 163.49 (116.26-242.6) | 0.06 (0.04-0.08) |
| Togo | 3092 (2095-4538) | 120.05 (86.06-166.74) | 8314 (5743-12163) | 121.04 (86.75-167.89) | -0.02 (-0.03-0) |
| Tokelau | 2 (1-3) | 140.63 (101.87-192.12) | 2 (2-3) | 151.42 (109.14-208.9) | 0.24 (0.23-0.25) |
| Tonga | 114 (77-164) | 146.93 (104.77-209.81) | 150 (105-216) | 160.5 (115.47-229.38) | 0.31 (0.29-0.33) |
| Trinidad and Tobago | 1310 (959-1783) | 118.08 (88.54-159) | 2025 (1540-2714) | 128.03 (95.68-172.16) | 0.28 (0.27-0.29) |
| Tunisia | 9752 (6890-14096) | 132.56 (95.63-184.28) | 16670 (11941-23656) | 129.96 (93.49-185.13) | -0.13 (-0.17--0.1) |
| Türkiye | 5854 (4173-8362) | 190.33 (141.77-265.41) | 10047 (7441-13678) | 197.61 (148.19-265.03) | 0.14 (0.13-0.15) |
| Turkmenistan | 13 (9-18) | 148.8 (108.48-212.3) | 19 (14-27) | 161.75 (121.07-226.97) | 0.25 (0.24-0.26) |
| Tuvalu | 81605 (54678-121154) | 158.04 (109.45-229.96) | 129025 (89028-193772) | 141.12 (96.92-211.43) | -0.24 (-0.34--0.13) |
| Uganda | 11372 (7422-17767) | 94.56 (65.74-140.64) | 29461 (18883-46365) | 94.03 (64.24-142.57) | -0.01 (-0.03-0) |
| Ukraine | 77064 (52692-117365) | 131.17 (88.22-200.66) | 74712 (52645-110421) | 137.62 (92.65-208.38) | 0.15 (0.13-0.17) |
| United Arab Emirates | 3086 (2097-4692) | 166.98 (120.29-233) | 20239 (14912-28331) | 165.47 (121.22-228.88) | -0.06 (-0.09--0.03) |
| United Kingdom | 71830 (64883-78666) | 108.06 (97.66-118.09) | 85568 (77298-93873) | 100.78 (91.66-110.78) | -0.3 (-0.4--0.2) |
| United Republic of Tanzania | 17066 (11327-25518) | 87.92 (62.41-128.06) | 43358 (28783-68086) | 92.58 (64.9-140.31) | 0.34 (0.28-0.41) |
| United States of America | 332505 (300555-365885) | 120.16 (108.7-132.08) | 520141 (475875-566622) | 131.55 (120.19-143.73) | 0.41 (0.31-0.5) |
| United States Virgin Islands | 118 (84-164) | 113 (81.39-156) | 122 (92-162) | 117.4 (84.84-162.46) | 0.13 (0.11-0.15) |
| Uruguay | 2973 (2138-4192) | 91.44 (64.9-129.43) | 3550 (2525-4984) | 91.71 (64.36-130.11) | -0.09 (-0.12--0.05) |
| Uzbekistan | 34077 (23862-47976) | 193.88 (144.19-260.37) | 69032 (50780-93765) | 202.98 (151.09-273.16) | 0.19 (0.17-0.21) |
| Vanuatu | 163 (110-235) | 143.63 (101.25-197.67) | 434 (300-622) | 162.03 (115.54-225.88) | 0.4 (0.39-0.42) |
| Venezuela (Bolivarian Republic of) | 21378 (15324-30250) | 138.31 (104.14-189.15) | 39976 (30474-53552) | 138.99 (104.82-188.6) | 0.08 (0.04-0.12) |
| Viet Nam | 77555 (49662-124604) | 137.95 (91.93-211.92) | 150840 (97411-236704) | 139.29 (90.45-220.26) | 0.15 (0.09-0.21) |
| Yemen | 11211 (7443-16858) | 117.71 (82.38-170.42) | 32534 (21618-49891) | 119.14 (83.14-174.41) | -0.02 (-0.06-0.02) |
| Zambia | 5407 (3602-7871) | 92.91 (65.33-129.45) | 14491 (9677-21390) | 94.72 (67.78-134.72) | 0.1 (0.08-0.12) |
| Zimbabwe | 9363 (6663-13958) | 135.15 (100.11-186.92) | 17095 (12100-25020) | 142.83 (103.71-199.46) | 0.21 (0.2-0.23) |

| **TableS27: National Burden of chronic kidney disease due to glomerulonephritis: deaths, ASDR, and EAPC (1990–2021).** | | | | | |
| --- | --- | --- | --- | --- | --- |
| Country | **1990** | | **2021** | | **EAPC_95%CI** |
|  | Number_95%UI | ASR | Number_95%UI | ASR |  |
| Afghanistan | 288 (189-451) | 4.06 (2.68-6.62) | 506 (287-879) | 4.33 (2.41-7.95) | 0.28 (0.23-0.33) |
| Albania | 43 (32-56) | 2.07 (1.53-2.74) | 68 (47-95) | 1.69 (1.19-2.32) | -0.6 (-0.9--0.3) |
| Algeria | 220 (147-376) | 1.91 (1.2-3.52) | 739 (531-975) | 2.45 (1.73-3.32) | 1.2 (1-1.4) |
| American Samoa | 0 (0-0) | 0.76 (0.49-1.17) | 1 (0-1) | 1.59 (1.09-2.18) | 2.55 (2.3-2.8) |
| Andorra | 1 (1-1) | 1.84 (1.21-2.71) | 2 (2-3) | 1.35 (0.9-1.96) | -0.65 (-0.88--0.42) |
| Angola | 451 (321-602) | 9.45 (6.72-12.84) | 1246 (906-1709) | 9.26 (6.35-12.65) | -0.16 (-0.26--0.06) |
| Antigua and Barbuda | 2 (1-2) | 3.01 (2.5-3.66) | 5 (4-6) | 4.66 (3.78-5.67) | 2.08 (1.83-2.33) |
| Argentina | 1032 (790-1317) | 3.3 (2.56-4.17) | 1505 (1123-1928) | 2.66 (1.99-3.37) | -0.57 (-0.83--0.31) |
| Armenia | 4 (3-5) | 0.14 (0.11-0.18) | 56 (40-78) | 1.35 (0.98-1.87) | 6.86 (5.61-8.11) |
| Australia | 451 (396-505) | 2.46 (2.13-2.79) | 1286 (956-1648) | 2.51 (1.91-3.16) | 0.53 (0.2-0.87) |
| Austria | 144 (110-182) | 1.22 (0.94-1.5) | 479 (369-614) | 2.11 (1.67-2.61) | 2.91 (2.41-3.42) |
| Azerbaijan | 58 (41-80) | 1 (0.71-1.37) | 149 (99-213) | 1.47 (0.98-2.06) | 1.3 (1.02-1.58) |
| Bahamas | 6 (5-7) | 2.96 (2.42-3.55) | 19 (14-25) | 4.55 (3.4-5.91) | 1.91 (1.69-2.13) |
| Bahrain | 4 (3-6) | 2.94 (1.96-4.29) | 23 (16-30) | 3.48 (2.48-4.77) | 0.13 (-0.21-0.47) |
| Bangladesh | 1692 (1065-2247) | 2.21 (1.75-2.84) | 2179 (1580-3040) | 1.67 (1.23-2.33) | -0.91 (-1.15--0.67) |
| Barbados | 6 (5-7) | 2.2 (1.85-2.6) | 13 (10-18) | 3.04 (2.24-4.11) | 1.72 (1.36-2.07) |
| Belarus | 36 (31-42) | 0.3 (0.26-0.35) | 123 (95-158) | 0.83 (0.65-1.05) | 3.1 (2.22-3.98) |
| Belgium | 180 (133-233) | 1.15 (0.87-1.47) | 317 (220-435) | 1.09 (0.79-1.47) | 0.34 (0.14-0.54) |
| Belize | 4 (4-5) | 3.07 (2.58-3.68) | 20 (16-24) | 5.45 (4.41-6.64) | 2.45 (2.07-2.84) |
| Benin | 192 (153-237) | 7.51 (5.81-9.47) | 480 (366-619) | 7.54 (5.63-9.88) | -0.06 (-0.16-0.05) |
| Bermuda | 1 (1-1) | 1.5 (1.22-1.82) | 2 (1-2) | 1.43 (1.1-1.84) | 0.1 (-0.2-0.41) |
| Bhutan | 7 (4-10) | 2.38 (1.58-3.49) | 17 (11-25) | 2.85 (1.74-4.09) | 0.59 (0.53-0.66) |
| Bolivia (Plurinational State of) | 290 (228-373) | 6.92 (5.3-9.06) | 743 (519-1019) | 8.04 (5.76-11.08) | 0.47 (0.41-0.54) |
| Bosnia and Herzegovina | 72 (55-93) | 1.84 (1.39-2.38) | 112 (83-144) | 1.83 (1.36-2.35) | -0.31 (-0.49--0.13) |
| Botswana | 28 (19-42) | 4.79 (3.22-7.07) | 81 (57-119) | 5.61 (4-8.18) | 0.57 (0.36-0.78) |
| Brazil | 3305 (2833-3817) | 3.1 (2.6-3.64) | 6676 (5361-8122) | 2.72 (2.19-3.3) | -0.49 (-0.64--0.34) |
| Brunei Darussalam | 3 (3-5) | 3.12 (2.23-4.36) | 8 (6-11) | 2.87 (2.09-3.75) | 0.29 (0.07-0.51) |
| Bulgaria | 162 (124-199) | 1.56 (1.25-1.87) | 480 (352-644) | 3.55 (2.66-4.68) | 3.48 (3.01-3.96) |
| Burkina Faso | 395 (309-503) | 7.76 (5.84-9.97) | 962 (735-1217) | 8.62 (6.38-11.11) | 0.43 (0.34-0.51) |
| Burundi | 336 (245-446) | 12.69 (9.07-17.1) | 558 (392-820) | 10.74 (7.24-15.78) | -0.92 (-1.07--0.76) |
| C?te d'Ivoire | 8 (6-10) | 3.04 (2.32-3.79) | 23 (15-30) | 5 (3.16-6.69) | 1.34 (1.11-1.57) |
| Cabo Verde | 103 (72-135) | 1.15 (0.86-1.49) | 150 (101-213) | 1.03 (0.71-1.43) | -0.52 (-0.65--0.39) |
| Cambodia | 361 (260-495) | 6.45 (4.59-9.04) | 993 (645-1430) | 6.08 (4.05-8.85) | 0.55 (0.01-1.1) |
| Cameroon | 291 (225-367) | 0.93 (0.72-1.15) | 926 (703-1192) | 1.28 (1.01-1.59) | 1.44 (1.25-1.63) |
| Canada | 156 (121-198) | 11.62 (8.79-14.88) | 302 (209-444) | 11.45 (7.86-16.29) | -0.04 (-0.08-0.01) |
| Central African Republic | 180 (134-244) | 5.24 (3.76-7.29) | 473 (332-690) | 6.21 (4.24-9.29) | 0.43 (0.25-0.6) |
| Chad | 173 (136-216) | 1.74 (1.36-2.19) | 491 (370-637) | 1.9 (1.45-2.46) | 0.67 (0.29-1.05) |
| Chile | 4940 (3900-6249) | 0.5 (0.4-0.62) | 5320 (3752-7180) | 0.3 (0.22-0.39) | -1.9 (-2--1.79) |
| China | 960 (818-1113) | 4.85 (3.99-5.79) | 1970 (1519-2489) | 3.57 (2.74-4.54) | -0.81 (-0.95--0.68) |
| Colombia | 27 (20-35) | 12.81 (9.43-16.92) | 64 (45-84) | 13.52 (9.35-17.96) | -0.02 (-0.16-0.11) |
| Comoros | 155 (113-201) | 13.27 (9.21-17.19) | 358 (235-492) | 12.44 (8.04-16.75) | -0.4 (-0.54--0.27) |
| Congo | 0 (0-0) | 0.29 (0.2-0.4) | 0 (0-0) | 0.34 (0.24-0.47) | 0.6 (0.51-0.69) |
| Cook Islands | 65 (53-77) | 3.48 (2.8-4.2) | 356 (285-441) | 6.49 (5.18-7.99) | 1.97 (1.33-2.6) |
| Costa Rica | 120 (93-149) | 2.19 (1.71-2.73) | 271 (196-366) | 2.82 (2.07-3.76) | 0.14 (-0.34-0.61) |
| Croatia | 110 (94-128) | 1.02 (0.87-1.2) | 282 (220-364) | 1.64 (1.29-2.06) | 1.95 (1.65-2.26) |
| Cuba | 30 (20-42) | 5.79 (3.87-8.28) | 50 (35-69) | 2.88 (2.06-3.93) | -2.44 (-2.68--2.2) |
[truncated: 181,346 more chars]
