# Supplementary figures and images for: Global burden of chronic kidney disease and risk factors, 1990–2021: an update from the global burden of disease study 2021
Source: Front Public Health. 2025 Jul 24;13:1542329. doi: 10.3389/fpubh.2025.1542329 (PMC12366504; doi:10.3389/fpubh.2025.1542329)

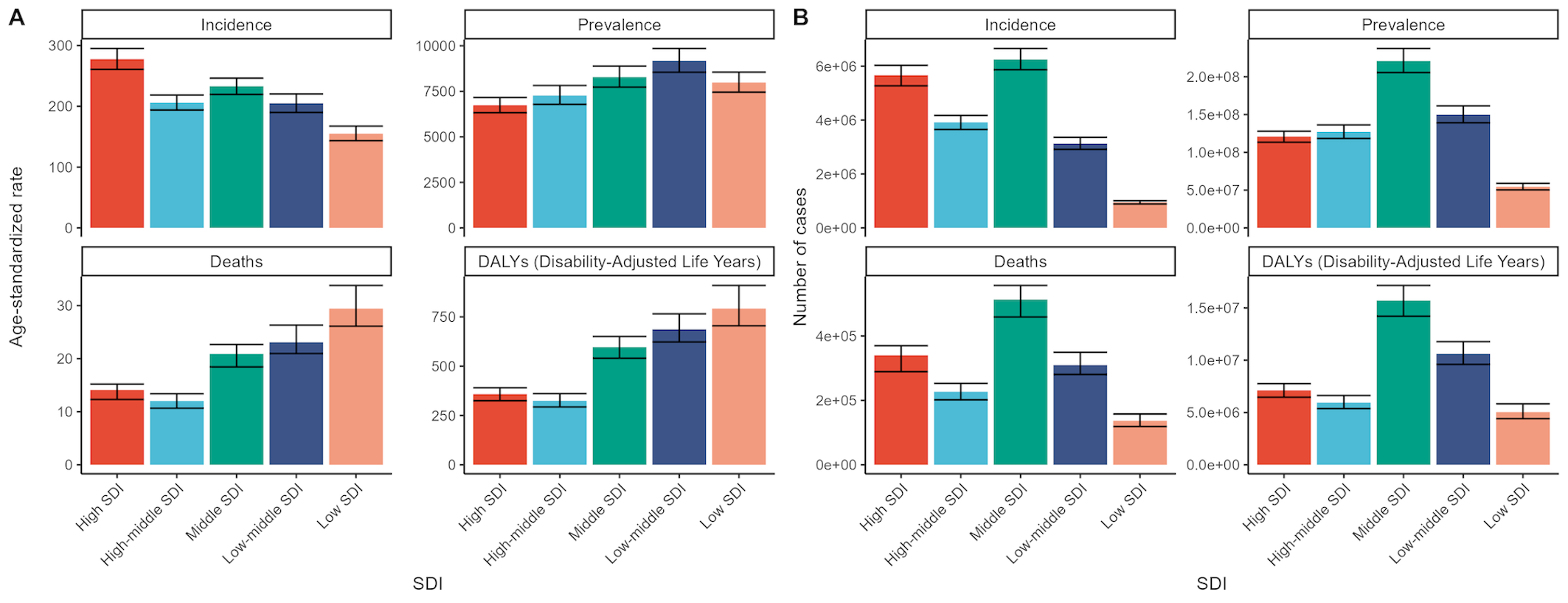

Supplement: Supplementary file 2 [file Image_1.tiff]

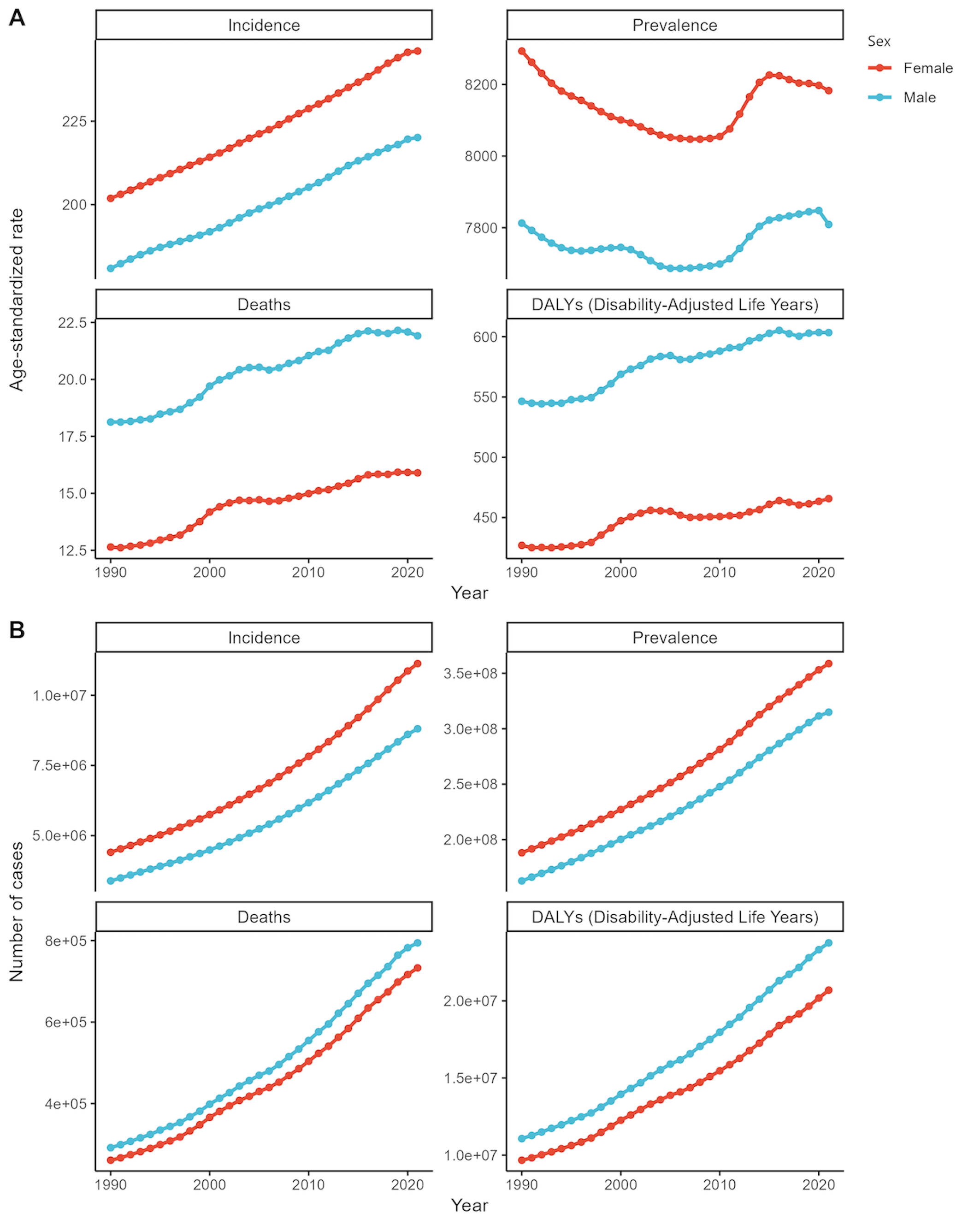

Supplement: Supplementary file 3 [file Image_2.tiff]

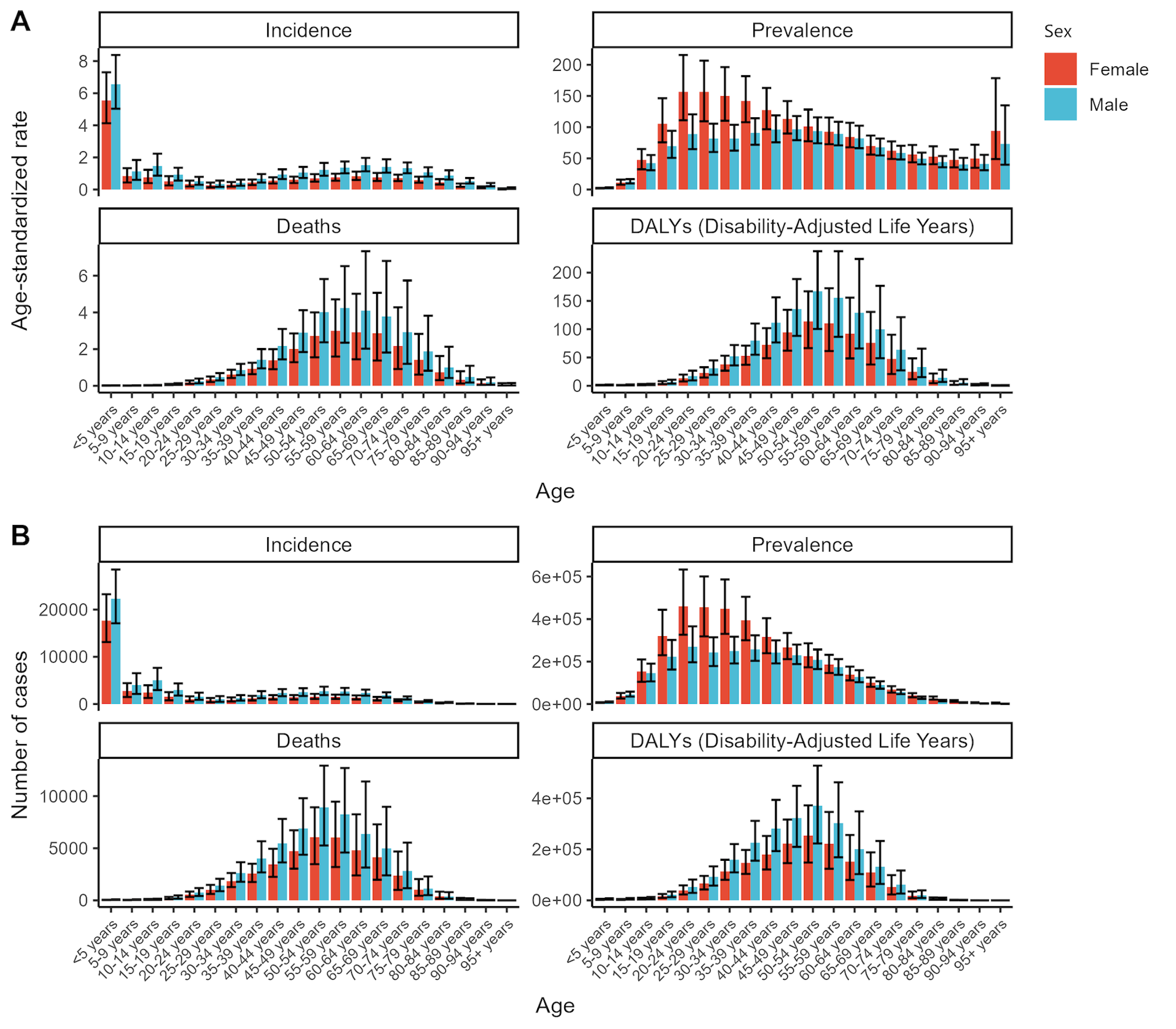

Supplement: Supplementary file 4 [file Image_3.tiff]

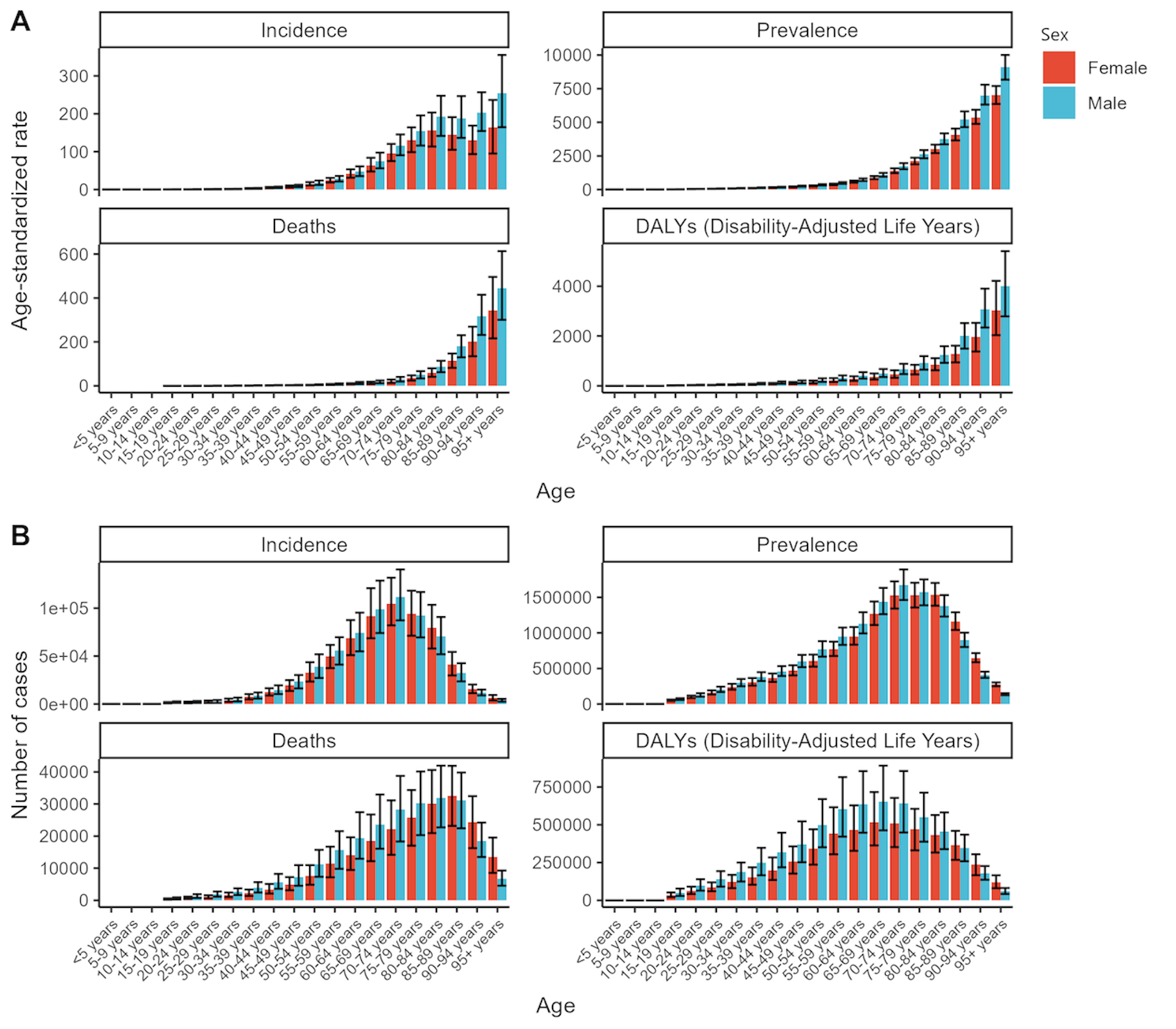

Supplement: Supplementary file 5 [file Image_4.tiff]
